# Public Meets Private: Conversations Between Coca‐Cola and the CDC

**DOI:** 10.1111/1468-0009.12368

**Published:** 2019-01-29

**Authors:** NASON MAANI HESSARI, GARY RUSKIN, MARTIN McKEE, DAVID STUCKLER

**Affiliations:** ^1^ London School of Hygiene and Tropical Medicine; ^2^ U.S. Right to Know; ^3^ Dondena Research Center Bocconi University

**Keywords:** commercial determinants of health, Centers for Disease Control and Prevention, government transparency, public health

## Abstract

**Policy Points**

- There is growing understanding of how manufacturers of harmful products influence health policy. The strategies, approaches, and influences from such manufacturers that are detrimental to health have been termed the “corporate” or “commercial” determinants of health. However, while partnerships with the tobacco industry are clearly unacceptable for public health organizations, ties to other industries continue to be pursued.
- Such partnerships may influence health organizations in a number of ways detrimental to population health. However, with the exception of tobacco industry tactics as revealed by internal documents, we know relatively little about how this influence operates.
- This article uses emails between the Coca‐Cola Company and the Centers for Disease Control and Prevention, which we obtained through Freedom of Information Act requests, to explore the nature of corporate influence, conflicts of interest, and lobbying “in their own words,” and highlights the need for greater transparency and clearer policies on engaging with such industries.

**Context:**

There is a continuing debate about the appropriateness of contacts between manufacturers of some harmful products and health researchers, as well as practitioners and policymakers. Some argue that such contacts may be a means of exerting undue influence, while others present them as an opportunity to pursue shared health goals. This article examines interactions between the Centers for Disease Control and Prevention (CDC) and the Coca‐Cola Company (Coca‐Cola) as revealed by communications obtained through Freedom of Information Act (FOIA) requests.

**Methods:**

We sent 10 US FOIA requests in 2016/2017 for communications between employees at the CDC and Coca‐Cola. We then performed a thematic content analysis of the documents provided.

**Findings:**

Of our 10 FOIA requests, 3 requests are still pending (at the time of this publication); 5 were rejected as too broad or because no records were found; and 3 returned 295 pages from 86 emails. The CDC withheld 102 pages to “protect commercial or financial information which is privileged or confidential.” The returned emails demonstrate three main themes in Coca‐Cola's contact with CDC employees: to gain and expand access, to lobby, and to shift attention and blame away from sugar‐sweetened beverages.

**Conclusions:**

The emails we obtained using FOIA requests reveal efforts by Coca‐Cola to lobby the CDC to advance corporate objectives rather than health, including to influence the World Health Organization. Our findings provide a rare example of the ways in which corporate interests attempt to influence public health practitioners “in their own words,” and they demonstrate a need for clearer policies on avoiding partnerships with manufacturers of harmful products.

Although there is widespread agreement about the importance of not engaging with the tobacco industry, interactions between health researchers, practitioners, and policymakers and the manufacturers of other potentially harmful products are controversial. While some see such interactions as a means to promote dialogue and reduce harm, others draw attention to the influence that these manufacturers exert, for example, through their funding of academic research.^1^, ^2^, ^3^, ^4^, ^5^ There is also growing concern that nonfinancial influence is perhaps just as important, even though it may be harder to detect. Corporations may seek to reframe policy debates, build opposing constituencies, and lobby politicians to avert public health policies that could undermine their profits.^6^ An Australian study showed how the Coca‐Cola Company (Coca‐Cola) and other companies frame obesity debates as being predominantly about individuals and exercise.^7^ The now‐defunct Global Energy Balance Network (GEBN), a US‐based group that focused on lack of exercise as the primary driver of obesity, was disbanded after the media revealed Coca‐Cola's involvement.^8^, ^9^ Such strategies may complement traditional lobbying activities, such as blocking taxes on sugar‐sweetened beverages (SSBs).^10^

Concerns regarding the breadth and potency of such corporate strategies have contributed to an increase in research on what are termed the “corporate determinants of health,”^11^ including describing how manufacturers of harmful products and activities—such as tobacco, alcohol, and gambling—use similar language and tactics when faced with policies that threaten their profits.^12^, ^13^ This research calls for caution in all interactions with these industries, highlighting the risks of corporate access to and influence on public health organizations. As we noted, however, this is not a universal view, and achieving consensus is made more difficult by the relative lack of knowledge of the nonfinancial influence of these corporate actors, with the exception of the tobacco industry, for which we now have a greater evidence base.

Ideally, information on interactions between public bodies and industry should be transparent, with legislators signaling their commitment to this principle by passing freedom‐of‐information laws. Yet in practice, it can be difficult to discover what is happening behind closed doors. We illustrate these challenges by means of a case study, in which we describe the challenges in obtaining information about interactions between two leading players on different sides of the obesity debate in the United States, the Centers for Disease Control and Prevention (CDC) and Coca‐Cola.

This case study is of particular relevance because the CDC has recently faced criticism for its links to manufacturers of unhealthy products, especially those of SSBs.^14^, ^15^, ^16^ In 2016, Barbara Bowman, director of the CDC's Division for Heart Disease and Stroke Prevention, resigned after emails between her and a former Coca‐Cola executive were disclosed.^15^ The emails, obtained from a Colorado Open Records Act request to the University of Colorado, showed that Bowman had advised the former Coca‐Cola and industry association executive on how to influence the director‐general of the World Health Organization (WHO) to stop promoting taxes on sugar.^14^, ^15^, ^16^ Brenda Fitzgerald, who was appointed in July 2017 to head the CDC, had previously been commissioner of the Georgia Department of Public Health from 2011 to 2017, during which time she accepted a US $1 million donation from Coca‐Cola for Georgia Shape, a childhood obesity initiative.^15^

Corporations and individuals may contribute to the CDC either directly or indirectly, via the National Foundation for the Centers for Disease Control and Prevention (CDC Foundation), a nonprofit organization established by Congress in 1992 “to support and carry out activities for the prevention and control of diseases … and for promotion of public health.”^17^ Coca Cola's transparency website reports that between 2010 and 2015, the company donated more than US $1 million to the CDC Foundation, primarily to “build global capacity for NCD prevention.”^18^ The foundation's own records report additional gifts from Coca‐Cola in 2016^19^ and 2017,^20^ although they do not appear on Coca‐Cola's transparency website.^18^ The potential challenges in operating such a foundation were made clear in the bill establishing the CDC Foundation, in which Congress instructed it to prepare bylaws to ensure that its activities would not “compromise, or appear to compromise, the integrity of any governmental program or any officer or employee involved in such program.”^17^ While the CDC Foundation website refers to processes that “safeguard against potential conflicts of interest,”^21^ concerns remain that such funding may affect research and policy. Indeed, studies reporting funding by the SSB industry are significantly more likely to find no association between the consumption of SSBs and obesity.^22^ Moreover, even when researchers have received funding from Coca‐Cola, not all their papers report this conflict of interest.^1^

In this article, we examine interactions between the CDC and Coca‐Cola using Freedom of Information Act (FOIA) requests. We used these requests to reveal exchanges between the CDC and Coca‐Cola “in their own words,” enabling us to examine the attempts by Coca‐Cola–affiliated individuals to influence the CDC and the CDC Foundation on this controversial subject.

## Methods

U.S. Right to Know (USRTK), a nongovernmental organization that investigates the food and agrichemical industries, made a series of FOIA requests for correspondence between employees at the CDC and the CDC Foundation and current or former employees of Coca‐Cola or the International Life Sciences Institute (ILSI). Both individuals and organizations can obtain records from federal agencies unless one of nine exemptions applies, relating to issues such as national security, breach of other federal laws, trade secrets, and invasion of privacy. We made 10 requests between June 26, 2016, and August 15, 2017, for communications between January 1, 2011, and the time of the request between named staff at the CDC and the CDC Foundation and Coca‐Cola or the ILSI. ILSI is an organization that receives funding from Coca‐Cola, was founded by a Coca‐Cola executive, and was involved in the CDC media coverage mentioned earlier. Some of the responses led to follow‐up requests.

We read the documents received in the order of their date, and a qualitative researcher interpreted and thematically coded them. Our choice of themes was inductive, based on a framework developed to examine the political activities of the food industry^6^ and informed by research on the tobacco industry's documentation.^23^, ^24^, ^25^, ^26^ The coding was iterative, allowing us to adapt the framework to reflect emerging corporate strategies. We present here illustrative quotations relating to each activity identified; the online supplementary material includes all the documents we obtained. We further compare these documents to the 2014 CDC ethics guidelines, which state that interactions between the CDC and private entities must be based on “mutual, explicit and transparent benefits for all partners.”^27^ The guidelines also state that staff considering a partnership must ask whether “partnering with the private entity presents a conflict of interest (real or perceived).”^27^

## Results

### FOIA Response Rate

**Figure 1:**
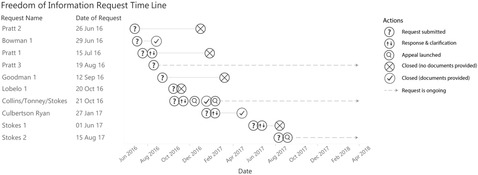
Time Line of FOIA Requests to the CDC Regarding Specific Employees’ Contacts With Coca‐Cola or the International Life Sciences Institute and the CDC's Subsequent Responses

Figure 1 shows the time line of our FOIA requests. As shown, response times varied significantly, from less than two weeks to several months. Three of our requests were initially rejected as being too broad (although the scope of all our requests was similar), and two returned no records. At the time of writing (two years later), three of our requests are still pending. Three of our requests returned 295 pages of communications from 86 emails. The CDC further withheld 105 pages of communications, including 102 pages on the grounds of 5 USC §552(b)(4) (which protects privileged or confidential commercial or financial information).

### Thematic Analysis of Email Documents

The emails offer examples of Coca‐Cola's successful efforts to gain access, lobby policymakers, and frame the debate on diet and obesity. Given the small number of FOIA requests generating material, we report findings by activity, using illustrative quotations.

#### Theme 1: Gaining Access and Influence

The emails we obtained indicate that former Coca‐Cola staff attempted to meet with CDC staff members in order to build relationships with them. Accordingly, on April 3, 2013, Rhona Applebaum, then Coca‐Cola's chief science and health officer and an architect of the Global Energy Balance Network,^8^ contacted the CDC's Janet Collins:

Heartfelt congratulations on being named Director of The Division of Nutr [*sic*], Physical Activity and Obesity at CDC. Once settled would welcome the opportunity to come by and discuss current activities and what more can be done.

Collins replied:

Thanks Rhona. I am delighted to have joined the Division. I have some international travel coming up through mid‐April but would be happy to meet after that. (Supplementary file 1)

Such contacts also enabled the strengthening of institutional ties. On October 27, 2014, Collins asked Applebaum:

I hope you don't mind if I share the fact that a CDC colleague of mine (Maureen Culbertson) is very interested in working at Coca‐Cola … she would be great for external and governmental relations especially on food, beverage and physical activity policy as well as corporate philanthropy.

Applebaum replied:

Many thanks for the CV. I will share internally. (Supplementary file 1)

Emails between Barbara Bowman, then director of the CDC's Division for Heart Disease and Stroke Prevention, and Alex Malaspina, a former Coca‐Cola senior vice president of external affairs and the founding president of ILSI, show their efforts to expand access to and influence at the CDC. On September 22, 2014, Malaspina wrote:

I was very impressed with all you have accomplished and your new responsibilities. I always had such faith in your abilities and your great knowledge in nutrition. I would very much like to see you again and also introduce you to a very delightful and intelligent young lady from Kenya [Wamwari Waichungo, Coca‐Cola vice president for global scientific and regulatory affairs], who for the last year has had my old job at Coke, as Head of SRA.… How is your schedule? If you agree, give me some dates and I will arrange for a nice dinner for the three of us.

Bowman responded:

I'd love to see you and to meet Wamwary [*sic*] Waichungo! … looking forward to getting together. (Supplementary file 2)

This networking subsequently expanded, as following up to arrange the suggested meeting. Malaspina wrote on November 21, 2014, asking to include more Coca‐Cola executives in the meeting:

Is it OK with you, if I also invite two close friends, who would like to meet you. One is Clyde Tuggle, Senior VP in charge of Public Affairs. The other is Ed Hays, who is in charge of Science. (Supplementary file 2)

Later Bowman wrote:

What a lovely time we had on Saturday nights [*sic*], many thanks, Alex, for your hospitality. (Supplementary file 2)

#### Theme 2: Framing Debates on Nutrition, Artificial Sweeteners, and Obesity

We found evidence that meetings between the staffs of the two organizations enabled their sharing of information, although the emails cannot capture the full scope of such meetings. At least one visit was arranged for CDC staff to visit Coca‐Cola's headquarters to “provide an overview of OMHHE's [Office of Minority Health and Health Equity's] priorities, and share other collaborations between the CDC Foundation and Coca Cola” (Supplementary file 3).

Coca‐Cola staff sent follow‐up messages suggesting that the meeting was

helpful to understand your areas of focus and where we may have mutual interests. There are clearly areas where we can work collaboratively and share insights to advance the work in prevention of obesity and inform of [*sic*] the consumer of choices. We valued getting to know you and your team better and enjoyed the rich discussion relating to your main initiatives. Susan [Roberts, then Coca‐Cola's director of Nutrition in Global Scientific and Regulatory Affairs] will be sharing with you further the work on the low and no calorie beverage research and will follow‐up as more of the data become publically available. We can also forward the papers on the scientific method and interpretation of the epidemiological studies as per our discussion and impact of heterogeneity. It would be helpful to have another meeting in the future to follow‐up on the key discussions on methods and interventions, especially with regards to the fortification programs and grocery channels. (Supplementary file 3)

Subsequent emails from Coca‐Cola regarding this meeting make clear that evidence supportive of Coca‐Cola products was being shared. An example is the focus on low‐calorie sweeteners, with Coca‐Cola's staff claiming in correspondence that “associations between diet beverages and weight in the epidemiological studies is likely the result of reverse causality.” Coca‐Cola's staff also shared a then‐advance copy of a Coca‐Cola–funded publication on heterogeneity in research methods as a reason for the overestimation of SSB‐related diabetes risk in pooled estimates, suggesting that “in many studies adjustment for covariates explained half to all of the association between SSB and T2D.”^28^ This is an instance of Coca‐Cola's using research it funded to influence the CDC staff's perceptions of obesity challenges and likely solutions. Subsequent emails show that these publications were also disseminated among other CDC staff (Supplementary file 3).

#### Theme 3: Helping Coca‐Cola Lobby WHO

Emails exchanged between June 25 and 27, 2015, between Bowman and Malaspina reveal how Coca‐Cola used its contacts inside both the CDC and academia to avert potential business threats, through an exchange previously reported in the print media. On June 25, 2015, Malaspina referred to a news report from an internal Coca‐Cola mailing list regarding Margaret Chan, then the WHO's director‐general, in which she invokes SSB producers as contributors to global obesity and backs restrictions on the consumption of full‐sugar soft drinks. Malaspina forwarded this report to Coca‐Cola staff, academics, and former ILSI officials, stating: “Please see report on WHO. This is getting a lot of publicity. We must find a way of some one [*sic*] such as a famous scientist [to] arrange to pay her a visit. Maybe Jim Hill or someone of similar stature or a US government scientist.” James Hill, a prominent researcher formerly at the University of Colorado Denver, now at the University of Alabama at Birmingham, and a member of the National Academy of Sciences, was among the recipients of this message (Supplementary file 2).

Malaspina described his successful experiences as president of ILSI with lobbying former WHO directors‐general, including alongside the future president of Coca‐Cola, E. Neville Isdell, before concluding: “In summary I am suggesting that collectively we must find a way to start a dialogue with Dr. Chen [*sic*]. If not, she will continue to blast us with significant negative consequences on a global basis. This threat to our business is serious. Warmest Regards, Alex” (Supplementary file 2).

Malaspina then forwarded this message to the CDC's Bowman:

Dear Barbara: How are you? Are you having a nice summer? Any ideas on how to have a conversation with WHO? Now, they do not want to work with industry. Who finds all the new drugs? Not WHO, but industry. She is influenced by the Chinese Govt [*sic*] and is against US. Something must be done. (Supplementary file 2)

Bowman responded the same day:

Am wondering wether [*sic*] anyone with ILSI China, perhaps Madame Chen, might have ideas. Another thought, perhaps someone with connections to the PEPFAR [US President's Emergency Plan for AIDS Relief] program. Or Gates and Bloomberg people, many have close connections with the WHO regional offices. Perhaps an issue of defining legacy. (Supplementary file 2)

After exchanging emails about the nature of PEPFAR and ILSI China, Malaspina wrote:

Dear Barbara, you gave some very good leads. I like the one especially about having Mr. Bill Gates help. Our Chairman knows him well. I will explore this idea with Clyde [Coca‐Cola Senior Vice President Clyde Tuggle]. We would want WHO to start working with ILSI again, with the GEBN and with the food industry in general to resolve issues of food safety and nutrition and for the WHO to not only consider sugary foods as the only cause of obesity but to consider also the life style changes that have been occurring throughout the universe. Since WHO, as you stated has been helped by the pharmaceutical industry to combat HIV/AIDs, why not work closely with the food industry to combat obesity. The Food industry is very willing to come to the table. Let us have dinner soon. (Supplementary file 2)

## Discussion

Records provided by the CDC demonstrate efforts by current and former Coca‐Cola staff to influence the CDC by building relationships, attempting to frame the debate on the role of SSBs in obesity, and using existing contacts to lobby decision makers. These activities are consistent with those observed in previous interactions of SSB companies with policymakers, academia, and the public. Furthermore, these activities may contravene ethics guidelines for CDC staff, which ask staff to consider potential conflicts of interest before engaging with potential partners.^27^ These guidelines state that the CDC should not engage in partnerships in which the “potential partner represents any product that exacerbates morbidity or mortality when used as directed.” In addition, the CDC's ethics guidelines on gifts, including those given via the CDC Foundation, state that the CDC should not accept gifts “if acceptance of the gift could compromise the integrity of a government program or any official involved in that program.”^29^

Yet even though the evidence we obtained does raise important questions, the process of obtaining it was not straightforward. Several of our FOIA requests to the CDC were denied as being too broad, even though the scope of all our FOIA requests was similar, consisting of communications among named CDC employees, CDC Foundation employees with CDC email addresses, and staff or email domains from Coca‐Cola or ILSI (some of these outstanding requests are currently the subject of legal action by U.S. Right to Know). Furthermore, it appears that in at least some instances, the CDC did not supply records, even though their existence was confirmed through FOIA requests to other institutions that were part of the same communication trails. This finding raises questions regarding the CDC's transparency with regard to connections between CDC staff and Coca‐Cola. For example, on March 29, 2012, Applebaum forwarded research to the CDC's Michael Pratt, among others, regarding the health effects of prolonged sitting and the need to “amplify these messages” (Supplementary file 4).

There also appear to have been ongoing research collaborations involving Pratt and Coca‐Cola. On April 4, 2012, Pratt wrote to Applebaum and others to express his concern that Mexico was being dropped from the International Study of Childhood Obesity, Lifestyle and the Environment (ISCOLE), to which Applebaum replied:

Mike—from what was explained to me and during the BtD Symposium—They don't appear to understand the importance of routine and discipline as it relates to the data. It's not a “manana” [*sic*] exercise—it's a “today” requirement. They didn't seem to get it despite outreach from the PI's. (Supplementary file 5)

These examples, obtained from a Louisiana Public Records Act request to Louisiana State University, suggest that there were indeed emails between Pratt and Coca‐Cola that should have been available via FOIA to the CDC but were not provided. The reasons for this are unclear. Despite the importance of transparency in interactions between a leading public health agency and a manufacturer of SSBs, several of our FOIA requests elicited no documents (in some cases, not even a response), even after appeals and timely responses for additional information. Our experience raises questions about how effective the legislation designed to ensure transparency actually is.

One particular email exchange sheds light on the seriousness with which the industry takes the threat of taxing SSBs and the possibility that ongoing relationships between the CDC and SSB companies could alleviate this threat. Alex Malaspina described Margaret Chan's support of a sugar tax as a “global threat to our business.” This statement, while striking, is consistent with Coca‐Cola's communications to shareholders in its annual reports, which make clear that “possible new or increased taxes on sugar‐sweetened beverages to reduce consumption or to raise revenue… could adversely affect our profitability.”^30^ Malaspina then asked for and received advice from a senior CDC contact on how to arrange a meeting with Margaret Chan in order to influence her. This interaction is a troubling example of the core conflict of interest between these two parties, in that the CDC exists to promote public health, while Coca‐Cola exists to maximize profits. In the United States, taxation targeting diet could substantially reduce the burden of cardiovascular disease and diabetes,^31^, ^32^ a conclusion supported by a growing body of evidence that has led the WHO to recommend implementing a “sugar tax” as part of a comprehensive obesity strategy, with a goal of increasing SSB prices by 20%.^33^

As a content analysis of FOIA‐sourced documentation, our research has several important limitations. In order to ensure the validity of our findings,^34^ we have attempted to account for personal biases by reporting all the emails in the online supplementary material so that our interpretations are accessible to all readers and by quoting as much as possible directly from the emails, along with providing information about dates. We have acknowledged potential biases in sampling due to the nature of the data collection methods (FOIA), including the information that the CDC has not yet provided a full response to some of our FOIA requests. We structured our analysis into the main emerging themes in an open process. To ensure reproducibility, we engaged in regular discussions with colleagues working on the corporate determinants of health. Although respondent validation can be an important way of confirming findings, this would not be feasible in our case because of the possible incentives for those involved to conceal their activities.

Our analysis is, by nature, not comprehensive, relying only on FOIA requests that have resulted in returned emails. This is therefore not necessarily indicative of any broader activity between CDC staff and Coca‐Cola, but it is sufficient to reveal the CDC staff's allowing conflicted corporate actors to engage in well‐established tactics to further commercial goals, something that should not occur in an organization established to protect public health. Taken together with the recent resignation of the head of the CDC over purchases of tobacco stock and conflicting financial interests,^35^ such findings should be cause for a re‐evaluation of the CDC's approach, as well as of the nature and purpose of the CDC Foundation, so that it cannot be a vehicle for corporate influence, particularly considering that unhealthy diets are major risk factors for noncommunicable diseases,^36^ with SSB producers identified among the major drivers of these diseases globally.^12^

It is unacceptable for public health organizations to engage in partnerships with companies that have such a clear conflict of interest. The obvious parallel would be to consider the CDC's working with cigarette companies and the dangers that such a partnership would pose. Our analysis has highlighted the need for organizations like the CDC to ensure that they refrain from engaging in partnerships with harmful product manufacturers,^3^ lest they undermine the health of the public they serve.

## Supporting information


**Supplementary file 1**: Responses from the CDC to our FOIA request dated October 21, 2016, for communications between Dr. Janet Collins, Chloe Tonney, and/or Charles Stokes and (1) the Coca‐Cola Co., (2) Rhona Applebaum, (3) Alex Malaspina, and/or (4) the International Life Sciences Institute (January 1, 2011‐present).

**Supplementary file 2**: Responses from the CDC to our FOIA request dated June 29, 2016, for all emails (including attachments) and written correspondence between Dr. Barbara A. Bowman and Alex Malaspina (January 1, 2011‐present).

**Supplementary file 3**: Responses from the CDC to our FOIA request dated January 27, 2017, regarding communications between CDC employees Maureen Culbertson, Kevin Ryan, Leandris Liburd, and Deborah Galuska and (1) the Coca‐Cola Company, (2) Rhona Applebaum, (3) Alex Malaspina, and (4) the International Life Sciences Institute (January 1, 2011‐present).

**Supplementary file 4**: Email response (subject: Dangers of sitting) from a Louisiana Public Records Act request to Louisiana State University dated September 19, 2016, regarding communications to or from (or Cc or Bcc) Professor Katzmarzyk or Professor Church with any staff or employees of the Coca‐Cola Company or the American Beverage Association, including any contract related to the International Study of Childhood Obesity, Lifestyle and the Environment (ISCOLE) study.

**Supplementary file 5**: Email response (subject: RE: ISCOLE news: Confidential) from a Louisiana Public Records Act request to Louisiana State University dated September 19, 2016, regarding communications to or from (or Cc or Bcc) Professor Katzmarzyk or Professor Church with any staff or employees of the Coca‐Cola Company or the American Beverage Association, including any contract related to the International Study of Childhood Obesity, Lifestyle and the Environment (ISCOLE) study.
