# Supplementary material for: Public Meets Private: Conversations Between Coca‐Cola and the CDC
Source: Milbank Q. 2019 Jan 29;97(1):74–90. doi: 10.1111/1468-0009.12368 (PMC6422605; doi:10.1111/1468-0009.12368)
Supplement: Supplementary file 1 — Responses from the CDC to our FOIA request dated October 21, 2016, for communications between Dr. Janet Collins, Chloe Tonney, and/or Charles Stokes and (1) the Coca‐Cola Co., (2) Rhona Applebaum, (3) Alex Malaspina, and/or (4) the International Life Sciences Institute (January 1, 2011‐present). [file MILQ-97-74-s001.pdf]

From: Rhona Applebaum
Sent: 3 Apr 2013 16:44:49 +0000
To: Collins, Janet L. (CDC/ONDIEH/NCCDPHP)
Subject: Congratulations!

Dear Janet—
Heartfelt congratulations on being named Director of The Division of Nutr, Physical Activity and Obesity at CDC. Once settled would welcome the opportunity to come by and discuss current activities and what more can be done.
Have a great day!
Rhona

Rhona S. Applebaum, PhD | VP, Chief Scientific & Regulatory Officer | The Coca-Cola Company
Email: rapplebaum@Coca-Cola.com
Twitter: @RhonaA_CocaCola
Office: 404-676-2177
Fax: 404-598-2177

Coca-Cola Technical I am social

From: Collins, Janet L. (CDC/ONDIEH/NCCDPHP)
Sent: 27 Oct 2014 14:50:44 +0000
To: rapplebaum@coca-cola.com
Subject: Great to see you
Attachments: (b)(6) Resume_ 2014.pdf

Hi Rhona,
It was great to see you Friday. It was a very interesting discussion â€“ I hope we were helpful.
I hope you donâ€™t mind if I share the fact that a CDC colleague of mine (b)(6) is very interested in working at Coca-Cola. I have worked with her for many years and she is dynamic, effective, fast-paced - - I canâ€™t say enough. She would be great for external and governmental relations especially on food, beverage and physical activity policy as well as corporate philanthropy. She has significant international experience as well.
I have attached her CV here. I guess my question is how best for her to proceed? Should she speak to someone there or should she watch position postings?
Thanks!
Janet

Janet Collins, Ph.D.
Director, Division of Nutrition, Physical Activity, and Obesity
National Center for Chronic Disease Prevention and Health Promotion, CDC
770-488-6042 (w); 404-409-0522 (c); jcollins@cdc.gov

(b)(6)

(b)(6)

(b)(6)

(b)(6)

(b)(6)

From: Lankford, Tina J. (CDC/OCOO/OSSAM)
Sent: 22 Sep 2015 13:15:42 -0400
To:
peggyh@uw.edu;cjransaw@gsu.edu;debra.arnold@dm.usda.gov;dawn.m.bading@kp.org;mbaughman@coca-cola.com;candice.bovian@ohr.gatech.edu;chris.calitz@heart.org;jcarter@southernco.com;michaela@hpcareer.net;christa.davis@choa.org;elizabeth.dixon@chick-fil-a.com;cdrogula@dch.ga.gov;brantley.eaton@ohr.gatech.edu;tfariss@kcc.com;tyneshia.farmer@gapac.com;wytaria.henley@choa.org;amber.hosch@turner.com;gimoukhuede@gsu.edu;irvinkooris@homedepot.com;jae.kullar@delta.com;Lindsay, Garry (PSC/FOH/WHPS);teresa.lovely@ky.gov;demaclean@coca-cola.com;melissa.morgan@emory.edu;lucy.polk@opm.gov;ginny.reddick@chick-fil-a.com;eduardo.sanchez@heart.org;Flynn.Jennifer@mayo.edu;Jamal, Ahmed (CDC/ONDIEH/NCCDPHP);bklepper@nbch.org;Tonney, Chloe (CDC cdcfoundation.org);cgillespie@indianachamber.com;Dave.Gardner@dhhs.nc.gov;Galuska, Deborah A. (CDC/ONDIEH/NCCDPHP);Matson Koffman, Dyann M. (CDC/OD/OADS);ewalker@astho.org;Collins, Janet L. (CDC/ONDIEH/NCCDPHP);Jason.Horay@MMICNC.COM;O'Connor, Jean (CDC dph.ga.gov);jh7@uw.edu;VanderVeur, Jennifer L. (CDC/ONDIEH/NCCDPHP) (CTR);jerry.noyce@the-hero.org;jparsons@projectopenhand.org;Manguno, Joseph (CDC/ONDIEH/NCCDPHP) (CTR);Wilson, Katherine (Kathi) (CDC/OPHSS/CSELS);kwan@astho.org;Theis, Kristina (CDC/ONDIEH/NCCDPHP);Greenlund, Kurt (CDC/ONDIEH/NCCDPHP);laura.whitsel@heart.org;Linnan, Laura (CDC email.unc.edu);lcluff@rti.org;mari@advwellness.com;Harper, Mark Neville (CDC/OCOO/OCIO);mwilson@uga.edu;White, Mary C. (CDC/ONDIEH/NCCDPHP);Meg@forprevention.org;Massoudi, Mehran S. (CDC/ONDIEH/NCCDPHP);MBillet@USChamber.com;Schooley, Michael (CDC/ONDIEH/NCCDPHP);Doyle, Nadine (CDC/ONDIEH/NCIPC);nickhobar@learningfront.com;Jones-Jack, Nkenge (CDC/ONDIEH/NCCDPHP) (CTR);Allweiss, Pamela (CDC/ONDIEH/NCCDPHP);pterry@staywell.com;pwrye@coca-cola.com;Mukhtar, Qaiser (CDC/ONDIEH/NCCDPHP);ron.goetzel@truvenhealth.com;syb@ashlininc.com;spolis@tfah.org;shemphill@ashlininc.com;Redd, Tara R. (CDC/ONDIEH/NCCDPHP) (CTR);Bauer, Ursula (CDC/ONDIEH/NCCDPHP);Giles, H. Wayne (CDC/ONDIEH/NCCDPHP);Lang, Jason (CDC/ONDIEH/NCCDPHP)
Cc: Jennifer Childress
Subject: HealthLead at CDC in January

Hi all—I just wanted to call your attention, in case you have missed, that these updates from the HealthLead forum will appear like below from "HealthLead".
Also, check your spam/junk mail folders to make sure you are receiving them.
There are also options to "unsubscribe" if you are not interested in receiving the emails.
We hope to see you all at the Forum!!

Tina Lankford, MPH
CAPT, USPHS
Director, WorkLife Wellness Office
Office of Safety, Security, and Asset Management
CDC/OCOO
p. 404-639-5117
c. 404-996-7366
tlankford@cdc.gov

KNOW
Y UR
NUMBERS CDC Health Day

2015 CDC Flu Shots and Health Days are starting in October.

Full schedule available in mid-September.

Visit your WorkLife Wellness website to help you find a more balanced and healthy lifestyle.

From: HealthLead Forum [mailto:jchildress=ushealthiest.org@mail75.atl51.rsgsv.net] **On Behalf Of** HealthLead Forum
Sent: Tuesday, September 22, 2015 12:02 PM
To: Lankford, Tina J. (CDC/OCOO/OSSAM)
Subject: Take advantage of early bird registration between now and 10/15/15 for the Annual HealthLead Forum Hosted by US Healthiest in Partnership with CDC

Early Bird Registration! - For The January 14-15, 2016
Fourth Annual HealthLead Forum
Atlanta, GA

Contact: Jennifer Childress
jchildress@ushealthiest.org

Email not displaying correctly?
View it in your browser.

US HEALTHIEST

CDC
CENTERS FOR DISEASE CONTROL AND PREVENTION

2016 US Healthiest HealthLead™ Annual Forum

The Bottom Line Future of Worksite Health and Well-being

In Partnership with the Centers for Disease Control and Prevention (CDC)

About the Forum

The two day event will recognize corporate and academic institutions that achieved US Healthiest "HealthLead" accreditation in 2015; include keynotes from respected leaders in the field, case studies featuring HealthLead accredited organizations representing leading practices in comprehensive employee health and well-being; and problem solving sessions jointly facilitated by subject matter experts from the CDC and "boots on the ground" experts within the field on topics including: designing wellness in the culture, selecting metrics of value, building resilience, and engaging leaders.

The HealthLead Forum will be held Thursday and Friday, January 14-15, 2016 at the CDC in Atlanta, Georgia.

Keynotes

- ***Dr. Ray Fabius***, 2014 Global Leadership in Corporate Health Award Recipient, Co-Founder of HealthNEXT -- The link between workforce health and safety and the health of the bottom line
- ***Douglas Stover***, Senior Managing Consultant, Gallup -- The future of worksite health promotion based on findings from the Gallup Engagement Survey and how to drive greater engagement
- ***Dee Edington,*** Founder and Chairman, Edington Associates -- Beyond Zero Trends

Case Studies

- ***Tina Lankford, MPH,*** Director, WorkLife Wellness Office, Office of Safey, Security and Asset Management, CDC
- ***Elizabeth Swanson,*** Vice President, Human Resources, HealthPartners
- ***Megan Amaya, PhD, CHES, AFAA,*** Director of Health Promotion & Wellness, Assistant Professor in Practice, College of Nursing, The Ohio State University

And more!

- Engage with others in the fields of worksite and academic community health as a participant through round table discussions
- Hear thoughts based upon your discussions from the Expert Reactor Panel

- Learn strategies from the Practitioner's Clinics focusing on both the science and the application of current topics in the field

Check out the registration link below for additional information on speakers and topics that will be covered!

Registration

Please click on the following for the Forum. Early bird registration runs through October 15th, 2015.

Lodging

Due to high demand in the area, and extremely limited parking at CDC, we highly recommend that hotel reservations ***be made as soon as possible at the Emory Conference Center.***

The link provided below will take you directly to our block/group rate.

- - $135/night + taxes
  The Emory Conference Center hotel is located directly across from CDC's campus and is within walking distance to CDC; as well as to several restaurants. A free shuttle (for a one mile radius from the hotel) is available for guests. Overnight parking is $15/night.

Sponsorship Opportunities

- Sponsorship opportunities for this event are available. US Healthiest is a non-profit 501 (c) 3. Sponsorship fees are tax deductible. Please refer to the registration link page for additional information. **HHS/CDC is not involved in this solicitation for funds. Sponsorship funds will go directly toward defraying the expenses involved with holding the HealthLead Forum; and not to HHS/CDC.*
- If you are interested in sponsorship opportunities, please contact Jennifer Childress at jchildress@ushealthiest.org

About CDC

CDC works 24/7 to protect America from health, safety and security threats, both foreign and in the U.S. Whether diseases start at home or abroad, are chronic or acute, curable or preventable, human error or deliberate attack, CDC fights disease and supports communities and citizens to do the same.

CDC increases the health security of our nation. As the nation's health protection agency, CDC saves lives and protects people from health threats. To accomplish our mission, CDC conducts critical science and provides health information that protects our nation against expensive and dangerous health threats, and responds when these arise.

About US Healthiest *HealthLead*™ In 2012, US Healthiest created the HealthLead Accreditation Program to recognize public and private sector organizations that demonstrate best practices in employee or academic community health management and well-being. HealthLead is designed to set the standard for workplace and campus health management by expanding the definition and breadth of health to include integrated well-being information and support services, individual/group engagement strategies, and leadership in community health issues.

Sponsors

The Forum is sponsored by the following:

HealthPartners PLUS3.COM

Follow on Twitter Friend on FacebookForward to Friend

Copyright © 2015 US Healthiest, All rights reserved.

You are receiving this email as a friend or partner of US Healthiest.

Our mailing address is:
US Healthiest
2231 Crystal Drive, Arlington, VA
Suite 450
Arlington, VA 22202

Add us to your address book

unsubscribe from this list update subscription preferences

From: Collins, Janet L. (CDC/ONDIEH/NCCDPHP)
Sent: 3 Apr 2013 18:41:04 +0000
To: Rhona Applebaum
Cc: Brown, Andrea (CDC/ONDIEH/NCCDPHP) (CTR)
Subject: RE: Congratulations!

Thanks Rhona. I am delighted to have joined the Division. I have some international travel coming up through mid-April but would be happy to meet after that. My assistant is Andrea Brown (copied here) who can help us arrange a time that works for you.
Best,
Janet

From: Rhona Applebaum [mailto:rapplebaum@coca-cola.com]
Sent: Wednesday, April 03, 2013 12:45 PM
To: Collins, Janet L. (CDC/ONDIEH/NCCDPHP)
Subject: Congratulations!

Dear Janetâ€"
Heartfelt congratulations on being named Director of The Division of Nutr, Physical Activity and Obesity at CDC. Once settled would welcome the opportunity to come by and discuss current activities and what more can be done.
Have a great day!
Rhona

Rhona S. Applebaum, PhD | VP, Chief Scientific & Regulatory Officer | The Coca-Cola Company
Email: rapplebaum@Coca-Cola.com
Twitter: @RhonaA_CocaCola
Office: 404-676-2177
Fax: 404-598-2177

Coca-Cola Technical I am social

From: Rhona Applebaum
Sent: 27 Oct 2014 15:05:01 +0000
To: Collins, Janet L. (CDC/ONDIEH/NCCDPHP)
Subject: RE: Great to see you

Great to see you as well Janet. Prof Veller very much enjoyed the discussion and potential for collaborations
Many thanks for the CV. I will share internally –must she stay in Atlanta or is she willing to move to DC?
Rhona

From: Collins, Janet L. (CDC/ONDIEH/NCCDPHP) [mailto:jlc1@cdc.gov]
Sent: Monday, October 27, 2014 10:51 AM
To: Rhona Applebaum
Subject: Great to see you

Hi Rhona,
It was great to see you Friday. It was a very interesting discussion – I hope we were helpful.
I hope you don't mind if I share the fact that a CDC colleague of mine (Maureen Culbertson) is very interested in working at Coca-Cola. I have worked with her for many years and she is dynamic, effective, fast-paced - - I can't say enough. She would be great for external and governmental relations especially on food, beverage and physical activity policy as well as corporate philanthropy. She has significant international experience as well.
I have attached her CV here. I guess my question is how best for her to proceed? Should she speak to someone there or should she watch position postings?
Thanks!
Janet

Janet Collins, Ph.D.
Director, Division of Nutrition, Physical Activity, and Obesity
National Center for Chronic Disease Prevention and Health Promotion, CDC
770-488-6042 (w); 404-409-0522 (c); jcollins@cdc.gov

From: Collins, Janet L. (CDC/ONDIEH/NCCDPHP)
Sent: 27 Oct 2014 21:20:38 +0000
To: Rhona Applebaum
Subject: RE: Great to see you

Most likely Atlanta; but she traveled to DC nearly every week for her last assignment. Perhaps she would considerâ€¦.
I can check with her if you want me to.
Thanks!
Janet
Janet Collins, Ph.D.
Director, Division of Nutrition, Physical Activity, and Obesity
National Center for Chronic Disease Prevention and Health Promotion, CDC
770-488-6042 (w); 404-409-0522 (c); jcollins@cdc.gov

From: Rhona Applebaum [mailto:rapplebaum@coca-cola.com]
Sent: Monday, October 27, 2014 11:05 AM
To: Collins, Janet L. (CDC/ONDIEH/NCCDPHP)
Subject: RE: Great to see you
Great to see you as well Janet. Prof Veller very much enjoyed the discussion and potential for collaborations
Many thanks for the CV. I will share internally â€“must she stay in Atlanta or is she willing to move to DC?
Rhona
From: Collins, Janet L. (CDC/ONDIEH/NCCDPHP) [mailto:jlc1@cdc.gov]
Sent: Monday, October 27, 2014 10:51 AM
To: Rhona Applebaum
Subject: Great to see you
Hi Rhona,
It was great to see you Friday. It was a very interesting discussion â€“ I hope we were helpful.
I hope you donâ€™t mind if I share the fact that a CDC colleague of mine (Maureen Culbertson) is very interested in working at Coca-Cola. I have worked with her for many years and she is dynamic, effective, fast-paced - - I canâ€™t say enough. She would be great for external and governmental relations especially on food, beverage and physical activity policy as well as corporate philanthropy. She has significant international experience as well.
I have attached her CV here. I guess my question is how best for her to proceed? Should she speak to someone there or should she watch position postings?
Thanks!
Janet
Janet Collins, Ph.D.
Director, Division of Nutrition, Physical Activity, and Obesity
National Center for Chronic Disease Prevention and Health Promotion, CDC
770-488-6042 (w); 404-409-0522 (c); jcollins@cdc.gov

From: Collins, Janet L. (CDC/ONDIEH/NCCDPHP)
Sent: 25 Jun 2014 12:47:51 +0000
To: Susan A. Roberts;Beate Lloyd;Ryan, Kevin A. (CDC/ONDIEH/NCCDPHP);Liburd, Leandris C. (CDC/OD/OMHHE)
Cc: Karima Kendall;Galuska, Deborah A. (CDC/ONDIEH/NCCDPHP)
Subject: RE: Thank you.

Thank you so much for these resources. I am copying our Associate Director of Science, Deb Galuska, to help us make optimal use of these resources.
Thanks!
Janet

Janet Collins, Ph.D.
Director, Division of Nutrition, Physical Activity, and Obesity
National Center for Chronic Disease Prevention and Health Promotion, CDC
770-488-6042 (w); 404-409-0522 (c); jcollins@cdc.gov

From: Susan A. Roberts [mailto:suroberts@coca-cola.com]
Sent: Tuesday, June 24, 2014 10:27 PM
To: Beate Lloyd; Collins, Janet L. (CDC/ONDIEH/NCCDPHP); Ryan, Kevin A. (CDC/ONDIEH/NCCDPHP); Liburd, Leandris C. (CDC/OD/OMHHE)
Cc: Karima Kendall
Subject: RE: Thank you.

Dear Janet, Kevin and Leandris,
I was very glad to be able to participate in the meeting by phone on Friday and also look forward to on-going discussions with you to identify areas of potential collaboration and information sharing.
In follow up to Beateâ€™s note below, please find attached two recent publications on LNCS as well as a draft manuscript on heterogeneity in meta-analyses with suggested approaches to help the non-expert better interpret the outcome statistic.

1) Low-calorie sweeteners vs water during weight loss study (Jim Hill from Univ of Colorado and Gary Foster from Temple Univ, both former presidents of The Obesity Society, were the co-PIs). As mentioned on the call, this is phase I of a two-part study. Phase 2 , the maintenance phase (additional 9 months) will be completed by end of year. Leandris, this is the paper I sent to you on Friday. I have also attached the editorial associated with the paper for an additional perspective. We plan to address many of the outstanding questions of mechanism/appetite in future studies.
2) Meta-analysis of low-calorie sweeteners and weight â€" the analysis is done on both RCTs and prospective population studies. The findings show a modest but significant benefit of low-calorie sweeteners in weight loss and the effect is largely driven by the beverage studies. It also provides additional support for the view that the positive associations between diet beverages and weight in the epidemiological studies is likely the result of reverse causality.
3) The heterogeneity methods paper. This paper has been accepted in the journal Systematic Reviews. Therefore please treat as confidential until it is published.

I would be glad to discuss any of this further.
Best wishes, Susan

From: Beate Lloyd
Sent: Tuesday, June 24, 2014 6:39 PM
To: jcollins@cdc.gov; kir0@cdc.gov; Liburd, Leandris C. (CDC/OD/OMHHE)
Cc: Susan A. Roberts; Karima Kendall
Subject: Thank you.

Dear Janet,

I would like to thank you, Kevin and Leandris very much for taking the time to meet with us. It was very helpful to understand your areas of focus and where we may have mutual interests. There are clearly areas where we can work collaboratively and share insights to advance the work in prevention of obesity and inform of the consumer of choices.

We valued getting to know you and your team better and enjoyed the rich discussion relating to your main initiatives. Susan will be sharing with you further the work on the low and no calorie beverage research and will follow-up as more of the data become publically available. We can also forward the papers on the scientific method and interpretation of the epidemiological studies as per our discussion and impact of heterogeneity.

It would be helpful to have another meeting in the future to follow-up on the key discussions on methods and interventions, especially with regards to the fortification programs and grocery channels.

Thank you again very much for your interest and time.

Warmest regards,

Beate

Beate B. Lloyd PhD RD LD
Senior Director Nutrition COE
Global Scientific and Regulatory Affairs
The Coca-Cola Company
P: (404) 676-2149
C: (404) 545-3141

From: Collins, Janet L. (CDC/ONDIEH/NCCDPHP)
Sent: 25 Jun 2014 12:46:07 +0000
To: Beate Lloyd
Cc: Susan A. Roberts;Karima Kendall;Ryan, Kevin A. (CDC/ONDIEH/NCCDPHP);Liburd, Leandris C. (CDC/OD/OMHHE)
Subject: RE: Thank you.

Beate,
Thanks again for meeting with us. We enjoyed it and learned a lot. I would be happy to set up a meeting with my fortification group either here or at the Coca-Cola offices. We also look forward to thinking about other areas of mutual interest and continuing our discussions.
Thanks,
Janet
Janet Collins, Ph.D.
Director, Division of Nutrition, Physical Activity, and Obesity
National Center for Chronic Disease Prevention and Health Promotion, CDC
770-488-6042 (w); 404-409-0522 (c); jcollins@cdc.gov

From: Beate Lloyd [mailto:belloyd@coca-cola.com]
Sent: Tuesday, June 24, 2014 6:39 PM
To: Collins, Janet L. (CDC/ONDIEH/NCCDPHP); Ryan, Kevin A. (CDC/ONDIEH/NCCDPHP); Liburd, Leandris C. (CDC/OD/OMHHE)
Cc: Susan A. Roberts; Karima Kendall
Subject: Thank you.
Dear Janet,
I would like to thank you, Kevin and Leandris very much for taking the time to meet with us. It was very helpful to understand your areas of focus and where we may have mutual interests. There are clearly areas where we can work collaboratively and share insights to advance the work in prevention of obesity and inform of the consumer of choices.
We valued getting to know you and your team better and enjoyed the rich discussion relating to your main initiatives. Susan will be sharing with you further the work on the low and no calorie beverage research and will follow-up as more of the data become publically available. We can also forward the papers on the scientific method and interpretation of the epidemiological studies as per our discussion and impact of heterogeneity.
It would be helpful to have another meeting in the future to follow-up on the key discussions on methods and interventions, especially with regards to the fortification programs and grocery channels.
Thank you again very much for your interest and time.
Warmest regards,
Beate
Beate B. Lloyd PhD RD LD
Senior Director Nutrition COE
Global Scientific and Regulatory Affairs
The Coca-Cola Company
P: (404) 676-2149

C: (404) 545-3141

From: Susan A. Roberts
Sent: 25 Jun 2014 02:27:25 +0000
To: Beate Lloyd;Collins, Janet L. (CDC/ONDIEH/NCCDPHP);Ryan, Kevin A. (CDC/ONDIEH/NCCDPHP);Liburd, Leandris C. (CDC/OD/OMHHE)
Cc: Karima Kendall
Subject: RE: Thank you.
Attachments: Miller 2014 AJCN Meta-analysis o fLow-Calorie Sweeteners Body Weight.pdf, Anton 2014 Obesity editorial.pdf, Peters et al 2014 Obesity LNCS vs Water on weight loss.pdf, Heterogeneity Final March 28 2014.docx

Dear Janet, Kevin and Leandris,
I was very glad to be able to participate in the meeting by phone on Friday and also look forward to on-going discussions with you to identify areas of potential collaboration and information sharing.
In follow up to Beate's note below, please find attached two recent publications on LNCS as well as a draft manuscript on heterogeneity in meta-analyses with suggested approaches to help the non-expert better interpret the outcome statistic.

1) Low-calorie sweeteners vs water during weight loss study (Jim Hill from Univ of Colorado and Gary Foster from Temple Univ, both former presidents of The Obesity Society, were the co-PIs). As mentioned on the call, this is phase I of a two-part study. Phase 2 , the maintenance phase (additional 9 months) will be completed by end of year. Leandris, this is the paper I sent to you on Friday. I have also attached the editorial associated with the paper for an additional perspective. We plan to address many of the outstanding questions of mechanism/appetite in future studies.
2) Meta-analysis of low-calorie sweeteners and weight – the analysis is done on both RCTs and prospective population studies. The findings show a modest but significant benefit of low-calorie sweeteners in weight loss and the effect is largely driven by the beverage studies. It also provides additional support for the view that the positive associations between diet beverages and weight in the epidemiological studies is likely the result of reverse causality.
3) The heterogeneity methods paper. This paper has been accepted in the journal Systematic Reviews. Therefore please treat as confidential until it is published.

I would be glad to discuss any of this further.
Best wishes, Susan

From: Beate Lloyd
Sent: Tuesday, June 24, 2014 6:39 PM
To: jcollins@cdc.gov; kir0@cdc.gov; Liburd, Leandris C. (CDC/OD/OMHHE)
Cc: Susan A. Roberts; Karima Kendall
Subject: Thank you.

Dear Janet,
I would like to thank you, Kevin and Leandris very much for taking the time to meet with us. It was very helpful to understand your areas of focus and where we may have mutual interests. There are clearly areas where we can work collaboratively and share insights to advance the work in prevention of obesity and inform of the consumer of choices.
We valued getting to know you and your team better and enjoyed the rich discussion relating to your main initiatives. Susan will be sharing with you further the work on the low and no calorie beverage research and will follow-up as more of the data become publically available. We can also forward the papers on the scientific method and interpretation of the epidemiological studies as per our discussion and impact of heterogeneity.

It would be helpful to have another meeting in the future to follow-up on the key discussions on methods and interventions, especially with regards to the fortification programs and grocery channels.
Thank you again very much for your interest and time.

Warmest regards,

Beate

Beate B. Lloyd PhD RD LD
Senior Director Nutrition COE
Global Scientific and Regulatory Affairs
The Coca-Cola Company
P: (404) 676-2149
C: (404) 545-3141

AJCN. First published ahead of print June 18, 2014 as doi: 10.3945/ajcn.113.082826.

Low-calorie sweeteners and body weight and composition: a meta-analysis of randomized controlled trials and prospective cohort studies[1–3]

Paige E Miller and Vanessa Perez

Abstract

Background: Replacement of caloric sweeteners with lower- or no-calorie alternatives may facilitate weight loss or weight maintenance by helping to reduce energy intake; however, past research examining low-calorie sweeteners (LCSs) and body weight has produced mixed results.

Objective: The objective was to systematically review and quantitatively evaluate randomized controlled trials (RCTs) and prospective cohort studies, separately, that examined the relation between LCSs and body weight and composition.

Design: A systematic literature search identified 15 RCTs and 9 prospective cohort studies that examined LCSs from foods or beverages or LCSs consumed as tabletop sweeteners. Meta-analyses generated weighted mean differences in body weight and composition values between the LCS and control groups among RCTs and weighted mean correlations for LCS intake and these parameters among prospective cohort studies.

Results: In RCTs, LCSs modestly but significantly reduced all outcomes examined, including body weight (−0.80 kg; 95% CI: −1.17, −0.43), body mass index [BMI (in kg/m^2): −0.24; 95% CI: −0.41, −0.07], fat mass (−1.10 kg; 95% CI: −1.77, −0.44), and waist circumference (0.83 cm; 95% CI: 1.29, 0.37). Among prospective cohort studies, LCS intake was not associated with body weight or fat mass, but was significantly associated with slightly higher BMI (0.03; 95% CI: 0.01, 0.06).

Conclusions: The current meta-analysis provides a rigorous evaluation of the scientific evidence on LCSs and body weight and composition. Findings from observational studies showed no association between LCS intake and body weight or fat mass and a small positive association with BMI; however, data from RCTs, which provide the highest quality of evidence for examining the potentially causal effects of LCS intake, indicate that substituting LCS options for their regular-calorie versions results in a modest weight loss, and may be a useful dietary tool to improve compliance with weight loss or weight maintenance plans. *Am J Clin Nutr* doi: 10.3945/ajcn.113.082826.

INTRODUCTION

Over the past several decades, the worldwide prevalence of overweight and obesity has increased markedly (1, 2). Because overweight and obesity are major causes of comorbidities, including cardiovascular disease, hypertension, type 2 diabetes, certain cancers, and other health conditions (3), identifying strategies that help regulate body weight is imperative. Replacement of caloric sweeteners (herein referred to as sugar) with lower-calorie alternatives is one such strategy that may help reduce energy intake, thereby facilitating weight loss, weight maintenance, or prevention of weight gain (4). Low-calorie sweeteners (LCSs)[4] may improve adherence to weight loss or maintenance plans by preserving the palatability of foods and beverages with fewer calories than sugar (5). Conversely, a hypothesis that LCS intake promotes, rather than prevents, weight gain by altering taste and metabolic signaling, decreasing satiety, and increasing appetite, hunger, sweets cravings, and ultimately food intake emerged nearly 3 decades ago (6, 7). However, a recent review of randomized controlled trials (RCTs) (8), and new findings from an RCT that examined the effect of low-calorie sweetened beverages (LCSBs) on overall dietary patterns (9), failed to support this hypothesis.

LCSs are classified into 2 categories: *1*) nonnutritive sweeteners, which are also referred to as high-intensity, high-potency, and intense sweeteners and *2*) bulk sweeteners or sugar alcohols (eg, polyols). Nonnutritive sweeteners have an intense sweet taste, contribute negligible to zero calories as consumed, and are used in minimal quantities to replace a larger amount of sugar. Polyols replace the bulk of sugar but are generally less sweet (with the exception of xylitol and maltitol); therefore, they are often used in combination with nonnutritive sweeteners. As delivered, polyols contribute 0 to 3.0 kcal/g, compared with 4 kcal/g from sugar (10, 11). LCSs allowed in the United States by the Food and Drug Administration include acesulfame potassium, aspartame, luo han guo extract, neotame, saccharin, steviol glycosides, and sucralose; other nonnutritive sweeteners, such as cyclamate, thaumatin, neohesperidin dihydrochalcone,

[1] From the Center for Epidemiology, Biostatistics, and Computational Biology, Exponent Inc, Chicago, IL.

[2] Supported by the North American Branch of the International Life Sciences Institute. This is a free access article, distributed under terms (http://www.nutrition.org/publications/guidelinesand-policies/license/) that permit unrestricted noncommercial use, distribution, and reproduction in any medium, provided the original work is properly cited.

[3] Address reprint requests and correspondence to V Perez, Exponent Inc, 525 West Monroe Street, Suite 1050, Chicago, IL 60661. E-mail: vperez@exponent.com.

[4] Abbreviations used: LCS, low-calorie sweetener; LCSB, low-calorie sweetened beverage; RCT, randomized controlled trial; SSB, sugar-sweetened beverage; WGMC, weighted group mean correlation; WGMD, weighted group mean difference.

Received December 29, 2013. Accepted for publication May 19, 2014.
doi: 10.3945/ajcn.113.082826.

Copyright (C) 2014 by the American Society for Nutrition

and alitame, are authorized for use in other countries (4, 12, 13). Among the polyols, the Food and Drug Administration has approved the use of erythritol, hydrogenated starch hydrolysates, isomalt, lactitol, maltitol, mannitol, sorbitol, and xylitol (11, 13); polyglycitol syrup is authorized for use by the European Commission (14).

Overall, research into the potential health effects of LCSs is complicated by the diversity of available LCSs and the growing number of foods and beverages sweetened with one or more LCSs (8, 15). Contributing to this complexity, the composition of LCSs in foods and beverages and consumer preference for particular LCSs continue to change over time. Although past reviews on LCSs and weight control have been published (16–19), none to date have provided a quantitative evaluation of the evidence from both RCTs and prospective cohort studies, examined all types of LCSs, investigated body-composition outcomes, or included several RCTs published in recent years (20–24). Therefore, the purpose of the current study was to systematically review and quantitatively evaluate results from RCTs and prospective cohort studies, separately, that examined the relation between LCSs and body weight, fat mass, BMI, and waist circumference.

MATERIALS AND METHODS

Literature search and study selection

This systematic review and meta-analysis follows the Preferred Reporting Items for Systematic Reviews and Meta-Analyses statement (25). No prespecified protocol was followed for this study. A comprehensive literature search using PubMed (http://www.ncbi.nlm.nih.gov/pubmed) was performed to identify RCTs and prospective cohort studies through 16 September 2013 with no lower date limit. The complete search string can be found elsewhere (*see* Supplemental Figure 1 under "Supplemental data" in the online issue). In brief, a combination of MeSH and relevant free text terms designed to capture the following were used: all individual LCSs (generic and name brands) approved for use globally; food and beverage sources of LCS such as "diet soda"; different names for LCS such as "intense sweetener" and "polyol"; body weight and composition parameters (eg, "waist circumference" and "fat mass"); and relevant study designs, including "cohort" and "controlled trial." The MeSH terms included "sweetening agent," "body mass index," "adipose tissue," "adiposity," "body weight," "cohort studies," and "randomized controlled trial." Supplementary literature searches involved examining the reference lists of all relevant studies and pertinent review articles to identify articles not captured in the initial search. Established guidelines for systematic reviews provided by the Agency for Health Care Research and Quality (26) were followed, and special considerations for reviews in the field of nutrition (27) were addressed.

Prospective cohorts and RCTs were eligible if the following criteria were met: *1*) study population was generally healthy (ie, not hospitalized or acutely ill); *2*) dose or intake data for at least one LCS (nonnutritive sweetener or polyol) or delivery vehicle of LCS were provided; *3*) the effect of LCS, compared with the control arm, could be examined independently of other intervention components; and *4*) outcome data for at least one measure of body weight or composition were available. Child and adult populations were eligible. A minimum study duration of ≥2 wk for RCTs (28) and ≥6 mo for prospective cohorts (29) was selected to be consistent with past published meta-analyses that were similar in design (28, 29) and to be inclusive in an area of research with a relatively small pool of studies.

Data extraction

The following information was extracted from each RCT or prospective cohort: first author, publication year, geographic location, demographic and health characteristics, sample size, source and type of LCS, and outcomes measured. For RCTs, additional information on the intervention and control regimens, dose of specific LCS (or LCS source), and means and SDs of changes in the outcomes from baseline to trial end for all study arms were obtained. To avoid double-counting results from 3 studies that had more than one control arm (23, 24, 30), we extracted results a priori from the most relevant comparison group, ie, the one that was most comparable with the other included studies. This included sugar-sweetened beverages (SSBs) in 2 studies (23, 30) and a usual diet that included 280-kcal caloric beverages/d other than milk in the third study (24). When change SDs were unavailable, methods described in the Cochrane Handbook for Systematic Reviews of Interventions (31) were relied on to calculate or estimate SDs from other statistics in the published articles (eg, SDs were calculated from SEs or CIs). For studies with missing measures of variance for mean change, SDs were estimated by using the correlation coefficient (r) method, in which the average correlation coefficient ($r = 0.965$) between baseline and trial-end values from all other included studies was used. For one study with missing baseline, trial-end, and change SDs (32), values for change SD were imputed from the average change SD among the other RCTs included in the meta-analysis. Sensitivity analyses evaluated the change in overall study results by removing *1*) the RCT by Kanders et al (32) that had imputed SDs for change in the outcome measurements and, in a separate analysis, *2*) the RCT by Knopp et al (33) that had an inherently different intervention design (aspartame capsules were provided rather than LCSs in foods or beverages or as tabletop sweeteners).

Additional data extracted from prospective cohort studies included cohort name, dietary-assessment method, year in which diet was assessed, exposure unit, intakes within each exposure category, statistical adjustments, and results data as presented by the author, which were as follows: the β estimate, SE, and associated t statistic for change in the outcome per unit increase in the baseline exposure (estimated from linear regression models) or the change in outcome and SE in each category of intake. Two studies reported outcome data as RRs and 95% CIs: one for risk of overweight/obesity (34) and the other for risk of elevated waist circumference (35); these data were extracted and reviewed but were not included in the meta-analysis because ≥2 studies were required to pool results. Authors were contacted up to 3 times, if needed, for missing data and study details.

Statistical analysis

A meta-analysis was performed by using random-effects modeling with Comprehensive Meta-Analysis Software (version 2.2.046; Biostat). The primary meta-analyses for the RCTs evaluated the mean change in body weight, fat mass, BMI, or waist circumference (mean value at follow-up minus the mean

value at baseline for both groups) between the LCS intervention group and the comparator arm. Two or more RCTs by outcome were required to generate weighted group mean differences (WGMDs), 95% CIs, and corresponding P values for heterogeneity. The degree of inconsistency between studies was evaluated by using the I^2 statistic ($0\% \leq I^2 \leq 100\%$, where increasing values correspond to greater heterogeneity) (36).The same outcomes measured on different scales were converted to the same unit (eg, pounds to kilograms) for comparability between studies (31). Meta-analyses by the following subgroups were performed: *1*) age group [children compared with adults (>18 y)], *2*) sex, *3*) source of LCS (beverage, foods, or tabletop sweetener), and *4*) whether the LCS intervention resulted in significantly lower energy intake compared with the comparator arm.

The primary meta-analyses of the prospective cohort studies evaluated the reported or calculated t statistics (regression slope divided by its SE) and specified the effect direction as determined by a positive or negative regression coefficient. This analysis allowed for the synthesis of the regression slopes based on a standardized metric (37).Two or more studies by outcome were required to generate weighted group mean correlations (WGMCs), 95% CIs, corresponding P values for heterogeneity, and the I^2 statistic. Whenever possible, subgroup analyses were conducted as done for the RCTs and according to whether the studies provided *1*) total energy-adjusted results and *2*) baseline BMI-adjusted analyses (adjustments for other baseline body-composition variables were eligible but not performed).

Pooled summary estimates from the random-effects models were compared with the results from fixed-effects models to examine the potential for small-study bias. Potential publication bias was examined by visual inspection of funnel plots and with Egger's regression test (38). The x axis in the funnel plots represents the effect size of each RCT or the Fisher-transformed correlation value of each prospective cohort study. The y axis represents the SE of the effect size or correlation value of the corresponding study. The solid vertical line is the pooled summary estimate from the meta-analysis. In the absence of publication bias, the plot resembles a symmetrical inverted funnel, and Egger's regression test will fail to reject the null hypothesis of no funnel plot asymmetry.

RESULTS

Literature search and study characteristics

The PubMed search yielded 522 references, of which 93 articles were retained for full-text screening. Fifteen RCTs (20–24, 30, 32, 33, 39–45) and 9 prospective cohorts were ultimately eligible for inclusion in the meta-analysis (**Figure 1**). Primary characteristics of the 15 included RCTs are provided in **Table 1**. A total of 1951 participants were included in the meta-analysis; individual trial size ranged from 19 adults (42) in a crossover study to 632 children in a parallel-design trial (20). Most studies were conducted in adult populations; 4 studies were conducted in children (20, 21, 33, 39). Study duration varied widely [3 wk (30) to 78 wk (20)], as did the mean age of the participants [4 y (20) to 65 y (24)]. The mean BMI (in kg/m^2) across the studies varied from 22.5 (44) to 37.7 (32) (median: 29.1), with the exception of one study among young children aged 4–11 y (20), in which the mean BMI was 16.8 (0.03 z score for age) (20). Eight of the trials were conducted solely in overweight or obese populations (21–24, 32, 40, 43, 45). Nine studies presented

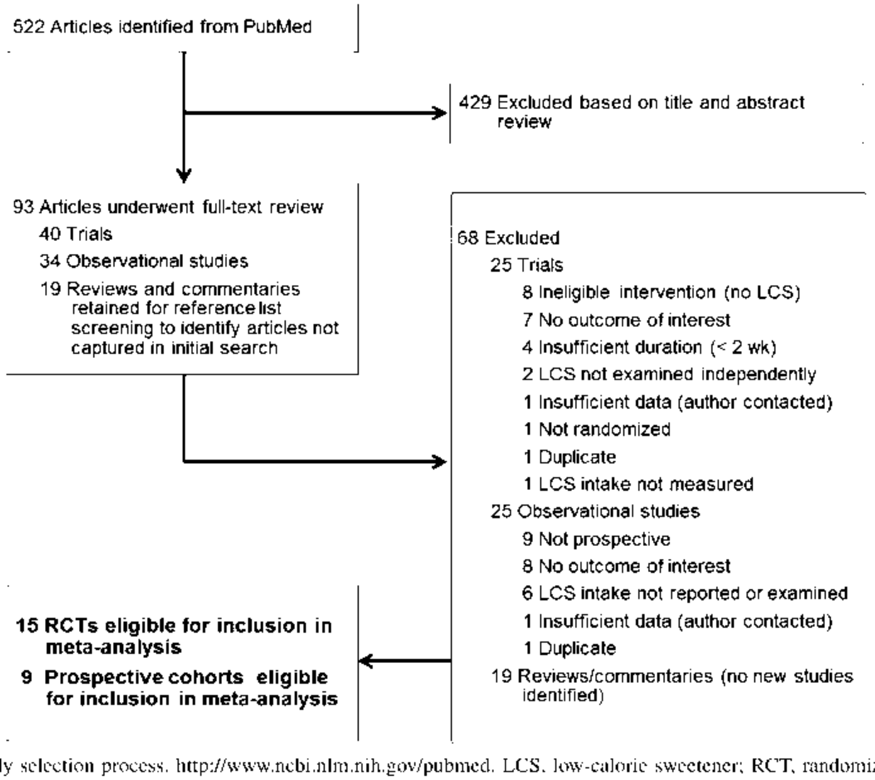

FIGURE 1. Study selection process. http://www.ncbi.nlm.nih.gov/pubmed. LCS, low-calorie sweetener; RCT, randomized controlled trial.

TABLE 1
Characteristics of the randomized controlled trials included in the meta-analysis[1]

Study (ref)	Age[2]	Sex (M/F)	Mean BMI	Intervention details: Control group	Intervention details: LCS group	LCS dose/d[3]	Duration[4]	Outcome
	y		*kg/m*[2]				*wk*	
Blackburn et al, 1997 (40)	20–60	0/136	37.3	Energy-reduced diet with sucrose-sweetened foods and beverages plus table sugar for sweetener	Energy-reduced diet with aspartame-sweetened foods and beverages plus aspartame sweetener	285 ± 235 mg aspartame	16	BW
de Ruyter et al, 2012 (20)	4–11	343/289	16.8 (0.03 *z* score)	8 oz/d SSB	8 oz/d LCSB	34 mg sucralose + 12 mg ACK	78	BMI *z* score, BW, fat mass,[5] WC
Ebbeling et al, 2006 (39)	13–18	47/56	25.3	Usual diet, which included ≥ 1 SSB/d	Up to 4 cans or bottles of LCSBs and water per day	21.7 oz LCSB	25	BMI
Ebbeling et al, 2012 (21)	14–16	124/100	30.3	Usual diet, which included ≥ 1 SSB or fruit juice/d	LCSBs and water	10.8 oz LCSB	52	BMI, BW, fat mass[5]
Gatenby et al, 1997 (41)	18–50	0/65	23.1	Usual diet that did not include habitual consumption of reduced-sugar foods	Detailed instructions provided for substituting conventional sugar-containing foods with those containing LCS	NR	10	BW
Gostner et al, 2005 (42)	21–53	7/12	NR	Sucrose-sweetened foods as part of an otherwise typical Western-style diet (low fiber, high fat)	Isomalt-sweetened foods as part of an otherwise typical Western-style diet (low fiber, high fat)	30 g isomalt	4	BW
Kanders et al, 1988 (32)	26–60	11/45	37.7	Energy-reduced diet without any aspartame- or saccharin-sweetened products	Energy-reduced diet with aspartame-sweetened foods and beverages plus aspartame sweetener	383 mg aspartame	12	BW, BMI, fat mass[5]
Knopp et al, 1976 (33)	10–21	4/51	NR[6]	Energy-reduced diet plus lactose capsules	Energy-reduced diet plus aspartame capsules	2700 mg aspartame	13	BW
Maersk et al, 2012 (23)	20–50	17/30	32.1	SSB	Aspartame-sweetened soda	33.8 oz LCSB	26	BW, fat mass[7]
Njike et al, 2011 (22)[8]	52 − 11	6/33	30.3	Sugar-sweetened hot cocoa	ACK- and aspartame-sweetened hot cocoa	16 oz LCSB	6	BW, BMI, WC

(Continued)

TABLE 1 (*Continued*)

Study (ref)	Age[2]	Sex (M/F)	Mean BMI	Intervention details: Control group	Intervention details: LCS group	LCS dose/d[3]	Duration[4]	Outcome
Raben et al, 2002 (43)	20–50	6/35	27.8	Sugar-sweetened food and beverages	Food and beverages with LCS (by weight, 54% aspartame, 22% ACK, 23% cyclamate, and 1% saccharin)	480–670 mg aspartame + ACK + cyclamate + saccharin	10	BW, fat mass[5]
Reid et al, 2007 (44)	20–55	0/133	22.5	SSB	Aspartame- and ACK-sweetened soda	34 oz LCSB	4	BMI
Reid et al, 2010 (45)	20–55	0/53	27.5	SSB	Aspartame- and ACK-sweetened soda	34 oz LCSB	4	BW
Tate et al, 2012 (24)	18–65	50/268	36.2	Usual diet, which included 280-kcal caloric beverages/d other than milk	Replacement of caloric beverages with LCSB (population consumed ≥ 280-kcal caloric beverages/d other than milk before intervention)	24–32 oz LCSB	26	BW, WC
Tordoff and Alleva, 1990 (30)[8]	22.9 ± 3.7	21/9	25.2	SSBs	Aspartame-sweetened soda	38 oz LCSB	3	BW

[1] 1 oz = ~30 mL. ACK, acesulfame potassium; BW, body weight; LCS, low-calorie sweetener; LCSB, low-calorie sweetened beverage; NR, not reported; ref, reference; SSB, sugar-sweetened beverage; WC, waist circumference.

[2] Mean ± SD is shown when age range was not reported by the authors.

[3] The dose of the LCS source (beverages) is shown when the actual dose of LCS was not reported in the study.

[4] Reflects the length of time in each study arm, not the duration of the entire study in the case of crossover trials.

[5] Measured by bioelectrical impedance analysis.

[6] Study population was overweight, on average, but mean BMI was not provided.

[7] Measured by dual-energy X-ray absorptiometry.

[8] Crossover design.

results for males and females combined, 2 studies reported results separately (30, 32), and 4 studies were conducted only in women (40, 41, 44, 45).

Most of the LCS intervention regimens exclusively evaluated LCSBs (20–24, 30, 39, 44, 45); information on the LCS composition in these beverages was available in 5 of the studies [aspartame alone (23, 30) or aspartame plus acesulfame potassium (22, 44, 45)]. Of the remaining 6 trials, 2 assigned participants to diets with aspartame-sweetened foods and beverages plus aspartame for tabletop sweetener (32, 40), 1 provided aspartame capsules (33), 1 had participants substitute conventional sugar-containing foods with those containing LCS (41), 1 provided isomalt-sweetened foods (42), and 1 provided participants with foods and beverages sweetened with aspartame, cyclamate, acesulfame potassium, and saccharin (43). The group that received SSBs or sugar-sweetened foods or the group assigned to follow a habitual (usual) diet that contained SSBs and sugar-sweetened foods was evaluated as the control arm for most studies (20–24, 30, 39–45). The other control arms consisted of groups that received lactose capsules (33) or an energy-reduced diet (similar to the intervention) that did not include LCSs (32).

The main characteristics of the prospective cohort studies are shown in **Table 2**. The number of subjects in each study ranged from 465 (46) to 51,603 (47), with a total of 103,940 subjects across the 9 cohorts. Four studies were conducted in children and adolescents (48–51) and 5 in adults (34, 35, 46, 47, 52). Five studies provided results for men and women combined (34, 35, 46, 49, 51), 2 provided results for each sex separately (48, 50), and 2 examined women only (47, 52). Most of the cohort studies reported only one outcome (35, 46–48, 51, 52), and the outcome reported by each of these 6 studies varied: BMI (48), body weight (46, 47, 52), fat mass (51), and risk of elevated waist circumference (35). The other studies examined BMI and fat mass (50), BMI and body weight (49), and BMI and incidence of becoming overweight/obese or obese (34). Only 2 types or sources of LCS were examined across the cohorts—beverages sweetened with LCS (34, 35, 47–51) or saccharin (46, 52).

Meta-analysis results from RCTs

Body weight

Shown in **Figure 2** are the effect sizes, 95% CIs, and precisions of each study from the meta-analysis of RCTs examining LCSs and body weight among all subjects (forest plot A) and by age group (forest plot B), sex (forest plot C), and source of LCS (forest plot D). In the meta-analysis of all subjects, LCS reduced body weight by 0.80 kg (95% CI: 1.17, 0.43; fixed-effect WGMD = −0.61) compared with the comparator arm. Removal of data from Kanders et al (32) and Knopp et al (33), in separate analyses, marginally affected the results: 0.79 kg (95% CI: −1.17, −0.42; fixed-effect WGMD = −0.60) and −0.79 kg (95% CI: −1.18, −0.41; fixed-effect WGMD = −0.60), respectively. In stratified models by age group, LCSs decreased body weight in children (−1.06 kg; 95% CI: −1.57, −0.56; fixed-effect WGMD = −1.06) and adults (−0.72 kg; 95% CI: −1.15, −0.30; fixed-effect WGMD = 0.52). Results among children with data from Knopp et al (33) removed [−1.09 kg (95% CI: −1.70, −0.48); fixed-effect WGMD = −1.06] and results among adults with data from Kanders et al (32) removed [0.71 kg (95% CI: 1.14, −0.28); fixed-effect WGMD = −0.51] were not appreciably different from the results with these studies included. Analyses by sex showed significant reductions in body weight with LCSs among women (−0.72; 95% CI: −1.19, −0.25; fixed-effect WGMD = −0.62); the summary estimate for men was null but based on only 2 trials (no evidence of small-study bias was observed) (30, 32). Meta-analyses examining change in body weight by source of LCS were also limited because most studies examined LCSBs rather than foods or tabletop sweeteners (no evidence of small-study bias was observed; forest plot D).

BMI, fat mass, and waist circumference

The effects of LCS on BMI (kg/m^2; forest plot A), fat mass (kg; forest plot B), and waist circumference (cm; forest plot C), compared with the comparator arm, are shown in **Figure 3**. LCS significantly reduced BMI (0.24 kg/m^2; 95% CI: 0.41, −0.07; fixed-effect WGMD = −0.24), fat mass (−1.10; 95% CI: −1.77, −0.44; fixed-effect WGMD = −1.41), and waist circumference (−0.83; 95% CI: −1.29, −0.37; fixed-effect WGMD = −0.83). Additional results from subgroup analyses are shown elsewhere (*see* Supplemental Table 1 under "Supplemental data" in the online issue).

Meta-analysis results from prospective cohort studies

Meta-analyses of the prospective cohort studies were limited largely by differences across the individual studies; the models that were feasible are shown in **Figure 4** and elsewhere (*see* Supplemental Table 2 under "Supplemental data" in the online issue). Modest statistically significant positive associations between baseline LCS intake and change in BMI (WGMC: 0.03; 95% CI: 0.01, 0.06; fixed-effect WGMC = 0.03) are shown in Figure 4 (forest plot A). In the meta-analysis of LCS intake and weight gain (Figure 4; forest plot B) and fat mass (*see* Supplemental Table 2 under "Supplemental data" in the online issue), no statistically significant associations were observed, and statistical evidence for small-study bias was lacking (data not shown). Only one prospective cohort study examined waist circumference (specifically, risk of elevated waist circumference) (35); therefore, a meta-analysis examining the effect of LCS on waist circumference was not possible.

Publication bias

The symmetric funnel plot of RCTs that examined LCS and body weight (**Figure 5**; plot A), which was the largest set of studies, does not provide evidence of publication bias—a finding supported by Egger's regression test ($P = 0.164$). There was some evidence of publication bias among the prospective cohorts that examined BMI (the largest set of cohort studies), based on a visual assessment of the funnel plot (Figure 5; plot B), although this was not supported by Egger's regression test ($P = 0.818$).

DISCUSSION

The current meta-analysis provides a rigorous evaluation of the scientific evidence on LCS and body weight and composition. Findings from the meta-analysis of 15 RCTs—the gold standard study design in medical research—indicate that substituting LCS for sugar modestly reduces body weight, BMI, fat mass, and waist circumference. Although the mean reduction in body

TABLE 2
Characteristics of the prospective cohorts included in the meta-analysis[1]

Study	Cohort	Age[2]	Sex	BMI	Follow-up	Year diet assessed	Dietary-assessment method	LCS source or type	Results, energy-adjusted	Results, BMI-adjusted[3]	Outcome[4]
		y	*M/F*	*mean*	*y*						
Berkey, 2004 (48)	GUTS	9–14	5067/ 6688	NR	1	1997–1998	Validated youth FFQ	LCSB	Yes	Yes	BMI
Colditz, 1990 (52)	NHS	30–55	0/ 31940	23.4[5]	4	1980	Validated FFQ	Saccharin	Yes	Yes	BW
Fowler, 2008 (34)	SAHS	25–64	1421/ 1950	27.4	7.5	1979–88	24-h recall + survey questions	LCSB	No	Yes	BMI, overweight/ obesity incidence
Johnson, 2007 (51)[6]	CIF	7	471 (M+F)	16.2 (0.10 z score)	2	1999	3-d food records	LCSB	No	Yes	Fat mass[7]
Laska, 2012 (50)	IDEA + ECHO	10–17	276/ 286	22.0	2	2006–2008	4 validated survey questions	LCSB	Yes	No	BMI, fat mass[8]
Nettleton, 2009 (35)[8]	MESA	45–84	1307/ 1121	27.9	3–7	2000–2002	1 FFQ question	LCSB	Yes	No	Risk of elevated WC
Newby, 2004 (49)	ND WIC Program	2.9 ± 0.7	675/ 670	16.6	0.5–1	1995–1998	Validated FFQ	LCSB	Yes	No	BMI, BW
Parker, 1997 (46)	PHHP	18–64	176/ 289	26.5	4	1986–1987	Validated FFQ	Saccharin	Yes	Yes	BW
Schulze, 2004 (47)	NHS II	24–44	0/ 51603	24.5	4	1991–1999	3 validated FFQ questions	LCSB	No	Yes	BW

[1] BW, body weight; CIF, Children In Focus; ECHO, Etiology of Childhood Obesity; FFQ, food-frequency questionnaire; GUTS, Growing Up Today Study; IDEA, Identifying Determinants of Eating and Activity; LCS, low-calorie sweetener; LCSB, low-calorie sweetened beverage; MESA, Multi-Ethnic Study of Atherosclerosis; ND WIC, North Dakota Women, Infants, and Children; NHS, Nurses' Health Study; NR, not reported; PHHP, Pawtucket Heart Health Program; SAHS, San Antonio Heart Study; WC, waist circumference.

[2] Mean + SD when the age range was not reported.

[3] Adjustment for other baseline body-composition measures was eligible but not performed in any studies.

[4] Reflects change in the measure from baseline, unless noted otherwise.

[5] Mean BMI was estimated from categorical data provided in the article.

[6] Findings among 7-y-olds are shown; authors also report findings among a smaller sample of the population at 5 y.

[7] Measured by dual-energy X-ray absorptiometry.

[8] Measured by bioelectrical impedance analysis.

[9] Met eligibility criteria but was not included in the meta-analysis because it was the only study with risk of elevated WC as an outcome.

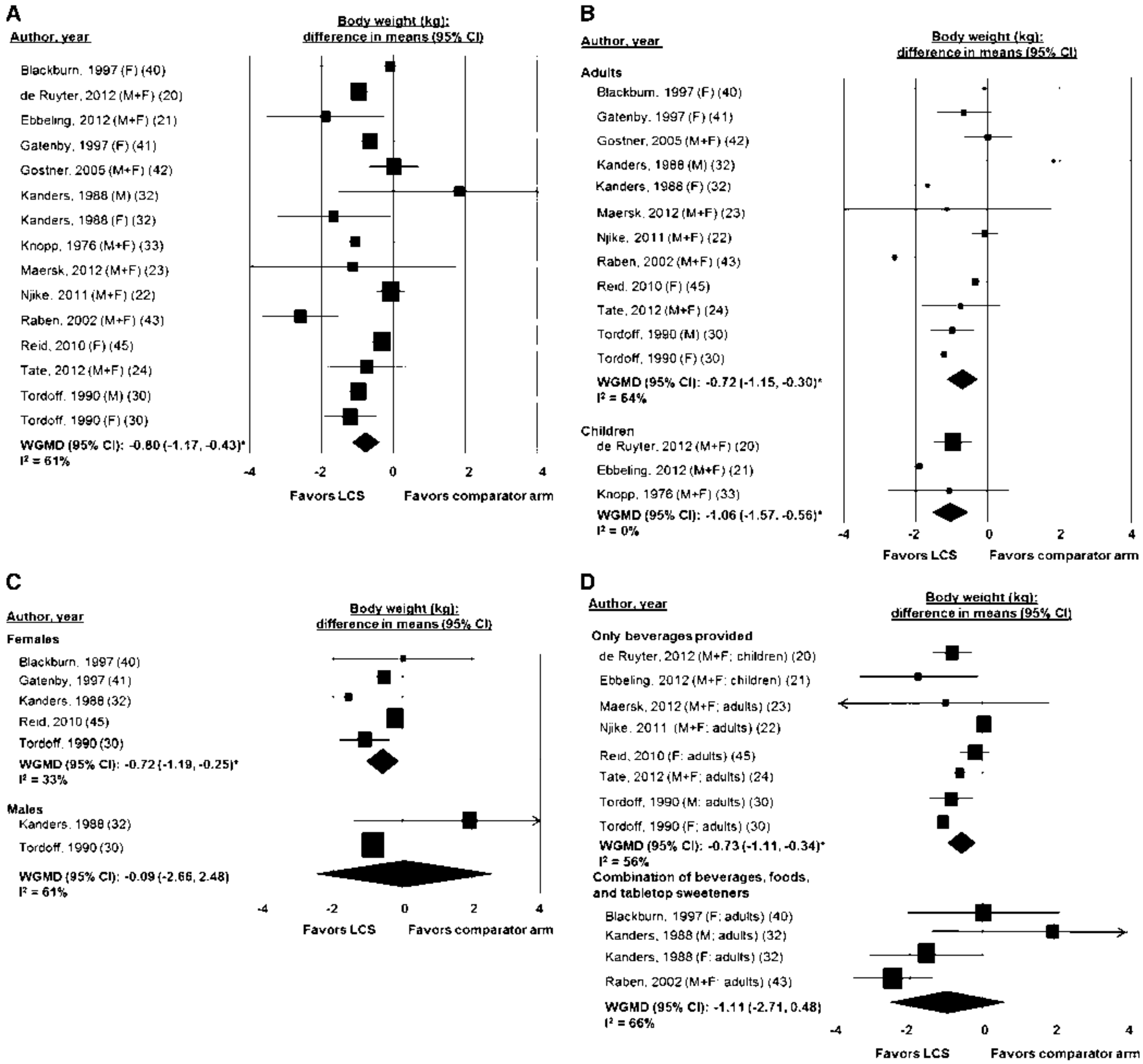

FIGURE 2. Forest plots derived from random-effects models depicting the effect of LCS on body weight in RCTs among all subjects (A) and by age (B), sex (C), and source of LCS (D). Squares represent mean change in body weight within the individual studies; 95% CIs are represented by horizontal lines. Square size is proportional to the weight of each study. Diamonds represent the WGMD. *$P < 0.05$. LCS, low-caloric sweetener; RCT, randomized controlled trial; WGMD, weighted group mean difference.

weight was modest (0.80-kg decrease), it would not be expected for a single dietary change, ie, replacement of sugar with LCS, to cause clinically meaningful weight loss (53). Rather, leading nutrition and health authorities recommend a multifaceted approach to weight loss and weight maintenance—one that includes an overall healthy dietary pattern, physical activity, and other lifestyle behavior changes (54, 55). By maintaining the palatability of foods and beverages with fewer calories than sugar, LCS could help improve adherence to weight-loss or maintenance plans (5).

The current meta-analysis also examined the relation between LCS intake and body weight and composition among prospective cohort studies because experimental and observational research methods can be complementary tools in understanding diet-health relations. This meta-analysis showed statistically nonsignificant associations between LCS intake and body weight and fat mass, but a significant, albeit modest, positive association with BMI. Compared with findings from well-controlled, randomized trials, wherein reported effects can be attributed to the dietary intervention under investigation (56), findings from observational studies in the field of nutrition are not easily interpreted. Specifically, the meta-analysis of prospective cohort studies was limited because few studies (46, 48, 52) adequately controlled for potential confounding by other diet and lifestyle factors. Only 3

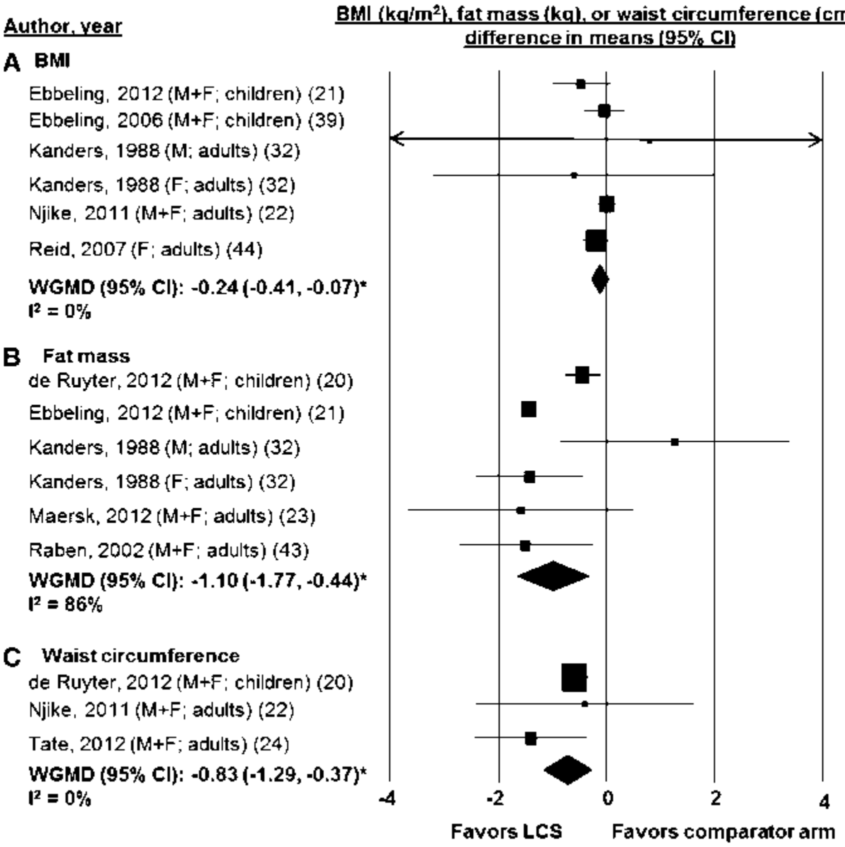

FIGURE 3. Forest plots derived from random-effects models depicting the effects of LCS on BMI (A), fat mass (B), and waist circumference (C) in RCTs. Squares represent mean change within the individual studies; 95% CIs are represented by horizontal lines. Square size is proportional to the weight of each study. Diamonds represent the WGMD. *$P < 0.05$. LCS, low-calorie sweeteners; RCT, randomized controlled trial; WGMD, weighted group mean difference.

studies (46, 48, 52) controlled for both total energy intake and a measure of baseline body weight or composition. Several other potential sources of bias include the possibility of reverse causality and dietary measurement error (57)—2 methodologic issues that were not sufficiently addressed in most studies included in the meta-analysis. Importantly, 7 (35, 46–50, 52) of the prospective cohorts assessed LCS intake at baseline, and only a few survey or food-frequency questionnaire questions pertained to LCS intake (largely consumption of diet soda). This insufficient measurement of LCS intake provides limited information on individual intakes and, as a result, may have biased the reported associations with body weight and composition (58).

Variations in overall dietary patterns among subjects in observational studies should be considered in the study of LCSs and body weight and composition because individuals who consume LCSs may have differential patterns of eating compared with those who do not (59). Recent findings from the Choose Healthy Options Consciously Everyday RCT provide supporting evidence that LCS intake plays a role in influencing overall dietary patterns (9). In this 6-mo study, replacement of regular-calorie beverages with either water in one study arm or LCSBs in a second study arm resulted in significant changes in other food and nutrient intakes. Both groups consumed less total energy, whereas intakes of desserts, caloric sweeteners, and alcohol were significantly reduced in the LCSB group but not in the water group. This finding provides suggestive evidence that LCSs do not, contrary to past hypotheses (6, 7), increase the desire or inclination to consume more sweet foods. Taken together, observational and experimental investigations into LCS intake as part of overall dietary patterns provide useful insight into how individuals are currently consuming LCSs and the effect of LCS intake on dietary patterns. In turn, these findings may be useful in informing the development of dietary guidelines and public health recommendations.

In both the prospective cohort studies and RCTs, the sources and types of LCSs investigated were limited. Seven (34, 35, 47–51) of the 9 prospective cohorts examined intakes of LCSBs, which is just one of many sources of LCS in the diet. The other 2 cohort studies (46, 52) investigated intakes of only one type of LCS (saccharin). There was more diversity in the sources and types of LCS evaluated among the RCTs, although 9 (20–24, 30, 39, 44, 45) of the 15 studies exclusively examined LCSBs. The others evaluated aspartame (32, 33, 40), unspecified LCSs (41), isomalt (42), and a combination of aspartame, cyclamate, acesulfame potassium, and saccharin (43). In addition to the limited types and sources of LCS examined, far fewer studies examined the effect of LCSs on BMI, fat mass, and waist circumference compared with body weight. Nevertheless, the direction of effects was the same across the different outcomes, and all reductions were statistically significant.

Only one RCT (33) examined the effect of capsules of LCS (specifically aspartame) on body weight. The main research objective in the RCT by Knopp et al (33)—to evaluate potential

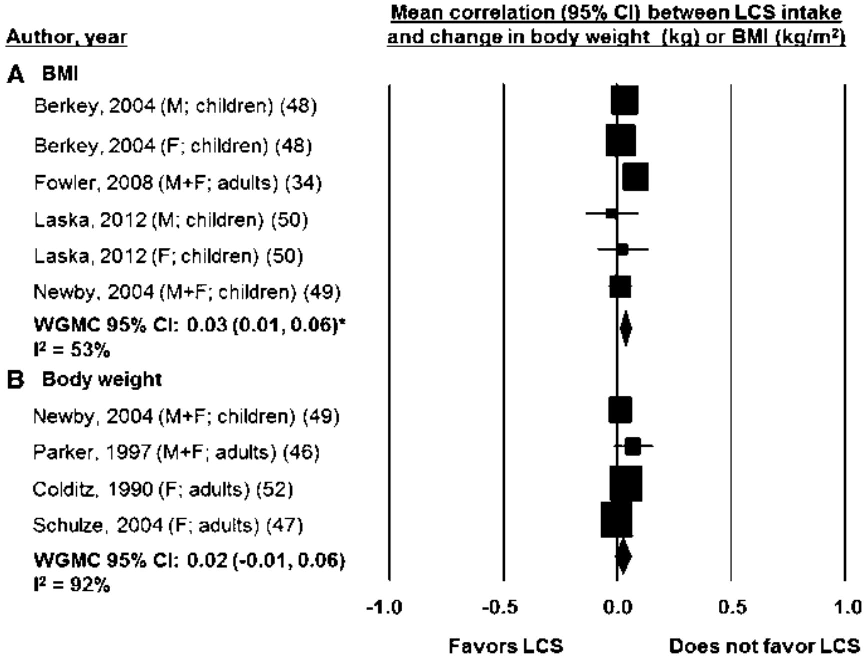

FIGURE 4. Forest plots derived from random effects models summarizing results from the meta-analysis of prospective cohort studies that examined LCS intake and change in BMI (A) or body weight (B). The squares represent the mean correlation within each study, with 95% CIs represented by horizontal lines. Square size is proportional to the weight of each study. Diamonds represent the WGMC. Reference numbers are shown in parentheses. $^*P < 0.05$. LCS, low-calorie sweetener; WGMC, weighted group mean correlation.

toxicity from aspartame intake administered in capsule form—is inherently different from the objectives in the other RCTs, which were designed to examine the effects of LCS as a sugar substitute (provided in foods, in beverages, or as tabletop sweeteners). Body weight was not a primary outcome in the study by Knopp et al; however, because it was measured and the study met a priori inclusion criteria, it was included in the current meta-analysis. Knopp et al found nonstatistically significant reductions in body weight, and removal of this RCT from the meta-analysis did not appreciably influence the summary findings.

Past reviews examining the relation between LCS and body weight have focused solely on one type of LCS (18) or have been qualitative in nature (5, 16, 17, 19, 60). Two of the recent qualitative reviews (16, 19) noted a lack of evidence to draw firm conclusions and called for additional research, including long-term relatively large trials, to advance understanding and address key questions. The current systematic review and meta-analysis aimed to address many of these questions by quantitatively summarizing results from RCTs, 5 of which have been published since 2011 (20–24). One of these trials (20)—the largest (n = 641 enrolled) and longest (18 mo) to date—found that replacement of SSBs with LCSBs reduced weight gain and fat accumulation in normal-weight children aged 4–11 y.

Although the body of evidence on LCSs and body weight has grown in recent years, several research questions remain. Examinations into specific LCSs, particularly understudied yet commonly used LCSs such as polyols, sucralose, and steviol glycosides, are warranted. Research into the role of LCSs as part of overall dietary patterns would provide important insight for developing guidelines and public health recommendations. Few studies provided separate estimates for men and women, which limited evaluations of sex-specific effects. Observational studies that use new dietary-assessment tools, such as those that integrate technology in mobile phones with image processing, visualization, and food and nutrient databases (61), have the potential to substantially improve the quality and validity of dietary intake data and thus studies that depend on these observational data. In addition, the inclusion of additional LCSs and products sweetened with LCS into food and nutrient databases would facilitate comprehensive investigations into the relation between LCS intake and body weight and composition.

In conclusion, the meta-analysis of observational studies showed a small positive association between LCS intake and BMI, but no association with body weight or fat mass. On the other hand, data from RCTs, which provide the highest quality of evidence for examining the potentially causal effects of LCS intake on body weight, indicate that substituting LCSs for calorically dense alternatives results in a modest reduction of body weight, BMI, fat mass, and waist circumference. Compared with the consistent findings among the RCTs, results from prospective cohort studies were limited and more difficult to interpret, particularly because of inadequate control of important confounders, including total energy intake and baseline differences between LCS consumers and nonconsumers in body weight and composition. On the basis of the available scientific literature to date, substituting LCS options for their regular-calorie versions results in a modest weight loss and may be a useful dietary tool to improve compliance with weight-loss or weight-maintenance plans.

We acknowledge the International Life Sciences Institute Low-Caloric Sweetener Committee for providing feedback and review of the study protocol and manuscript.

The authors' responsibilities were as follows—PEM: conceptualized the study, conducted the literature review, extracted the study data, and designed the study; VP: conducted a separate data extraction for quality control and

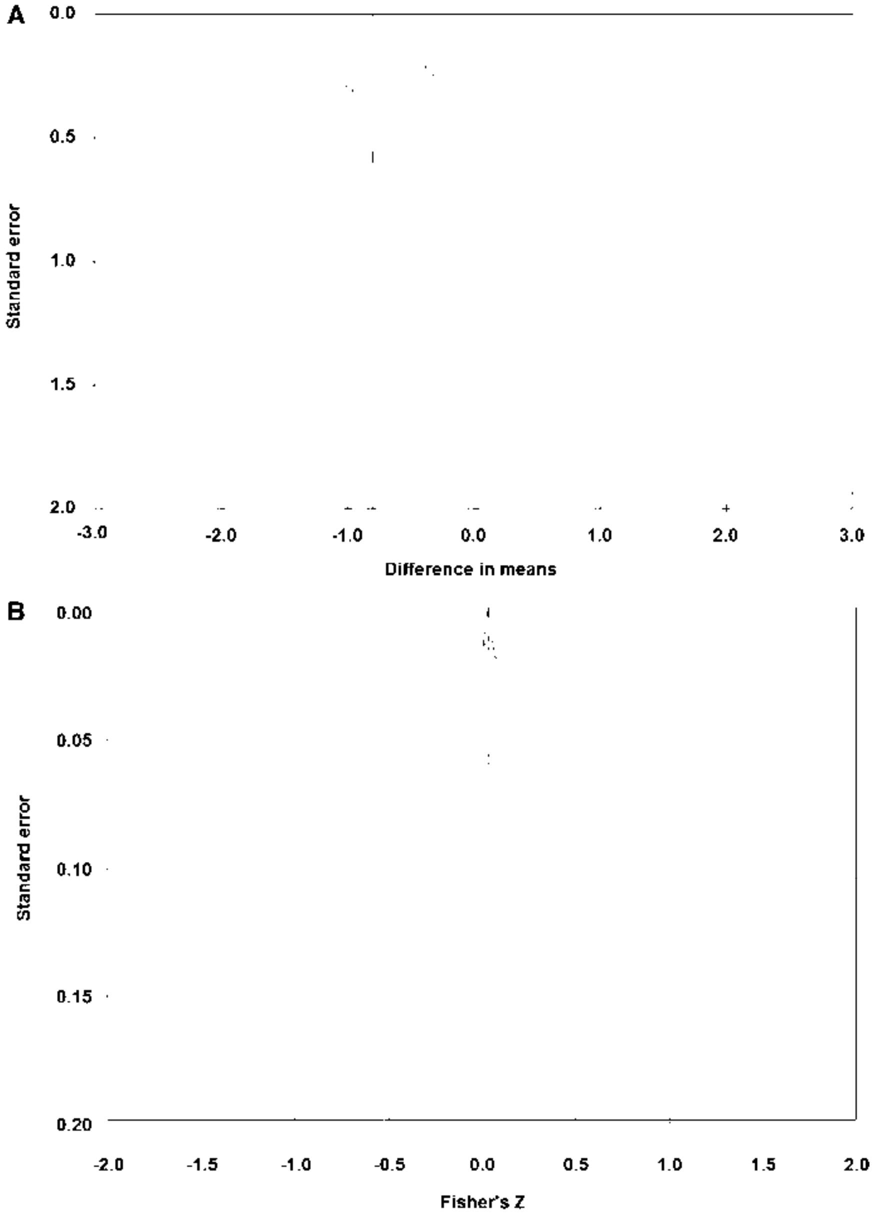

FIGURE 5. Funnel plots for the detection of publication bias among RCTs that examined body weight (A) and prospective cohort studies that examined BMI (B). The x axis represents the effect size of each RCT (A) or the Fisher-transformed correlation value of each prospective cohort study (B). The y axis represents the SE of the effect size (A) or the correlation value (B) of the corresponding study. The solid vertical line is the pooled summary estimate from the meta-analysis. RCT, randomized controlled trial.

conducted the meta-analyses; PEM and VP: interpreted the results; PEM: drafted the manuscript, with substantial support from VP; and both authors critically reviewed the manuscript for intellectual content. PEM and VP received funding to conduct this research from the North American Branch of the International Life Sciences Institute (ILSI). ILSI had no role in the study design, data collection and analysis, interpretation of the data, or preparation of the manuscript. At the time this research was completed, PEM was employed at Exponent. Neither of the authors had a conflict of interest.

REFERENCES

1. Flegal KM, Carroll MD, Kit BK, Ogden CL. Prevalence of obesity and trends in the distribution of body mass index among US adults, 1999-2010. JAMA 2012;307:491–7.
2. Ogden CL, Carroll MD, Kit BK, Flegal KM. Prevalence of obesity and trends in body mass index among US children and adolescents, 1999-2010. JAMA 2012;307:483–90.
3. US Department of Health and Human Services. The Surgeon General's call to action to prevent and decrease overweight and obesity. Washington, DC: US Government Printing Office, 2001.
4. Fitch C, Keim KS. Academy of Nutrition and Dietetics. Position of the Academy of Nutrition and Dietetics: use of nutritive and nonnutritive sweeteners. J Acad Nutr Diet 2012;112:739–58.
5. Foreyt J, Kleinman R, Brown RJ, Lindstrom R. The use of low-calorie sweeteners by children: implications for weight management. J Nutr 2012;142:1155S–62S.
6. Stellman SD, Garfinkel L. Artificial sweetener use and one-year weight change among women. Prev Med 1986;15:195–202.
7. Blundell JE, Hill AJ. Paradoxical effects of an intense sweetener (aspartame) on appetite. Lancet 1986;1:1092–3.

8. Mattes RD, Popkin BM. Nonnutritive sweetener consumption in humans: effects on appetite and food intake and their putative mechanisms. Am J Clin Nutr 2009;89:1–14.
9. Piernas C, Tate DF, Wang X, Popkin BM. Does diet-beverage intake affect dietary consumption patterns? Results from the Choose Healthy Options Consciously Everyday (CHOICE) randomized clinical trial. Am J Clin Nutr 2013;97:604–11.
10. Kroger M, Meister K, Kava R. Low-calorie sweeteners and other sugar substitutes: a review of the safety issues. In: Comprehensive Reviews in Food Science and Food Safety 2006:35–47.
11. Calorie Control Council. Polyols. Version current 2013. Available from: http://www.caloriecontrol.org/sweeteners-and-lite/polyols (cited 20 October 2013).
12. Commission Regulation (EU) No 1129/2011 of 11 November 2011 amending Annex II to Regulation (EC) No 1333/2008 of the European Parliament and of the Council by establishing a Union list of food additives. Offic J European Union 2011;54:1–177.
13. US Food and Drug Administration. Everything added to food in the United States (EAFUS). Version current 23 April 2013. Available from: http://www.accessdata.fda.gov/scripts/fcn/fcnnavigation.cfm?rpt=eafuslisting (cited 20 October 2013).
14. Commission Regulation (EU) No 1050/2012 of 8 November 2012 amending Regulation (EU) No 231/2012 laying down specifications for food additives listed in Annexes II and III to Regulation (EC) No 1333/2008 of the European Parliament and of the Council as regards polyglycitol syrup. Offic J European Union 2012;310:45–6.
15. Raben A, Richelsen B. Artificial sweeteners: a place in the field of functional foods? Focus on obesity and related metabolic disorders. Curr Opin Clin Nutr Metab Care 2012;15:597–604.
16. Anderson GH, Foreyt J, Sigman-Grant M, Allison DB. The use of low-calorie sweeteners by adults: impact on weight management. J Nutr 2012;142:1163S–9S.
17. Bellisle F, Drewnowski A. Intense sweeteners, energy intake and the control of body weight. Eur J Clin Nutr 2007;61:691–700.
18. de la Hunty A, Gibson S, Ashwell M. A review of the effectiveness of aspartame in helping with weight control. Nutr Bull 2006;31:115–28.
19. Pereira MA, Odegaard AO. Artificially sweetened beverages–do they influence cardiometabolic risk? Curr Atheroscler Rep 2013;15:375.
20. de Ruyter JC, Olthof MR, Seidell JC, Katan MB. A trial of sugar-free or sugar-sweetened beverages and body weight in children. N Engl J Med 2012;367:1397–406.
21. Ebbeling CB, Feldman HA, Chomitz VR, Antonelli TA, Gortmaker SL, Osganian SK, Ludwig DS. A randomized trial of sugar-sweetened beverages and adolescent body weight. N Engl J Med 2012;367:1407–16.
22. Njike VY, Faridi Z, Shuval K, Dutta S, Kay CD, West SG, Kris-Etherton PM, Katz DL. Effects of sugar-sweetened and sugar-free cocoa on endothelial function in overweight adults. Int J Cardiol 2011;149:83–8.
23. Maersk M, Belza A, Stodkilde-Jorgensen H, Ringgaard S, Chabanova E, Thomsen H, Pedersen SB, Astrup A, Richelsen B. Sucrose-sweetened beverages increase fat storage in the liver, muscle, and visceral fat depot: a 6-mo randomized intervention study. Am J Clin Nutr 2012;95:283–9.
24. Tate DF, Turner-McGrievy G, Lyons E, Stevens J, Erickson K, Polzien K, Diamond M, Wang X, Popkin B. Replacing caloric beverages with water or diet beverages for weight loss in adults: main results of the Choose Healthy Options Consciously Everyday (CHOICE) randomized clinical trial. Am J Clin Nutr 2012;95:555–63.
25. Moher D, Liberati A, Tetzlaff J, Altman DG. Preferred reporting items for systematic reviews and meta-analyses: the PRISMA statement. PLoS Med 2009;6:e1000097.
26. Methods Guide for Effectiveness and Comparative Effectiveness Reviews. AHRQ publication no. 10(13)-EHC063-EF. Rockville, MD: Agency for Healthcare Research and Quality. Version current 2013. Available from: www.effectivehealthcare.ahrq.gov (cited 1 October 2013).
27. Lichtenstein AH, Yetley EA, Lau J. Application of systematic review methodology to the field of nutrition. J Nutr 2008;138:2297–306.
28. Te Morenga L, Mallard S, Mann J. Dietary sugars and body weight: systematic review and meta-analyses of randomised controlled trials and cohort studies. BMJ 2013;346:e7492.
29. Malik VS, Schulze MB, Hu FB. Intake of sugar-sweetened beverages and weight gain: a systematic review. Am J Clin Nutr 2006;84:274–88.
30. Tordoff MG, Alleva AM. Effect of drinking soda sweetened with aspartame or high-fructose corn syrup on food intake and body weight. Am J Clin Nutr 1990;51:963–9.
31. Higgins JPT, Green S. Cochrane Collaboration. Cochrane handbook for systematic reviews of interventions. Hoboken, NJ: Wiley-Blackwell. 2008.
32. Kanders BS, Lavin PT, Kowalchuk MB, Greenberg I, Blackburn GL. An evaluation of the effect of aspartame on weight loss. Appetite 1988;11(suppl 1):73–84.
33. Knopp RH, Brandt K, Arky RA. Effects of aspartame in young persons during weight reduction. J Toxicol Environ Health 1976;2:417–28.
34. Fowler SP, Williams K, Resendez RG, Hunt KJ, Hazuda HP, Stern MP. Fueling the obesity epidemic? Artificially sweetened beverage use and long-term weight gain. Obesity (Silver Spring) 2008;16:1894–900.
35. Nettleton JA, Lutsey PL, Wang Y, Lima JA, Michos ED, Jacobs DR Jr. Diet soda intake and risk of incident metabolic syndrome and type 2 diabetes in the Multi-Ethnic Study of Atherosclerosis (MESA). Diabetes Care 2009;32:688–94.
36. Higgins JP, Thompson SG, Deeks JJ, Altman DG. Measuring inconsistency in meta-analyses. BMJ 2003;327:557–60.
37. Becker BJ, Wu M-J. The synthesis of regression slopes in meta-analysis. Stat Sci 2007;22:414–29.
38. Egger M, Davey Smith G, Schneider M, Minder C. Bias in meta-analysis detected by a simple, graphical test. BMJ 1997;315:629–34.
39. Ebbeling CB, Feldman HA, Osganian SK, Chomitz VR, Ellenbogen SJ, Ludwig DS. Effects of decreasing sugar-sweetened beverage consumption on body weight in adolescents: a randomized, controlled pilot study. Pediatrics 2006;117:673–80.
40. Blackburn GL, Kanders BS, Lavin PT, Keller SD, Whatley J. The effect of aspartame as part of a multidisciplinary weight-control program on short- and long-term control of body weight. Am J Clin Nutr 1997;65:409–18.
41. Gatenby SJ, Aaron JI, Jack VA, Mela DJ. Extended use of foods modified in fat and sugar content: nutritional implications in a free-living female population. Am J Clin Nutr 1997;65:1867–73.
42. Gostner A, Schaffer V, Theis S, Menzel T, Luhrs H, Melcher R, Schauber J, Kudlich T, Dusel G, Dorbath D, et al. Effects of isomalt consumption on gastrointestinal and metabolic parameters in healthy volunteers. Br J Nutr 2005;94:575–81.
43. Raben A, Vasilaras TH, Moller AC, Astrup A. Sucrose compared with artificial sweeteners: different effects on ad libitum food intake and body weight after 10 wk of supplementation in overweight subjects. Am J Clin Nutr 2002;76:721–9.
44. Reid M, Hammersley R, Hill AJ, Skidmore P. Long-term dietary compensation for added sugar: effects of supplementary sucrose drinks over a 4-week period. Br J Nutr 2007;97:193–203.
45. Reid M, Hammersley R, Duffy M. Effects of sucrose drinks on macronutrient intake, body weight, and mood state in overweight women over 4 weeks. Appetite 2010;55:130–6.
46. Parker DR, Gonzalez S, Derby CA, Gans KM, Lasater TM, Carleton RA. Dietary factors in relation to weight change among men and women from two southeastern New England communities. Int J Obes Relat Metab Disord 1997;21:103–9.
47. Schulze MB, Manson JE, Ludwig DS, Colditz GA, Stampfer MJ, Willett WC, Hu FB. Sugar-sweetened beverages, weight gain, and incidence of type 2 diabetes in young and middle-aged women. JAMA 2004;292:927–34.
48. Berkey CS, Rockett HR, Field AE, Gillman MW, Colditz GA. Sugar-added beverages and adolescent weight change. Obes Res 2004;12:778–88.
49. Newby PK, Peterson KE, Berkey CS, Leppert J, Willett WC, Colditz GA. Beverage consumption is not associated with changes in weight and body mass index among low-income preschool children in North Dakota. J Am Diet Assoc 2004;104:1086–94.
50. Laska MN, Murray DM, Lytle LA, Harnack LJ. Longitudinal associations between key dietary behaviors and weight gain over time: transitions through the adolescent years. Obesity (Silver Spring) 2012;20:118–25.
51. Johnson L, Mander AP, Jones LR, Emmett PM, Jebb SA. Is sugar-sweetened beverage consumption associated with increased fatness in children? Nutrition 2007;23:557–63.
52. Colditz GA, Willett WC, Stampfer MJ, London SJ, Segal MR, Speizer FE. Patterns of weight change and their relation to diet in a cohort of healthy women. Am J Clin Nutr 1990;51:1100–5.

53. Stevens J, Truesdale KP, McClain JE, Cai J. The definition of weight maintenance. Int J Obes (Lond) 2006;30:391–9.
54. Seagle HM, Strain GW, Makris A, Reeves RS. American Dietetic A. Position of the American Dietetic Association: weight management. J Am Diet Assoc 2009;109:330–46.
55. USDA, US Department of Health and Human Services. Dietary guidelines for Americans, 2010. 7th ed. Washington, DC: US Government Printing Office, 2010.
56. Moher D, Jones A, Lepage L, Group C. Use of the CONSORT statement and quality of reports of randomized trials: a comparative before-and-after evaluation. JAMA 2001;285:1992–5.
57. Flegal KM. Evaluating epidemiologic evidence of the effects of food and nutrient exposures. Am J Clin Nutr 1999;69:1339S–44S.
58. Subar AF, Thompson FE, Kipnis V, Midthune D, Hurwitz P, McNutt S, McIntosh A, Rosenfeld S. Comparative validation of the Block, Willett, and National Cancer Institute food frequency questionnaires: the Eating at America's Table Study. Am J Epidemiol 2001;154:1089–99.
59. Jacobs DR Jr, Steffen LM. Nutrients, foods, and dietary patterns as exposures in research: a framework for food synergy. Am J Clin Nutr 2003;78:508S–13S.
60. Brown RJ, de Banate MA, Rother KI. Artificial sweeteners: a systematic review of metabolic effects in youth. Int J Pediatr Obes 2010;5:305–12.
61. Thompson FE, Subar AF, Loria CM, Reedy JL, Baranowski T. Need for technological innovation in dietary assessment. J Am Diet Assoc 2010;110:48–51.

Obesity

Can Non-Nutritive Sweeteners Enhance Outcomes of Weight Loss Interventions?

Stephen D. Anton[1,2]

With obesity reaching epidemic proportions in many countries around the world, the search for the causes of and potential solutions for this condition continues. It is noteworthy that the increase in the consumption of added or discretionary sugars has mirrored the dramatic rise in the prevalence of obesity over the past few decades. Although certainly not the only factor contributing to the obesity epidemic, a large body of evidence now exists indicating that consumption of sugar (sucrose) sweetened beverages promotes weight gain in both children and adults (1). Moreover, some experts have recently argued that there is now sufficient scientific evidence to conclude that decreasing sugar-sweetened beverage consumption will reduce the prevalence of obesity and obesity-related diseases (2). Accordingly, the potential value of replacing sucrose with low-caloric sweeteners, also known as non-nutritive sweeteners (NNS), for promoting weight loss has become a topic of increasing scientific interest.

To date, research has produced mixed findings regarding the effects of NNS on body weight. Although many studies have found that the consumption of NNS is associated with weight loss, other studies have found that some NNS may actually stimulate appetite and ultimately lead to weight gain. In a 2009 review (3), Mattes & Popkin concluded that "The addition of NNS to diets poses no benefit for weight loss or reduced weight gain without energy restriction." However, the authors also specified that "If non-nutritive sweeteners are used as substitutes for higher energy yielding sweeteners, they have the potential to aid in weight management." Given that consumption of foods and beverages containing NNS has significantly increased during the past decade (3), it is critical to understand the potential impact that use of NNS may have on body weight, as well as the potential role that NNS may have in weight loss interventions.

This important question was recently explored in a prospective, randomized trial in which consumption of water versus NNS beverages was compared within the context of a behavioral weight loss program. In this large-scale trial conducted by Peters and colleagues (4), a total of 303 overweight and obese (Class I and II) participants were assigned to one of two treatment conditions (1) NNS, in which participants were instructed to consume 24 ounces of NNS beverages per day (water consumption was not restricted) or (2) Water group, in which participants were instructed to consume at least 24 ounces of water per day and not drink any NNS beverages. Participants in this condition were, however, allowed to consume NNS in foods. Both groups received a one year community based lifestyle intervention (i.e. The Colorado Weigh) in which equal emphasis was placed on diet and physical activity changes. Participants' energy intake targets were set to equal their resting metabolic rate (RMR), with these targets adjusted as needed by group leaders so as to produce a weight loss of 1-2 pounds per week.

The recent article by Peters et al. (2014) (4) reports the findings from the first 12 weeks of this intervention, with the key finding being that participants in the NNS beverage treatment group lost significantly more weight than participants in the water treatment group (5.5 kg versus 3.8 kg). Weekly hungers scores also decreased to a greater extent among participants in the NNS group compared to participants in the Water group; however, the absolute magnitude of this reduction was small. Participants in the NNS treatment group also had greater reductions in total and LDL cholesterol, which the authors note may have been due to the greater weight loss observed in this group. Sedentary behavior decreased significantly in the Water group over time but not in the NNS group; however, there were no differences in changes in physical activity or sedentary behavior between groups.

The authors are to be commended on conducting this large-scale and comprehensive trial. Several notable strengths of this study are worth mentioning. First, the study was conducted at two research sites (i.e., Colorado and Temple), increasing confidence in the generalizability of the study findings. Second, both sexes were included, and there was a similar distribution of men and women in both treatment sites (approximately 80% women in both groups). Minority individuals were also well represented with approximately 27% of the participants being African American. Additionally, adherence to both the NNS and Water interventions was excellent (>95% in both groups), as was participant retention, with 92% of the participants who started treatment completing the 12-week follow-up assessments.

Although this clinical trial had a number of strengths and represents an important contribution to the literature, there are a few important questions that are left unanswered by this study. First, twelve weeks is a short period of observation, really just a preliminary look at the outcome. We look forward to reports of at least a year, which will provide the critical information needed to more fully evaluate the effects of NNS on weight loss outcomes.

Second, intake of NNS does not appear to have been well controlled since participants in both conditions were allowed to consume NNS, with the only restriction being that participants in the water group

[1] Department of Aging and Geriatric Research, College of Medicine, University of Florida, Gainesville, FL, USA. Correspondence: Stephen D. Anton (santon@ufl.edu) [2] Department of Clinical and Health Psychology, University of Florida, Gainesville, FL

Received: 10 April 2014; **Accepted:** 14 April 2014; **Published online 27 May 2014.** **doi:10.1002/oby.20779**

could not consume NNS in beverages. Since all enrolled participants were required to consume three or more NNS beverages per week to be eligible to participate in this study, many participants in the NNS group may have already been consuming NNS beverages at the levels tested in this study. Although adherence to NNS beverage consumption was high (97%), no information is provided on the actual amount of NNS consumed through either beverages or food prior to study enrollment or during study participation. Thus, it is difficult to know to what extent participants in the NNS treatment group increased the amount of NNS consumed, if at all, during study participation. Similarly, it is unknown whether participants in the Water group decreased consumption of NNS since participants in this group were allowed to consume NNS in foods.

Another important issue that was not answered by this article is the potential mechanism through which the NNS beverage consumption may have produced greater weight loss, in comparison to water consumption. Since the two treatment groups did not differ in their changes in physical activity or sedentary behavior, the findings of this study would suggest that the greater weight loss observed among the participants in the NNS group was due to larger reductions in caloric intake. Unfortunately, no information is provided on participant's caloric intake or adherence to dietary recommendations. Without this critical information, it is difficult to know if dietary intake differed between groups or if another potential mechanism may be responsible for the observed weight loss outcomes. A third group in which participants received the same instructions for NNS consumption without a comprehensive behavioral weight loss intervention could help clarify the potential mechanism(s) for the observed effects.

Another critical issue that was not addressed in this study is whether the different types of NNS have the same or differential effects on weight loss and metabolic outcomes. In the Peters et al study (4), participants were allowed to consume any type of NNS (examples include aspartame, NutraSweet or Equal, sucralose-Splenda, stevia-Truvia, as well as diet creamers). Previous studies suggest, however, that not all NNS have the same metabolic effects. For example, intake of the NNS, stevia, has been found to produce a smaller postprandial release of glucose and insulin compared to the NNS, aspartame, even when caloric content of meals are held constant (5). At present, the long-term effects of these different NNS on body weight, food intake, and other metabolic outcomes are currently unknown.

In summary, the findings of the Peters et al. (2014) study (4) provide an important contribution to the literature and strongly suggest that individuals who consume NNS should not be discouraged from continuing to consume these beverages during weight loss efforts. These findings, however, should be interpreted with caution since longer term outcomes are needed to confirm these results and the potential mechanism(s) for the superior effects of NNS on weight loss outcomes observed in this study is currently unknown. Additionally, the generalizability of these findings is limited to individuals who regularly consume NNS, and it is unknown if similar effects would be observed among individuals who do not regularly consume NNS. Future studies are also needed to determine if all NNS have similar metabolic effects or if these effects differ between the different types of NNS. Until such data become available, it is advised that NNS are not widely recommended in weight loss programs, particularly for individuals who do not regularly consume them. O

Acknowledgments

Support was provided by the University of Florida's Claude D. Pepper Older Americans Independence Center (NIH/NIA P30AG028740), and Clinical and Translational Science Institute (NIH/NCRR UL1TR000064). Stephen Anton is supported by a K23 AT004251-01A2 and previously by the Thomas H. Maren Foundation.

© 2014 The Obesity Society

References

1. Malik VS, Pan A, Willett WC, Hu FB. Sugar sweetened beverages and weight gain in children and adults: a systematic review and meta-analysis. *Am J Clin Nutr* 2013; 98:1084-1102.
2. Hu FB. Resolved: there is sufficient scientific evidence that decreasing sugar sweetened beverage consumption will reduce the prevalence of obesity and obesity-related diseases. *Obes Rev* 2013;14:606-619.
3. Mattes RD, Popkin BM. Nonnutritive sweetener consumption in humans: effects on appetite and food intake and their putative mechanisms. *Am J Clin Nutr* 2009;89: 1-14.
4. Peters J, Wyatt H, Foster G et al. The effects of water and non-nutritive sweetened beverages on weight loss during a 12 week weight loss treatment program. *Obesity*. In press.
5. Anton SD, Martin CK, Han H et al. Effects of stevia, aspartame, and sucrose on food intake, satiety, and postprandial glucose and insulin levels. *Appetite* 2010;55: 37-43.

The Effects of Water and Non-Nutritive Sweetened Beverages on Weight Loss During a 12-week Weight Loss Treatment Program

John C. Peters[1], *Holly R. Wyatt*[1], *Gary D. Foster*[2], *Zhaoxing Pan*[1], *Alexis C. Wojtanowski*[2], *Stephanie S. Vander Veur*[2], *Sharon J. Herring*[2], *Carrie Brill*[1] *and James O. Hill*[1]

Objective: To compare the efficacy of non-nutritive sweetened beverages (NNS) or water for weight loss during a 12-week behavioral weight loss treatment program.

Methods: An equivalence trial design with water or NNS beverages as the main factor in a prospective randomized trial among 303 men and women was employed. All participants participated in a behavioral weight loss treatment program. The results of the weight loss phase (12 weeks) of an ongoing trial (1 year) that is also evaluating the effects of these two treatments on weight loss maintenance were reported.

Results: The two treatments were not equivalent with the NNS beverage treatment group losing significantly more weight compared to the water group (5.95 kg versus 4.09 kg; $P < 0.0001$) after 12 weeks. Participants in the NNS beverage group reported significantly greater reductions in subjective feelings of hunger than those in the water group during 12 weeks.

Conclusion: These results show that water is not superior to NNS beverages for weight loss during a comprehensive behavioral weight loss program.

Obesity (2014) **22**, 1415-1421. doi:10.1002/oby.20737

Introduction

Beverage consumption recommendations (1) suggest water as the gold-standard beverage for optimal health. The US Dietary Guidelines (2) suggest that while beverages with non-nutritive sweeteners (NNS) are preferable to those with caloric sweeteners, there is still a question about whether they are beneficial for weight management. While numerous clinical trials have examined the effects of nutritive sugar sweetened beverages (NS) compared to NNS beverages on weight loss, few studies have directly compared water and NNS beverages on weight loss using an equivalence trial design.

NNS were introduced into the food supply over 50 years ago and are being used in hundreds of different food and beverage products. Despite the long history of usage there continues to be considerable controversy concerning their role in the diet, particularly whether they are a useful tool as an aid in weight loss and weight loss maintenance (3-6). NNS provide sweetness equivalent to NS but contribute essentially zero energy. Since the 1980s a number of short-term experimental studies have compared NNS to NS and several comprehensive reviews have concluded that the evidence supports either a beneficial effect or no effect of NNS on appetite and energy intake (7-11). Other studies have reported findings of increased hunger with consumption of NNS (11) but generally without an accompanying increase in caloric intake.

Several observational studies have reported a positive association between NNS consumption and greater body weight and weight gain over time (4,12). Determining causality is not possible with these studies but it is possible that they represent "reverse causality" whereby obesity may cause people to seek diet beverages (10,13).

The largest and most recent randomized trial (14) to compare water, diet beverages and an attention control for their effects on weight loss used a superiority trial design. The authors found that the diet beverage treatment, but not water, significantly increased the probability of losing 5% of body weight over the 6-month study duration compared to a standard weight loss education and monitoring program. Subjects in both treatment groups lost a significant amount of weight but the amount of weight lost compared to the control was not different between treatment groups.

[1] Anschutz Health and Wellness Center, University of Colorado, Anschutz Medical Campus, Aurora, CO, USA. Correspondence: John C. Peters (john.c.peters@ucdenver.edu) [2] Temple University, Center for Obesity Research and Education, Department of Medicine, Philadelphia, PA, USA

Funding Agencies: The study was fully funded by The American Beverage Association.

Disclosure: JCP, JOH received consulting fees from The Coca-Cola Company outside of the submitted work. The remaining co-authors declared no conflict of interest.

Author contributions: JP, HW, GF, ZP, JH, were involved in study design, data analysis and data interpretation; CB, SH, SV, AW were involved in data collection. All authors were involved in writing the manuscript and approved the final submission.

Received: 16 January 2014; **Accepted:** 7 March 2014; **Published online 27 May 2014. doi:10.1002/oby.20737**

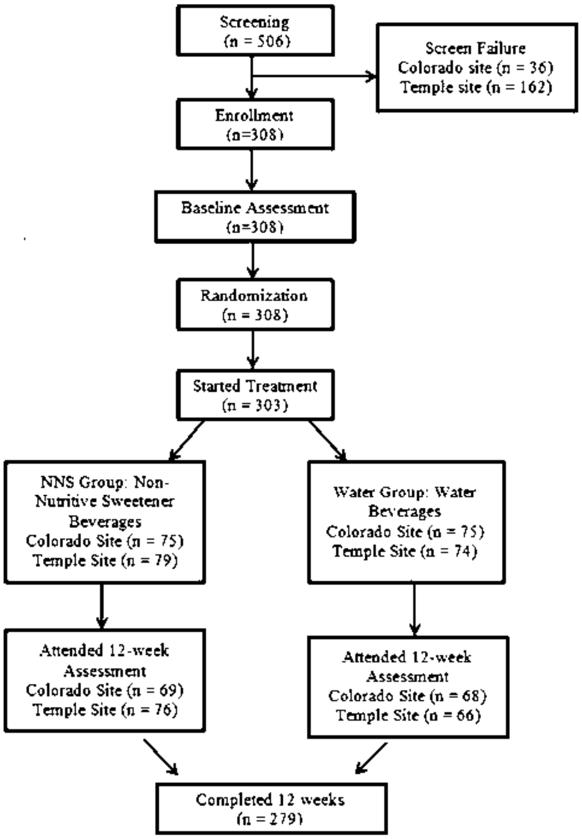

FIGURE 1 Screening, enrollment, randomization, and follow up of study participants.

Given the great interest in losing weight, preventing weight gain and maintaining weight loss (15), it is important to understand whether NNS beverages are a benefit or a hindrance to people actively trying to manage their weight. As water has been deemed the "gold standard" beverage recommended for weight management it is important to assess, in a randomized trial, whether NNS beverages and water produce equivalent weight loss among people enrolled in a behavioral weight management program.

Here, we report findings from the 12-week weight loss phase of a 1-year randomized, clinical trial to test the hypothesis that the amount of weight lost (12 weeks) and maintained (9 months) in a behavioral weight management program will be equivalent in participants consuming beverages containing NNS compared to water.

Methods

Participants

Five hundred and six participants were screened and 308 were enrolled in the trial between October 2012 and April 2013 at University of Colorado (n = 151:4 cohorts) and Temple University (n = 157:5 cohorts), see Figure 1. Participants were male and female, ages 21-65, BMI 27-40. Enrollees represented a range of ethnicities and races (Table 1).

Screening was carried out over the phone or through completion of a secure web based screening form. Eligible participants had to report being weight stable within 10 pounds in the past 6 months, engaging in fewer than 300 min of physical activity per week and consuming at least 3 NNS beverages per week. Applicants were excluded if they were lactating or pregnant within the past 6 months or were planning on becoming pregnant during the time frame of the study. Other exclusions included but were not limited to: diabetes, CVD, uncontrolled hypertension, and use of current medications affecting weight and metabolism. Participants needed physician approval stating they were in good general health and that nutrition and exercise requirements would not be contraindicated.

The study was approved by the Western IRB at the University of Colorado site and the Temple University IRB. Informed consent was obtained from all participants.

Study design

The study was designed as a 1-year equivalence randomized trial composed of a 12-week weight loss phase followed by a 9-month weight maintenance phase. Participants were randomly assigned to the NNS beverage or water treatment arms by a computer-generated randomization schedule that ensured an equal distribution of women and men in each behavioral weight loss treatment group. Participants

TABLE 1 Baseline subject characteristics by group[a]

Characteristic	NNS group (n = 158)	Water group (n = 150)
Age (y)[b]	48.3 ± 10.4	47.3 ± 10.6
Gender [n, (%)]		
Female	130 (82%)	125 (83%)
Male	28 (18%)	25 (17%)
Ethnicity [n, (%)][c]		
Hispanic/Latino	23 (15%)	12 (8%)
Not Hispanic/Latino	133 (85%)	138 (92%)
Race [n, (%)]		
White	107 (68%)	101 (67%)
Black/African American	42 (27%)	43 (29%)
Asian or Pacific Islander	1 (0.6%)	4 (3%)
American Indian or Alaskan Native	1 (0.6%)	0 (0%)
Multiracial Origin	1 (0.6%)	1 (0.6%)
Other	6 (4%)	1 (0.6%)
BMI (kg m^{-2})[d]	33.92 ± 4.25	33.30 ± 3.98
Baseline weight (kg)	93.92 ± 13.29	93.03 ± 12.99
Systolic BP (mm Hg)	118.79 ± 12.08	117.87 ± 12.58
Diastolic BP (mm Hg)	76.70 ± 7.56	76.21 ± 7.29

[a]There were no significant differences between the two groups in demographic profile.
[b]Chi-square analyses completed for Gender, Ethnicity, and Race. Chi-square analysis showed no between group differences.
[c]Two sample t test statistics showed no between group differences. Two sample t test analyses completed for age, weight, systolic BP, diastolic BP, BMI. Mean ± SD (all such values).
[d]n = 156 in NNS group.
[e]n = 156 in NNS group and n = 147 in Water group.

had to be willing to discontinue drinking NNS beverages for the 1-year study should they be randomized to the water treatment group.

The protocol specified preplanned data analyses to be conducted on the primary outcome of weight loss at 12 weeks (weight loss period) and at the end of 1 year (weight loss maintenance).

Intervention

All participants. All participants received a comprehensive cognitive- behavioral weight loss intervention called The Colorado Weigh (16). Participants attended 12 weekly, 60-min group meetings that were led by registered dietitians or clinical psychologists. Participants attended group meetings organized by treatment (NNS or Water) and were instructed on behavioral weight loss strategies. Weekly weigh-ins were conducted at each group meeting. Examples of weekly topics include self-monitoring; portion sizes, reading food labels; physical activity and insights into weight loss maintenance from the National Weight Control Registry (16-19).

Individual energy targets for weight loss were set as equal to each participant's estimated resting metabolic rate (RMR), rounded up to the nearest 100 kcal, determined using a Tanita Model TBF-300A bioelectrical impedance device that assesses body composition and provides an imputed RMR. Energy targets were adjusted, as needed, by the group leader in an attempt to achieve a weight loss of 1 to 2 pounds per week. Weekly physical activity targets were set based on increasing moderate to vigorous activity by 10 min week $^{-1}$ above the subject's usual activity level with a target goal to reach 60 min day $^{-1}$, 6 days week $^{-1}$. Physical activity was assessed by two methods: (1) participants wore a Body Media armband (Manufacturer: BodyMedia, Model AB155) for 1 week during weeks 1 and 12, and (2) participants reported daily physical activity minutes on exercise logs turned in weekly. Participants received the same curriculum regardless of which treatment arm they were assigned to with the only difference being discussion of the type of beverages they were instructed to consume during the study.

NSS beverage group. Participants randomized to the NNS beverage group were asked to consume at least 24 fluid ounces of NNS beverage per day and their water consumption was not restricted. An NNS beverage qualified if it had <5 kcal per 8 ounce-serving, was pre-mixed, and contained non-nutritive sweeteners.

Water group. Participants randomized to the water group were asked to consume at least 24 fluid ounces of water per day, and not drink any NNS beverages. They could, however, eat foods that contained NNS (examples: artificially sweetened yogurt, gum, candies, cookies, ice cream, gelatin, pudding), but could not intentionally add NNS (examples: aspartame—NutraSweet or Equal, sucralose—Splenda, stevia Truvia; as well as diet creamers) to beverages such as coffee.

Participants were given manufacturers coupons weekly (from the three largest beverage manufacturers: The Coca-Cola Company, PepsiCo and Dr Pepper Snapple Group), redeemable for bottled water or NNS beverages at retail stores. Participants were asked to record their beverage intake daily, and this information was used to assess treatment adherence.

Measurements

All assessments, except for height, were conducted at baseline and after 12 weeks of treatment. Height without shoes was measured to the nearest 0.1 cm at the screening visit using a wall-mounted stadiometer. Body weight in light clothing and without shoes was measured to the nearest 0.1 kg on a digital scale. Waist circumference was measured at the top of the iliac crest until two consecutive measures within 0.5 cm were obtained. Resting blood pressure was measured while the subjects were seated after a 5-min rest; the average of two measures was used. Blood samples were collected using standard venipuncture method after a 10- to 12-h fast for measurement of lipids and glucose. Participants provided a urine sample collected in a sterile container for measurement of urine osmolality. Blood samples from both study sites were analyzed at the University of Colorado Hospital laboratory. Urine samples collected at the Colorado site were measured at the University of Colorado Hospital laboratory; those from Temple University were measured at Quest Diagnostics, Madison, NJ.

Participants completed questionnaires at baseline and 12 weeks to assess changes in perceived hunger (using a 100 mm visual analog scale anchored at "not at all hungry" and "extremely hungry"). Beverage treatment adherence was determined from daily beverage logs, collected weekly, on which participants recorded all beverages consumed.

Participants received $75 for completing the assessment visit at 12 weeks and $50 for completing at least 9 of 11 food and beverage logs during the 12-week weight loss intervention. Total compensation if they completed all requirements was $125.

Power of the study

The primary outcome addressed in this report is change in body weight during the 12-week weight loss phase of this 1 year trial. The study was designed as an equivalence trial with the hypothesis that there would be no clinically meaningful difference in weight change between those consuming NNS beverages or water. Specifically, the bounds of equivalence for between-group difference in 12-week weight loss were prespecified to ± 1.7 kg. Assuming the true difference was 0.57 (1/3 of the equivalence margin) and common SD of 3.9 kg, a sample size of 150 per arm was required using two, one-sided t tests to ensure at least 80% power with an alpha level of $P < 0.05$ to establish equivalence.

Statistical analysis

Intent-to-treat (baseline observation carried forward) was used as the primary analysis for efficacy of weight loss using the weekly body weights as the dependent variable. As a secondary analysis we also looked at only participants who completed all 12 weeks of the trial. Five participants were randomized but did not begin treatment (Figure 1) and were excluded. The primary outcome measure was change in body weight from baseline to 12 weeks. The results were the same using baseline carried forward or a mixed model (accounting for missing data) analytic schemes.

The primary hypothesis tested in this study was that water and NNS beverage treatments would be equivalent with upper and lower bounds of equivalence set at ±1.7 kg. This body weight difference was chosen as a value that would not be meaningfully different in a

clinical setting. To be considered equivalent, the mean and the upper and lower 90% confidence limits for the difference in weight loss between NNS beverage and Water groups would have to be within the pre-set upper and lower bounds of equivalence, ±1.7 kg. Other weight-related outcomes included weight change from baseline for participants who completed all 12 weeks of the trial (for whom we had a 12-week body weight) and percentage of participants who lost at least 5% of their initial weight. Differences between treatment groups for weight loss were assessed using several different methods: a mixed model, ANCOVA and two independent *t* tests (or chi-square when appropriate). All methods showed the same results. We report here the *t* test results [two one-sided *t* tests; the standard approach for evaluating equivalence (20)] and 90% confidence intervals. Secondary outcomes (waist circumference, systolic blood pressure, blood measures, urine osmolality, hunger, and physical activity) were analyzed using linear mixed effects model which consisted of classification variables of time (baseline or 12 weeks) and group (NNS or water) as well as their interaction term as fixed effects and an unstructured covariance. Between-group and within-group contrasts were tested under this model.

Results

A total of 303 participants began the study treatment and 279 participants completed the 12-week weight loss phase of the study, representing 92% of the starting population (Figure 1). Study dropouts were similar across the two study sites (9.27% at Colorado, 9.55% at Temple) as well as between the treatment groups (5.8% for NNS, 10% for water). There were no significant baseline differences in age, gender, race/ethnicity or other study measures between the water and NNS beverage treatment groups (Table 1). Almost 80% of the participants were female, 68% white, and 27% African American.

There were no significant differences between groups in adherence to the study beverages as assessed by the weekly beverage consumption logs. Percent adherence for reported daily consumption of at least 24 ounces of NNS or water was 96.6% vs. 95.7%, respectively ($P = 0.34$). Weekly group meeting attendance was also not different between the groups (attendance: 90.8% for NNS; 89.7% for Water, $P = 0.24$).

The mean weight loss difference between Water and NNS was −1.85 kg (90% CI: −1.12 kg, −2.58 kg). Because the lower confidence limit (−2.58 kg) was outside of the equivalence bounds set *a priori* in our hypotheses, the two treatments were not equivalent and paired comparisons were conducted. This analysis indicated that weight loss in the NNS beverage group [5.95 kg ± 3.94 kg (SD)] was significantly greater than the Water group (4.09 kg ± 3.74 kg (SD), $P < 0.0001$) using an intent to treat (LOCF) analytic scheme (Table 2). Similar findings were observed using observations only from those completing the 12-week assessment (Table 3). In the Water group, 43.0% of participants lost >5% of their body weight, while 64.3% of participants in the NNS beverage group lost > 5% ($P = 0.0002$; Figure 2).

After 12 weeks of treatment, changes in waist circumference, glucose, systolic blood pressure, HDL, triglycerides and urine osmolality were not significantly different between treatment groups. Reductions in total cholesterol and LDL were significantly greater in the NNS group than in water group (Table 4).

There was no significant difference between groups in change in physical activity over 12 weeks as determined by either armband or activity log measures. Hunger increased slightly in the Water group while it declined slightly in the NNS group, resulting in a significant between group difference ($P = 0.013$, Table 4).

Discussion

In this 12-week weight loss study, consuming water and NNS beverages were not equivalent for weight loss, with the NNS group losing significantly more weight than the water group. The results provide support for the use of NNS beverages in the context of a behavioral weight management program and should be reassuring for people who choose to consume NNS beverages. It demonstrates that they can drink a NNS beverage without the caloric contribution of nutritive sweeteners and without concern that their weight loss efforts will be undermined and, in fact, may be slightly enhanced. It should be noted that because eligible subjects were already NNS drinkers assignment to the NNS treatment did not require as great a behavior change as the Water group who had to abstain from NNS beverages for the trial. We chose this design rather than admitting all comers in order to ensure that subjects assigned to NNS would adhere to the treatment giving us the ability to see if NNS adversely affected weight loss. Despite this, subject completion was high and did not differ between groups and adherence to the treatment was >95% based on beverage logs.

These findings build on the only other published study similar to the present trial. Tate et al. (14) compared water, diet beverages and an attention control over 6 months of intentional weight loss using a superiority trial design in 318 participants. Those authors found no significant differences in mean weight loss between the water and diet beverage groups when compared to the attention control group. However, the probability of losing 5% of body weight was significantly better in the NNS group compared to the attention control group ($P = 0.04$). The likelihood of achieving a 5% loss was not different between the group assigned to water compared to the attention control ($P = 0.13$). A significant difference between that study and the present trial is the intensity of the intervention. Tate et al. asked participants to make a single substitution in their diet, changing beverage options, while in our study participants in both the water and NNS groups received a comprehensive behavioral treatment program. Participants in the Tate trial lost <2% of body weight on average over the first 12 weeks while participants in the present trial lost between 4 and 7% of body weight. Taken together, results from both studies suggest that NNS beverages can be an effective strategy for weight management both in low intensity and high intensity behavioral interventions. Furthermore, in the present study NNS beverages performed better than water in supporting weight loss during the 12-week weight loss phase.

The purpose of this trial was to compare directly the "gold standard" beverage for supporting good health, water, with NNS beverages in the context of weight loss. This is an important question as many people choose to consume NNS beverages as part of a weight management strategy, and others may be more likely to do so if they

TABLE 2 Baseline-carried-forward analysis for absolute weight loss (kg)

Group	Baseline weight (kg)	Week 12 clinic weight (kg)	Change	90% CL mean change	*P* value for change
NNS (*n* = 154)	93.91 (13.46)	87.97 (13.39)	−5.95 (3.94)*	−5.42, −6.47	**<0.0001**
Water (*n* = 149)	93.15 (12.94)	89.06 (12.86)	−4.09 (3.74)*	−3.59, −4.60	**<0.0001**
NNS—water	0.76 (13.21)	−1.09 (13.13)	−1.85 (3.84)*	−1.12, −2.58	**<0.0001**

Analysis includes those participants who dropped out of the study in the analysis, using the baseline observation carried forward. This analysis mimics the clinical setting. Although equivalence cannot be established, participants lost more weight in the NNS group as compared to the water group. All analyses were completed using a Satterthwaite two sample *t* test. All values are Mean (SD) unless otherwise noted. Statistically significant values ($P < 0.05$) are shown by an asterisk (*) and statistically significant *P* values are shown in bold.

had confidence that it would not hinder their success. The popular media continues to raise questions about the value of NNS beverages in weight loss (21,22) citing concerns from some experts that NNS beverage usage is associated with obesity and weight gain in observational studies (21-23). The current results, along with results of Tate et al. (14), provide strong evidence from large randomized controlled trials that NNS beverages do not hinder and can help with weight loss when compared to water. In addition, Phelan and Wing examined the use of NNS beverages by those in the National Weight Control Registry and found that successful weight losers drank three times the NNS beverages compared to those who had never lost weight (24).

We chose 12 weeks as the weight loss phase because most studies show that weight loss slows considerably after 6 months of treatment with more than half of the weight loss occurring in the first 12 weeks (25,26), probably owing to difficulty with longer term adherence to a hypocaloric regimen. Furthermore, it is now recognized that weight loss is a different process from weight maintenance, both behaviorally and physiologically, so it is important to study treatment effects on these two processes separately (27). The benefit of the current 1 year trial is that we will be able to compare both weight loss and weight loss maintenance within the same group of participants. The trial was designed to allow preplanned analysis of the treatment effects after just the 12-week weight loss phase as well as after 9 months of weight maintenance (still underway) which will be reported separately.

While most secondary outcomes were not different between the groups, the NNS group showed greater reductions in total- and LDL-cholesterol. This may be due to the greater weight loss in the NSS group. There was also no significant difference between the groups in urine osmolality although osmolality decreased slightly in the water and increased slightly in the NNS group. It is unlikely that changes in hydration status were responsible for the significant differences in body weight between treatments. The small changes observed were well within the normal range for urine osmolality (500-800 mOsmol kg^{-1}) suggesting no adverse effect on fluid intake regulation. Physical activity increased significantly in both groups as a function of the behavioral treatment but was not significantly different between groups. Sedentary behavior actually decreased significantly in the Water group over time but not the NNS group. The changes over time were not significant between groups. Taken together, changes in physical activity and sedentary behaviors cannot account for the difference in weight loss observed.

Based on the design of this study we are unable to say, what is the mechanism for the greater weight loss in the NNS group compared to the water group. Weekly hunger scores were significantly lower among the NNS group than the water group although the absolute changes were small. While it is plausible that the NNS participants were more likely to adhere to the dietary recommendations due to less hunger than the Water group we cannot conclude this based on this study. Some authors (3,5,6) have suggested that use of NNS may increase appetite for sweet foods and disrupt regulation of energy balance. Weight loss results for the present study suggest that NNS consumption did not increase energy intake from other foods compared to water. This is consistent with other studies that have not found increased consumption of sweet or high energy foods while using NNS (28,29). Further studies will be needed to ascertain the mechanism(s) that may be responsible for the weight loss results.

TABLE 3 Absolute weight loss (kg) for completers

Group	Baseline weight (kg)	Week 12 clinic weight (kg)	Change	90% CL mean for change	*P* value for change
NNS (*n* = 142)	93.56 (13.23)	87.11 (12.85)	−6.45 (3.68)*	−5.94, −6.96	**<0.0001**
Water (*n* = 134)	93.88 (12.99)	89.33 (13.07)	−4.55 (3.67)*	−4.03, −5.08	**<0.0001**
NNS—water	−0.32 (13.12)	−2.22 (12.96)	−1.90 (3.67)*	−1.16, −2.63	**<0.0001**

Analysis including participants who completed 12 weeks of the trial. Although equivalence cannot be established participants lost more weight in the NNS group as compared to the water group. All analyses were completed using a Satterthwaite two sample *t* test. All values are Mean (SD) unless otherwise noted. Statistically significant values ($P < 0.05$) are shown by an asterisk (*) and statistically significant *P* values are shown in bold.

TABLE 4 Cardiometabolic, hunger, physical activity, and sedentary activity changes from baseline to week 12 in the NNS and water groups[a]

Outcome variable and group	Assessment period[b]		Change	P value for change
	Baseline	Week 12		
Waist circumference (cm)				
NNS	108.00 (0.86)	102.27 (0.88)	5.73 (0.49)*	**<0.0001**
Water	107.10 (0.87)	102.74 (0.90)	−4.36 (0.50)*	**<0.0001**
NNS—water	0.90 (1.22)	−0.46 (1.26)	−1.36 (0.70)	0.0528
Systolic BP (mm Hg)				
NNS	118.84 (0.99)	112.60 (1.07)	−6.25 (0.95)	**<0.0001**
Water	117.93 (1.01)	113.71 (1.10)	−4.23 (0.97)*	**<0.0001**
NNS—water	0.91 (1.42)	1.11 (1.54)	2.02 (1.36)	0.1372
Glucose (mg dl ^{1})				
NNS	91.44 (1.45)	93.59 (0.95)	2.15 (1.03)*	**0.0375**
Water	90.92 (1.47)	93.40 (0.98)	2.48 (1.05)*	**0.0193**
NNS—water	0.52 (2.07)	0.19 (1.37)	−0.33 (1.47)	0.8224
Cholesterol (mg dl ^{1})				
NNS	190.68 (2.76)	173.92 (2.70)	16.76 (1.99)*	**<0.0001**
Water	193.23 (2.80)	184.38 (2.77)	−8.86 (2.05)*	**<0.0001**
NNS—water	−2.56 (3.93)	−10.46 (3.87)*	−7.90 (2.86)*	**0.0061**
HDL (mg dl ^{1})				
NNS	53.67 (1.65)	50.55 (1.17)	−3.12 (1.36)*	**0.0224**
Water	55.91 (1.68)	52.00 (1.20)	−3.91 (1.39)*	**0.0053**
NNS—water	2.24 (2.36)	1.45 (1.68)	0.79 (1.94)	0.6831
LDL (mg dl ^{1})				
NNS	114.92 (2.42)	103.39 (2.34)	−11.53 (1.72)*	< **0.0001**
Water	116.44 (2.45)	110.77 (2.39)	5.68 (1.77)*	**0.0015**
NNS—water	−1.52 (3.44)	−7.38 (3.34)*	−5.86 (2.47)*	**0.0184**
Triglycerides (mg dl ^{1})				
NNS	120.71 (6.35)	104.16 (6.43)	−16.56 (5.12)*	**0.0014**
Water	119.20 (6.46)	109.30 (6.59)	−9.90 (5.28)	0.0617
NNS—water	1.51 (9.06)	−5.14 (9.21)	−6.65 (7.35)	0.3662
Urine osmolality (mOsmol kg ^{1})				
NNS	567.36 (21.35)	597.67 (22.88)	30.31 (25.44)	0.2346
Water	592.54 (21.71)	565.79 (23.53)	−26.75 (26.10)	0.3063
NNS—water	−25.18 (30.45)	31.88 (32.82)	57.06 (36.45)	0.1186
How hungry did you feel over the past week (scale 1–100)?				
NNS	51.91 (1.57)	48.42 (1.47)	−3.49 (1.92)	0.0694
Water	47.93 (1.62)	51.34 (1.53)	3.41 (1.99)	0.0877
NNS—water	3.98 (2.25)	−2.92 (2.12)	−6.90 (2.76)*	**0.0130**
Total moderate PA (hrs/week)				
NNS	4.30 (0.23)	6.09 (0.26)	1.79 (0.25)*	**<0.0001**
Water	4.40 (0.24)	5.49 (0.27)	1.10 (0.26)*	**<0.0001**
NNS—water	−0.10 (0.33)	0.59 (0.37)	0.69 (0.36)	0.0547
Total sedentary activity (hrs/week)				
NNS	158.06 (1.92)	156.39 (2.27)	−1.67 (2.55)	0.5129
Water	160.78 (1.96)	155.03 (2.34)	−5.75 (2.63)*	**0.0305**
NNS—Water	−2.71 (2.74)	1.36 (3.26)	4.07 (3.67)	0.2685

[a]All analyses are from mixed effect models. Statistically significant values (P < 0.05) are shown by an asterisk (*) and statistically significant P values are shown in bold. NNS, Non-nutritive sweetener group; water, water group; BP, blood pressure; PA, physical activity. For systolic BP and waist circumference: n = 142 for NNS and n = 134 for water. For glucose, cholesterol, HDL, and triglycerides: n = 142 for NNS and n = 133 for water. For LDL: n = 140 for NNS and 131 for water. For urine osmolality: n = 141 for NNS and n = 133 for water. For "How hungry did you feel over the past week": n = 132 for NNS and n = 122 for water. For total moderate PA: n = 136 for NNS and n = 126 for water. For total sedentary activity: n = 136 for NNS and n = 126 for water.
[b]All values are means; Standard Error in parentheses.

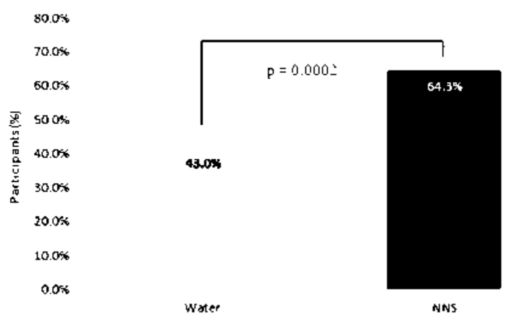

FIGURE 2 Percent participants who achieved at least 5% weight loss. Results based on Chi-square analysis. Analysis includes those participants who dropped out of the study in the analysis, using the baseline observation carried forward. This analysis mimics the clinical setting. Difference 0.2133 or 21.33% difference between groups with 95% CI (0.1212 0.3054). n = 154 for NNS, n = 149 for Water

These results strongly suggest that NNS beverages can be part of an effective weight loss strategy and individuals who desire to consume them should not be discouraged from doing so because of concerns that they will undermine short-term weight loss efforts. A longer term follow-up of this randomized cohort, now underway, will clarify the utility of NNS beverages in weight loss maintenance. O

Acknowledgments

Additional thanks: University of Colorado study team: Kristen Bing, Gina Malloy-Claxton, Kristen Frie, Danielle Ostendorf, and Rebecca Stark. Temple University study team: Brooke Bailer, Kaitlyn Beauregard, Raymond Carvajal, Pamela Davis, Hannah Lawman, Michelle Lent, Darnice Martinez, Heather Polonsky and Colleen Tewksbury, Biofortis Clinical Research, Inc., for providing CRO support. This trial was registered at www.clinicaltrials.gov, NCT01766700

© 2014 The Obesity Society

References

1. Popkin BM, Armstrong, LE, Bray GM, Caballero B, Frei B, Willett WC. A new proposed guidance system for beverage consumption in the United States. *Am J Clin Nutr* 2006;83:529-542.
2. US Department of Agriculture and Health and Human Services. *Dietary Guidelines for Americans*, 7th ed. Washington, DC: US Government Printing Office; 2006, p 19.
3. Davidson TL, Martin AA, Clark K, Swithers S. Intake of high-intensity sweeteners alters the ability of sweet taste to signal caloric consequences: implications for the learned control of energy and body weight regulation. *Q J Exp Psychol* 2011;64/7:1430-1441.
4. Fowler SP, Williams K, Resendez RG, Hunt KJ, Hazuda HP, Stern MP. Fueling the obesity epidemic?. Artificially sweetened beverage use and long-term weight gain *Obesity* 2008;16:1894 1900.
5. Nettleton JA, Lutsey PL, Wang Y, Lima JA, Michos ED, Jacobs DR. Diet soda intake and risk of incident metabolic syndrome and type 2 diabetes in the multi-ethnic study of atherosclerosis (MESA). *Diabetes Care* 2009;32:688 694.
6. Swithers SE, Martin AA, Davidson TL. High-intensity sweeteners and energy balance. *Physiol Behav* 2010;100/1:55 62.
7. Blackburn GL, Kanders BS, Lavin PT, Keller SD, Whatley J. The effect of aspartame as part of a multidisciplinary weight-control program on short- and long-term control of body weight. *Am J Clin Nutr* 1997;65:409 418.
8. Raben A, Vasilaras TH, Moller AC, Astrup A. Sucrose compared with artificial sweeteners: different effects on ad libitum food intake and body weight after 10 wk of supplementation in overweight subjects. *Am J Clin Nutr* 2002;76:721-729.
9. Bellisle F, Drewnowski A. Intense sweeteners, energy intake and the control of body weight. *Eur J Clin Nutr* 2007;61:691-700.
10. Mattes RD, Popkin BM. Non-nutritive sweetener consumption in humans, effects on appetite and food intake and their putative mechanisms. *Am J Clin Nutr* 2009;89:1 14.
11. Rolls BJ. Effects of intense sweeteners on hunger, food intake, and body weight: a review. *Am J Clin Nutr* 1991;53:872-878.
12. Stellman SD, Garfinkel L. Artificial sweetener use and one-year weight change among women. *Prev Med* 1986;15:195 202.
13. Pereira MA. Diet beverages and the risk of obesity, diabetes, and cardiovascular disease: a review of the evidence. *Nutr Rev* 2013;71:433-440.
14. Tate DF, Turner-McGrievy G, Lyons E, et al. Replacing caloric beverages with water or diet beverages for weight loss in adults: main results of the choose health options consciously everyday (CHOICE) randomized clinical trial. *Am J Clin Nutr* 2012;95:555-563.
15. Americans Concerned About Their Weight: The International Food Information Council Foundation 2010 Food & Health Survey Looks at What Motivates Americans (2010, August). *Food Insight 3*, available at http://www.foodinsight.org/Newsletter/Detail.aspx?topic=Americans Concerned About Their Weight; accessed 12/7/13.
16. Wyatt HR, Jortberg, BT, Doing F, et al. Weight loss in a community initiative that promotes decreased energy intake and increased dairy consumption: Calcium weighs ins. *J Phys Activity Health* 2008;5:28 44.
17. Klem ML, Wing RR, McGuire MT, Seagle HM, Hill JO. A descriptive study of individuals successful at long-term maintenance of substantial weight loss. *Am J Clin Nutr* 1997;66:239 246.
18. Wyatt HR, Phelan S, Wing RR, Hill JO. Lessons from patients who have successfully maintained weight loss. *Obes Manage* 2005;1:56-61.
19. Wing RR, Hill JO. Successful weight loss maintenance. *Annu Rev Nutr* 2001;21:323 341.
20. Walker E, Novacki AS. Understanding equivalence and noninferiority testing. J Gen Intern Med 2010;26:192-196.
21. Mosbergen D. Diet soda health risks: Study says artificial sweeteners may cause weight gain, deadly diseases, 2013. Available at: http://www.huffingtonpost.com/2013/07/11/diet-soda-health-risks_n_3581812.html; accessed 12/7/13.
22. Klatell J. Can diet soda make you gain weight? 2007. Available at: http://www.cbsnews.com/news/can-diet-soda-make-you-gain-weight/; accessed 12/7/13.
23. Neubert AP. Prof: Diet drinks are not the sweet solution to fight obesity, health problems. 2013. Available at: http://www.purdue.edu/newsroom/releases/2013/Q3/prof-diet-drinks-are-not-the-sweet-solution-to-fight-obesity,-health-problems.html; accessed 12/7/13.
24. Phelan S, Lang W, Jordan D, Wing RR. Use of artificial sweeteners and fat modified foods in weight loss maintainers and always-normal weight individuals. *Int J Obes* 2009;33:1183-1190.
25. Foster GD, Wyatt HR, Hill JO, et al. Weight and metabolic outcomes after 2 years on a low-carbohydrate versus low-fat diet: a randomized trial. *Ann Intern Med* 2010;153:147-157.
26. US Department of Health and Human Services, Public Health Service, National Institutes of Health, National Heart, Lung, and Blood Institute. Publication Number 00-4084. The Practical Guide: Identification, Evaluation and Treatment of Overweight and Obesity in Adults; 2000, p 24.
27. Seagle HM, Wyatt HR, Hill JO. Obesity: Overview of treatments and interventions. In: Coulston AB, Coushey CJ, editors. Nutrition in the Prevention and Treatment of Disease. Burlington, MA: Elsevier; 2008, Chapter 22, p 379-388.
28. Binkley JG, Golub A. Comparison of grocery purchase patterns of diet soda buyers to those of regular soda buyers. *Appetite* 2007;49:561 571.
29. Piernas C, Tate DF, Wang X, Popkin BM. Does diet-beverage intake affect dietary consumption patterns?. Results from the choose healthy options consciously everyday (CHOICE) randomized trial. *Am J Clin Nutr* 2013;97:604 611.

Evidence-based mapping of design heterogeneity prior to meta-analysis: a systematic review and research synthesis

March 28, 2014

1. Michelle D. Althuis, PhD – corresponding author
EpiContext
1115 East Capitol Street SE
Washington, DC 20003
Michelle@EpiContext.com

2. Douglas L. Weed, MD, MPH, PhD
DLW Consulting Services, LLC
1302 N. Oak Forest Rd.
Salt Lake City, UT 84103
Douglaslweed@aol.com

3. Cara L. Frankenfeld, PhD
Department of Global and Community Health
George Mason University
4400 University Drive, MS 5B7
Fairfax, VA 22030
cfranken@gmu.edu

ABSTRACT

Introduction. Assessment of design heterogeneity conducted prior to meta-analysis is infrequently reported; it is often presented post hoc to explain statistical heterogeneity. However, design heterogeneity determines the mix of included studies and how they are analyzed in a meta-analysis, which in turn can importantly influence the results. The goal of this work is to introduce ways to improve the assessment and reporting of design heterogeneity prior to statistical summarization of epidemiologic studies.

Methods. In this paper we show how a technique called "evidence mapping" can be used to organize studies and evaluate design heterogeneity prior to meta-analysis, using assessment of sugar-sweetened beverages (SSB) and type 2 diabetes (T2D) as an example. Employing a systematic and reproducible approach, we evaluated across 11 selected cohort studies: variation in definitions of SSB, T2D, and co-variables, design features and population characteristics associated with specific definitions of SSB, and diversity in modeling strategies.

Results. Evidence mapping strategies effectively organized complex data and clearly depicted design heterogeneity. For example, across 11 studies of SSB and T2D, 7 measured diet only once (with 7 to 16 years of disease follow-up), 5 included primarily low SSB consumers, and 3 defined the study variable (SSB) as consumption of either sugar or artificially-sweetened beverages. This exercise also identified diversity in analysis strategies, such as adjustment for 9-17 co-variables and a large degree of fluctuation in SSB-T2D risk estimates depending on variables selected for multivariable models (2-95% change in the risk estimate from the age-adjusted model).

Conclusions. Meta-analysis seeks to understand heterogeneity in addition to computing a summary risk estimate. This strategy effectively documents design heterogeneity, thus improving the practice of meta-analysis by aiding in: 1. protocol planning; 2. transparent reporting of differences in study designs; and 3. interpretation of pooled estimates. We recommend expanding the practice of meta-analysis reporting to include a table that summarizes design heterogeneity. This would provide readers with more evidence to interpret the summary risk estimates.

Keywords

Heterogeneity, evidence map, systematic review, meta-analysis, sugar-sweetened beverages, type 2 diabetes

Background

Meta-analyses, which are a quantitative method for pooling results from epidemiologic studies, inform research priorities and health policy. Combining similar studies asking a similar research question is fundamental to the interpretability of summary risk estimates [1]. Combining results in a meta-analysis from studies that are designed to answer different scientific questions may lead to imprecise and possibly invalid inferences [2, 3].

An assessment of the similarity of studies (i.e. design heterogeneity) is a fundamental element of a meta-analysis of epidemiological studies [3-8]. There are two major types of heterogeneity: statistical heterogeneity and design heterogeneity (sometimes referred to as clinical and methodological diversity) [9]. Statistical heterogeneity is purely a mathematical assessment; evidence of statistical heterogeneity indicates that there is greater statistical variance between the study results than would be expected by chance if the effect size was similar across studies [8,10]. Design heterogeneity, in contrast, involves the extent to which the studies being considered for inclusion in a meta-analysis differ in study design, including population studied, specificity of exposure measurement, uniformity of diagnostic criteria (in the outcome), confounders measured, concomitant exposures measured, and statistical models [3,7].

Reviews of the practice of meta-analysis in observational epidemiology have observed that investigators often emphasize the summarization function over the assessment of heterogeneity [2, 11]. Additionally in a systematic overview of meta-analyses, we found less than a third of 47 eligible meta-analyses of lifestyle and dietary risk factors for type 2 diabetes (T2D) reported a detailed characterization of design heterogeneity that was used to guide the quantitative pooling of study results (manuscript in preparation). In contrast, more than 90% of the meta-analyses reported some assessment of statistical heterogeneity (Q statistic or I^2 index). These observations illustrate that the assessment of design heterogeneity frequently occurs after statistical heterogeneity has been identified. In practice, design heterogeneity assessment would be informative if undertaken before any quantitative summarization takes place [2].

In 2013, the journal Research Synthesis Methods devoted an entire issue to describing the challenges associated with meta-analysis of observational studies [12], calling for more strategies to aid conduct and reporting [13]. In this paper, we present a strategy for objectively and transparently characterizing design heterogeneity of epidemiologic studies prior to meta-analysis.

METHODS

Evidence-based mapping was used as a tool to diagram and tabulate data across relevant studies, with the following three primary objectives:

1. To compare definitions for the study variable, outcome, and co-variables;
2. To assess the design features and population characteristics associated with specific definitions of the study variable; and
3. To evaluate the diversity in modeling strategies and suggest simple summary measures to benchmark susceptibility of the exposure risk estimate to the influences of included (and excluded) co-variables in multivariable regression models.

We sought to summarize the detailed work of multiple evidence maps created to meet these objectives, into a single table with a universal adaptable format. The aim of this table is to facilitate the reporting of design heterogeneity, which is fundamental to developing a protocol, analyzing data, and interpreting meta-analyses.

Tools

Evidence maps are used to transparently generate a clear visual depiction of complicated data, either in the form of a diagram or a table [14]. Evidence maps have been used to set research priorities by displaying existing research landscapes without linking study designs to study results [14-23]. Precisely because evidence mapping seeks to organize studies without summarizing results, they are natural tools for assessing design heterogeneity prior to meta-analysis. Therefore, we expanded evidence mapping methods by demonstrating their usefulness in planning a meta-analysis. This work is guided by previously published evidence maps whose focus was research priority setting [14-23] and the existing standards for conducting and reporting of systematic reviews of observational research [4,24]. Evidence maps were created in Excel (Microsoft Excel. Redmond, Washington: Microsoft, 2007); however, it is possible to conduct the work using other database software.

Definitions

In this paper, design heterogeneity refers to differences across studies in socio-demographic and health characteristics of the populations studied, methods of study execution and data ascertainment, and statistical approaches as well as analyses conducted and reported.

In addition to traditional confounding variables such as age or health status, the definition of co-variables when summarizing findings across multiple studies includes design features.

Evidence-based mapping framework: steps for evaluating design heterogeneity

Prior to applying this three-step framework, we recommend use of a PICO (Participants, exposure/Intervention, Comparator, and Outcome) table to identify key research components and to develop/clarify the research question [25], which is then followed by a systematic search for

eligible studies [4]. In order to present the evidence-based mapping framework in a way that other investigators can easily translate to their own research questions, the next section describes the each step generally. The details of the application of the framework to a specific example are described in the subsequent section, including the systematic search process.

In order to thoroughly use this framework, we recommend at least three investigators: an author to abstract data at the onset and another to verify accuracy; and a consensus of experts to review completed maps and evaluate aspects of design heterogeneity that may importantly influence meta-analysis of the selected studies.

Step 1. To compare the variation in definitions for the study variable, outcome, and co-variables

The goal of the first step of this framework was to understand the range of definitions for the exposure variable across all included studies. For every study, the definition used for exposure was abstracted, including information on the measurement tools, timing of variable collection, and method/criteria (self-report, interviewer administered, medical or biochemical test). When possible, the exact language used to ascertain exposure status or details of test preformed was recorded.

A diagram (evidence map) was created to describe how definitions of the same variable related to one another, quantitatively and qualitatively. The description of the variable definition, using in as much as possible the original language from the publication, was summarized in a text box. Text boxes were organized to group together exposure variables with similar definitions. Like definitions were physically grouped together in the diagram and the review investigator assigned descriptive 'category headings' accordingly. The category of the exposure variable most frequently employed across studies was placed at the top of the diagram, with other categories arranged in order of decreasing frequency. An evaluation of whether the most frequently used definition was indeed the most appropriate definition was not undertaken at this point in the review process. The step should be examined later along with study quality and risk of bias.

The resulting map visually depicted patterns within the exposure definitions and was used to preliminarily evaluate whether the collective group of studies directly addressed the review question or addressed more than one distinct question. It also facilitated an initial assessment of frequently occurring sub-groups of the exposure variable, which could be considered for stratified or sensitivity analyses in a meta-analysis.

Step 1 was repeated for the outcome variable (with particular attention to diagnostic method/criteria) and as needed for complicated co-variables. Co-variables included study location/ethnicity, gender, study size, study duration, timing of participant assessments, and baseline population characteristics such as age, body size, or health status. Univariate statistics (n, median, proportion, range) were used to describe the diversity of variable definitions (exposure, outcome, and co-variables) across included studies.

Step 2. To assess the design features and population characteristics associated with specific definitions of the study variable

The second step was to employ evidence mapping strategies to assess whether specific definitions of the exposure variable tended to aggregate with specific study design features or population characteristics. Evidence maps were used to organize categories of the exposure variable identified in Step 1 in relation to study design features and population characteristics. In separate diagrams for each category of the exposure variable, important design features and population characteristics were listed for each study.

Diagrams for each category of the exposure definition were qualitatively inspected and compared to evaluate emerging patterns. Particular attention was focused on differences between categories of the exposure variable that were identified in Step 1 as potentially not directly answering the review question. Likewise, among exposure categories from studies directly answering the review question, the aggregation of study design/population characteristics was used to augment decisions from Step 1 about stratified/sensitivity analyses in a future review/meta-analysis.

Step 3. To evaluate the diversity in multivariable modeling strategies

The aim of step 3 was to evaluate co-variables selected for models by primary studies and to facilitate the selection of an adequately adjusted model(s) for combining by meta-analysis. Evidence-based mapping was used to visually display the patterns of co-variables adjusted for in each model as reported by each publication. For each study, the table summarized how the exposure variable was analyzed (e.g., continuous measure or categories); and tallied the number of models from each publication and the number of covariates adjusted for in each model.

Every regression model was listed in sequential order as presented in the original research publication and a checklist format was used to summarize co-variables adjusted for in each model. Co-variables most frequently adjusted for in multivariable models across all studies were listed in the map header. A check denoted inclusion of a co-variable in the model and a superscript was added to indicate the timing of the measurement of the co-variable (e.g. BL for baseline). Less frequently adjusted co-variables were listed in a single column of the table.

For each multivariable model, the percent change in the exposure-outcome risk estimate from the age-adjusted model was calculated using the following equation: (age-adjusted relative risk - multivariable adjusted relative risk)/(age-adjusted relative risk   1). This provided a quantitative assessment of the degree of fluctuation in the exposure-response risk estimate from the age-adjusted value, depending on the co-variables included in a model.

Frequency (n), median, proportion, and range were used to describe across included studies the diversity in definitions of the study variable used for analysis (e.g. how categories of exposure were defined), number of multivariable models presented, number of co-variables adjusted for in

multivariable models, and change in exposure-outcome risk estimate from the age-adjusted risk estimate.

Summarization of evidence-based mapping efforts

A single table was designed to capture important findings of the detailed mapping efforts and bridge the more practical need to concisely document and report design heterogeneity. We adopted a universal adaptable format that would be flexible for summarizing the large amounts of complex data organized by evidence maps. Using cohorts as the unit of analysis, for each major category of exposure (as determined by Step 1) the following was summarized: the distribution of important design and population characteristics (as determined in Step 2: n, percent), operationalization of the study variable in the multivariable model (Step 3: n, percent), the number of multivariable models presented in the original publication (Step 3: median, range), the number of co-variables in multivariable models (Step 3: median, range), and the change in exposure-outcome risk estimate from the age-adjusted risk estimate (Step 3: median, range).

Illustration of method using prospective observational studies of SSB and T2D

We illustrate the utility of an evidenced-based mapping framework using an example from nutritional epidemiology: sugar-sweetened beverages (SSB) and type 2 diabetes mellitus (T2D). This example is ideal for illustrating this framework, because studies of this relationship characteristically have considerable variability in study design.

Selection criteria. Firstly, we identified published work for the example. We used an electronic search strategy to identify all cohort studies of dietary sugar intake and T2D. Published research that met the following inclusion criteria were identified for full text review: 1) a prospective observational study (i.e., dietary sugar consumption was measured in chronologic time prior to measurement of T2D); and 2) a study analyzing the risk of T2D associated with dietary sugar intake, dietary patterns, or glycemic load/index. To address the possibility that electronic search strategies might omit publications of findings not important enough (e.g. null findings) for inclusion in the title, keywords, or abstract, our search ascertained published research on dietary patterns as well as dietary sugar intake. Additionally, we identified reviews and meta-analyses of epidemiologic studies on this topic in order to examine their reference lists.

Systematic search. We conducted database searches of PubMed and Scopus (inception to March 10, 2014). We limited our search of the PubMed database to human studies and English language publications, and used the following combination of search terms and medical subject headings (inception to September 19, 2013: 2005 titles): sweetening agents, energy intake, calories, caloric intake, fructose, glucose, sucrose, monosaccharides, disaccharides, dietary carbohydrates, soda, sugar beverage, sweetened beverage, soft drink, dietary sugar, juice, sugar intake, sugary foods, sweets, sweet foods, carbohydrate intake, glycemic index, glycemic load, macronutrients AND diet, dietary patterns, dietary intake AND cohort studies, incidence, follow-up, prospective studies, meta-analysis AND Diabetes Mellitus, type 2 diabetes). We conducted a similar title,

abstract, and keyword search of the Scopus database (1143 titles): (diet* and sugar*) OR (diet* and pattern*) OR soda OR juice OR (sweet* and drink*) OR (sweet* and beverage*) OR (sweet* and food*) and ("type 2 diabetes"). The search results were downloaded into Refworks (©Proquest 2012). Titles, abstracts, and keywords of all articles were examined, and those that continued to meet the inclusion criteria were ascertained for further full text review.

To ensure accurate identification of eligible studies, we conducted two pilot tests of our methodology prior to implementing the search described above. First, we assessed and revised a search strategy after retrieval and review of citations from several years, 2010-2013. The revised search strategy included more terms and more specific terms for dietary sugar, glycemic load/index and energy intake. This led to a broader more inclusive search and the review of more titles. Secondly, two authors independently reviewed a subset of citations identified by our search strategy for eligibility (titles from 2012). Because both authors identified the same articles (inter-rater reliability = 100%), decisions regarding inclusion/exclusion reliably was based on review of one author.

Identification and tracking of eligible publications. A flow chart tracked eligible publications identified by the literature searches and illustrated a two-stage evaluation process (Figure 1).

As part of the process of identifying eligible cohorts we displayed how epidemiologic studies of SSB fit into the broader field of research on dietary sugar intake and T2D. We tabulated the cohorts that published on measures of dietary sugar intake (including SSB) by study size. The number of publications and corresponding cohorts were depicted for each definition of dietary sugar intake, including sweetened beverages and macronutrients (sucrose, fructose, & glucose). Table 1 was based on the World Health Organization and the Food and Agricultural Organization of the United Nations definitions [26] of dietary sugar intake as "all monosaccharides and disaccharides added to foods by the manufacturer, cook, or consumer; sugars naturally present in honey, syrups and fruit juices [27]." Table 1 facilitated identifying the studies that focus on SSB as a subset of all studies on dietary sugar intake.

Data abstraction. For eligible prospective observational studies of SSB and T2D, we created detailed data abstraction tables. For each study we abstracted data on sample size and population characteristics (e.g. country, baseline age and body size), SSB definition and consumption at baseline, T2D diagnosis, dietary assessment timing and tools, duration of follow-up, timing of ascertainment of beverage consumption, variables included in multivariable models, and statistical analyses. We present this work without linking study design features to study results. We recommend this step in order to minimize in as much is possible selection bias when planning a protocol for a subsequent meta-analysis. One author abstracted data at the onset, all authors contributed to strategy and map designs, and another author verified the accuracy of data abstracted at the end stage.

Step 1 (Map 1), Describing the range of definitions for the exposure variable, outcome, and co-variables. Evidence maps were used to categorize studies based on the definition of the exposure variable, taking into account the type of beverage, data collection instruments, and frequency/timing of data collection. Variation in definitions of T2D was evaluated based on criteria for diagnosis and method of ascertainment such as by a physician or self-report. Using the study as the unit of analysis, univariate statistics (n, median, proportion, range) were used to describe across included cohorts heterogeneity of SSB intake, T2D diagnosis, study location, gender, study size, duration of follow-up, baseline BMI, and baseline SSB consumption.

Step 2 (Map 2), Describing design features and population characteristics associated with the exposure across eligible cohorts. Cohorts were organized in a diagram according to category of the sweetened beverage consumption identified in Step 1. Design and population characteristics for cohorts falling into each beverage category were summarized. This provided an organized illustration of whether specific definitions of SSB tended to aggregate with specific study design features or population characteristics.

Step 3 (Map 3), Describing modeling strategies across eligible cohorts. Evidence-based mapping visually displayed the patterns of covariates adjusted for in each model of SSB and T2D as reported by each publication. A check denoted adjustment for co-variables age, smoking, physical activity, family history, alcohol intake, diet quality score, energy intake, and body mass index. Overall and within important strata of the study variable, diversity of multivariable modeling strategies was described by summarizing operationalization of the SSB intake (n, percent), the maximum number of models of SSB and T2D presented in the original publication (median, range), the maximum number of co-variables in models (median, range), and the maximum change in SSB-T2D risk estimate from the age-adjusted risk estimate (median, range).

Summarization of evidence-based mapping efforts (Table 2) Using cohorts as the unit of analysis, for categories defined by SSB-intake (as determined by Step 1) the following was tabulated: the diversity of important design and population characteristics (as determined in Step 2: n, percent), operationalization of the SSB in the multivariable model (Step 3: n, percent), the number of multivariable models of SSB-T2D presented in the original publication (Step 3: median, range), the number of co-variables in SSB-T2D multivariable models (Step 3: median, range), and the change in SSB-T2D risk estimate from the age-adjusted risk estimate (Step 3: median, range).

Results

Literature search and identification of eligible publications

The search results are summarized in Figure 1. Briefly, a total of 3148 titles were reviewed (2005 from Pubmed & 1143 from Scopus). After duplicate removal (N=180), 2968 titles were examined and reviewed in a two-step process. We identified 146 titles broadly on topic: a second review revealed that 90 were prospective epidemiology studies, commentary and reviews of sugar and T2D. Excluded were publications whose focus was not dietary sugar intake and T2D, and case-control, cross-sectional and ecologic studies (N=56). Full-text review of the 90 publications identified 22 primary research publications of the relationship between dietary sugar intake and T2D [28-49], and one meta-analysis with previously unpublished data [50]. A bibliography search of systematic reviews and meta-analyses did not identify any more potentially eligible titles [50-52].

Table 1 summarizes 21 publications from 17 cohorts that report the relationship between the following measures of dietary sugar intake and T2D [28-48]: category of SSB (broadly defined including some studies which include both artificially and sugar-sweetened beverages) and sugar-related macronutrients (total sugars, sucrose, fructose, glucose). Studies of SSB (broadly defined) represent the majority of the published work on measures of dietary sugar intake, with 15 publications from 11 cohorts, most with more than 5000 study participants. Nine publications from 8 cohorts analyze sugar-related macronutrients and T2D, with total sugars and sucrose being the most frequently assessed. With the exception of a Finnish study initiated prior to 1970, all studies of macronutrients have > 25,000 participants. A very small Swedish study assessing cake and biscuit consumption [49] was not summarized by Table 1.

Organizing and evaluating design heterogeneity among cohorts assessing SSB and T2D

For the assessment of design heterogeneity, we selected one publication from each cohort that had multiple publications (n=3 cohorts). We selected the one in which SSB was either the main study variable or the definition was the clearest. Details of unselected publications are noted at the bottom of Figure 1.

Step 1 (Map 1), Describing the range of definitions for the study variable, outcome, and co-variables. Study variables and outcomes were categorized into logical groups by definitions reported in each of the 11 eligible cohorts (Map 1). No two cohorts define the main study variable alike. As shown in Map 1, two definitions of sweetened beverage consumption emerged: (1) 3 studies used the nonspecific definition soft drinks (SD) that included both sugar and artificially-sweetened beverages, and (2) 8 studies restricted the definition to SSB-only. Three distinct subgroups were identified among cohorts defining the study variable as exclusively sugar-sweetened. The general definition "SSB, not 100% juice" includes all drinks with added sugar (sodas, colas, other carbonated SSB, non-carbonated SSBs such as fruit punches, lemonades or other fruit drinks). Two other SSB patterns were identified within the remaining five cohorts based on whether they excluded beverages (SSB minus, 3 cohorts) or

included additional beverages (SSB plus, 2 cohorts) from the anchor definition (SSB, not 100% juice). We found that investigators more frequently excluded beverages from the anchor definition, most broadly non-carbonated soft drinks as an entire group or fruit drinks. Two studies added beverages to the definition, one orange and grapefruit juice and the other non-alcoholic beer. This detailed characterization of the study variable identified two broad research question addressed by this series of selected studies, T2D risk associated with intake of 1) SSB-only or 2) any SD (artificially or sugar-sweetened).

Univariate analysis of design features and population characteristics across the 11 cohorts revealed heterogeneity in study location (US: n=6, Europe: n=2, China/Japan: n=3), size (> 5000 cases: n=2, 500-4999 cases: n=6, 1-499 cases: n=3), duration of follow-up (< 10 years: n=4, 10-14 years: n=4, 15 or more years: n=3); mean baseline body mass index (< 24 kg/m^2: n=3, 24-26 kg/m^2: n=4, > 26 kg/m^2: n=4), ascertainment of diet (food frequency questionnaire: n=8, diet history n=3), and frequency of diet assessment (baseline only: n=7, twice every 6 years: n=1, every 4 years: n=3). Of the 11 cohorts, method of diagnosis was based on self-report (n=6; 3 of the 6 were studies of health professionals), registry linkage (n=2), and an examination by a health professional (n=3). We also found a relatively low consumption of SSB across the 11 cohorts with nearly half (n=5) reporting that 10% or fewer participants consumed one or more servings per day.

Step 2 (Map 2), Describing design features and population characteristics associated with the study variable across eligible cohorts. The upper portion of Map 2 compares design features and population characteristics of cohorts defining sweetened beverage consumption as SSB-only and SD. SSB cohorts were mainly US-based (n=6), completed diet assessments at least twice (n=4), followed subjects for fewer than 10 years (n=4), and reported a mean body mass index (BMI) ≥ 24 kg/m^2 (n=7). We identified three levels of SSB consumption ascertained at the baseline visit of these 8 combined cohorts: frequency of one or more servings a day (highest consumption group for each study) was reported by 10% or fewer (n=2), between 11-15% (n=3) and more than 15% (n=2) of cohort participants.

Design and population characteristics for SD cohorts presented differently. Two of three SD cohorts were Asian populations; the one western population was a small Finnish study that enrolled participants between 1967 and 1972. Mean BMI was <24 kg/m^2 in 2 of the 3 studies, SD consumption was measured only at the baseline visit (10 to 12 years prior to maximum follow-up duration), and SD consumption overall three cohorts was low. Comparison of the study design features of cohorts assessing SSB-only with SD suggests it would not be sensible to combine all eleven studies in a meta-analysis, rather that the main pooled analysis include the 8 SSB-only studies.

Further division of SSB cohorts into categories of SSB, SSB minus, and SSB plus uncovered patterns according to study design, as shown in the lower portion of Map 2. Large studies of women (Black Women's Health Study [BWHS], Nurse's Health Study [NHS], NHSII) with

multiple dietary assessments more narrowly defined SSB consumption as excluding non-carbonated drinks (SSB, minus). The multicultural cohorts initiated to study atherosclerosis (Multi-Ethnic Study of Atherosclerosis [MESA], Atherosclerosis Risk in Community study [ARIC]) more broadly defined SSB by including either juice or non-alcoholic beer. These cohorts have higher baseline SSB consumption when compared to studies defining SSB more narrowly. No clear pattern emerged for cohorts defining the study variable as SSB, not 100% juice. Stratification of a meta-analysis on SSB subcategories and gender may additionally be important for understanding pooled T2D risk estimates.

We used a similar process to evaluate design heterogeneity of the outcome definitions used in these cohorts. While several different criteria for T2D were used in the 11 cohorts, the main defining characteristic was whether the diagnosis was based on self-report (all included a validation study), physical examination, or linkage to a registry or other health database. With the exception of the European Investigation of Cancer (EPIC) study, which verified cases via a registry, the larger studies (> 25,000 participants) relied on self-reported diagnoses. Three studies conducted routine physical exams.

Step 3 (Map 3), Diversity of modeling strategies. Multivariable models compared risk in the highest category of consumption (quartile or quintile) to the lowest. Map 3 summarizes the different definitions of high and low categories of sweetened beverage consumption. Among studies of SSB-only, the highest consumption category was 1+ glasses or servings each day in 6 (of 8) cohorts and 2+ drinks or cups each day for 2 (serving sizes varied). In comparison, the highest consumption category for 2 of 3 cohorts evaluating SD was less than one serving per day. Never or rare sweetened beverage consumption was the most frequently employed reference group (7 of 11), followed by never consumption (3 of 11). ARIC was the only study to include more frequent consumption in the reference group: up to one cup of SSB per day.

Map 3 visually depicts multivariable models and the pattern of covariate adjustment across 11 cohorts. The majority of cohorts present a multivariate model adjusting for age, physical activity, smoking, family history, alcohol intake, energy intake and BMI. Four studies adjust for a diet quality score, although all measure and adjust for some aspect of diet. Many models further adjust for multiple other covariates (up to 17).

Many models use different definitions to adjust for the listed co-variables and 9 of 11 adjust for covariates as measured at baseline. For example, measures of other dietary factors ranged from one variable measuring dietary fiber to healthy eating scores based on the entire diet (for 3 cohorts only). Likewise body mass index is adjusted in many ways: as a continuous variable (3 of 11 cohorts), a categorical variable (5 of 11 cohorts), and as measured at baseline (6 of 11 cohorts).

Multivariable models adjust for between 5 and 17 co-variables which corresponded to a 46% to 95% maximum reduction from the age-adjusted model in T2D risk associated with SSB-only

intake (Map 3). Change in the risk estimate was most pronounced among the large cohorts of US women using sugar-sweetened carbonated beverages as the study variable: reductions were as large as 95% in the BWHS, 61% in the NHS, and 67% in the NHSII. Change in risk estimates with addition of co-variables was less pronounced among SD cohorts (range, 2-26%).

Summarization of evidence-based mapping efforts. Table 2 concisely summarizes the considerable amount of variability in study design, population characteristics, and statistical analysis among the 11 cohorts of sweetened beverages and T2D. This table represents a prototype for a universal table on study design heterogeneity summarizing key design features uncovered by detailed evidence-based mapping efforts. The results in Table 2 are presented stratified by SSB-only and SD to display the association of different definitions of the study variable with specific design and population characteristics. In addition, Table 2 highlights diversity in statistical analysis overall and between definitions of sweetened beverage consumption.

Discussion

This evidence-based mapping strategy aims to improve the assessment and reporting of design heterogeneity prior to meta-analysis of epidemiologic studies. The framework described herein is useful for all study designs, but particularly for observational epidemiologic studies, which are complex and rich in important detail. If studies are found to be similar enough to combine via meta-analysis, this framework is useful for evaluating diversity in study designs, including statistical methods; facilitating the logical categorization of studies for stratified and sensitivity analyses when designing a protocol for meta-analysis; developing tools for model selection in meta-analysis of observational studies reporting multiple multivariable models; and designing a table for reporting heterogeneity across eligible studies in a meta-analysis.

Evidence maps are ideal tools for characterizing heterogeneity prior to a meta-analysis. Previously they have been used for research priority setting by the Cochrane Collaboration [53] and other organizations such as the Agency for Healthcare Research and Quality [54-56]. In addition to organizing a complex body of research, another defining feature of evidence mapping is that the mapping of study characteristics is undertaken without linking to study results [15]. Although we used prospective observational studies of SSB and T2D to explain our approach, the framework is robust and this strategy can be applied to other exposure-disease relationships and epidemiologic study types.

The logical grouping of studies on key design features using evidence maps suggested variables appropriate for stratified or sensitivity analyses, which is particularly useful when preparing a protocol for meta-analysis. Using SSB and T2D as an example, we found considerable variability in the definition and methods of collection of the exposure variable (sweetened beverages). Most notable is the inclusion of artificially-sweetened beverages in the definition in 3 of 11 cohorts. Consequently two broad research questions are addressed by this series of selected studies: T2D risk associated with intake of 1) SSB-only and 2) any soft drink (artificially or sugar-sweetened). Diversity in the definition of the exposure variable may be due to a combination of factors, including availability of data from the dietary assessment tool, the definition used by the study investigators, or the level of detail provided in the publication. Improving the interpretability of meta-analyses will require investigators of primary studies, in as much as possible, to define variables, conduct analyses, and report findings with an eye towards how their results may be compared to or possibly combined with other studies in the future.

Selection of statistical models by the study investigators from the primary publication and by a review investigator for a meta-analysis influences the final outcome of a pooled analysis of observational studies. This particular bias, called selective analysis reporting, has recently been discussed as a major concern for meta-analysis of observational studies [57]. Therefore, we also sought to summarize the diversity in statistical modeling techniques. Cohorts of SSB and T2D reported up to 8 multivariable models adjusting for a large number of co-variables and resulting

in a 2-95% reduction in risk of T2D associated with sweetened beverage consumption in fully adjusted relative to minimally adjusted models. In other words, in many studies adjustment for co-variables explained some to all of the association between SSB and T2D. Covariate selection and modeling strategies require careful consideration in the final interpretation of a pooled analysis of SSB and T2D. Evidence maps can help provide the tools necessary for this task.

When undertaking systematic reviews and meta-analyses of cohorts, careful consideration of the limitations of the observational nature inherent in the study design is necessary. Unlike experimental studies, in which the study variable is an investigator-designed intervention and subjects are randomized to equalize the groups being compared on all factors other than the intervention, associations from cohort studies are more prone to bias. The large fluctuation in the risk of T2D with SSB consumption depending on the covariates adjusted for in the model demonstrates the potential for bias intrinsic in observational studies. Consequently a single estimate for a pooled risk from a meta-analysis of SSB and T2D may be an oversimplification of complex data. Evidence maps can help facilitate the selection of additional models for sensitivity analyses in a meta-analysis.

The systematic approach described herein culminated in a prototype for a table that can be employed widely for reporting design heterogeneity across eligible studies in a meta-analysis. This table is recommended in addition to the classic table 1 in a systematic review (which usually describes studies individually). A standard table summarizing design heterogeneity across all selected studies will bring to the fore many elements necessary for interpreting the pooled risk estimate from a meta-analysis. One example from our assessment of cohorts of SSB and T2D is 7 of 11 studies measured beverage intake only once at baseline, each using a different diet questionnaire and following participants for T2D from 7 to 16 years. The etiologically relevant time period for most chronic diseases, including T2D, is most often not known, and a one-time measurement of dietary intake may not capture intake in the relevant time frame. This is a fundamental consideration when interpreting results of chronic disease studies, including meta-analysis of these data.

Heterogeneity of studies can be a reason not to perform a meta-analysis. For example, a systematic review of whole grain foods and T2D which had intended to complete a meta-analysis concluded a qualitative synthesis was more appropriate for the data [58]. Other investigators have determined that a meta-analysis of this topic was informative [59, 60]. A tool (such as the proposed summary table) that clearly displays design heterogeneity would be helpful in weighing both sides of this type of debate.

Systematic reviews and meta-analyses are well accepted research synthesis methods that serve to inform researchers, policy makers and, increasingly, the public of the potential causes of disease and the extent to which disease (or preventive) interventions are effective. The efficiency of these efforts depends largely on the quality of data from primary studies and a clear assessment of the extent to which that data can be combined.

Conclusion

We illustrate a framework employing evidenced-based mapping to organize, evaluate and document design heterogeneity. This exercise culminated in a recommendation for a standardized table format that clearly summarizes design heterogeneity of eligible studies, with the goal of informing a protocol for meta-analysis and subsequently facilitating interpretation of summary risk estimates after quantitative synthesis. We recommend expanding the practice of meta-analysis of cohort studies to include a standard table that summarizes design heterogeneity. Addition of this table to reporting of meta-analyses provides the reader with more evidence to interpret the summary risk estimates.

List of Abbreviations in Text

SSB = sugar-sweetened beverages; T2D = type 2 diabetes; SD = soft drinks; PICO = participants, exposure/intervention, comparator, and outcome; BWHS = Black Women's Health Study; NHS = Nurse's Health Study; MESA = Multi-Ethnic Study of Atherosclerosis; ARIC = Atherosclerosis Risk in Community study; EPIC = European Investigation of Cancer; BMI = body mass index.

Competing Interests Statement

This methodological study was supported by The Coca-Cola Company. By contractual agreement, decisions regarding the content of the manuscript –including the design, analysis and interpretation– rest solely with the authors.

Author Contributions

All authors contributed to the concept, design, interpretation of data and writing of the manuscript. MDA and CLF completed data abstraction and quality control.

Author's Information

We recognize that because this work was funded by industry it may be perceived as having an industry bias. Dr. Althuis and work at EpiContext seeks to synthesize data with extraordinary clarity using transparent and reproducible methods – with the ultimate goal of a product that provides the reader with enough data from which to establish his/her own conclusions – and thereby striving to present research objectively.

Reference List

1. Borenstein M, Hedges LV, Higgins JPT, et al. Introduction to meta-analysis. Hoboken, NJ:Wiley. 2008.
2. Egger M, Davey Smith G, Schneider M. Systematic reviews of observational studies. Chapter 12 in: Systematic Reviews in Health Care: Meta-analysis in Context. 2nd ed. Egger M, Davey Smith G, Altman DG (eds). BMJ Books. 2001:211-27.
3. Wong O, Raabe GK. Application of meta-analysis in reviewing occupational cohort studies. Occup Environ Med 1996;53:793-800.
4. Stroup DF, Berlin JA, Morton SC, Olkin I, Williamson GD, Rennie D, Moher D, Becker BJ, Sipe TA, Thacker SB. Meta-analysis of observational studies in epidemiology: a proposal for reporting. Meta-analysis Of Observational Studies in Epidemiology (MOOSE) group. JAMA. 2000 Apr 19;283(15):2008-12.
5. Blettner M, Sauerbrei W, Schlehofer B, Scheuchenpflug T, Friedenreich C. Traditional reviews, meta-analyses and pooled analyses in epidemiology. Int J Epidemiol. 1999 Feb;28(1):1-9.
6. Mosteller F, Colditz GA. Understanding research synthesis (meta-analysis). Annu Rev Pub Health 1996;17:1-23.
7. Thompson SG. Why and how sources of heterogeneity should be investigated. Chapter 9 in: Systematic Reviews in Health Care: Meta-analysis in Context. 2nd ed. Egger M, Davey Smith G, Altman DG (eds). BMJ Books. 2001:157-74.
8. Thompson SG. Why sources of heterogeneity in meta-analysis should be investigated. BMJ 1994;309:1351-5.
9. Deeks JJ, Higgins JPT, Altman DG. Analysing data and undertaking meta-analyses. Chapter 9 in: Cochrane Handbook for Systematic Reviews of Interventions. Higgins JPT, Green S (eds). Wiley-Blackwell. 2008:243-296.
10. Higgins JP, Thompson SG, Deeks JJ, Altman DG. Measuring inconsistency in meta-analyses. BMJ. 2003 Sep 6;327(7414):557-60.
11. McElvenny DM, Armstrong BG, Järup L, Higgins JP. Meta-analysis in occupational epidemiology: a review of practice. Occup Med (Lond). 2004 Aug;54(5):336-44.
12. Reeves BC, Higgins JPT, Ramsay C, Shea B, Tugwell P, Wells GA. An introduction to methodological issues when including non-randomised studies in systematic reviews on the effects of interventions. Res Syn Meth, 2013 (4) 1-11.
13. Wells GA, Shea B, Higgins JPT, Sterne J, Tugwell P, Reeves BC. Checklists of methodologic issues for review authors to consider when including non-randomized studies in systematic reviews. Res Syn Meth. 2013 (4) 63-77.
14. Althuis MD, Weed DL. Evidence mapping: methodologic foundations and application to intervention and observational research on sugar-sweetened beverages and health outcomes. Am J Clin Nutr. 2013 Sep;98(3):755-68.
15. Callahan P, Liu P, Purcell R, Parker AG, Hetrick SE. Evidence map of prevention and treatment interventions for depression in young people. Depress Res Treat. 2012;2012:820735.
16. Gough D, Thomas J, Oliver S. Clarifying differences between review designs and methods. Syst Rev. 2012 Jun 9;1(1):28.
17. Bragge P, Clavisi O, Turner T, Tavender E, Collie A, Gruen RL. The Global Evidence Mapping Initiative: scoping research in broad topic areas. BMC Med Res Methodol. 2011 Jun 17;11:92.
18. Hetrick SE, Parker AG, Callahan P, Purcell R. Evidence mapping: illustrating an emerging methodology to improve evidence-based practice in youth mental health. J Eval Clin Pract. 2010 Dec;16(6):1025-30.

19. Levac D, Colquhoun H, O'Brien KK. Scoping studies: advancing the methodology. Implement Sci. 2010 Sep 20;5:69.
20. Liu Y, Wen XM, Lui EL, Friedman SL, Cui W, Ho NP, Li L, Ye T, Fan ST, Zhang H. An evidence map of interventions across premorbid, ultra-high risk and first episode phases of psychosis. Schizophrenia Research 2010;123:37-44.
21. Curran C, Burchardt T, Knapp M, McDaid D, Li B. Challenges in multidisciplinary systematic reviewing: a study on social exclusion and mental health policy. Social Policy Adminstration 2007;41:289-312.
22. Arksey H, O'Malley L. Scoping studies: towards a methodological framework. Int J Social Research Methodology 2005;8:19-32.
23. Katz DL, Williams AL, Girard C, Goodman J, Comerford B, Behrman A, Bracken MB. The evidence base for complementary and alternative medicine: methods of evidence mapping with application to CAM. Altern Ther Health Med 2003;9:22-30.
24. Moher D, Liberati A, Tetzlaff J, Altman DG; PRISMA Group. Preferred reporting items for systematic reviews and meta-analyses: the PRISMA statement. BMJ. 2009 Jul 21;339:b2535.
25. de Costa Santos CM, de Mattos Pimenta CA, Nobre MR.The PICO strategy for the research question construction and evidence search. Rev Lat Am Enfermagem. 2007;15(3):508-11.
26. Mann J, Cummings JH, Englyst HN, Key T, Liu S, Riccardi G, Summerbell C, Uauy R, van Dam RM, Venn B, Vorster HH, Wiseman M. FAO/WHO scientific update on carbohydrates in human nutrition: conclusions. Eur J Clin Nutr. 2007 Dec;61 Suppl 1:S132-7.
27. Te Morenga L, Mallard S, Mann J. Dietary sugars and body weight: systematic review and meta-analyses of randomised controlled trials and cohort studies. BMJ. 2012 Jan 15;346:e7492.
28. Palmer JR, Boggs DA, Krishnan S, Hu FB, Singer M, Rosenberg L. Sugar-sweetened beverages and incidence of type 2 diabetes mellitus in African American women. *Arch Intern Med.* 2008;168(14):1487-1492.
29. InterAct Consortium. Consumption of sweet beverages and type 2 diabetes incidence in European adults: Results from EPIC-InterAct. *Diabetologia.* 2013;56(7):1520-1530.
30. Sluijs I, Beulens JW, van der Schouw YT, et al. Dietary glycemic index, glycemic load, and digestible carbohydrate intake are not associated with risk of type 2 diabetes in eight European countries. *J Nutr.* 2013;143(1):93-99.
31. Fagherazzi G, Vilier A, Sartorelli DS, Lajous M, Balkau B, Clavel-Chapelon F. Consumption of artificially and sugar-sweetened beverages and incident type 2 diabetes in the etude epidé miologique auprè s des femmes de la mutuelle Générale de l'education nationale-european prospective investigation into cancer and nutrition cohort. *Am J Clin Nutr.* 2013;97(3):517-523.
32. Sluijs I, van der Schouw YT, van der ADL, et al. Carbohydrate quantity and quality and risk of type 2 diabetes in the european prospective investigation into cancer and nutrition-Netherlands (EPIC-NL) study. *Am J Clin Nutr.* 2010;92(4):905-911.
33. Schulze MB, Schulz M, Heidemann C, Schienkiewitz A, Hoffmann K, Boeing H. Carbohydrate intake and incidence of type 2 diabetes in the European prospective investigation into cancer and nutrition (EPIC)-Potsdam study. *Br J Nutr.* 2008;99(5):1107-1116.
34. de Koning L, Malik VS, Rimm EB, Willett WC, Hu FB. Sugar-sweetened and artificially sweetened beverage consumption and risk of type 2 diabetes in men. *Am J Clin Nutr.* 2011;93(6):1321-1327.
35. Bhupathiraju SN, Pan A, Malik VS, et al. Caffeinated and caffeine-free beverages and risk of type 2 diabetes. *Am J Clin Nutr.* 2013;97(1):155-166.

36. Meyer KA, Kushi LH, Jacobs DR,Jr, Slavin J, Sellers TA, Folsom AR. Carbohydrates, dietary fiber, and incident type 2 diabetes in older women. *Am J Clin Nutr.* 2000;71(4):921-930.
37. Eshak ES, Iso H, Mizoue T, Inoue M, Noda M, Tsugane S. Soft drink, 100% fruit juice, and vegetable juice intakes and risk of diabetes mellitus. *Clinical Nutrition.* 2013;32(2):300-308.
38. Hodge AM, English DR, O'Dea K, Giles GG. Glycemic index and dietary fiber and the risk of type 2 diabetes. *Diabetes Care.* 2004;27(11):2701-2706.
39. Bazzano LA, Li TY, Joshipura KJ, Hu FB. Intake of fruit, vegetables, and fruit juices and risk of diabetes in women. *Diabetes Care.* 2008;31(7):1311-1317.
40. Colditz GA, Manson JE, Stampfer MJ, Rosner B, Willett WC, Speizer FE. Diet and risk of clinical diabetes in women. *Am J Clin Nutr.* 1992;55(5):1018-1023.
41. Schulze MB, Manson JE, Ludwig DS, et al. Sugar-sweetened beverages, weight gain, and incidence of type 2 diabetes in young and middle-aged women. *JAMA.* 2004;292(8):927-934.
42. Odegaard AO, Koh WP, Arakawa K, Yu MC, Pereira MA. Soft drink and juice consumption and risk of physician-diagnosed incident type 2 diabetes: The Singapore Chinese health study. *Am J Epidemiol.* 2010;171(6):701-708.
43. Janket SJ, Manson JE, Sesso H, Buring JE, Liu S. A prospective study of sugar intake and risk of type 2 diabetes in women. *Diabetes Care.* 2003;26(4):1008-1015.
44. Paynter NP, Yeh H-, Voutilainen S, et al. Coffee and sweetened beverage consumption and the risk of type 2 diabetes mellitus: The atherosclerosis risk in communities study. *Am J Epidemiol.* 2006;164(11):1075-1084.
45. Nettleton JA, Lutsey PL, Wang Y, Lima JA, Michos ED, Jacobs DR,Jr. Diet soda intake and risk of incident metabolic syndrome and type 2 diabetes in the multi-ethnic study of atherosclerosis (MESA). *Diabetes Care.* 2009;32(4):688-694.
46. Ahmadi-Abhari S, Luben RN, Powell N, et al. Dietary intake of carbohydrates and risk of type 2 diabetes: The european prospective investigation into cancer-Norfolk study. *Br J Nutr.* 2013:1-11.
47. Montonen J, Jarvinen R, Knekt P, Heliovaara M, Reunanen A. Consumption of sweetened beverages and intakes of fructose and glucose predict type 2 diabetes occurrence. *J Nutr.* 2007;137(6):1447-1454.
48. Sakurai M, Nakamura K, Miura K, et al. Sugar-sweetened beverage and diet soda consumption and the 7-year risk for type 2 diabetes mellitus in middle-aged Japanese men. *Eur J Nutr.* 2013:1-8.
49. Lundgren H, Bengtsson C, Blohme G, et al. Dietary habits and incidence of noninsulin-dependent diabetes mellitus in a population study of women in Gothenburg, Sweden. *Am J Clin Nutr.* 1989;49(4):708-712.
50. Malik VS, Popkin BM, Bray GA, Després JP, Willett WC, Hu FB. Sugar-sweetened beverages and risk of metabolic syndrome and type 2 diabetes: a meta-analysis. Diabetes Care. 2010 Nov;33(11):2477-83.
51. Alhazmi A, Stojanovski E, McEvoy M, Garg ML. Macronutrient intakes and development of type 2 diabetes: a systematic review and meta-analysis of cohort studies. J Am Coll Nutr. 2012 Aug;31(4):243-58.
52. Hauner H, Bechthold A, Boeing H, Brönstrup A, Buyken A, Leschik-Bonnet E, Linseisen J, Schulze M, Strohm D, Wolfram G; German Nutrition Society. Evidence-based guideline of the German Nutrition Society: carbohydrate intake and prevention of nutrition-related diseases. Ann Nutr Metab. 2012;60 Suppl 1:1-58. Lonardo A, Adinolfi LE, Petta S, Craxì A, Loria P. Hepatitis C and diabetes: the inevitable coincidence? Expert Rev Anti Infect Ther. 2009 Apr;7(3):293-308.

53. Jaramillo A, Welch VA, Ueffing E, Gruen RL, Bragge P, Lyddiatt A, Tugwell P. Prevention and self-management interventions are top priorities for osteoarthritis systematic reviews. J Clin Epidemiol. 2012 Sep 17. doi:pii: S0895-4356(12)00219-3.
54. Greer N, Brasure M, Wilt TJ. Wheeled mobility (wheelchair) service delivery [Internet]. Rockville (MD): Agency for Healthcare Research and Quality (US); 2012 Jan. Available from http://www.ncbi.nlm.nih.gov/books/NBK83951/
55. Wysocki A, Butler M, Shamliyan T, Kane RL. Whole-body vibration therapy for osteoporosis [Internet]. Rockville (MD): Agency for Healthcare Research and Quality (US); 2011 Nov. Available from http://www.ncbi.nlm.nih.gov/books/NBK83043/
56. Chung M, Dahabreh IJ, Hadar N, Ratichek SJ, Gaylor JM, Trikalinos TA, Lau J. Emerging MRI technologies for imaging musculoskeletal disorders under loading stress [Internet]. Rockville (MD): Agency for Healthcare Research and Quality (US); 2011 Nov. Available from http://www.ncbi.nlm.nih.gov/books/NBK82287/
57. Norris SL, Moher D, Reeves BC, Shea B, Loke Y, Garner S, Anderson L, Tugwell P, Wells G. Issues relating to selective reporting when including non-randomized studies in systematic reviews on the effects of healthcare interventions. Res Syn Meth. 2013 (4) 36-47.
58. Priebe MG, van Binsbergen JJ, de Vos R, Vonk RJ. Whole grain foods for the prevention of type 2 diabetes mellitus. Cochrane Database Syst Rev. 2008 Jan 23;(1):CD006061.
59. Ye EQ, Chacko SA, Chou EL, Kugizaki M, Liu S. Greater whole-grain intake is associated with lower risk of type 2 diabetes, cardiovascular disease, and weight gain. J Nutr. 2012 Jul;142(7):1304-13.
60. de Munter JS, Hu FB, Spiegelman D, Franz M, van Dam RM. Whole grain, bran, and germ intake and risk of type 2 diabetes: a prospective cohort study and systematic review. PLoS Med. 2007 Aug;4(8):e261.

Figure 1. Systematic search for eligible studies of dietary sugar intake and type 2 diabetes

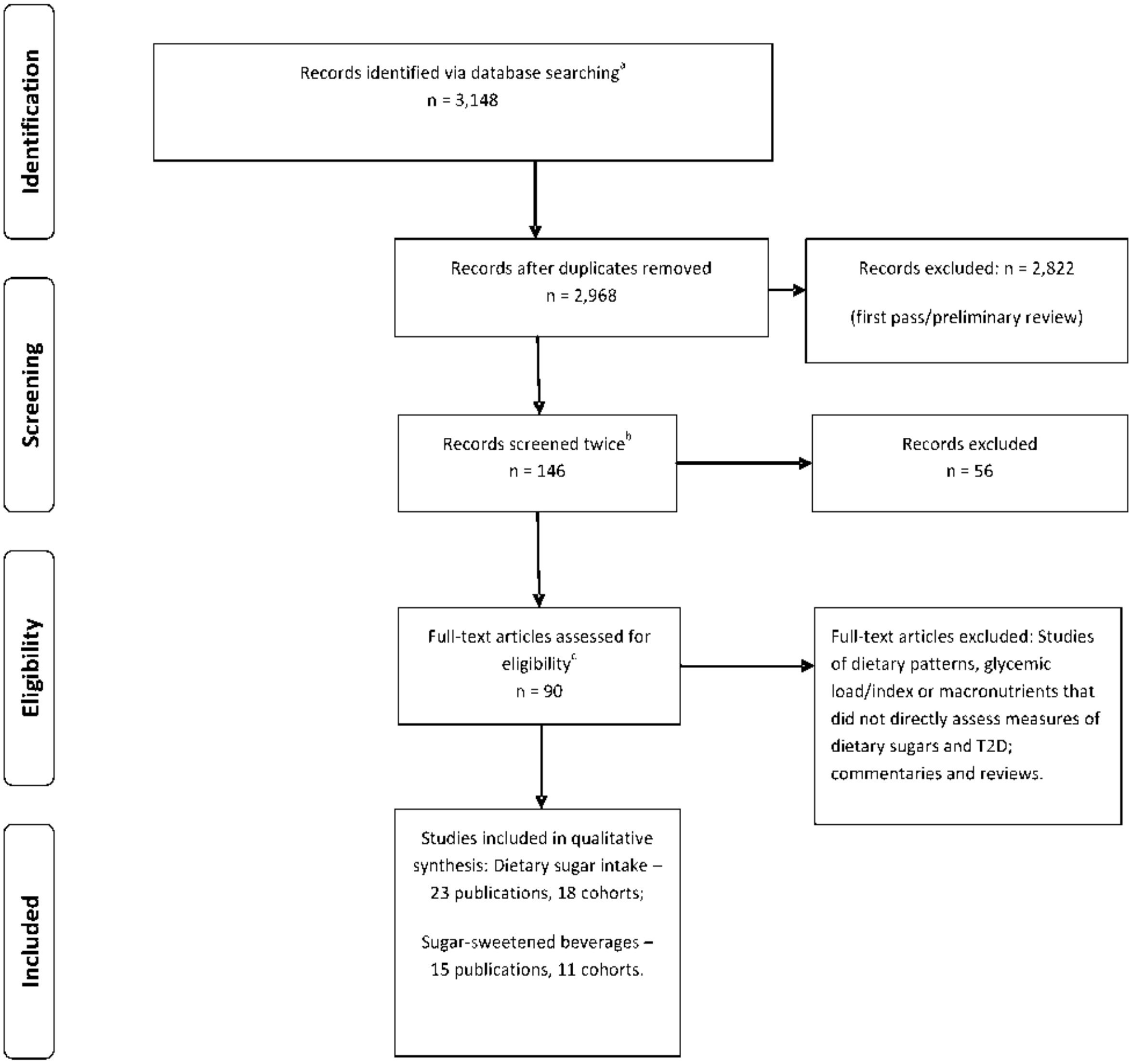

a. 2005 from Pubmed and 1143 from Scopus data base searches.
b. Titles remotely on topic were screened twice.
c. We complete full-text review of all studies of dietary patterns, glycemic load/index, and carbohydrates to assess whether a measure of dietary sugar was examined individually. We also reviewed the full text and bibliographies of studies of sugar-sweetened beverages, juices, sugars, macronutrients and key reviews and commentary.
d. We identified 3 cohorts with multiple publications, from which we selected for this synthesis the one in which SSB was either the main study variable or the definition was the clearest. We identified two publications of the Health Professionals Follow-up study (HPFS), the one that assessed SSB as the primary study variable was selected for inclusion [34] and the other that presented analyses stratified by the main variable, caffeine consumption, was excluded [35]. We selected one of the three publications from the Nurse's Health Study (NHS). Bazzano and coworkers [39] reported risk separately for a one-increment serving of sugar-sweetened colas, fruit punch, low caloric cola, and other carbonated beverage. In a personal communication from a 2010 meta-analysis [50], Malik and coworkers report a risk estimate for SSB intake, but the definition was not provided nor was the analysis adjusted for age. Although not ideal, the Bhupathiraju and coworker analysis of SSB, stratified by caffeinated and caffeine-free beverage consumption, provides a clear definition (sugar-sweetened carbonated beverages) and analysis, and therefore was selected for inclusion in this paper [35]. Our final exclusion was a 2013 publication of EPIC-France [31], from which all participants were represented by an included EPIC publication [29].

Table 1. Publications and cohorts that report the relationship between measures of dietary sugar intake and Type 2 diabetes

Cohorts (reference)[1]	SSB - broadly defined	Macronutrients: Total sugars[2]	Sucrose	Fructose	Glucose	Fructose & Glucose
> 25,000 Participants						
BWHS [28]	√					
EPIC-All [29, 30]	√(29)	√(30)				
EPIC-FR [31]	√					
EPIC-NL [32]		√				
EPIC-P [33]			√	√	√	
HPFS [34,35]	√					
IWHS [36]			√	√	√	
JPHC [37]	√					
MelC [38]		√				
NHS [35, 39, 40]	√(35,39)		√(40)			
NHSII [41]	√					
SCHS [42]	√					
WHS [43]		√	√	√	√	
10,000 to 24,999 Participants						
ARIC [44]	√					
5000 to 9,999 Participants						
MESA [45]	√					
1000 to 4,999 Participants						
EPIC-Nor [46]		√	√	√	√	
FMC [47]	√	√	√	√	√	√
Jfact [48]	√					
Total publications:	15	6	6	5	5	1
Total unique cohorts represented:[3]	11	4	6	5	5	1

Macronutrients: 9 Publications: 8 Cohorts

1. ARIC – Atherosclerosis Risk in Communities Study, WHS – Women's Health Study (B–Black, I–Iowa), EPIC-All, P, N, NL, FR – European Prospective Investigation of Cancer (InterAct Study, Potsdam, Norfolk, Netherlands, France), FMC – Finnish Mobile Clinic Heath Examination Survey, HPFS – Health Professional's Follow up Study, Jfact – study of Japanese factory workers, JPHC – Japan Public Health Centre-based Prospective Study, MESA – Multi-Ethnic Study of Atherosclerosis, NHS – Nurse's Health Study, SCHS –Singapore Chinese Health Study.
2. Total sugars – disaccarides and monosaccarides.
3. Total cohorts represented enumerates unique cohorts. Eight of 10 countries are represented in EPIC-All, which overlaps with country specific EPIC publications except for Norway & Greece.

Note: SSB = sugar-sweetened beverages. In this map SSB was broadly defined to include studies that defined sweetened beverages as either SSB-only or soft drinks (either sugar and artificially sweetened).

Map 1 (Step 1). Categorizing cohorts according to the definition of the study variable, sugar-sweetened beverages

Study variable evalutated for 11 Cohorts

Definition clearly excludes artificially-sweetened beverages.

Only SSB
8 Cohorts

SSB, Not 100% Juice (3 Cohorts)

EPIC [29]: Sugar-sweetened carbonate/soft/isotonic drinks and diluted syrups: 1 glass ~ 8.8 oz serving

HPFS [34]: Sugar-sweetened colas, other carbonated & non-carbonated beverages (fruit punches, lemonades, or other fruit drinks). Serving size = standard glass, can, or bottle.

Jfact [48]: Regular soft drinks, sugar-sweetened soda, sports drinks excluding 100% juice: 1 glass ~ 250 mL.

SSB, Minus… (3 Cohorts)

NHS [35]: Sugar-sweetened carbonated beverages; did not include fruit drinks or uncarbonated SSB. Serving size = a standard glass, can or bottle.

BWHS [28]: Regular soft drinks (not diet soda). Other fruit juices, fortified fruit drinks and Kool-aid assessed separately.
serving ~ 12 oz.

NHSII [41]: Coke, Pepsi or other cola with sugar, carbonated beverage with sugar, frequency of a "common portion".

SSB, Plus… (2 Cohorts)

ARIC [44]: Fruit punch, non-diet soda, orange and grapefruit juice, frequency of a 1 cup (8 oz) serving.

MESA [45]: Regular soft drinks, sweetened mineral water (not diet), non-alcoholic beer, serving size not specified.

Inclusion of artificially-sweetened beverages cannot be ruled out.

All Soft Drinks (SD)
3 Cohorts

SCHC [42]: SD, NOS measured in glasses of intake; investigators expected heterogeneity of serving size.

JPHC [37]: Colas and fruit drinks, sugar and artificially-sweetened. Serving size not specified.

FMC [47]: SD, NOS; g/day. No details provided. Variable only available for a subset of the population.

Note: ARIC = Atherosclerosis Risk in Communities Study, BWHS = Black Women's Health Study, EPIC = European Prospective Investigation of Cancer (InterAct Study), FMC = Finnish Mobile Clinic Heath Examination Survey, HPFS = Health Professional's Follow up Study, Jfact = study of Japanese factory workers, JPHC = Japan Public Health Centre-based Prospective Study, MESA = Multi-Ethnic Study of Atherosclerosis, NHS = Nurse's Health Study, SCHS =Singapore Chinese Health Study; SD = soft drink; SSB = sugar-sweetened beverage.

Map 2 (Step 2). Sweetened beverage definitions by cohort description and methods: studies of incident type 2 diabetes

SSB, broadly defined
11 Cohorts

Definition clearly excludes artificially-sweetened beverages. | *Inclusion of artificially-sweetened beverages cannot be ruled out.*

**Only SSB (8 Cohorts)**		**All Soft Drinks (SD) (3 Cohorts)**
US (6) - multi-ethnic cohorts[ARIC, MESA]<br>Europe (1) - eight countries<br>Japan (1)	**Location (ethnicity)**	China (1): 2,273 T2D cases[SR, SCHS [42]]<br>Japan (1): 824 T2D cases[SR, JPHS [37]]<br>Finland (1) - 177 cases[Reg, enrolled 1976-72, FMC [47]]
Once (4)<br>Twice: 6 y interval (1)<br>Every 4 years (3)	**Number of beverage assessments**	One (3)
< 10 (4)<br>10 to 19 y (2)<br>20 to 24 y (2)	**Maximum follow-up (range)**	10-12 y (3)
< 24 (1)<br>24-26 (4)<br>> 26 (3)	**Mean baseline BMI (kg/m^2)**	< 24 (2)<br>24-26 (0)<br>> 26 (1)[FMC [47]]
% reporting ≥ 1 serving/d:<br>10% or fewer (2)<br>Between 11-15% (3)<br>More than 15% (2)<br>Not reported (1)	**Highest consumption category**	Low overall consumption:<br>mean ~ 0.2 servings/d (1)[FMC [47]]<br>≥ 2 glasses/wk (1)[SCHS [42]]: 10.6%<br>≥ 1 serving/d (1)[JPHC [37]]: 9% men, 5% women

SSB, Not 100% Juice (3 Cohorts)

**Cohort:**	**EPIC [29]**	**HPFS [34]**	**Jfact [48]**
**Cases (T2D):**	11,684[Reg]	2680[SR, M]	170[Ex, M]
**Maximum follow-up:**	16 y	20 y	7 y
**Beverage assessments:**	Once (BL)	Every 4 yrs	Once (BL)
**Highest consumption category:**	≥ 1 serving/d: 8%	4.5 servings/wk to 7.5/d: 25%	≥ 1 serving/d: 12%

SSB, Minus... (3 Cohorts)

**Cohort:**	**NHS [35]**	**BWHS [28]**	**NHS II [41]**
**Cases (T2D):**	7370[SR, W]	2713[SR, W]	741[SR, W]
**Maximum follow-up:**	24 y	10 y	8 y
**Beverage assessments:**	Every 4 yrs	Twice: 6 y interval	Twice: every 4 yrs
**Highest consumption category:**	NR	≥ 1 serving/d: 17%	≥ 1 serving/d: 9.5%
**Definition:**	Sugar-sweetened carbonated beverage	Separate analysis for fruit drinks	Sugar-sweetened carbonated beverages

SSB, Plus... (2 Cohorts)

**Cohort:**	**ARIC [44]**	**MESA [45]**
**Cases (T2D):**	1437[Ex]	413[Ex]
**Maximum follow-up:**	9 y	6-7 y
**Beverage assessments:**	Once (BL)	Once (BL)
**Highest consumption category:**	≥ 1 serving/d: 17% men, 13% women	≥ 1 serving/d: 14%
**Plus:**	Orange & grape-fruit juice	Non-alcoholic beer

Note: T2D diagnosed by self-report of symptoms/medication or physician diagnosis (SR), linkage to a registry (Reg), or upon exam (Ex), NR = not reported, BL = baseline, M = men, W = women; ARIC = Atherosclerosis Risk in Communities Study, BWHS = Black Women's Health Study, EPIC = European Prospective Investigation of Cancer (InterAct Study), FMC = Finnish Mobile Clinic Heath Examination Survey, HPFS = Health Professional's Follow up Study, Jfact = study of Japanese factory workers, JPHC = Japan Public Health Centre-based Prospective Study, MESA = Multi-Ethnic Study of Atherosclerosis, NHS = Nurse's Health Study, SCHS =Singapore Chinese Health Study; SD = soft drink; SSB = sugar-sweetened beverage.

Map 3 (Step 3). Covariates adjusted for in multivariable models of sugar-sweetened beverages and type 2 diabetes: 11 cohorts

Cohort (ref)	Hi v. lo Comparison	Model (no. covar.)	% Reduction from least adjusted[1]	Age	Smoking	Phys. Activity	Fam. Hist.	Alcohol Intake	Diet Quality Score	EI	BMI	Other covariates (diet score in italic):
STUDIES OF ONLY SUGAR SWEETENED BEVERAGES, NOT 100% JUICE												
EPIC-ALL	≥1 glass/d vs.	1 (1)	0	√								Models 2-4 were also adjusted for gender,
[29]	< 1 glass/mo	2 (7)	42%	√	√BL	√BL		√BL				education level, juice and artificially-
	(BL→16y)	3 (8)	42%	√	√BL	√BL		√BL		√BL		sweetened beverage intake.
		4 (9)	57%	√	√BL	√BL		√BL		√BL	√BL	
HPFS	4.5 servings/wk to	1 (1)	0	√								Models 2-8 also adjusted for multivitamin use.
[34]	7.5 servings/d	2 (5)	16%	√	√	√		√				Model 4 further adjusted for high triglycerides
	vs. never	3 (6)	12%	√	√	√	√	√				in 1986, high blood pressure, & diuretics.
		4 (9)	20%	√	√	√	√	√				Model 5 further adjusted for weight gain or loss
	(Cumulative	5 (11)	0%	√	√	√	√	√				between 1981-1986 and adherence to a low
	average up to 2y	6 (12)	12%	√	√	√	√	√	√			calorie diet in 1994. Model 6 further adjusts for
	prior to interval	7 (13)	52%	√	√	√	√	√	√	√		the *Alternative Healthy Eating Index*.
	→ 20y)	8 (14)	4%	√	√	√	√	√	√	√	√	Model 7for energy intake, and Model 8 for BMI.
Japanese	≥1 serving/d	1 (1)	0									Models 3 & 4 are additionally adjusted for
Factory	vs. rare or never	2 (2)	17%	√							√BL	hypertension, dyslipidemia, diet treatment for
[48]	(BL→7y)	3 (11)	↑46%	√	√BL	√BL	√BL	√BL		√BL	√BL	chronic disease, and fiber intake. Model 4 is
		4 (15)	↑42%	√	√BL	√BL	√BL	√BL		√BL	√BL	further adjusted for diet soda, juice and coffee.
STUDIES OF SUGAR SWEETENED BEVERAGES, DEFINITION EXCLUDING SOME SSB TYPES												
NHS	≥ 1 serving SSB/d	Caffeinated:										Models 2,3 are also adjusted for postmenopausal
[35]	vs. < 1 serving/mo	1 (1)	0	√								hormone replacement therapy, coffee, fruit
		2 (15)	19%	√	√	√	√	√	√			punch, caffeinated tea, artificially-sweetened
	Cumulative	3 (17)	61%	√	√	√	√	√	√	√	√	beverages, hypertension, hypercholesterolemia,
	average up to 2y	Caffeine-free:										low-calorie diet in 1992, weight change
	prior to interval	1 (1)	0	√								beween 1981-1986, *Alternative Healthy*
	→ 24y)	2 (15)	52%	√	√	√	√	√	√			*Eating Index*.
		3 (17)	57%	√	√	√	√	√	√	√	√	
BWHS	> 2 drinks/d	1 (1)	0	√								Models 2-5 adjusted for years of education,
[28]	vs. < 1/mo	2 (7)	33%	√	√	√BL	√BL					sweetened fruit drinks (BL & Y6), orange and
	(BL or Y6→10y)	3 (12)	68%	√	√	√BL	√BL		√BL			grapefruit juice (BL & Y6). Models 3-5 also
		4 (13)	93%	√	√	√BL	√BL		√BL		√	adjusted for red meat, processed meats, cereal
		5 (14)	95%	√	√	√BL	√BL		√BL	√BL	√	fiber, coffee, *glycemic index* (BL). Model 4 further adjusted for BMI and model 5 for BMI (BL or Y6) and energy intake.
NHSII	>1 serving/d	1 (1)	ref	√								Models 2-4 also adjusted for hormone use,
[41]	vs. <1 mo	2 (14)	15%	√	√	√	√BL	√				oral contraceptive use, cereal fiber, trans-fat,
	(BL&Y4→8y)	3 (15)	60%	√	√	√	√BL	√			√	ratio of polyunsaturated fat, magnesium, diet
		4 (16)	67%	√	√	√	√BL	√		√	√	soft drinks, fruit juice/punch.

1. Calculated as porportion change from the age-adjusted model or a fairly simple model: $(RR_{age\ adjusted} - RR_{model})/(RR_{age\ adjusted} - 1)$. ↑ denotes an increase in the risk estimate.
2. For the MESA cohort, the model information was based on author correspondence reported in a 2010 meta-analysis [50].
Note: BL – adjustment variable based on baseline assessment. All cohorts used Cox proportional hazards models, except JPHC [37], which used logistic regression.
Continued.

Map 3 (Step 3) continued. Covariates adjusted for in multivariable models of sugar-sweetened beverages and type 2 diabetes: 11 cohorts

Cohort	Hi v. lo Comparison	Model (no. covar.)	% Reduction from least adjusted[1]	Age	Smoking	Phys. Activity	Fam. Hist.	Alcohol Intake	Diet Quality Score	EI	BMI	Other covariates:
STUDIES OF SUGAR SWEETENED BEVERAGES, DEFINITION INCLUDING JUICE OR NONALCOHOLIC BEER												
ARIC	2+ Cups/d vs.	Men: 1 (2)	0	√								All models adjusted for race. Models 2 are
[44]	< 1 cup/d	2 (12)	67%	√	√BL	√B-	√BL	√B-		√B-	√BL	additionally adjusted for baseline measures of
	(BL→9y)	Women:										education, dietary fiber, hypertension &
		1 (2)	0	√								waist-hip ratio.
		2 (12)	94%	√	√BL	√B-	√BL	√B-		√B-	√BL	
	Investigators note that removing juice from the sweetened beverage definition did not affect the results.											
MESA	≥ 1 serving/d	1 (10)		√	√BL	√B-				√B-		Model also adjusted for gender, BL waist circum-
[50][2]	vs. 0 (BL →6y)											ference, race, education, BL supplement use.
STUDIES OF SOFT DRINKS, SUGAR AND ARTIFICIALLY SWEETENED												
SCHS	2-3+ servings/wk	1 (4)	0%	√								All models adjusted for gender, Chinese dialect,
[42]	vs. almost never	2 (13)	9%	√	√BI	√B.		√B.				& year of interview. Model 2 further adjusted
	(BL→10y)	3 (15)	26%	√	√BL	√B-		√B-		√B-	√BL	for educational level, coffee consumption, fiber, saturated fat, dairy and juice intake.
JPHC	Almost every day	BL →5 yrs (Men):										Models 2 and 4 further adjusted for baseline
[37]	vs. rarely	1 (1)	0	√BL								measure of education, occupation, history of
		2 (17)	↑2%	√BI	√BI	√B.	√BI	√B.		√B.	√BI	hypertension, coffee, green tea, dietary
		BL→10 yrs (Men):										magnesium, calcium, vitamin D, rice and
		3 (1)	0	√BI								dietary fiber.
		4 (17)	2%	√BL	√BL	√B-	√BL	√B-		√B-	√BL	
		BL →5 yrs (Women):										
		1 (1)	0	√BL								
		2 (17)	18%	√BI	√BI	√B.	√BI	√B.		√B.	√BI	
		BL→10 yrs (Women):										
		3 (1)	0	√BI								
		4 (17)	19%	√BL	√BL	√B-	√BL	√B-		√B-	√BL	
FMC	Quartiles	1 (8)	Age-	√	√BL	√B-	√BL			√B-	√BL	Models adjusted for gender, geographical area,
[47]	(median g/d)	2 (9)	adjusted	√	√BI	√B.	√BI		√BI	√B.	√BI	*prudent & conservative dietary pattern score.*
	143 g/d vs. 0	3 (14)	model NR	√	√BL	√B-	√BL		√BL	√B-	√BL	Model 3 further adjusted for serum cholesterol,
	(BL→12y)											blood pressure, history of infarction, angina pectoris or cardiac failure.

1. Calculated as porportion change from the age-adjusted model or a fairly simple model: $(RR_{age\ adjusted} - RR_{model})/(RR_{age\ adjusted} - 1)$. ↑ denotes an increase in the risk estimate.
2. For the MESA cohort, the model information was based on author correspondence reported in a 2010 meta-analysis [50].
Note: BL = adjustment variable based on baseline assessment. All cohorts used Cox proportional hazards models, except JPHC [37], which used logistic regression.

Table 2. Design heterogeneity across 11 cohorts assessing risk of type 2 diabetes, stratafied by inclusion of artificially-sweetened beverages in the study variable definition

	Only SSB (8 Cohorts)		SD (3 Cohorts)	
**Study Design and Population Characteristics**	**n**	**%**	**n**	**%**
Study location				
USA	6	75%	0	
Europe	1	13%	1	33%
Japan/China	1	13%	2	67%
Gender				
Women	3	38%	0	
Men	2	25%	0	
Both men and women	3	38%	3	100%
Cases of T2D				
1-499	2	25%	1	33%
500-4999	4	50%	2	67%
5000+	2	25%	0	
Duration of follow-up				
< 10 y	4	50%	0	
10-14 y	1	13%	3	100%
15+ y	3	38%	0	
Mean baseline BMI (kg/m^2)				
< 24	1	13%	2	67%
24-26	4	50%	0	
> 26	3	38%	1	33%
Number/timing of beverage assessments				
Once at baseline (study length range from 7 to 16 years)	4	50%	3	100%
Twice (6 year intervals)	1	13%	0	
Every 4 years	3	38%	0	
Proportion of study participants reporting ≥ 1 serving/day				
10% or fewer or low consumption	2	25%	3	100%
Between 11-15%	3	38%	0	
More than 15%	2	25%	0	
Not reported	1	13%	0	
Method of T2D diagnosis				
Self-report with validation	4	50%	2	67%
Direct measurement/medical records	4	50%	1	33%
**Operation of study variable in multivariable models: highest versus lowest category of consumption**				
Highest consumption category:				
2+ drinks or cups/day[ARIC,BWHS]	2	25%	0	
1+ glasses or servings/day	6	75%	1	33%
< 1 serving/day	0		2	67%
Lowest consumption category:				
Never	2	25%	1	33%
never or rarely	5	63%	2	67%
<1 cup/day[ARIC]	1	13%	0	
**Characterization of multivariable models**	**Range**	**Median**	**Range**	**Median**
No. multivariable models presented	1-8	4	2-4	3
Maximum no. co-variables in multivariable models[1]	9-17	14	14-17	15
Maximum % change in SSB-T2D risk from age-adjusted estimate	46-95%	61%	2-26%	18%

1. Covariates most frequently adjusted for in multivariable models of the 11 eligible cohorts include physical activity (11 of 11), smoking (11), energy intake (11), BMI (10), family history (9), alcohol intake (8), education (5), and diet quality score (4).

Note: SSD = sugar-sweetened beverages and SD = soft drinks.

From: Susan A. Roberts
Sent: 25 Jun 2014 14:06:36 +0000
To: Collins, Janet L. (CDC/ONDIEH/NCCDPHP)
Cc: Beate Lloyd;Ryan, Kevin A. (CDC/ONDIEH/NCCDPHP);Liburd, Leandris C. (CDC/OD/OMHHE);Karima Kendall;Galuska, Deborah A. (CDC/ONDIEH/NCCDPHP)
Subject: Re: Thank you.
Attachments: image001.jpg

Janet, thank you.
Deb, feel free to reach our to me to discuss any of this or to learn more about our current research efforts in this area.
Best wishes, Susan

On Jun 25, 2014, at 8:48 AM, "Collins, Janet L. (CDC/ONDIEH/NCCDPHP)" <jlc1@cdc.gov> wrote:

Thank you so much for these resources. I am copying our Associate Director of Science, Deb Galuska, to help us make optimal use of these resources.
Thanks!
Janet

Janet Collins, Ph.D.
Director, Division of Nutrition, Physical Activity, and Obesity
National Center for Chronic Disease Prevention and Health Promotion, CDC
770-488-6042 (w); 404-409-0522 (c); jcollins@cdc.gov

From: Susan A. Roberts [mailto:suroberts@coca-cola.com]
Sent: Tuesday, June 24, 2014 10:27 PM
To: Beate Lloyd; Collins, Janet L. (CDC/ONDIEH/NCCDPHP); Ryan, Kevin A. (CDC/ONDIEH/NCCDPHP); Liburd, Leandris C. (CDC/OD/OMHHE)
Cc: Karima Kendall
Subject: RE: Thank you.

Dear Janet, Kevin and Leandris,
I was very glad to be able to participate in the meeting by phone on Friday and also look forward to on-going discussions with you to identify areas of potential collaboration and information sharing.
In follow up to Beate's note below, please find attached two recent publications on LNCS as well as a draft manuscript on heterogeneity in meta-analyses with suggested approaches to help the non-expert better interpret the outcome statistic.

1) Low-calorie sweeteners vs water during weight loss study (Jim Hill from Univ of Colorado and Gary Foster from Temple Univ, both former presidents of The Obesity Society, were the co-PIs). As mentioned on the call, this is phase I of a two-part study. Phase 2 , the maintenance phase (additional 9 months) will be completed by end of year. Leandris, this is the paper I sent to you on Friday. I have also attached the editorial associated with the paper for an additional perspective. We plan to address many of the outstanding questions of mechanism/appetite in future studies.
2) Meta-analysis of low-calorie sweeteners and weight – the analysis is done on both RCTs and prospective population studies. The findings show a modest but

significant benefit of low-calorie sweeteners in weight loss and the effect is largely driven by the beverage studies. It also provides additional support for the view that the positive associations between diet beverages and weight in the epidemiological studies is likely the result of reverse causality.

3) The heterogeneity methods paper. This paper has been accepted in the journal Systematic Reviews. Therefore please treat as confidential until it is published.

I would be glad to discuss any of this further.

Best wishes, Susan

From: Beate Lloyd
Sent: Tuesday, June 24, 2014 6:39 PM
To: jcollins@cdc.gov; kir0@cdc.gov; Liburd, Leandris C. (CDC/OD/OMHHE)
Cc: Susan A. Roberts; Karima Kendall
Subject: Thank you.

Dear Janet,

I would like to thank you, Kevin and Leandris very much for taking the time to meet with us. It was very helpful to understand your areas of focus and where we may have mutual interests. There are clearly areas where we can work collaboratively and share insights to advance the work in prevention of obesity and inform of the consumer of choices.

We valued getting to know you and your team better and enjoyed the rich discussion relating to your main initiatives. Susan will be sharing with you further the work on the low and no calorie beverage research and will follow-up as more of the data become publically available. We can also forward the papers on the scientific method and interpretation of the epidemiological studies as per our discussion and impact of heterogeneity.

It would be helpful to have another meeting in the future to follow-up on the key discussions on methods and interventions, especially with regards to the fortification programs and grocery channels. Thank you again very much for your interest and time.

Warmest regards,

Beate

Beate B. Lloyd PhD RD LD
Senior Director Nutrition COE
Global Scientific and Regulatory Affairs
The Coca-Cola Company
P: (404) 676-2149
C: (404) 545-3141

CONFIDENTIALITY NOTICE
NOTICE: This message is intended for the use of the individual or entity to which it is addressed and may contain information that is confidential, privileged and exempt from disclosure under applicable law. If the reader of this message is not the intended recipient, you are hereby notified that any printing, copying, dissemination, distribution, disclosure or forwarding of this communication is strictly prohibited. If you have received this communication in error, please contact the sender immediately and delete it from your system. Thank You.

25 YEARS

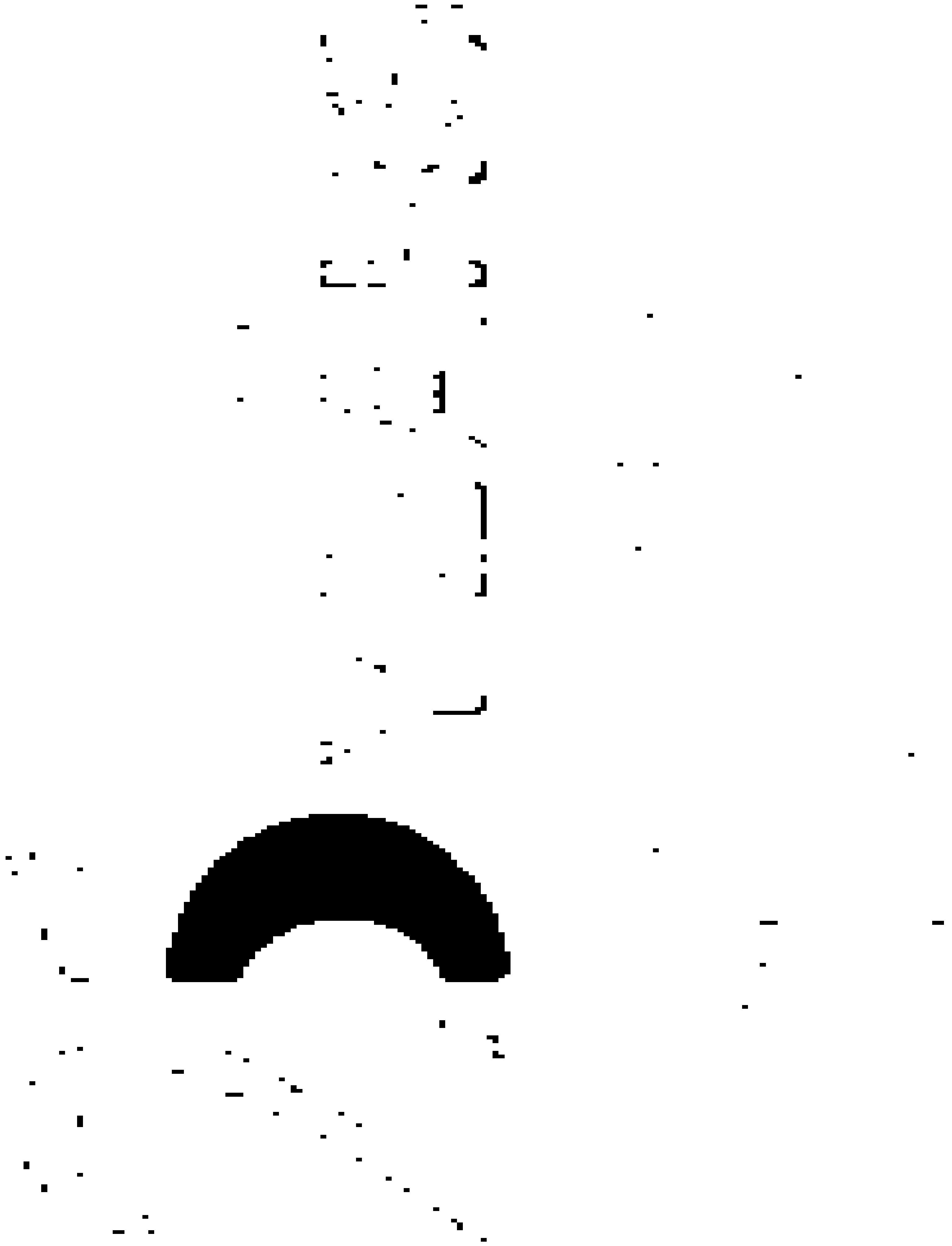

From: Beate Lloyd
Sent: 24 Jun 2014 22:38:40 +0000
To: Collins, Janet L. (CDC/ONDIEH/NCCDPHP);Ryan, Kevin A. (CDC/ONDIEH/NCCDPHP);Liburd, Leandris C. (CDC/OD/OMHHE)
Cc: Susan A. Roberts;Karima Kendall
Subject: Thank you.

Dear Janet,
I would like to thank you, Kevin and Leandris very much for taking the time to meet with us. It was very helpful to understand your areas of focus and where we may have mutual interests. There are clearly areas where we can work collaboratively and share insights to advance the work in prevention of obesity and inform of the consumer of choices.
We valued getting to know you and your team better and enjoyed the rich discussion relating to your main initiatives. Susan will be sharing with you further the work on the low and no calorie beverage research and will follow-up as more of the data become publically available. We can also forward the papers on the scientific method and interpretation of the epidemiological studies as per our discussion and impact of heterogeneity.
It would be helpful to have another meeting in the future to follow-up on the key discussions on methods and interventions, especially with regards to the fortification programs and grocery channels.
Thank you again very much for your interest and time.
Warmest regards,
Beate
Beate B. Lloyd PhD RD LD
Senior Director Nutrition COE
Global Scientific and Regulatory Affairs
The Coca-Cola Company
P: (404) 676-2149
C: (404) 545-3141
