# Supplementary material for: Public Meets Private: Conversations Between Coca‐Cola and the CDC
Source: Milbank Q. 2019 Jan 29;97(1):74–90. doi: 10.1111/1468-0009.12368 (PMC6422605; doi:10.1111/1468-0009.12368)
Supplement: Supplementary file 2 — Responses from the CDC to our FOIA request dated June 29, 2016, for all emails (including attachments) and written correspondence between Dr. Barbara A. Bowman and Alex Malaspina (January 1, 2011‐present). [file MILQ-97-74-s002.pdf]

Lucido, Sal (CDC/ONDIEH/NCCDPHP)

From: bbb8@cdc.gov
Sent: Friday, June 24, 2016 7:45 AM
To: Lucido, Sal (CDC/ONDIEH/NCCDPHP)
Subject: Fwd: Note

Sal, poor connectivity here, found one last night, attached.
Barbara

Begin forwarded message:

> **From:** "Bowman, Barbara (CDC/ONDIEH/NCCDPHP)" <bbb8@cdc.gov>
> **Date:** October 26, 2015 at 12:19:41 PM EDT
> **To:** "Goodman, Richard A. (CDC/ONDIEH/NCCDPHP)" <rag4@cdc.gov>, "Alex Malaspina (b)(6) (b)(6)
> **Cc:** Deborah Kowal <(b)(6), "Home (b)(6)" <(b)(6)>
> **Subject: RE: Note**
>
> Hi Alex, Rick, and Debbie,
>
> This is to Just share e-mail addresses/coordinates for all of us, my home e-mail is on the CC line.
>
> Again, what a lovely time we had on Saturday nights, many thanks, Alex, for your hospitality.
>
> Best wishes to all,
> Barbara
>
> **From:** Goodman, Richard A. (CDC/ONDIEH/NCCDPHP)
> **Sent:** Sunday, October 25, 2015 10:20 PM
> **To:** Bowman, Barbara (CDC/ONDIEH/NCCDPHP) <bbb8@cdc.gov>
> **Cc:** Deborah Kowal (b)(6)
> **Subject:** Note
>
> Barbara:
>
> Following links from the Chicago Tribune are Mathon's seafood restaurant in Waukegan (no longer exists) and are for you and Alex -- please pass along to him, as I don't have his e-mail address.
>
> 1967 (contains feature on search in Greece for missing arms of Venus de Milo) http://archives.chicagotribune.com/1967/06/25/page/100/article/missing-arms-of-venus-call-again-to-waukegans-mathon
>
> 1986 - http://articles.chicagotribune.com/1986-12-19/entertainment/8604050006_1_restaurant-fresh-fish-selections-statue
>
> 1994 - http://articles.chicagotribune.com/1994-04-24/features/9404240088_1_fishing-boat-commercial-fishing-planked

Rick

Lucido, Sal (CDC/ONDIEH/NCCDPHP)

From: Bowman, Barbara (CDC/ONDIEH/NCCDPHP)
Sent: Thursday, September 18, 2014 5:28 PM
To: Alex Malaspina (b)(6)
Subject: (b)(6)

Dear Alex,

(b)(6)

Barbara

Barbara Bowman

Lucido, Sal (CDC/ONDIEH/NCCDPHP)

From: Alex Malaspina <(b)(6)>
Sent: Saturday, September 20, 2014 10:08 AM
To: Bowman, Barbara (CDC/ONDIEH/NCCDPHP)
Subject: (b)(6)
Attachments:

(b)(6)

How have you been? With my warmest regards. Alex

-----Original Message-----
From: Bowman, Barbara (CDC/ONDIEH/NCCDPHP) (CDC/ONDIEH/NCCDPHP) <bbb8@cdc.gov>
To: Alex Malaspina (b)(6) <(b)(6)
Sent: Thu, Sep 18, 2014 5:27 pm
Subject: (b)(6)

Dear Alex,

(b)(6)

Barbara

Barbara Bowman

(b)(6)

(b)(6)

Lucido, Sal (CDC/ONDIEH/NCCDPHP)

From: Bowman, Barbara (CDC/ONDIEH/NCCDPHP)
Sent: Monday, September 22, 2014 4:24 PM
To: (b)(6)
Subject: (b)(6)
Attachments:

(b)(6)

B

From: Alex Malaspina [mailto (b)(6)
Sent: Saturday, September 20, 2014 10:08 AM
To: Bowman, Barbara (CDC/ONDIEH/NCCDPHP)
Subject: (b)(6)

(b)(6)
How have you been? With my warmest regards. Alex

-----Original Message-----
From: Bowman, Barbara (CDC/ONDIEH/NCCDPHP) (CDC/ONDIEH/NCCDPHP) <bbb8@cdc.gov>
To: Alex Malaspina (b)(6) (b)(6)
Sent: Thu, Sep 18, 2014 5:27 pm
Subject: (b)(6)

Dear Alex,

(b)(6)

Barbara

Barbara Bowman

Bowman, Barbara (CDC/ONDIEH/NCCDPHP)

From: Bowman, Barbara (CDC/ONDIEH/NCCDPHP)
Sent: Wednesday, September 24, 2014 12:34 PM
To: Alex Malaspina
Subject: RE: (b)(6)

Dear Alex,

I'd love to see you and to meet Wamwary Waichungo!

Generally weekends are best for me (Friday, Saturday or Sunday), anything but Monday or Tuesday usually can work too. At the moment, I don't have any travel scheduled in October, most unusual.

Looking forward to getting together.

Warmest personal regards,
Barbara

From: Alex Malaspina [mailto (b)(6)
Sent: Wednesday, September 24, 2014 6:08 AM
To: Bowman, Barbara (CDC/ONDIEH/NCCDPHP)
Subject: Re: (b)(6)

Dear Barbara:
(b)(6) I was very impressed with all you have accomplished and your new responsibilities . I always had such faith in your abilities and your great knowledge in nutrition. I would very much like to see you again and also introduce you to a very delightful and intelligent young lady from Kenya , who for the last year has had my old job at Coke, as Head of SRA. Her name is Wamwary Waichungo. How is your schedule? If you agree, give me some dates and I will arrange for a nice dinner for the three of us at a Fish Restaurant, either the "Atlanta Fish Market " or a Greek one called Kyma [means Wave in Greek} that I am sure you will like. . Warmest personal regards.
Alex

-----Original Message-----
From: Bowman, Barbara (CDC/ONDIEH/NCCDPHP) (CDC/ONDIEH/NCCDPHP) <bbb8@cdc.gov>
To: Alex Malaspina (b)(6)
Sent: Mon, Sep 22, 2014 11:27 am
Subject: RE: (b)(6)

Dear Alex,

(b)(6)

I'm doing well, am now the Director of the Division for Heart Disease and Stroke Prevention at CDC, our budget doubled this year to about $155 million. I cannot tell you how often I think of how much I learned from you at Coca-Cola, and how often I think to myself, "How would Alex handle this?"

With warmest regards (b)(6)
Barbara

From: Alex Malaspina (b)(6)
Sent: Saturday, September 20, 2014 10:08 AM

To: Bowman, Barbara (CDC/ONDIEH/NCCDPHP)
Subject: Re: [redacted] (b)(6)

[redacted] (b)(6) With my warmest regards. Alex

-----Original Message-----
From: Bowman, Barbara (CDC/ONDIEH/NCCDPHP) (CDC/ONDIEH/NCCDPHP) <bbb8@cdc.gov>
To: Alex Malaspina [redacted] (b)(6) <[redacted] (b)(6)>
Sent: Thu, Sep 18, 2014 5:27 pm
Subject: [redacted] (b)(6)

Dear Alex,

[redacted] (b)(6)

Barbara

Barbara Bowman

Lucido, Sal (CDC/ONDIEH/NCCDPHP)

From: Alex Malaspina <[redacted] (b)(6)
Sent: Wednesday, September 24, 2014 6:08 AM
To: Bowman, Barbara (CDC/ONDIEH/NCCDPHP)
Subject: Re: [redacted] (b)(6)

Dear Barbara:
[redacted] (b)(6) I was very impressed with all you have accomplished and your new responsibilities . I always had such faith in your abilities and your great knowledge in nutrition. I would very much like to see you again and also introduce you to a very delightful and intelligent young lady from Kenya , who for the last year has had my old job at Coke, as Head of SRA. Her name is Wamwary Waichungo. How is your schedule? If you agree, give me some dates and I will arrange for a nice dinner for the three of us at a Fish Restaurant, either the "Atlanta Fish Market " or a Greek one called Kyma [means Wave in Greek} that I am sure you will like. . Warmest personal regards.
Alex

-----Original Message-----
From: Bowman, Barbara (CDC/ONDIEH/NCCDPHP) (CDC/ONDIEH/NCCDPHP) <bbb8@cdc.gov>
To: Alex Malaspina [redacted] (b)(6)
Sent: Mon, Sep 22, 2014 11:27 am
Subject: RE: [redacted] (b)(6)

Dear Alex,

[redacted] (b)(6)

I'm doing well, am now the Director of the Division for Heart Disease and Stroke Prevention at CDC, our budget doubled this year to about $155 million. I cannot tell you how often I think of how much I learned from you at Coca-Cola, and how often I think to myself, "How would Alex handle this?"

With warmest regards [redacted] (b)(6)
Barbara

From: Alex Malaspina [redacted] (b)(6)
Sent: Saturday, September 20, 2014 10:08 AM
To: Bowman, Barbara (CDC/ONDIEH/NCCDPHP)
Subject: Re: [redacted] (b)(6)

Dear Barbara: [redacted] (b)(6) How have you been? With my warmest regards. Alex

-----Original Message-----
From: Bowman, Barbara (CDC/ONDIEH/NCCDPHP) (CDC/ONDIEH/NCCDPHP) <bbb8@cdc.gov>
To: Alex Malaspina [redacted] (b)(6) <[redacted] (b)(6)
Sent: Thu, Sep 18, 2014 5:27 pm
Subject: [redacted] (b)(6)

Dear Alex,

[redacted] (b)(6)

(b)(6)

Barbara

Barbara Bowman

Bowman, Barbara (CDC/ONDIEH/NCCDPHP)

From: Alex Malaspina (b)(6)
Sent: Wednesday, September 24, 2014 4:13 PM
To: Bowman, Barbara (CDC/ONDIEH/NCCDPHP)
Cc: wwaichungo@coca-cola.com
Subject: Fwd: dinner with Wamwari Waichungo

Dear Barbara : Is next Thursday , Oct. 2nd OK? if so , we could meet at the Kyma at 5;45. It gets very busy later.. Kyma is on Piedmont Three blocks or so before Peachtree, I am really looking forward to seeing you and for getting you to meet Wamwary.. Warmest personal regards. Alex

-----Original Message-----
From: Mary Stewart <marystewart@coca-cola.com>
To: malaspina (b)(6)
Cc: Wamwari Waichungo <wwaichungo@coca-cola.com>
Sent: Wed, Sep 24, 2014 3:49 pm
Subject: dinner with Wamwari Waichungo

Hello Dr. Malaspina,

Wamwari asked me to come back to you with a date for dinner. Unfortunately, she has commitments this weekend that are long standing, but she is available next Thursday evening for dinner. Please let me know if this works for you and Mrs. Bowman.

Also, I wanted you to know I have thought of you often and hope you are doing well.

Kind regards,

Mary

Mary Stewart
Global Scientific and Regulatory Affairs |The Coca-Cola Company |Office: 404.676.2014
Mobile: 404.556.6121 |Fax: 404.598.2014
e-mail: marystewart@coca-cola.com

Technical
Scientific and Regulatory Affairs

From: Wamwari Waichungo
Sent: Wednesday, September 24, 2014 3:38 PM
To: Mary Stewart
Subject: Fwd: (b)(6)

Wamwari

Begin forwarded message:

From: Alex Malaspina <>
Date: September 24, 2014 at 12:43:23 PM EDT
To: <wwaichungo@coca-cola.com>
Subject: Fwd: (b)(6)

Dear Wamwary; Barbara would love to meet you. How about this Friday evening or next Friday evening. If you are not available, we could do it on a Saturday evening. Please ley me know. Warmest regards. Alex

-----Original Message-----
From: Bowman, Barbara (CDC/ONDIEH/NCCDPHP) (CDC/ONDIEH/NCCDPHP) <bbb8@cdc.gov>
To: Alex Malaspina <(b)(6)>
Sent: Wed, Sep 24, 2014 12:33 pm
Subject: RE: (b)(6)

Dear Alex,

I'd love to see you and to meet Wamwary Waichungo!

Generally weekends are best for me (Friday, Saturday or Sunday), anything but Monday or Tuesday usually can work too. At the moment, I don't have any travel scheduled in October, most unusual.

Looking forward to getting together.

Warmest personal regards,
Barbara

From: Alex Malaspina [mailto: (b)(6)
Sent: Wednesday, September 24, 2014 6:08 AM
To: Bowman, Barbara (CDC/ONDIEH/NCCDPHP)
Subject: Re: (b)(6)

Dear Barbara:
(b)(6) I was very impressed with all you have accomplished and your new responsibilities . I always had such faith in your abilities and your great knowledge in nutrition. I would very much like to see you again and also introduce you to a very delightful and intelligent young lady from Kenya , who for the last year has had my old job at Coke, as Head of SRA. Her name is Wamwary Waichungo. How is your schedule? If you agree, give me some dates and I will arrange for a nice dinner for the three of us at a Fish Restaurant, either the "Atlanta Fish Market " or a Greek one called Kyma [means Wave in Greek} that I am sure you will like. . Warmest personal regards. Alex

-----Original Message-----
From: Bowman, Barbara (CDC/ONDIEH/NCCDPHP) (CDC/ONDIEH/NCCDPHP) <bbb8@cdc.gov>
To: Alex Malaspina <(b)(6)
Sent: Mon, Sep 22, 2014 11:27 am
Subject: RE: (b)(6)

Dear Alex,

(b)(6)

I'm doing well, am now the Director of the Division for Heart Disease and Stroke Prevention at CDC, our budget doubled this year to about $155 million. I cannot tell you how often I think of how much I learned from you at Coca-Cola, and how often I think to myself, "How would Alex handle this?"

With warmest regards and sympathy,
Barbara

From: Alex Malaspina (b)(6)
Sent: Saturday, September 20, 2014 10:08 AM
To: Bowman, Barbara (CDC/ONDIEH/NCCDPHP)
Subject: (b)(6)

Dear Barbara: (b)(6) How have you been? With my warmest regards. Alex

-----Original Message-----
From: Bowman, Barbara (CDC/ONDIEH/NCCDPHP) (CDC/ONDIEH/NCCDPHP) <bbb8@cdc.gov>
To: Alex Malaspina (b)(6) <(b)(6)
Sent: Thu, Sep 18, 2014 5:27 pm
Subject: (b)(6)

Dear Alex,

(b)(6)

Barbara

Barbara Bowman

CONFIDENTIALITY NOTICE
NOTICE: This message is intended for the use of the individual or entity to which it is addressed and may contain information that is confidential, privileged and exempt from disclosure under applicable law. If the reader of this message is not the intended recipient, you are hereby notified that any printing, copying, dissemination, distribution, disclosure or forwarding of this communication is strictly prohibited. If you have received this communication in error, please contact the sender immediately and delete it from your system. Thank You.

Lucido, Sal (CDC/ONDIEH/NCCDPHP)

**From:**	Alex Malaspina <[redacted] (b)(6)
**Sent:**	Wednesday, September 24, 2014 6:31 PM
**To:**	Bowman, Barbara (CDC/ONDIEH/NCCDPHP)
**Cc:**	wwaichungo@coca-cola.com
**Subject:**	Re: dinner with Wamwari Waichungo

Dear Barbara: I will make the reservation for three at 5:45 at Kyma for Thursday October 2nd.. Looking forward to seeing you and Wamwary again. Warmest personal regards. Alex

-----Original Message-----
From: Bowman, Barbara (CDC/ONDIEH/NCCDPHP) (CDC/ONDIEH/NCCDPHP) <bbb8@cdc.gov>
To: Alex Malaspina <[redacted] (b)(6)
Cc: wwaichungo <wwaichungo@coca-cola.com>
Sent: Wed, Sep 24, 2014 4:41 pm
Subject: RE: dinner with Wamwari Waichungo

Dear Alex and Wamwari,

That will be wonderful! I look forward to meeting you next Thursday evening at Kyma.

Best regards,
Barbara

From: Alex Malaspina [mailto:[redacted] (b)(6)
Sent: Wednesday, September 24, 2014 4:13 PM
To: Bowman, Barbara (CDC/ONDIEH/NCCDPHP)
Cc: wwaichungo@coca-cola.com
Subject: Fwd: dinner with Wamwari Waichungo

Dear Barbara : Is next Thursday , Oct. 2nd OK? if so , we could meet at the Kyma at 5;45. It gets very busy later.. Kyma is on Piedmont Three blocks or so before Peachtree, I am really looking forward to seeing you and for getting you to meet Wamwary.. Warmest personal regards. Alex

-----Original Message-----
From: Mary Stewart <marystewart@coca-cola.com>
To: malaspina <[redacted] (b)(6)
Cc: Wamwari Waichungo <wwaichungo@coca-cola.com>
Sent: Wed, Sep 24, 2014 3:49 pm
Subject: dinner with Wamwari Waichungo

Hello Dr. Malaspina,

Wamwari asked me to come back to you with a date for dinner. Unfortunately, she has commitments this weekend that are long standing, but she is available next Thursday evening for dinner. Please let me know if this works for you and Mrs. Bowman.

Also, I wanted you to know I have thought of you often and hope you are doing well.

Kind regards,

Mary

Mary Stewart
Global Scientific and Regulatory Affairs |The Coca-Cola Company |Office: 404.676.2014
Mobile: 404.556.6121 |Fax: 404.598.2014
e-mail: marystewart@coca-cola.com
Coca-Cola Technical
Scientific and Regulatory Affairs
lab

From: Wamwari Waichungo
Sent: Wednesday, September 24, 2014 3:38 PM
To: Mary Stewart
Subject: (b)(6)

Wamwari

Begin forwarded message:

> **From:** Alex Malaspina <>
> **Date:** September 24, 2014 at 12:43:23 PM EDT
> **To:** <wwaichungo@coca-cola.com>
> **Subject:** (b)(6)
>
> Dear Wamwary; Barbara would love to meet you. How about this Friday evening or next Friday evening. If you are not available, we could do it on a Saturday evening. Please ley me know. Warmest regards. Alex
>
> -----Original Message-----
> From: Bowman, Barbara (CDC/ONDIEH/NCCDPHP) (CDC/ONDIEH/NCCDPHP) <bbb8@cdc.gov>
> To: Alex Malaspina (b)(6)
> Sent: Wed, Sep 24, 2014 12:33 pm
> Subject: (b)(6)
>
> Dear Alex,
>
> I'd love to see you and to meet Wamwary Waichungo!
>
> Generally weekends are best for me (Friday, Saturday or Sunday), anything but Monday or Tuesday usually can work too. At the moment, I don't have any travel scheduled in October, most unusual.
>
> Looking forward to getting together.
>
> Warmest personal regards,
> Barbara
>
> **From:** Alex Malaspina [mailto: (b)(6)
> **Sent:** Wednesday, September 24, 2014 6:08 AM
> **To:** Bowman, Barbara (CDC/ONDIEH/NCCDPHP)
> **Subject:** (b)(6)
>
> Dear Barbara:

(b)(6) I was very impressed with all you have accomplished and your new responsibilities . I always had such faith in your abilities and your great knowledge in nutrition. I would very much like to see you again and also introduce you to a very delightful and intelligent young lady from Kenya , who for the last year has had my old job at Coke, as Head of SRA. Her name is Wamwary Waichungo. How is your schedule? If you agree, give me some dates and I will arrange for a nice dinner for the three of us at a Fish Restaurant, either the "Atlanta Fish Market " or a Greek one called Kyma [means Wave in Greek} that I am sure you will like. . Warmest personal regards. Alex

-----Original Message-----
From: Bowman, Barbara (CDC/ONDIEH/NCCDPHP) (CDC/ONDIEH/NCCDPHP) <bbb8@cdc.gov>
To: Alex Malaspina (b)(6)
Sent: Mon, Sep 22, 2014 11:27 am
Subject: RE: (b)(6)

Dear Alex,

(b)(6)

I'm doing well, am now the Director of the Division for Heart Disease and Stroke Prevention at CDC, our budget doubled this year to about $155 million. I cannot tell you how often I think of how much I learned from you at Coca-Cola, and how often I think to myself, "How would Alex handle this?"

With warmest regards (b)(6)
Barbara

From: Alex Malaspina [mailto: (b)(6)
Sent: Saturday, September 20, 2014 10:08 AM
To: Bowman, Barbara (CDC/ONDIEH/NCCDPHP)
Subject: Re: (b)(6)

Dear Barbara: (b)(6) How have you been? With my warmest regards. Alex

-----Original Message-----
From: Bowman, Barbara (CDC/ONDIEH/NCCDPHP) (CDC/ONDIEH/NCCDPHP) <bbb8@cdc.gov>
To: Alex Malaspina (b)(6) <(b)(6)>
Sent: Thu, Sep 18, 2014 5:27 pm
Subject: (b)(6)

Dear Alex,

(b)(6)

Barbara

Barbara Bowman

CONFIDENTIALITY NOTICE
NOTICE: This message is intended for the use of the individual or entity to which it is addressed and may contain information that is confidential, privileged and

exempt from disclosure under applicable law. If the reader of this message is not the intended recipient, you are hereby notified that any printing, copying, dissemination, distribution, disclosure or forwarding of this communication is strictly prohibited. If you have received this communication in error, please contact the sender immediately and delete it from your system. Thank You.

Lucido, Sal (CDC/ONDIEH/NCCDPHP)

**From:**	Alex Malaspina <(b)(6)>
**Sent:**	Friday, September 26, 2014 1:53 PM
**To:**	Bowman, Barbara (CDC/ONDIEH/NCCDPHP)
**Cc:**	wwaichungo@coca-cola.com
**Subject:**	Fwd: dinner

Dear Barbara; Is this change OK ? I hope it does not inconvenience you. With my warmest personal regards. Alex

-----Original Message-----
From: Mary Stewart <marystewart@coca-cola.com>
To: malaspina <(b)(6)>
Sent: Fri, Sep 26, 2014 12:51 pm
Subject: dinner

Dear Dr. Malaspina,

Unfortunately, Dr. Waichungo has to go to Washington, DC next Thursday, can we reschedule the dinner for the following week, Thursday, October 9. I am so sorry it cannot take place on the 2nd. Please let me know if this is possible.
Kind regards,

Mary

Mary Stewart
Global Scientific and Regulatory Affairs |The Coca-Cola Company |Office: 404.676.2014
Mobile: 404.556.6121 |Fax: 404.598.2014
e-mail: marystewart@coca-cola.com
Coca-Cola Technical
Scientific and Regulatory Affairs
lab

CONFIDENTIALITY NOTICE
NOTICE: This message is intended for the use of the individual or entity to which it is addressed and may contain information that is confidential, privileged and exempt from disclosure under applicable law. If the reader of this message is not the intended recipient, you are hereby notified that any printing, copying, dissemination, distribution, disclosure or forwarding of this communication is strictly prohibited. If you have received this communication in error, please contact the sender immediately and delete it from your system. Thank You

Lucido, Sal (CDC/ONDIEH/NCCDPHP)

From: Bowman, Barbara (CDC/ONDIEH/NCCDPHP)
Sent: Friday, September 26, 2014 1:57 PM
To: Alex Malaspina
Cc: wwaichungo@coca-cola.com
Subject: RE: dinner

Dear Alex,

I'm so sorry, but the 9th would not work for me. You know, locking in a date so easily seemed too good to be true! Will hope we can find another time soon. I'm mostly out for the rest of October.

Best,
Barbara

From: Alex Malaspina [mailto:[redacted] (b)(6)
Sent: Friday, September 26, 2014 1:53 PM
To: Bowman, Barbara (CDC/ONDIEH/NCCDPHP)
Cc: wwaichungo@coca-cola.com
Subject: Fwd: dinner

Dear Barbara; Is this change OK ? I hope it does not inconvenience you. With my warmest personal regards. Alex

-----Original Message-----
From: Mary Stewart <marystewart@coca-cola.com>
To: malaspina <[redacted] (b)(6)
Sent: Fri, Sep 26, 2014 12:51 pm
Subject: dinner

Dear Dr. Malaspina,

Unfortunately, Dr. Waichungo has to go to Washington, DC next Thursday, can we reschedule the dinner for the following week, Thursday, October 9. I am so sorry it cannot take place on the 2nd. Please let me know if this is possible.
Kind regards,

Mary

Mary Stewart
Global Scientific and Regulatory Affairs |The Coca-Cola Company |Office: 404.676.2014
Mobile: 404.556.6121 |Fax: 404.598.2014
e-mail: marystewart@coca-cola.com
Coca-Cola Technical
Scientific and Regulatory Affairs
laB

CONFIDENTIALITY NOTICE
NOTICE: This message is intended for the use of the individual or entity to which it is addressed and may contain information that is confidential, privileged [illegible] exempt from disclosure under applicable law. If the reader of this message is not the intended recipient, you are hereby notified that any printing, copying, dissemination, distribution, disclosure or forwarding of this communication is strictly prohibited. If you have received this communication in error, please [illegible] the sender immediately and delete it from your system. Thank You

Lucido, Sal (CDC/ONDIEH/NCCDPHP)

From: Alex Malaspina <[redacted] (b)(6)>
Sent: Friday, September 26, 2014 2:07 PM
To: wwaichungo@coca-cola.com; marystewart@coca-cola.com
Cc: Bowman, Barbara (CDC/ONDIEH/NCCDPHP)
Subject: Fwd: dinner

Dear Wamwary and Mary: FYI. We will have to find a date in November. I am so sorry. Alex
i

-----Original Message-----
From: Bowman, Barbara (CDC/ONDIEH/NCCDPHP) (CDC/ONDIEH/NCCDPHP) <bbb8@cdc.gov>
To: Alex Malaspina <[redacted] (b)(6)
Cc: wwaichungo <wwaichungo@coca-cola.com>
Sent: Fri, Sep 26, 2014 1:57 pm
Subject: RE: dinner

Dear Alex,

I'm so sorry, but the 9th would not work for me. You know, locking in a date so easily seemed too good to be true! Will hope we can find another time soon. I'm mostly out for the rest of October.

Best,
Barbara

From: Alex Malaspina [mailto:[redacted] (b)(6)
Sent: Friday, September 26, 2014 1:53 PM
To: Bowman, Barbara (CDC/ONDIEH/NCCDPHP)
Cc: wwaichungo@coca-cola.com
Subject: Fwd: dinner

Dear Barbara; Is this change OK ? I hope it does not inconvenience you. With my warmest personal regards. Alex

-----Original Message-----
From: Mary Stewart <marystewart@coca-cola.com>
To: malaspina <[redacted] (b)(6)
Sent: Fri, Sep 26, 2014 12:51 pm
Subject: dinner

Dear Dr. Malaspina,

Unfortunately, Dr. Waichungo has to go to Washington, DC next Thursday, can we reschedule the dinner for the following week, Thursday, October 9. I am so sorry it cannot take place on the 2nd. Please let me know if this is possible.
Kind regards,

Mary

Mary Stewart
Global Scientific and Regulatory Affairs |The Coca-Cola Company |Office: 404.676.2014
Mobile: 404.556.6121 |Fax: 404.598.2014
e-mail: marystewart@coca-cola.com
Coca-Cola Technical
Scientific and Regulatory Affairs

CONFIDENTIALITY NOTICE
NOTICE: This message is intended for the use of the individual or entity to which it is addressed and may contain information that is confidential, privileged and exempt from disclosure under applicable law. If the reader of this message is not the intended recipient, you are hereby notified that any printing, copying, dissemination, distribution, disclosure or forwarding of this communication is strictly prohibited. If you have received this communication in error, please contact the sender immediately and delete it from your system. Thank You.

Lucido, Sal (CDC/ONDIEH/NCCDPHP)

From: Bowman, Barbara (CDC/ONDIEH/NCCDPHP)
Sent: Friday, September 26, 2014 2:15 PM
To: Alex Malaspina
Subject: RE:

Dear Alex, I'd love to see you next week, thanks a million.

Best,
Barbara

From: Alex Malaspina [mailto (b)(6)
Sent: Friday, September 26, 2014 2:14 PM
To: Bowman, Barbara (CDC/ONDIEH/NCCDPHP)
Subject:

Dear Barbara: How about you and I having this dinner together. It has been so long since I last saw you! Let me know. We could meet at Kyma ,same time, 5:45. Warmest personal regards. Alex

Lucido, Sal (CDC/ONDIEH/NCCDPHP)

From: Alex Malaspina <(b)(6)>
Sent: Friday, September 26, 2014 2:18 PM
To: Bowman, Barbara (CDC/ONDIEH/NCCDPHP)
Subject: Re:

This is great ! See you next Thursday. Alex

-----Original Message-----
From: Bowman, Bar[redacted]NCCDPHP) (CDC/ONDIEH/NCCDPHP) <bbb8@cdc.gov>
To: Alex Malaspina <(b)(6)>
Sent: Fri, Sep 26, 2014 2:15 pm
Subject: RE:

Dear Alex, I'd love to see you next week, thanks a million.

Best,
Barbara

From: Alex Malaspina [mailto(b)(6)
Sent: Friday, September 26, 2014 2:14 PM
To: Bowman, Barbara (CDC/ONDIEH/NCCDPHP)
Subject:

Dear Barbara: How about you and I having this dinner together. It has been so long since I last saw you! Let me know. We could meet at Kyma ,same time, 5:45. Warmest personal regards. Alex

Lucido, Sal (CDC/ONDIEH/NCCDPHP)

**From:**	Bowman, Barbara (CDC/ONDIEH/NCCDPHP)
**Sent:**	Thursday, October 02, 2014 9:08 AM
**To:**	Alex Malaspina
**Subject:**	RE: News Digest - October 2, 2014

Dear Alex,

Thank you for this, I read about it in today's paper, this is just great. I will never forget meeting Mr. Keough as he walked through the employee cafeteria one day, he definitely inspired confidence in the ranks!

I'm very much looking forward to seeing you this evening.

Best,
Barbara

From: Alex Malaspina [mailto: (b)(6)
Sent: Thursday, October 02, 2014 7:46 AM
To: Bowman, Barbara (CDC/ONDIEH/NCCDPHP)
Subject: Fwd: News Digest - October 2, 2014

Dear Barbara: I thought you might be interested In this email. I am looking forward to seeing you this evening. Warmest regards . Alex

-----Original Message-----
From: Alex Malaspina <(b)(6)>
To: (b)(6)
James.Hill <James.Hill@ucdenver.edu>; (b)(6) john.black <john.black@viu.ca>; (b)(6) sharris <sharris@ilsi.org>; (b)(6)
(b)(6)
Sent: Thu, Oct 2, 2014 7:10 am
Subject: Fwd: News Digest - October 2, 2014

Please see last article in "the News Digest" on my former boss [and mentor] at Coke , Mr. Keough's gift to Notre Dame entitled "Notre Dame to name first school in a century after ex- Coke executive Donald Keough. I am so proud at what Mr. Keough has been doing . Alex

-----Original Message-----
From: Glenn Cooper <gcooper@coca-cola.com>
To: Undisclosed recipients:;
Sent: Thu, Oct 2, 2014 5:02 am
Subject: News Digest - October 2, 2014

October 2, 2014

Profit

Coca-Cola Revises Equity Pay Plan
CNBC 'Squawk Box', October 01, 2014

Coca-Cola, bowing to pressure, amends employee equity plan
Reuters, October 01, 2014, By Devika Krishna Kumar and Anjali Athavaley

Coke Scales Back Executive Equity Compensation, Bowing To Pressure
Wall Street Journal, October 02, 2014, By Mike Esterl And Joann S. Lublin

Coca-Cola, Yielding to Criticism, Revises Its Plan for Executive Pay
New York Times, October 02, 2014, By Peter Eavis

Coca-Cola yields to investor pressure on pay
Financial Times, October 02, 2014, By Scheherazade Daneshkhu, Consumer Industries Editor

Coca-Cola to revise executive pay plan
Associated Press, October 01, 2014

Coca-Cola Trims Stock Compensation Plan After Criticism
Bloomberg News, October 01, 2014, By Craig Giammona

Buffett Gets His Wish: Coke Is Revising Executive Pay Plan
Forbes.com, October 01, 2014, By Maggie McGrath

Coca-Cola pours cold water on executive stock plan; Company yields to Warren Buffett and activist investor
Fortune.com, October 01, 2014, By Phil Wahba

Coca-Cola changes executive pay plan
Atlanta Business Chronicle online, October 01, 2014

How Will Coca-Cola (KO) Stock React Today to Its Equity Stewardship Guidelines?
TheStreet.com, October 01, 2014, By Kurumi Fukushima

Warren Buffett Will Discusss Coke's Equity Pay Plan on CNBC Thursday Morning
CNBC 'Squawk Box', October 01, 2014

Soft Drink Makers Are The Toast Of Defensive Stocks
Investor's Business Daily, October 02, 2014, By Ken Hoover

Back to top

Industry

Pepsi True, a stevia-sweetened cola, to launch on Amazon
CNBC.com, October 01, 2014, By Sara Eisen

New Pepsi Goes To Amazon
Wall Street Journal, October 02, 2014, By Tripp Mickle

Pepsi to launch soda sweetened with stevia
Associated Press, October 01, 2014

Pepsi to launch naturally sweetened soda on Amazon, bypassing stores (Pepsi True)
Reuters, October 01, 2014, By Anjali Athavaley

Cola wars are back: Pepsi takes aim at Coke with 'copycat' stevia product Pepsi True
BrandRepublic.com (UK), October 01, 2014, By Sara Spary

New Pepsi True Rolling Out on Amazon
CNBC 'Squawk on the Street', October 01, 2014

Back to top

Planet

Coca-Cola's Times Square Sign Goes Pink in Show of Support for Breast Cancer Awareness
ABC 'Good Morning America', October 01, 2014

We're Going Back To Counting Calories... And Here's Why That's A Good Thing
HuffingtonPost.com, October 02, 2014, By Hank Cardello

Coca-Cola and rivals promise health changes but are profits the real reason? Soda giants pledged to market bottled water, diet drinks and push for smaller sizes. Are they just what's good for business? (Opinion)
TheGuardian.com (UK), October 01, 2014, By Siri Srinivas

Singapore: New rules curb junk food ads aimed at kids
Straits Times (Singapore), October 01, 2014, By Kash Cheong

Back to top

People

Notre Dame to name first school in a century after ex-Coke executive Donald Keough
Atlanta Journal-Constitution, October 02, 2014, By Christopher Seward

Back to top

Full Text

Coca-Cola Revises Equity Pay Plan
CNBC 'Squawk Box', October 01, 2014

Link to broadcast segment [2:20] ... http://www.360mediawatch.com/videos/videos/48350/PayPlan_SquawkBox_CNBC_National_10-01-14_8a.wmv

- Broadcast Transcript -

Andrew Ross Sorkin, Host: Okay, we have some breaking news right now from Coca-Cola. Coca-Cola changing its pay plan which came under some scrutiny and criticism from David Winters of Wintergreen Advisors, an activist investor who said the plan would dilute shareholders too much. There's been a lot of debate about this. Of course Warren Buffett had abstained on this vote which some people was effectively a 'no' vote. Others did as well though the equity plan itself was approved by shareholders.

They, though, have come back and now changed the plan after consulting with shareholders including Warren Buffett this morning. That plan now is effectively the same plan to some degree in terms of the amount given out but here's what's changing about it. There will be a lot less shares that are going to be distributed to employees. Instead, there's going to be a lot more cash that's going to be distributed to employees.

So previously under the plan, all employees received a mix of sixty percent stock options, forty percent performance shares. Beginning in 2015, a substantial majority will receive a hundred percent performance cash so the stock options will go away for most employees at Coca-Cola. What will change is that by 2016, the mix of those that are getting shares in cash, it'll be two-thirds what are called performance shares, and one-third stock options.

Performance shares effectively means that you will get certainly like restricted shares but you will only get them over a three-year period if you hit certain benchmarks, not about what happens to the stock price but actually what happens to the performance of the company. So we will...

Rebecca Quick, Host: This looks like this is a real move for Coca-Cola. That was what David Winters had been pushing for as you mentioned Warren Buffett abstained from that vote. Didn't want to vote against Coca-Cola but clearly had some problems with that plan as well and this looks like this is a real move to address some of those concerns.

Andrew Ross Sorkin: And it really does on that dilution issue. Just to give one last point, the 2014 grants, in terms of shares outstanding, would have been

one-point-seven percent of the shares were going to go to employees. By 2015, that goes down to point-eight percent and then later on it goes down to point-four percent on average. So, we will try to reach out to David Winters and maybe Warren Buffett and see what they have to say about this this morning.

Back to top

Coca-Cola, bowing to pressure, amends employee equity plan
Reuters, October 01, 2014, By Devika Krishna Kumar and Anjali Athavaley

Coca-Cola Co, facing criticism from Warren Buffett and other investors for its outsized employee share rewards, said it had adopted new guidelines that would limit its stock compensation plan and improve transparency.

The company said its new guidelines would extend the number of years award shares will last, formalize its practice of share repurchases to minimize dilution and renew commitments to open dialogue with shareholders on compensation matters.

About 83.16 percent of shareholders voted for the company's 2014 equity plan when it came up for renewal in April, according to a Coca-Cola filing following the company's annual meeting.

However, the approval figure included a significant number of shareholders who had abstained from the vote, according to Reuters calculations.

Buffett, whose Berkshire Hathaway Inc holds 9.1 percent of Coca-Cola and is the company's biggest shareholder, was among those who abstained.

The billionaire said in an interview with Bloomberg that while he considered the plan to be "excessive" he did not vote against it out of loyalty to the company.

But it has been Wintergreen Advisers, which owns less than 1 percent of Coca-Cola on behalf of clients, which has been the most vocal critic of the company's equity plan, saying it greatly diluted the holdings of current shareholders.

"Coca-Cola has finally conceded that the equity compensation plan it put to a vote of shareholders in April was outrageously excessive and inconsistent with past plans," Wintergreen Chief Executive David Winters said in an emailed statement on Wednesday.

"No amount of backtracking by the Coca-Cola board of directors can hide the fact that we believe it tried to sneak one by shareholders in Coca-Cola's proxy materials and statements at the April shareholder meeting," he added.

Wintergreen said in September that proxy filings with the Securities and Exchange Commission showed that funds managed by State Street, Fidelity and Capital Group voted against the plan.

"It looks to be a very unique approach to tell shareholders how the shares under this equity plan will be used with a great deal of responsibility, while addressing the criticisms about the plan from Berkshire as well as the other shareholders," Frank Glassner, chief executive of Veritas Executive Compensation Consultants, told Reuters.

Under the new guidelines, Coke's compensation committee will limit the grants under the equity plan to an annual "burn rate" of no more than 0.8 percent in 2015. The burn rate refers to the number of shares granted as a percentage of outstanding shares.

The guidelines will also facilitate a shift toward performance shares and be less heavily weighted toward stock options.

"We consider the shift positive in that it means lower "automatic" payout of equity," Stifel analysts wrote in a note.

Coca-Cola said it expected shares authorized under the equity plan to last the full term of 10 years.

Coca-Cola shares closed up 0.2 percent at $42.74 in trading on the New York Stock Exchange.

Back to top

Coke Scales Back Executive Equity Compensation, Bowing To Pressure
Wall Street Journal, October 02, 2014, By Mike Esterl And Joann S. Lublin

Coca-Cola Co. is overhauling its executive-compensation plan before it goes into effect next year, scaling back controversial stock options and shifting to more cash-based performance awards.

The dramatic reversal by the world's largest beverage company--which championed the original plan at its April shareholder meeting--follows criticism from billionaire investor Warren Buffett and other Coke shareholders who called the equity plan excessive.

It also coincides with growing efforts by Chairman and Chief Executive Muhtar Kent and other board members to mollify investors impatient with Atlanta-based Coke's recent performance. Coke failed to meet its revenue growth targets last year, pulled down by weak soda sales in the U.S. and elsewhere, and could fall short again this year.

Coke said Wednesday that it isn't altering long-term incentive targets for 6,500 managers and that compensation could remain similar in dollar terms.

depending on the company's performance. But it will issue fewer shares to management, replacing equity awards with cash awards for all but about 1,000 managers, or a bit less than 1% of Coke's staff.

Annual share dilution will stay below 1% under the new plan, similar to other companies, according to Coke. Executives who receive long-term equity awards will get mostly performance-based share awards, not stock options, it added. Performance-share units are a form of stock in which individuals earn shares based on performance measures.

The shift from options to performance shares has been a growing trend in recent years, according to Mark Reilly, head of the executive compensation practice for Verisight Inc., a human-resources consultancy. Coke directors, he said, "are late to the party." Narrowing the number of executives who qualify for equity awards also makes sense because lower-level executives have less impact on share performance, he added.

Coke's equity-compensation plan passed easily at the annual shareholder meeting in April, with 83% of votes cast in favor. But "yes" votes represented just under half of shares outstanding, after including abstentions and non-votes. Two of the top five shareholders, State Street Global Advisors and Capital Group, voted against the plan.

Mr. Buffett, whose Berkshire Hathaway Inc. is Coke's largest shareholder, with a 9% stake, abstained from voting in April but called the plan "excessive" afterward. Mr. Buffett has frequently criticized pay plans that rely heavily on stock options as "lottery tickets" that often generate outsize rewards.

Mr. Buffett went public with his concerns after a smaller shareholder, David Winters, chief executive at Wintergreen Advisers LLC, began a public campaign against Coke's equity plan in March. Mr. Winters argued the plan, including issuing 340 million new shares and options over four years, could dilute shareholders by up to 16.6% and was more generous than the expiring plan approved in 2008.

Coke director Maria Elena Lagomasino, chair of the compensation committee, said in a statement Wednesday that shareholder input "has directly led" to the revised guidelines, which she said "are in line with the long-term interests of shareowners."

Coke said equity grants will have an annual "burn rate" of no more than 0.8% in 2015 and an average of 0.4% for the remainder of the 10-year plan. That compares with 1.7% so far in 2014. Burn rates refer to the number of shares granted as a percentage of shares outstanding. Two thirds of equity awards will be performance shares by 2016, up from 40% currently.

Under the revised plan, Coke would potentially issue about 200 million shares to managers over 10 years, compared with as many as 340 million over as few as four years under the April version. The company says the reworked plan translates into a potential dilution of less than 5% over the life of the plan for shareholders.

Mr. Buffett didn't respond Wednesday to requests for comment but he is pleased with the altered plan after Coke briefed him on the changes, according to a person familiar with the situation.

State Street and Capital Group declined to comment. Each of the investment firms owns between 3% and 4% of Coke's shares outstanding, according to regulatory filings.

Mr. Winters criticized Coke for moving slowly and said he still wants to see more details about changes to the plan and how they will be implemented. "At this point it's vague and it's just promises. We view this as just more red glitter in the air," said Mr. Winters. His Wintergreen fund owns 2.5 million Coke shares, or 0.06% of shares outstanding.

He said the board and management "haven't done a great job" running Coke and that Mr. Kent should personally apologize to shareholders for the original equity plan and "probably announce his retirement."

The State Board of Administration of Florida, which voted against the equity plan in April, praised the changes. "The changes to Coke's plan are very responsive to investors' prior concerns," said Michael McCauley, who oversees corporate governance issues at the fund, which owns 5.9 million Coke shares, according to regulatory filings.

There are no signs that Mr. Kent has lost the support of Coke's board. He also hasn't signaled any plans to step down anytime soon.

Mr. Kent said Coke "will continue to provide long-term incentive awards to a broad-based group of employees with performance metrics that drive line-of-sight accountability directly to business results."

http://online.wsj.com/articles/coca-cola-tweaks-executive-compensation-plan-1412170448?KEYWORDS=coca-cola

Back to top

Coca-Cola, Yielding to Criticism, Revises Its Plan for Executive Pay
New York Times, October 02, 2014, By Peter Eavis

Coca-Cola, facing sharp shareholder criticism of its executive pay, on Wednesday announced changes to the compensation plan that set off the squall.

The big question that now hangs over the company is whether the adjustments will satisfy the shareholders who had voiced their concerns over the plan, including Warren E. Buffett of Berkshire Hathaway.

Just over 83 percent of Coca-Cola's shareholders approved the so-called 2014 equity plan at its annual meeting in April. Despite that support, discontent about the plan remained strong. On Wednesday, Coca-Cola's board said that it had taken shareholders' voices into account when devising the changes.

"Share owner input on this important topic has directly led to the development of these new guidelines, which are in line with the long-term interests of share owners," Maria Elena Lagomasino, chairwoman of the board's compensation committee, said in a statement.

One criticism was that the plan issued too many new shares. Shareholders often resist the issuance of new shares because it can dilute the value of their existing stake in the company. The critics also said that the plan left out too many of the company's employees.

David J. Winters of Wintergreen Advisers, an asset management firm, spoke out fiercely against the plan when it was announced. "It's a capitulation," he said of the changes announced on Wednesday. "But there needs to be a lot more detail."

In recent months, Coca-Cola has reaped the benefits of a surprisingly successful marketing campaign that puts specific first names on Coke cans and bottles. Even so, shares in the company have risen less than the overall stock market this year. Investors are concerned that it will struggle to post solid sales when consumers appear to be moving away from sugary drinks.

At a time of such challenges, Coca-Cola's compensation was probably going to come under closer scrutiny. Mr. Winters's campaign struck a nerve. Mr. Buffett did not vote against the compensation plan at the annual meeting, choosing to abstain instead. He did, however, describe the plan as "excessive."

Mr. Buffett did not return a call on Wednesday seeking comment on whether the changes were sufficient.

Notably, Coca-Cola is not actually reducing the number of shares it intends to issue under the plan. Instead, it is substantially lengthening the period over which it expects to issue those shares. The company is going to reduce the amount of stock-based compensation that it pays out in the form of stock options.

Coca-Cola is also going to pay cash to most employees in the plan, and focus the stock-based compensation on a much smaller number of employees. Before, all 6,500 employees included in the plan were eligible for stock awards. Now, fewer than 1,000 employees will be receiving stock, Petro Kacur, a Coke spokesman, said.

But the shift to cash may draw new criticism. Some stockholders back compensation packages that include a lot of stock, believing that it aligns the interests of shareholders with those of employees. Cash awards may weaken that link, they assert.

Mr. Kacur, however, defended the shift to cash payments. "They are still designed to attract and retain world-class talent, and align their pay with performance," he said.

But Mr. Winters said that he needed more information. "Who's really getting this money and what is the criteria?" he said.

http://www.nytimes.com/2014/10/02/business/coca-cola-yielding-to-criticism-revises-its-plan-for-executive-pay.html?_r=0

Back to top

Coca-Cola yields to investor pressure on pay
Financial Times, October 02, 2014, By Scheherazade Daneshkhu, Consumer Industries Editor

Coca-Cola has yielded to activist pressure by watering down a controversial pay plan for senior executives, described as "excessive" by Warren Buffett, the US drinks company's biggest shareholder.

The Atlanta-based company said on Wednesday that it would issue fewer shares and increase transparency about equity awards. The new guidelines would "further align compensation to the long-term interests of shareholders", said Muhtar Kent, chief executive.

David Winters, chief executive of Wintergreen Advisers, who has been the most outspoken investor against the share award plan, said: "Coca-Cola has finally conceded that the equity compensation plan it put to a vote of shareholders in April was outrageously excessive and inconsistent with past plans."

The equity pay plan to award 340 million shares, worth $13 billion, to senior management garnered the required approval level from shareholders who voted at the group's annual meeting in April, despite fierce campaigning against the scheme by Mr Winters. He argued that it was overly dilutive to existing shareholders.

Mr Buffett, who holds a 9.1 per cent stake in Coke, abstained from the vote but said the plan was "excessive". He did not vote against it because he had not wished to signal disapproval of the management. "I could never vote against Coca-Cola, but I couldn't vote for the plan either," he said at the time.

Maria Elena Lagomasino, chairman of Coca-Cola's compensation committee, said the beverage group had "heard many points of view" about the plan.

"We heard positive feedback, not so positive feedback, and everything in between. We know that not all of our shareowners agree on every issue," she said in a statement on Wednesday.

"Shareowner input on this important topic has directly led to the development of these new guidelines," she said, which would lead to the issue of "substantially fewer shares for long-term equity awards overall. We will also be signficantly reducing the use of stock options."

But she said there was "no question of changing or reducing eligibility of long-term awards."

Mr Winters, who has criticised Mr Kent as being over-paid given recent performance, said the group's about-turn "calls into question the competence and leadership of the board of directors and management. Much more work has to be done to revitalise Coca-Cola and restore trust in the company."

Coca-Cola's sales and net profit fell in the second quarter as it battled flat sales of its fizzy sodas in the US, its biggest market. Consumers have been turning away from such drinks and from the artificial sweeteners used in diet drinks.

Mr Kent has vowed to return the business to growth and has been investing in new product launches, coffee, and natural sweeteners. It paid $2.2 billion to acquire a 16.7 per cent stake in Monster, the energy drink maker, in August.

http://www.ft.com/intl/cms/s/0/751d9878-498c-11e4-80fb-00144feab7de.html#axzz3Ey7oierN

Back to top

Coca-Cola to revise executive pay plan
Associated Press, October 01, 2014

Coca-Cola is revising its pay plan for executives after shareholders including Warren Buffett expressed disapproval and called it excessive.

The world's largest beverage maker says its long-term incentive program will now distribute the company's shares to a smaller group of executives, while the rest will be rewarded with cash instead. That will mean the total shares authorized to be awarded under the plan will last longer.

The Atlanta-based company also said it would increase transparency about its equity awards and formalize its practice of share repurchases to keep stockholders' stakes from being diluted.

Coca-Cola's pay plan had come under scrutiny after Wintergreen Advisers took issue with it earlier this year, calling it a "raw deal" for shareholders. Buffett also said he discussed his concerns with Coca-Cola CEO Muhtar Kent.

Buffett, whose Berkshire Hathaway is Coke's biggest shareholder, has been a longtime critic of excessive pay packages. But he said Berkshire Hathaway didn't vote against the plan at Coca-Cola's annual meeting in April because he didn't want to publicly express disapproval of the company's management.

Afterward, however, Buffett said he discussed his concerns about the pay plan with Coca-Cola CEO Muhtar Kent.

Last year, Kent was given a pay package worth $18.2 million, according to an Associated Press calculation. That was down 16 percent from the previous year because Coca-Cola failed to meet its own growth targets.

Back to top

Coca-Cola Trims Stock Compensation Plan After Criticism
Bloomberg News, October 01, 2014, By Craig Giammona

Coca-Cola Co., whose equity-compensation plan was called excessive by billionaire Warren Buffett and other investors, said it would rein in the program.

Fewer shares will be issued, and more transparency into how the program works will be provided, the Atlanta-based company said today in a statement. The number of shares granted will be no more than 0.8 percent of total outstanding stock in 2015 and an average of 0.4 percent for the remainder of the plan.

Coca-Cola investor Wintergreen Advisers LLC, run by David Winters, railed against the compensation plan this year, calling it a "raw deal" for shareholders that was too generous and diluted the stock. Warren Buffett, Coca-Cola's largest shareholder, also spoke out against the program, though only after the plan passed when he abstained from voting on it at Coca-Cola's annual meeting in April.

"Shareowner input on this important topic has directly led to the development of these new guidelines, which are in line with the long-term interests of shareowners," Maria Elena Lagomasino, chairman of Coca-Cola's compensation committee, said in today's statement.

Buffett didn't immediately respond to a request for comment sent to an assistant. Richard Mahony, a spokesman for Wintergreen who works for Bryant Park Financial Communications, had no immediate comment.

In April, Buffett said his loyalty to Coca-Cola prevented him from voting against the program, even though he didn't approve of it.

"I could never vote against Coca-Cola, but I couldn't vote for the plan either," he said in an interview at the time. "That's just the way I feel about the people there."

Wintergreen exited its stake in Buffett's Berkshire Hathaway Inc. after Buffett's abstention. A regulatory filing in August showed the firm held no shares in Omaha, Nebraska-based Berkshire at the end of June. Wintergreen had held 1.2 million Class B shares of Berkshire on March 31, when the stake was valued at about $151 million.

"As a longtime shareholder of Berkshire, Mr. Buffett's words and actions (or more aptly, inactions) regarding Coke's 2014 equity plan did not sit well with us," Winters said in an e-mailed statement in August. "We no longer felt that Warren Buffett was looking out for his shareholders' interests."

Winters had said Coca-Cola's equity plan, in addition to ones already enacted, could dilute shareholders by 16.6 percent and transfer $29.8 billion to managers. Coca-Cola had already granted long-term equity compensation to about 6,400 employees in 2013.

The investor also criticized Coca-Cola Chief Executive Officer Muhtar Kent, saying he was paid excessively despite recent underperformance.

Coke disputed Wintergreen's calculations, and Buffett said in May that he declined to join the investor's campaign in part because he didn't want to endorse assertions that were "wildly inaccurate."

Coca-Cola rose 0.2 percent to $42.73 at 10:28 a.m. in New York. The shares had risen 3.3 percent this year through yesterday, compared with a 12 percent gain for PepsiCo Inc. and a 6.7 percent advance for the Standard & Poor's 500 Index.

http://www.bloomberg.com/news/2014-10-01/coca-cola-reins-in-stock-compensation-plan-in-wake-of-criticism.html

Back to top

Buffett Gets His Wish: Coke Is Revising Executive Pay Plan

Forbes.com, October 01, 2014, By Maggie McGrath

It's been a good week for billionaire investors: on Tuesday, Carl Icahn got his way when eBay announced that it and PayPal will go their separate ways. And on Wednesday, Coca-Cola announced that it will revise the executive pay structure that Warren Buffett recently called "excessive."

Coke announced Wednesday morning that going forward, it will provide more transparency into how its equity compensation plan works, and that the plan will use fewer shares each year. According to the new guidelines, equity shares given through the plan will consist of no more than 0.8% of all outstanding stock in 2015 and 0.4% for the remaining life of the plan.

Coke also said that while it will continue to provide long-term incentive awards to a broad group of employees, those awards will shift from stock to cash: employees who are still eligible for equity awards will see a heavier weighting of performance shares and fewer stock options. By 2016, Coke says the mix will be two-thirds performance shares and one-third stock options.

"Further to the approval of the 2014 Equity Plan in April of this year, we have developed guidelines that further align compensation to the long-term interests of shareowners," Coca-Cola chairman and CEO Muhtar Kent said in a statement Wednesday morning, referencing the push-back he received from Buffett and other shareholders earlier in the year.

In April, Buffett called Coke's compensation plan "excessive" and was open about the fact that he thought it awarded too many shares of stock. At the time, he chose to abstain from a vote on the compensation plan in an attempt to "make a statement" but not go to war with the company.

Evidently, his statement got through to Coke.

"Shareowner engagement has produced positive results for our company on a variety of fronts, including on compensation matters," Maria Elena Lagomasino, the chair of Coke's compensation committee, said in a statement Wednesday. "Shareowner input on this important topic has directly led to the development of these new Guidelines, which are in line with the long-term interests of shareowners."

Following the news of Coke's revised compensation plan, shares of the beverage giant were relatively unmoved, down just 0.2% in early Wednesday trading. Year-to-date, the stock is up 4.9%.

http://www.forbes.com/sites/maggiemcgrath/2014/10/01/buffett-gets-his-wish-coke-is-revising-executive-pay-plan/

Back to top

Coca-Cola pours cold water on executive stock plan; Company yields to Warren Buffett and activist investor

Fortune.com, October 01, 2014, By Phil Wahba

Coca-Cola has given in to criticism from investors, including Warren Buffett, and is scaling back its executive equity compensation plan.

The beverage company's new guidelines for a plan already approved by shareholders at the annual meeting earlier this year mean it will issue fewer stock awards each year, addressing concerns that the plan would dilute their investments and was too generous.

Coke will also replace equity awards for lower-level executives with cash bonuses, and replace stock options with shares tied to performance as the main form of long-term equity compensation for its executives.

The change is a win for Wintergreen Advisers LLC, a firm run by activist investor David Winters, who despite a tiny stake in Coke, has loudly agitated against the stock-compensation plan, saying it was a "raw deal" for investors. Winters, whose firm holds 2.5 million Coke shares, or 0.06%, had said that Coke's plan could could dilute shareholders by up to 16.6%.

Warren Buffett's Berkshire Hathaway, Coke's largest shareholder, also criticized the plan, saying in April, it was "excessive," but abstained from voting on the plan at Coca-Cola's annual meeting in April, citing loyalty to the company.

The plan breezed through but with all the abstentions, fewer than half of Coke's outstanding shares were voted. Buffett owns 9.1% of Coke's shares.

(Wintergreen sold its shares in Berkshire in August in anger at Buffett's inaction on the Coke compensation plan. Many were surprised at Buffett's stance given that he has often slammed executive stock plans as being tantamount to giving away winning lottery tickets.)

The number of shares Coke will grant as a percentage of total outstanding stock will be no more than 0.8 percent in 2015 and an average of 0.4 percent for the remainder of the 10-year plan. (That compares to 1.4% so far in 2014, according to the Wall Street Journal.)

Coke also said it would formalize its current practice of share repurchases to lower the impact of dilution, and that it was renewing its commitments to "an open dialogue" with investors on compensation issues.

"Further to the approval of the 2014 Equity Plan in April of this year, we have developed Guidelines that further align compensation to the long-term interests of shareowners," said Muhtar Kent, Coke's CEO, in a statement.

Coke will give performance-related shares (stock given to an executive for meeting certain goals) more weight in the long term awards, moving the ratio to two-thirds shares, one-third stock options, by 2016, compared to 60% in options and 40% in performance-shares now.

http://fortune.com/2014/10/01/coca-cola-executive-compensation/

Back to top

Coca-Cola changes executive pay plan

Atlanta Business Chronicle online, October 01, 2014

The Coca-Cola Co. has changed its executive pay plan following pressure from shareholders, including billionaire investor Warren Buffett.

In a press release, Atlanta-based Coca-Cola (NYSE: KO) said it will now distribute shares to a smaller group of executives and give cash rewards to others.

"The majority of employees currently eligible for long-term awards will begin receiving long-term incentives as performance cash awards in 2015, which will continue to provide competitive incentives consistent with the company's pay-for-performance philosophy," it said.

For executives receiving shares, "the mix of equity awards will be adjusted to be more heavily weighted to performance shares and less heavily weighted to stock options. After a one-year transition, by 2016, the mix is expected to be approximately 2/3 performance shares and 1/3 stock options."

A breakdown of the new payment guidelines here.

Maria Elena Lagomasino, chair The Coca-Cola Co.'s Compensation Committee, sent a letter to shareholders to discuss the new guidelines. Her full letter follows:

-- Dear Fellow Shareowners,

"Today, the Compensation Committee of The Coca-Cola Company's Board of Directors announced Equity Stewardship Guidelines for the Company's existing 2014 Equity Plan, which was approved by shareowners at the Company's 2014 Annual Meeting in April. View Press Release and Guidelines. I want to take a moment to tell you more about this announcement.

When the existing 2014 Equity Plan was up for approval earlier this year, and even after it was approved at our Annual Meeting of Shareowners, we heard many points of view. We heard positive feedback, not so positive feedback, and everything in between. We know that not all of our shareowners agree on every issue, especially on a topic like equity compensation where views can vary greatly. The Compensation Committee thinks this kind of debate is healthy and constructive, and welcomes the views of all shareowners – after all, we represent you.

Throughout this process of reviewing the existing Equity Plan, "stewardship" was at the forefront of our minds. By approving the Equity Plan in April, our shareowners authorized the Compensation Committee to manage something precious – shares of The Coca-Cola Company. The Compensation Committee and entire Board of Directors of this great Company continue to take very seriously this responsibility. This simple concept guided our adoption of the Equity Stewardship Guidelines.

The existing Equity Plan was designed to provide significant flexibility in how and to whom long-term equity awards are made. These Guidelines are designed to be clear and simple, driven by our desire to enhance transparency with our shareowners. While the Guidelines and the announcement include some technical terms like burn rate and dilution, the key point is that, starting in 2015, we will be using substantially fewer shares for long-term equity awards overall. We will also be significantly reducing the use of stock options.

Importantly, we are not changing or reducing eligibility for long-term awards; it is important to the Compensation Committee to keep the long-term incentive program broad-based. In addition, we are not changing or increasing any of the award ranges for long-term incentive compensation.

Our Continued Commitment to You

The Compensation Committee will continue to encourage open dialogue with our shareowners about compensation. In addition, the Compensation Committee will continue to review how we use equity compensation and the mix of awards at least annually. When making determinations about compensation, we listen to shareowner feedback, and also look at market trends, how best to attract and retain world-class talent and most effectively align pay with performance. We commit to you that we will continue to approach these decisions with great care and be mindful of our stewardship role.

As always, thank you for the trust that you have placed in us to oversee the compensation programs of The Coca-Cola Company." --

Mel Lagomasino

http://www.bizjournals.com/atlanta/news/2014/10/01/coca-cola-changes-executive-pay-plan.html?page=all

Back to top

How Will Coca-Cola (KO) Stock React Today to Its Equity Stewardship Guidelines?

TheStreet.com, October 01, 2014, By Kurumi Fukushima

Shares of The Coca-Cola Company are up 0.16% to $42.73 after the beverage company announced it adopted equity stewardship guidelines for its existing 2014 equity plan this morning.

The guidelines include burn rate, transparency, share repurchases and open dialogue.

Under the existing equity plan, the annual burn rate is a maximum of 0.8% in 2015, and an average of 0.4% for the remainder of the plan.

Share repurchases under the equity stewardship guidelines are separate from its normal share repurchase program. All of the proceeds from the stock option will be used to repurchase shares, minimizing dilution.

"We will continue to provide long-term incentive awards to a broad-based group of employees with performance metrics that drive line-of-sight accountability directly to business results," said Chairman and CEO Muhtar Kent.

Separately, TheStreet Ratings team rates COCA-COLA CO as a Buy with a ratings score of A. TheStreet Ratings Team has this to say about their recommendation:

"We rate COCA-COLA CO (KO) a BUY. This is based on the convergence of positive investment measures, which should help this stock outperform the majority of stocks that we rate.

The company's strengths can be seen in multiple areas, such as its expanding profit margins, reasonable valuation levels, increase in stock price during the past year and notable return on equity. We feel these strengths outweigh the fact that the company has had generally high debt management risk by most measures that we evaluated."

Highlights from the analysis by TheStreet Ratings Team goes as follows:

* The gross profit margin for COCA-COLA CO is rather high; currently it is at 65.60%. It has increased from the same quarter the previous year. Along with this, the net profit margin of 20.63% is above that of the industry average.

* COCA-COLA CO earnings per share from the most recent quarter came in slightly below the year earlier quarter. The company has suffered a declining pattern of earnings per share over the past year. However, we anticipate this trend reversing over the coming year. During the past fiscal year, COCA-COLA CO reported lower earnings of $1.90 versus $1.96 in the prior year. This year, the market expects an improvement in earnings ($2.08 versus $1.90).

* KO, with its decline in revenue, slightly underperformed the industry average of 4.7%. Since the same quarter one year prior, revenues slightly dropped by 1.4%. The declining revenue appears to have seeped down to the company's bottom line, decreasing earnings per share.

* The change in net income from the same quarter one year ago has significantly exceeded that of the Beverages industry average, but is less than that of the S&P 500. The net income has decreased by 3.0% when compared to the same quarter one year ago, dropping from $2,676.00 million to $2,595.00 million.

http://www.thestreet.com/story/12898964/1/how-will-coca-cola-ko-stock-react-today-to-its-equity-stewardship-guidelines.html?cm_ven=RSSFeed&utm_source=feedburner&utm_medium=feed&utm_campaign=Feed%3A+tsc%2Ffeeds%2Frss%2Flatest-stories+%28TheStreet.com+Latest+Headlines%29

Back to top

Warren Buffett Will Discusss Coke's Equity Pay Plan on CNBC Thursday Morning

CNBC 'Squawk Box', October 01, 2014

Link to broadcast segment [0:48] ...
http://www.360mediawatch.com/videos/videos/48353/PayPlan_SquawkBox_CNBC_National_10-01-14_8b.wmv

- Broadcast Transcript -

Rebecca Quick, Host: Welcome back everybody. Earlier this half hour we had news out from Coca-Cola. Looks like Coca-Cola is actually taking heat from what it has heard from its investors over the course of the last several months. It is going to be cutting back on the stock options that it is handing out to its officers.

This, again, was something that David Winters had been first advocating for earlier this year back round March or some point. It was something that was defeated in a shareholder proposal at the Coke annual meeting this year but there were a number of abstentions including Warren Buffett who decided he wasn't thrilled with that plan but did not want to vote against Coca-Cola.

Programming note for you. Tomorrow [Thursday]. Warren Buffett will be joining us live right here on set. He'll be here starting at eight AM Eastern Time. We'll get the chance to talk to him about Coca-Cola, what he thinks about this move and many other things as well.

Back to top

Soft Drink Makers Are The Toast Of Defensive Stocks
Investor's Business Daily, October 02, 2014, By Ken Hoover

The stock market is having trouble, so it might be a good time to propose a toast to three dividend-paying soft-drink makers trading near highs.

Raise your glass to Coca-Cola, PepsiCo and Dr Pepper Snapple.

The strongest performer of the three is Dr Pepper. Its stock is performing better than about 90% of stocks over the past year.

The company has reported four straight quarters of double-digit earnings growth, with a 26% increase in the most recent report.

Analysts forecast a 1% drop in the next report.

On Friday, Dr Pepper will pay a 41-cent quarterly dividend to shareholders of record September 25. The dividend works out to an annualized yield of 2.6%. Dr Pepper has raised its dividend every year since Britain's Cadbury Schweppes spun it off in 2008.

It has a five-year annualized growth rate of 13% and an Earnings Stability Factor of 5 on a 0 to 99 scale, where low numbers correspond to stable growth.

PepsiCo announced Wednesday the release of a new drink called True in a green can. It contains stevia and real sugar, although less sugar than traditional Pepsi. The drink debuts exclusively on Amazon.com.

Earnings per share grew 1% in the most recent quarter, and analysts expect a 4% increase in the next report.

Pepsi paid out a 65.5-cent quarterly dividend on Tuesday. That works out to an annualized yield of 2.8%.

Pepsi has an Earnings Stability Factor of 3.

Coke has been a slower mover than Dr Pepper or Pepsi, but it has still slightly outperformed the S&P 500 over the past year. Coke shares hit a 52-week high Wednesday.

On Wednesday, Coke paid a 30.5-cent quarterly dividend, which works out to a 2.9% annualized dividend. Coke's Earnings Stability Factor is 3.

http://news.investors.com/Investing-The-Income-Investor/100114-719807-stock-market-today-soft-drink-coke-pepsi.htm?ven=rss&utm_source=feedburner&utm_medium=feed&utm_campaign=Feed%3A+InvestingRss+%28Investing+RSS%29

Back to top

Pepsi True, a stevia-sweetened cola, to launch on Amazon
CNBC.com, October 01, 2014, By Sara Eisen

Meet Pepsi True, the latest attempt by PepsiCo to sweeten soft drink sales with a midcalorie cola.

By using sugar and stevia, the new drink contains 30 percent less sugar than a regular Pepsi, but no artificial sweeteners or high-fructose corn syrup. It's also the first cola under the flagship Pepsi brand since Pepsi Next 2½ years ago, and it will be among the first colas in the U.S. to use stevia, a plant native to Paraguay, as its primary sweetener.

Rival Coca-Cola is also in the process of a gradual and targeted launch of its own reduced-calorie, stevia-sweetened Coca-Cola Life, a cola that has 35 percent fewer calories than its flagship Coke. Like True, Coca-Cola Life mixes the stevia with sugar to achieve its sweet taste, and it also sports green-hued packaging.

The stevia cola war comes as Pepsi and its peers fight a multiyear slump in U.S. soda sales and a seismic shift in consumer attitudes and tastes toward healthier and more natural beverages.

Stevia was approved by the Food and Drug Administration nearly six years ago, and since it comes from the leaves of a plant and has no calories, it has led the charge in the search for more natural alternatives to sugar. It is now being used in everything from flavored water to energy bars, candy to cookies and yogurt.

But stevia can be tricky to work with as it often comes with a bitter aftertaste, and it has taken beverage makers some time to nail down the right recipe to maintain a familiar and sweet cola taste.

Simon Lowden, chief marketing officer at Pepsi Beverages North America, said the product captures the "true taste of the cola" and hearkens back to a time when colas used real sugar.

But in a departure from the past, True will be launched in a novel way: It will be sold exclusively on Amazon.com in mid-October and Pepsi will compensate its bottlers for purchases made in their respective regions.

"It's a brand-new product proposition with a brand-new media platform, and we want to make sure the launch reflects a right-size approach," Lowden said.

Coke also is releasing its stevia cola gradually. Coca-Cola Life began to roll out in the U.S. at Fresh Market stores in the South in late August, after launching in Argentina, Chile, the U.K. and Mexico.

The slow rollout signals a cautious approach by the two beverage giants, whose previous attempts to market "midcalorie" colas in the U.S. were unsuccessful.

"While we do not believe that either of these new sweeteners/flavoring agents will be the natural, great-tasting and calorie-free 'silver bullet' that the industry has been waiting for, we believe it is possible that they will be able to drive interest, engagement and potentially sales growth because of the massive consumer/societal need to reduce sugar and enhance healthiness," Ali Dibadj, an analyst at Sanford Bernstein, said in a note last December that previewed sweetener innovations expected this year from Coke and Pepsi.

The launch follows a nine-year decline in U.S. carbonated soft drink sales. Increased concerns about health and wellness, along with research linking sodas to obesity and health problems, have driven the drop.

Making matters worse, diet sodas that use no-calorie and low-calorie artificial sweeteners like aspartame are losing fans fast. Industry newsletter Beverage Digest reported that U.S. volumes last year for Diet Coke and Diet Pepsi tumbled nearly 7 percent from the prior year, worse than the 4 percent slump in U.S. soda volumes overall.

But it remains to be seen if consumers seeking natural and healthy products will find Pepsi True and Coca-Cola Life appealing.

In a recent U.S. consumer survey, Jefferies analysts found that "health concerns, particularly around artificial sweeteners continue to plague the U.S. diet carbonated soft drinks industry."

However, 45 percent of those drinking less diet soft drinks suggested they would possibly consume more "if they were healthier" and 30 percent said they would do so "if they were made with a natural sweetener like stevia." However, 34 percent of those drinking less suggested nothing would get them to boost their consumption.

Consumers also will need to be convinced that the taste is worth the calories in these so-called midcalorie beverages.

Pepsi thinks it's achieved that. "It's taken us three years to get to a place we feel good about," Lowden said about the development process. "No one is willing to give up on taste. Taste is king."

But calorie-conscious consumers have found the concept of midcalorie drinks tough to swallow in the past. In 2004, when sucralose, or Splenda, was the hot sugar alternative, Coke and Pepsi launched the short-lived brands, C2 and Pepsi Edge. Pepsi Next also is a midcalorie drink, but unlike True, it contains artificial sweeteners in the U.S. market.

"When it comes to soda, people want either the taste of a full-sugar product and are willing to accept 140 calories per can, or they want a zero-calorie solution and are open to a product that doesn't taste exactly like a full-sugar soda. There really isn't much in between," said Paddy Spence, CEO of Zevia,which makes naturally sweetened, zero- and low-calorie sodas using stevia.

Spence, formerly head of sales and marketing for Kashi, said his company adds monk fruit, another zero-calorie natural sweetener to its mix "to boost sweetness without adding aftertaste."

According to John Sicher, CEO of Beverage Digest, it's too early to tell whether stevia-based drinks will be the way forward.

"I think it will take three to five years to see how much better the [bigger] beverage companies will get working with stevia," Sicher said. "I think consumers will go for a sweetener that is natural, low and zero calories and tastes close to sugar, but the industry is not there yet."

http://www.cnbc.com/id/102046161

Back to top

New Pepsi Goes To Amazon

Wall Street Journal, October 02, 2014, By Tripp Mickle

PepsiCo Inc. is going to the Web to launch its newest soft drink, a mid-calorie soda called Pepsi True. The soda will be sold exclusively through Amazon.com Inc.

The product, slated for release in mid-October, will be made with sugar and stevia, a natural sweetener. Pepsi True will be the second big-name soft drink sold exclusively online. Coca-Cola Co. began selling Surge on Amazon last month.

Pepsi True is also copying Coke in another respect. Coke last year in Argentina introduced its own naturally-sweetened, mid-calorie soda, Coca-Cola Life, which also is sweetened with sugar and stevia. It introduced that drink to the U.S. in August.

A Pepsi spokeswoman said distributing Pepsi True through Amazon.com will give it a chance to raise consumer awareness for the product and gauge consumer response before it begins putting the new soda on shelves at convenience stores, supermarkets and retail outlets like Walmart.

"Both Coke and Pepsi are experimenting with new ways to launch beverages," said John Sicher, editor and publisher of Beverage Digest. "The bottling systems do better with more established products. If they [Coke and Pepsi] launch a product and build scale with Amazon before expanding to retail presence and it works, that's a good move."

Pepsi True and Coca-Cola Life are hitting the market at a time when sales of diet soda are declining and concerns about soft drink consumption and obesity are rising. U.S. consumption of diet soda fell 6.3% last year while regular soda fell just 1.7%, according to Beverage Digest.

The decline comes amid concern about artificial sweeteners, which have been identified as a potential cause of weight gain in recent health studies. Both Diet Coke and Diet Pepsi are sweetened with aspartame.

"There appears to be a growing set of consumers who graduate toward natural and want natural sweeteners in their beverages," Mr. Sicher said. "There's also a growing number of consumers concerned about obesity and health and wellness, so reduced caloric beverages with natural sweeteners would seem to be something that would appeal to consumers at this time."

Pepsi True is the fifth brand to carry the company's name. It will come in a 7.5-ounce cans containing 60 calories. The company plans to market it through digital advertising, primarily on Amazon.com, and sampling. Coca-Cola Life comes in 8-ounce glass bottles containing 60 calories each.

Back to top

Pepsi to launch soda sweetened with stevia

Associated Press, October 01, 2014

There's Pepsi, Diet Pepsi and now, Pepsi True, a midcalorie version of the soda made with natural sweeteners.

PepsiCo Inc. said Wednesday the latest version of its flagship soda will have 30 percent fewer calories than regular and be made with a mix of sugar and stevia, a natural sweetener with no calories.

The drink will go on sale later this month on Amazon.com before eventually being rolled out to supermarkets and other traditional outlets, according to the company. It will come in 7.5-ounce cans, each 60 calories.

As Americans keep cutting back on soda, Coca-Cola and Pepsi have been looking for ways to win back customers by addressing concerns about the high fructose corn syrup in regular soda as well as the artificial sweeteners in diet sodas.

Executives have pushed to come up with formulas with fewer calories using only natural sweeteners. But the bitter aftertaste of some natural sweeteners like stevia has made that a struggle. The solution so far has been to add at least some sugar — and, therefore, some calories.

Diet sodas, by contrast, have no calories, but use artificial sweeteners like aspartame that are seen as processed and fake.

In the meantime, Coca-Cola also is testing a version of its soda in the southeastern U.S. that's made with sugar and stevia. That drink, called Coca-Cola Life, comes in 8-ounce glass bottles and has 60 calories per bottle.

Ali Dibadj, a Bernstein analyst, said in a note Wednesday that interviews with store managers indicated sales of Coca-Cola Life so far seem "satisfactory."

It's not clear what type of demand there will be for midcalorie sodas like Pepsi True and Coca-Cola Life over the long term. Both companies have tested midcalorie versions of their flagship sodas in the past, but those were made with different sweeteners. In 2012, PepsiCo also rolled out Pepsi Next, which has about half the calories of regular Pepsi.

Pepsi Next, which is still on shelves, is made with a mix of three artificial sweeteners and high fructose corn syrup.

This product image provided by PepsiCo Inc. shows the company's new product, Pepsi True. PepsiCo Inc. says Pepsi True will have 30 percent fewer calories than the regular version and be made with a mix of sugar and stevia, a natural sweetener with no calories.

Back to top

Pepsi to launch naturally sweetened soda on Amazon, bypassing stores (Pepsi True)

Reuters, October 01, 2014, By Anjali Athavaley

PepsiCo Inc for the first time is introducing a product exclusively through Amazon.com Inc as the snack and soft drink maker aims to expand its footprint in e-commerce.

The product, a naturally sweetened soda called Pepsi True, will be available on Amazon in mid-October in 24-packs of 7.5-ounce cans, the company said. It will not be in brick-and-mortar outlets, though Pepsi said it plans to eventually sell True in grocery stores.

By introducing True through Amazon, Pepsi says it can better assess demand and gain insight into where people are buying it ahead of a wider launch. Industry insiders said the move illustrates how the food and beverage industry is expanding into non-traditional distribution channels like e-commerce as it searches for growth.

Kraft Foods Group Inc recently started selling Jell-O molds of university mascots and logos through Amazon and grocery stores. And other companies are using Amazon to test the waters when reintroducing a product. Coca-Cola Co said recently it would bring back Surge, a soda it discontinued 12 years ago, through Amazon.

"While it seems there is still demand for the product, we aren't sure how much or how quickly it will sell," a Coca-Cola spokesman said in an email. "Working with Amazon allows us to determine what the market for Surge really is."

Amazon says the consumer goods space is one of its fastest-growing categories. Last spring it introduced Prime Pantry, where its Prime members can shop for hundreds of household items including soft drinks, laundry detergent and cereal.

At Pepsi, "online selling is a relatively new endeavor," said Simon Lowden, chief marketing officer at Pepsi Beverages North America. "You should expect online commerce to be a much bigger part of our proposition going forward."

The e-commerce space comes with risks as well as opportunities. Amazon could put downward pressure on prices in the consumer packaged goods category, as it did with e-books, analysts at Sanford Bernstein said in a research note in April. And Amazon's vast array of products could make it harder for brands to stand out.

A Pepsi spokeswoman said prices for its other products sold on Amazon have been stable. The company says it decided to introduce True through Amazon after noting the success of other products such as its Starbucks Double Shot Light, Pepsi's top-selling item year-to-date in e-commerce. Pepsi sells the product through a joint venture with Starbucks Corp.

Still, e-commerce is especially tricky for soda companies because of their agreements with bottling partners, which have exclusive distribution rights in certain regions. Lowden said that although Pepsi True will not be distributed through Pepsi's bottler system, bottlers will be compensated for revenue they would have received when purchases are made through Amazon in their territories.

SOFTER SALES

Pepsi's move comes against the backdrop of nearly a decade of declining sales of carbonated soft drinks in the United States, according to the trade publication Beverage Digest, as consumers have become more health-conscious. More recently, consumers have also shifted away from diet soda because of health concerns about artificial sweeteners.

Pepsi True is a "mid-calorie" offering sweetened by sugar and stevia, a plant-based sweetener. It is aimed at people who want a naturally sweetened cola with fewer calories. A 7.5-ounce can of True contains 60 calories. That compares with 100 calories for the same-size can of regular Pepsi.

Stevia doesn't appeal to all palates, and soft drink makers have been trying to eliminate its bitter aftertaste. Pepsi said it spent three years developing True, drawing from its knowledge of stevia-sweetened colas in overseas markets and tweaking the formula for the U.S. consumer.

It is not the first company to launch soft drinks containing the sweetener in a measured way. Coke recently launched Coca-Cola Life, a sugar- and stevia-sweetened soda, in Fresh Market, a premium grocery chain, in four states. It said it plans to make the drink available in other locations across the United States by next month.

Pepsi plans to market True mainly through digital content such as videos on Amazon. It will also offer opportunities where consumers can sample the soda in certain markets.

"We recognize at the moment that Pepsi True is a niche product in our portfolio," Pepsi's Lowden said. "We want to be at the right size and the right scale to accommodate the 'mid- calorie' segment, which is still small today."

Back to top

Cola wars are back: Pepsi takes aim at Coke with 'copycat' stevia product Pepsi True
BrandRepublic.com (UK), October 01, 2014, By Sara Spary

PepsiCo has unveiled Pepsi True, a new stevia-sweetened Pepsi drink that will be available online through Amazon in the US later this month.

The food and drink giant announced today in the US it would launch the drink mid-October exclusively through Amazon ahead of a wider launch. Under the plans, it will eventually roll out to grocery stores.

The brand, Pepsi True, comes in a green coloured can, echoing Coca-Cola's Coke Life brand, which launched in September with a multimillion pound marketing campaign.

Reuters reported the drink had been designed to target the 'mid calorie segment'. The drink is set to contain 60 calories.

Brands have increasingly been using stevia in sweet products over the past few years in a bid to reduce caloric and sugar content as consumers become increasingly health conscious.

At the launch of Coke Life, marketing director Bobby Brittain told Marketing: "We know exactly who our Coke Life will appeal to. It's 20 and 30 somethings who have begun to realise they're not completely immortal and that they do have a sense of responsibility about what they consume".

Chris Arnold, creative director of agency and founder of Creative Orchestra said Pepsi True had 'copycatted' Coke Life, owing to the similar branding.

"You could say that Pepsi have 'copycatted Coke by using green branding for Pepsi True but they are both trading off the whole ethical green image and I think this further devalues green messaging," he said.

PepsiCo and Coca-Cola were contacted but had not responded at the time of writing.

http://www.brandrepublic.com/go/news/article/1315301/cola-wars-back-pepsi-takes-aim-coke-copycat-stevia-product-pepsi-true/

Back to top

New Pepsi True Rolling Out on Amazon
CNBC 'Squawk on the Street', October 01, 2014

Link to broadcast segment [3:54] ... http://www.360mediawatch.com/videos/videos/48363/PepsiTrue_SquawkStreet_CNBC_National_10-01-14_10a.wmv

- Broadcast Transcript -

Sara Eisen, Anchor: Pepsi is rolling out a new drink. It's called Pepsi True. You actually will not see this on the shelves because it's launching it

exclusively on Amazon.com in the middle of October, certainly a cautious and measured way to launch a new Pepsi.

This is the first time that Pepsi's launching a new cola under its brand in a few years since Next and it is a whole new approach because it has Stevia and real sugar. It's using real sugar to balance out what is often a bitter taste and aftertaste of Stevia but this is the first Stevia-based drink in the United States from Pepsi.

It's taken three years to develop it and says that True has thirty percent less sugar than a regular Pepsi. The bottom line here is that it has no artificial sweeteners and no high-fructose corn syrup, both of which are not in favor from consumers right now. Just to mention, the big competitor, Coca-Cola, is out with Coke Life. It's also taking a very measured approach here to the launch, only being released at the fresh markets in southern states right now after some debuts in the U.K. and Argentina. Also has Stevia in it.

And the problem here, overarching problem for the industry, is that soda sales have been in decline for the last nine years. People are increasingly worried about health, they're worried about obesity. Studies have increasingly been linking these problems to sugary drinks and also diets are now falling faster than the overall carbonated drinks category.

So beverages, Diane, here are trying to keep up, they're trying to release new drinks with alternative sweeteners like Stevia. But remember, back in 2004, Splenda was the sort of sugar alternative du Jour. Both Coke and Pepsi launched C2 and Pepsi Edge.

They didn't go anywhere and now they're trying it out with Stevia so we'll see. The key is it has to appeal to consumers. It has to taste sweet, be natural and be low calories. This can, this is the first time you're seeing it by the way, and it is green so you get the real true all-natural sense. Coca-Cola Life is also Green.

Simon Hobbs, Anchor: It also says it's sixty calories, sixty calories a can.

Sara Eisen: Which is thirty percent less overall sugar. I mean it has real sugar.

Simon Hobbs: But it depends on what your comparison is. If my comparison is a water, which is what the kids are drinking, or a Vitamin Water.

Sara Eisen: Well, this is not an alternative to water: it's an alternative to, say, Diet Pepsis or regular Pepsis for people who just want low-cal.

Carl Quintanilla, Anchor: Diet Pepsi has no calories.

Sara Eisen: Correct, but people are increasingly fed up with Aspartame, kind of the artificial sweeteners.

Simon Hobbs: We also had—did you see this study that came out in Nature two weeks ago that actually the artificial sweeteners may increase your blood sugar level. It's research from Israel because of what it does to the gut. That's a game changer for this category. They need to get people buying this and off the artificial if they're going to save the category.

David Faber, Anchor: What is Stevia?

Sara Eisen: It comes from a Paraguyan shrub.

Carl Quintanilla: You ever seen Breaking Bad?

David Faber: I have.

Carl Quintanilla: Stevia played a big role in the finale. No?

Sara Eisen: Yeah, yeah, yeah… No, no, no. It did.

Carl Quintanilla: Oh yeah.

Sara Eisen: That sort of launched it into the mainstream. It's used in all sorts of foods. It comes from this plant in South America. They use the leaves, the extract. It was approved by the FDA several years ago but the problem is the beverage makers have had trouble really finding the right mix and the right recipe.

Simon Hobbs: Producer says you need to taste it.

Sara Eisen: Alright, we got to taste it guys.

Carl Quintanilla: I've already had some!

Simon Hobbs: Was it nice?

Carl Quintanilla: I could totally get used to it.

Simon Hobbs: I usually have mine with Jameson. It's a bit early for me.

David Faber: I didn't get one, I don't know why.

Simon Hobbs: Here you are. You can have mine.

David Faber: I'm sorry; I'll take the unopened one.

Sara Eisen: I'm not a diet drinker, uh, but it's close to regular. You have to have it cold.

Carl Quintanilla: Warm soda is generally a no-brainer.

David Faber: Tastes like soda.

Sara Eisen: Will you taste this? Does it taste artificial?

Carl Quintanilla: Yes.

Sara Eisen: Does it taste like artificial sweetener to you?

Simon Hobbs: Does it to you?

Sara Eisen: You don't drink sodas?

David Faber: I don't drink sodas at all. Tastes like soda.

Sara Eisen: Anyway, first time you'll see it. Pepsi True.

Simon Hobbs: Do you want to mention Coke?

Sara Eisen: Sure. Coca-Cola. Out with a revised equity plan. Thank you for bringing this up.

Simon Hobbs: Revised pay plan for the senior management.

Sara Eisen: Exactly. They're not doing anything with the terms, in terms of who gets it and how much they get, but they are shifting away from the dilution problem and issuing less stock options to appease some of their shareholders.

Simon Hobbs: Did Warren Buffett like it?

Sara Eisen: Warren Buffett, they did talk to him during this. We'll see. We'll wait for a comment from him but they're trying to appease shareholders.

Back to top

Coca-Cola's Times Square Sign Goes Pink in Show of Support for Breast Cancer Awareness

ABC 'Good Morning America', October 01, 2014

Link to broadcast segment [1:30] ... (http://www.360mediawatch.com/videos/videos/48372/Coca Cola Times Square Goes Pink Good Morning America ABC National 10012014 0700-0900AM Length 0130.wmv)

- Broadcast Transcript -

George Stephanopoulos, Anchor: Good morning, America! Let's go back outside to Times Square. Look at all that pink everywhere here this morning in Times Square. Everyone going pink for this very big—as you said—very special day here, Robin, at GMA.

Robin Roberts, Anchor: October first, it launches the month of breast cancer awareness and we have every shade of pink covered this morning.

Ginger Zee, Weather Anchor: We do.

Robin Roberts: You should see the crew members, these big burly men wearing pink. They look great as well.

* * * * * *

Lara Spencer, Anchor: Alright, coming up on GMA everybody, you might have noticed that GMA is going pink. We have some important new research on breast cancer that you need to know right now. And Angelina Jolie's doctor is with us live.

* * * * * *

Ginger Zee: And we'd like to thank our wonderful neighbors here in Times Square for going pink with us starting with Bank of America, it's right there next to our jumbotron. And I'm going to have Sherri help me with the next couple.

Sherri, Fan in Times Square: Thank You to Aeropostale, Best Buy Theater, **Coca-Cola**, Hard Rock and Hyundai.

Unidentified Fan in Times Square #1: Thank you to LG, Lion King, M&M, Nasdaq and Oakley.

Unidentified Fan in Times Square #2: Thank you to Prudential, Reuters, Sony and Samsung.

Unidentified Fan in Times Square #3: And thank you to Toshiba, TDK, The United States Military Recruitment Station and Walgreens.

Ginger Zee: And we are celebrating everybody out here and are very grateful for all those who support us. Robin.

Robin Roberts: Well done! They could take our jobs. They're great like that.

Lara Spencer: Hold on, ladies.

Robin Roberts: Looking good, Ginger.

Back to top

We're Going Back To Counting Calories... And Here's Why That's A Good Thing

HuffingtonPost.com, October 02, 2014, By Hank Cardello

When we are talking about efforts to reduce obesity rates, the calories we buy, and consequently eat, do matter. Obesity is a supply problem: there are simply too many calories looking for too few mouths. Since the 1950s, the number of calories available for daily consumption per person has increased by a whopping 25 percent.

Who is best equipped to deal with reducing this overabundance of calories?

Certainly not the policymakers. Government programs to reduce consumption and educate consumers about healthy eating have been woefully ineffective. Since nutritional labeling on packaged goods was introduced in the early 1990s, the rate of obesity has more than doubled from 14 percent to over one-third of the adult population.

What about consumers? Shouldn't they be responsible for their health and well-being? Certainly. But repeated attempts to stick with diets and exercise regimens have proven challenging for families and individuals. Our Culture of Convenience demands food that is quick to heat and eat. More importantly, we have demonstrated an inability to say no to great tasting food at a great price.

That leaves the food companies. But unleashing the full power of the food industry to reduce America's dependence on high calorie foods and beverages demands a major shift.

That's why 16 of the largest food and beverage companies (think PepsiCo, Nestle, Coca-Cola) have committed to reducing calories as their pledge to the Healthy Weight Commitment Foundation (HWCF). The companies did a number of things to reach this goal, including developing new products, using smaller portion sizes and changing product recipes.

The result: Their success was not only counted in sales, but in reaching their goal of reducing 1.5 trillion calories from the marketplace. In fact, the HWCF member companies surpassed the goal reducing 6.4 trillion calories three years ahead of their commitment date.

In removing these calories from the marketplace, the Hudson Institute measured the sales impact of over 52,000 foods on shelves in 40,000-plus retail and grocery stores across the country, comparing sales of lower calorie foods to higher calorie foods.

A study by the Hudson Institute shows companies meeting consumer demand for lower-calorie products, with increasing availability in stores. This means selling an increased amount of lower-calorie products on display (up nine percent), and sustaining lower-calorie products on store shelves with a 14.1 percent net increase at the conclusion of the 5-year study.

As lower calorie products meet demands and drive sales growth, the real question is "what's next?" The impact of these companies' efforts is impressive. Because of the calorie cuts, we're eating 78 fewer calories per person per day.

But we can't stop cutting calories there – as individuals or industry. The beverage industry has just made a new commitment to reduce calories 20 percent per person by 2025, an effort that will help make choosing the lower calorie choice the easier choice.

http://www.huffingtonpost.com/hank-cardello/counting-calories_b_5876130.html?utm_hp_ref=business&ir=Business

Back to top

Coca-Cola and rivals promise health changes but are profits the real reason? Soda giants pledged to market bottled water, diet drinks and push for smaller sizes. Are they just what's good for business? (Opinion)

TheGuardian.com (UK), October 01, 2014, By Siri Srinivas

- Opinion -

What's in a name? Or 250 names? For Coca-Cola, it's massive profits.

Coke's dramatic turnaround in fortunes – a reverse in an 11-year slide in profits – has been a stunning development. What drove it was its global, personalized run of Coke bottles with names.

This summer, you could buy Coke cans blaring a number of international names suggested by customers, including Jake, Ali, Juan, Latoya, Maddy and

Cassandra. Other bottles announced the drinker a "BFF" or a "star". The idea was a social media and sales hit, bringing in a 2.4% increase in sales after 11 consecutive years of sales decline, according to Wells Fargo.

Gimmicks, however, don't keep global behemoths afloat forever.

Beverage companies including Coke have other plans to curry favor with consumers, including physical makeovers and new sweeteners.

At the Clinton Global Initiative last week, Coke, Pepsi and Dr Pepper pledged to manufacture smaller sizes of bottled water and diet drinks. Pepsi introduce a new diet sweetener, called Sweetmyx. The companies are also lobbying aggressively against "sin taxes" on beverages. The American Beverage Association has spent over $2m in an advertising blitz to convince people to vote "no" on such a tax that is on the ballot in Berkeley, California.

"It's unbelievable how much money is going into a small town," says Claire Wang, a professor of public health at Columbia University of the flood of money into Berkeley politics for the soda tax.

The beverage giants won't go without a fight – that much is clear. The question for consumers, however, is whether the attempts of the cola industry to redeem itself may come too late. They could even backfire.

Super-size me – or don't

The drinks companies' pledge to create smaller bottles harkens back to their glory days. Older Coke consumers will remember that the single-serve bottles were once only 6.5oz in the 1950s. For decades now, those bottles have been 12oz or 16oz.

"Now the kid size is 12," says Wang about McDonald's serving size. "The portion size has become ridiculous."

The recent pledge to sell smaller-sized drinks may seem like a reasonable solution. American consumers particularly tend to stockpile food, explains Harikesh Nair, a professor at Stanford Business School.

Selling smaller container sizes may reduce overall consumption in the long run, making it a business decision that will keep both soda businesses and health experts happy.

At the Clinton Global Initiative, Coke, Pepsi and Dr Pepper vowed not just to cut down on beverage size, but to reduce the calories Americans get from drinks by 20% over the next decade.

That sounds like a generous contribution to public health. But while nutritionists and health experts welcomed the move from soft drink companies to back away from super-sizing, they doubt it comes from real concern for growing national waistlines.

Many experts suspect that companies may be putting a positive spin on a global decline in sales of sugary drinks. The math is simple: selling lesser quantities of sugary beverages allows companies to charge higher prices for less product.

'Innovation' in sugary additives

Another headwind: consumers seem to have fallen out of love with cola, regardless of the bottle size.

"The consumption of sugary beverages worldwide, and especially the US, has declined, even before the pledge," says Wong.

Many health-conscious consumers have started to pay attention to the kinds of sweeteners in cola.

The popularity of cane-sugar based "Mexican Coke" in Los Angeles and New York is driven by a preference for more natural forms of sugar. Other Americans are showing an increased affinity for diet drinks. Coca-Cola already uses Acesulfame-K and aspartame for Diet Coke besides high fructose corn syrup in non-diet drinks.

PepsiCo's Indira Nooyi among other soda company chiefs admit that the industry relies heavily on innovation. This translates to a scramble for newer – and cheaper – flavour and sweetness enhancing chemical agents.

While many health experts say that diet drinks are considerably better than sugar- or syrup-based drinks, even diet colas are not ideal for health, many believe.

The search for the perfect cola sweetener has, however, driven advancements in biotech.

Pepsi recently announced that it would start to use Sweetmyx, a flavor enhancer in its drinks starting this year. Sweetmyx is a flavor enhancer that tricks your taste buds to register drinks as sweeter than they truly are.

The ingredient was developed by San Diego-based biotech firm Senomyx, which claimed it had obtained 'generally recognised as safe' approval from the Food and Drug Administration, meaning that it was safe to consume.

But what many don't know is that FDA issued a correction explaining that it does not regulate such additives. The safety assurance on Sweetmyx came not from the FDA, but a panel sponsored by the industry that manufactures these chemicals.

This conflict of interest has experts worried. "The people that create these flavour modifiers get to decide if they're safe," says Rebecca Solomon, director of clinical nutrition at New York's Mount Sinai Beth Israel.

She is concerned that these products may not get the same rigorous testing that standard FDA approval requires.

She says that while artificially sweetened drinks are healthier replacements for diabetics, using diet drinks to replace excess consumption of sugary drinks is bound to be harmful.

Why soda companies hate taxes

Another factor that allows prolific consumption is how these drinks are priced.

Carbonated drinks such as Coke and Pepsi are are significantly cheaper when bought in higher quantities. And so the consumer always benefits economically, if not physically, when she buys more.

"There was this chart I often cite," Wang says of how the Consumer Price Index explains price rise over several years. Fresh food and vegetables have become increasingly expensive. "Over time, soda has become cheaper," she says.

Enter the sin tax. Some cities have proposed taxes on soda that charges the producer by ounce.

The sin tax would replace the current sales tax on containers of soda. In states that tax soda, the average sales tax on the fizzy beverages was more than 5%, with a maximum of 7%, according to the Robert Wood Johnson Research Foundation.

But soda companies vehemently dislike the sin tax because it is profitable for them to sell smaller cans and bottles of soda at current prices. If prices for consumers rise due to higher taxes, sales volumes are likely to fall, denting profits again.

So opposed to the idea of a sin tax are beverage companies that the "aggressive marketing" they promised at the Clinton Global Initative is going towards political lobbying – not bottled water.

Ask Solomon what cola companies could do to promote healthier drinks, and expect a laugh in return. "What they could do that isn't a conflict of interest to their own business?" she counters.

The nutritionist believes that appropriate labelling of calories consumed and smaller portion sizing would make a genuine difference.

She also wishes there would be more honest advertising. "I think commercials need to indicate this is a treat. That this is a high-calorie beverage."

Coke alone has also increased its marketing budget overall by $1bn over the next three years.

Ultimately, Solomon said there needs to be more awareness about what it means when someone is consuming 300 calories in a single drink. A few educated consumers may understand the consequences. "But, to the majority of people buying that drink, the labeling isn't helping," Solomon said.

Nair agrees that transparent labelling is important: "That is for firms and regulation to fix," he says. But he points that consumers need to make responsible choices as well. "The health consequences are for the informed consumer [to consider and] to make a decision."

http://www.theguardian.com/money/us-money-blog/2014/oct/01/coca-cola-rivals-promise-changes-health

Back to top

Singapore: New rules curb junk food ads aimed at kids

Straits Times (Singapore), October 01, 2014, By Kash Cheong

Parents pestered for sweets, soft drinks or fast food by their children will get a helping hand to give the children a healthier diet.

New guidelines restricting advertisements of unhealthy food to children here will start in January. Promotion of sugary treats such as soft drinks and chocolate will be banned in media aimed at children aged 12 and below.

Drawn up by the health authorities and advertising and food industry representatives, the guidelines cover all child-targeted media, such as children's television channels and publications, and ads at bus stops within 50m of primary schools. They will be included in the Singapore Code of Advertising Practice administered by the Advertising Standards Authority of Singapore (ASAS).

The idea of curbing ads of unhealthy food was mooted two years ago by Health Minister Gan Kim Yong. A poll by the health authorities found that nine in 10 parents or guardians bought their children food or drink if the children asked for it after seeing an ad.

Parliamentary Secretary for Health Muhammad Faishal Ibrahim said: "Perhaps this is how we think we show love to our kids... but we must cultivate healthy dietary habits among them."

From next year, products advertised to children aged 12 and below have to meet the Common Nutrition Criteria endorsed by the Health Promotion Board, meaning they have to limit unhealthy ingredients such as sodium, sugar and saturated fat, and have more healthy ones like fibre and whole grain.

Companies must get a Nutrition Criteria Compliance Certificate and submit this to media owners before their child-targeted ads can be run. If an advertiser flouts the rules, the ASAS may ask it or media owners to pull the ad.

To determine if an ad is aimed at children, the ASAS will look at where it is placed and if advertising techniques are aimed at kids. For online ads, only children's websites with a Web address ending in ".sg" will be affected. The guidelines do not cover product packaging and ordinary in-store displays.

Countries such as Britain, Denmark and South Korea also restrict such ads.

In 2012, 14 major companies including Coca-Cola and McDonald's pledged not to advertise unhealthy food to children aged under 12.

Said Professor Ang Peng Hwa of the Wee Kim Wee School of Communication and Information: "In the short term, ad companies may lose out a little, but recipes will get reformulated and there will be a level playing field to advertise healthier products."

ASAS will hold workshops next month to help companies comply with the new guidelines.

Construction firm owner Sam Chew, 36, who has two children, said: "It's not only about what kids see in the media. Parents must also set an example and eat a healthy diet."

Back to top

Notre Dame to name first school in a century after ex-Coke executive Donald Keough

Atlanta Journal-Constitution, October 02, 2014, By Christopher Seward

The University of Notre Dame announced Wednesday it is establishing its first college or school in nearly a century and naming it for Donald Keough, former president and chief operating officer of Atlanta-based Coca-Cola Co.

The Donald R. Keough School of Global Affairs will conduct research on international development, peace, human rights and governance. The school will offer a master's degree in global affairs and support dual-degree programs and undergraduate programs.

The university said creation of the school is made possible by gifts of more than $50 million by Keough and his wife, Marilyn.

A year ago the Indiana university announced the Keoughs donated $30 million for a new international institutes building, Jenkins Hall, named for Notre Dame's president, the Rev. John Jenkins. The Keough School of Global Affairs will be housed in Jenkins Hall. The new building will also house the Keough-Naughton Institute for Irish Studies, Liu Institute for Asia and Asian Studies and other entities.

Construction is expected to begin in spring 2015 and be completed by the start of the 2017-18 academic year.

Donald Keough serves on the university's board of trustees. The Keough children are Notre Dame graduates, and one, Shayla Keough Rumely, is also a member of the board.

Keough served as president and COO of Coca-Cola from 1981 to 1993. He is currently chairman of the board of Allen & Company Inc., a New York investment banking firm.

http://www.ajc.com/news/business/notre-dame-to-name-first-school-in-a-century-after/nhZQg/

Back to top

~ Internal Use Only -- Do Not Redistribute ~

CONFIDENTIALITY NOTICE
NOTICE: This message is intended for the use of the individual or entity to which it is addressed and may contain information that is confidential, privileged and exempt from disclosure under applicable law. If the reader of this message is not the intended recipient, you are hereby notified that any printing, copying, dissemination, distribution, disclosure or forwarding of this communication is strictly prohibited. If you have received this communication in error, please contact the sender immediately and delete it from your system. Thank You.

From: Bowman, Barbara (CDC/ONDIEH/NCCDPHP)
Sent: Monday, October 06, 2014 4:34 PM
To: Alex Malaspina
Subject: RE: (b)(6)

Dear Alex,

Thanks, what an interesting resume. Is he able to relocate? Would definitely suggest that look at universities that have established programs in international relations and in China (Duke?), also business schools, seems like he'd be a great candidate for development work at a university or foundation work.

Best,
Barbara

From: Alex Malaspina [mailto: (b)(6)
Sent: Monday, October 06, 2014 7:51 AM
To: Bowman, Barbara (CDC/ONDIEH/NCCDPHP)
Subject: Fwd: (b)(6)

Dear Barbara:
(b)(6) is (b)(6) brother in- law. Any ideas where to apply for a job? The U. of Pennsylvania reorganized and he was let go. He is a very nice person. Warmest regards. Alex

-----Original Message-----
From: Alex Malaspina (b)(6)
To: sharris <sharris@ilsi.org>
Cc: (b)(6)
Sent: Mon, Oct 6, 2014 7:47 am
Subject: Fwd (b)(6)

Dear Suzie :
I received a revised CV for (b)(6) husband. It now stresses his Chinese experience and knowledge. Any ideas where to apply? I am going to write to Judy Rodin to see if The Rockefeller Foundation has any openings. Warmest regards.
Alex

-----Original Message-----
From: (b)(6)
To: Alex Malaspina (b)(6)
Sent: Sun, Oct 5, 2014 10:29 pm
Subject: (b)(6)

Alex,

As we have discussed, (b)(6) updated his resume and added more emphasis/details to his work in China. Let me know if this look better to you. Thanks again for thinking of us.

(b)(6)

Lucido, Sal (CDC/ONDIEH/NCCDPHP)

From: Bowman, Barbara (CDC/ONDIEH/NCCDPHP)
Sent: Friday, October 10, 2014 9:36 AM
To: Alex Malaspina
Subject: RE: dinner

Dear Alex,

(b)(6) mid-November and would love to get together then.

I hope you and yours are doing well.

Best,
Barbara

From: Alex Malaspina [mailto (b)(6)
Sent: Thursday, October 09, 2014 6:33 PM
To: Bowman, Barbara (CDC/ONDIEH/NCCDPHP)
Subject: Fwd: dinner

Dear Barbara:
Finally I get a response. I think that everyone at Coke Is under a lo t of Pressure. Do you have any dinner openings? Do you want to stick to the weekends:.This is fine with me . How are Thursday evenings. Kyma would not be so crow ded. Warmest Regards. Alex

-----Original Message-----
From: Mary Stewart <marystewart@coca-cola.com>
To: Alex Malaspina <(b)(6)
Sent: Thu, Oct 9, 2014 4:56 pm
Subject: RE: dinner

Hello Dr. Malaspina,
Could you get some dates that you and Barbara are available, and I will make it work?
Mary

From: Alex Malaspina [mailto (b)(6)
Sent: Saturday, September 27, 2014 12:49 PM
To: Mary Stewart
Subject: Re: dinner

Dear Mary: Why don't we let Barbara tell us what fits her, provided all these possibilities are OK with Wamwary. If the are , I will ask Barbara what is best for her. For me , all three possibilities are fine .Let me know . All the best wishes Alex

-----Original Message-----
From: Mary Stewart <marystewart@coca-cola.com>
To: Alex Malaspina <(b)(6)>
Sent: Sat, Sep 27, 2014 12:36 pm
Subject: RE: dinner

What would be your preference

Sent from my Verizon Wireless 4G LTE smartphone

-------- Original message (b)(6)
From: Alex Malaspina <(b)(6)
Date:09/27/2014 6:10 AM (GMT-05:00)
To: Mary Stewart <marystewart@coca-cola.com>
Cc: Wamwari Waichungo <wwaichungo@coca-cola.com>
Subject: Re: dinner

Dear Mary: Thursday , November 6th is not good for me. Just to be more certain , may I suggest Friday November 14th ? Barbara has indicated that Fridays ,Saturdays or Sundays, are better days for her. Maybe lunch on Saturday the 15th or lunch on the 16th, could also fit Barbara. At this late stage , I prefer to offer alternatives, as I gather that Barbara is extremely busy. Warmest personal regards. Alex

-----Original Message-----
From: Mary Stewart <marystewart@coca-cola.com>
To: Alex Malaspina <(b)(6)
Sent: Fri, Sep 26, 2014 3:25 pm
Subject: RE: dinner

Dr. Malaspina, I can offer Thursday, November 6 if this is agreeable.

Mary

From: Alex Malaspina [mailto(b)(6)
Sent: Friday, September 26, 2014 2:07 PM
To: Wamwari Waichungo; Mary Stewart
Cc: bbb8@cdc.gov
Subject: Fwd: dinner

Dear Wamwary and Mary: FYI. We will have to find a date in November. I am so sorry. Alex

-----Original Message-----
From: Bowman, Barbara (CDC/ONDIEH/NCCDPHP) (CDC/ONDIEH/NCCDPHP) <bbb8@cdc.gov>
To: Alex Malaspina <(b)(6)
Cc: wwaichungo <wwaichungo@coca-cola.com>
Sent: Fri, Sep 26, 2014 1:57 pm
Subject: RE: dinner

Dear Alex,

I'm so sorry, but the 9th would not work for me. You know, locking in a date so easily seemed too good to be true! Will hope we can find another time soon. I'm mostly out for the rest of October.

Best,
Barbara

From: Alex Malaspina [mailto(b)(6)
Sent: Friday, September 26, 2014 1:53 PM
To: Bowman, Barbara (CDC/ONDIEH/NCCDPHP)
Cc: wwaichungo@coca-cola.com
Subject: Fwd: dinner

Dear Barbara; Is this change OK ? I hope it does not inconvenience you. With my warmest personal regards. Alex

-----Original Message-----
From: Mary Stewart <marystewart@coca-cola.com>
To: malaspina <[redacted] (b)(6)>
Sent: Fri, Sep 26, 2014 12:51 pm
Subject: dinner

Dear Dr. Malaspina,

Unfortunately, Dr. Waichungo has to go to Washington, DC next Thursday, can we reschedule the dinner for the following week, Thursday, October 9. I am so sorry it cannot take place on the 2nd. Please let me know if this is possible.
Kind regards,

Mary

Mary Stewart
Global Scientific and Regulatory Affairs |The Coca-Cola Company |Office: 404.676.2014
Mobile: 404.556.6121 |Fax: 404.598.2014
e-mail: marystewart@coca-cola.com
Coca-Cola Technical
Scientific and Regulatory Affairs

CONFIDENTIALITY NOTICE
NOTICE: This message is intended for the use of the individual or entity to which it is addressed and may contain information that is confidential, privileged and exempt from disclosure under applicable law. If the reader of this message is not the intended recipient, you are hereby notified that any printing, copying, dissemination, distribution, disclosure or forwarding of this communication is strictly prohibited. If you have received this communication in error, please contact the sender immediately and delete it from your system. Thank You.

CONFIDENTIALITY NOTICE
NOTICE: This message is intended for the use of the individual or entity to which it is addressed and may contain information that is confidential, privileged and exempt from disclosure under applicable law. If the reader of this message is not the intended recipient, you are hereby notified that any printing, copying, dissemination, distribution, disclosure or forwarding of this communication is strictly prohibited. If you have received this communication in error, please contact the sender immediately and delete it from your system. Thank You.

CONFIDENTIALITY NOTICE
NOTICE: This message is intended for the use of the individual or entity to which it is addressed and may contain information that is confidential, privileged and exempt from disclosure under applicable law. If the reader of this message is not the intended recipient, you are hereby notified that any printing, copying, dissemination, distribution, disclosure or forwarding of this communication is strictly prohibited. If you have received this communication in error, please contact the sender immediately and delete it from your system. Thank You.

CONFIDENTIALITY NOTICE
NOTICE: This message is intended for the use of the individual or entity to which it is addressed and may contain information that is confidential, privileged and exempt from disclosure under applicable law. If the reader of this message is not the intended recipient, you are hereby notified that any printing, copying, dissemination, distribution, disclosure or forwarding of this communication is strictly prohibited. If you have received this communication in error, please contact the sender immediately and delete it from your system. Thank You

Lucido, Sal (CDC/ONDIEH/NCCDPHP)

From: Bowman, Barbara (CDC/ONDIEH/NCCDPHP)
Sent: Friday, October 10, 2014 10:19 AM
To: Alex Malaspina
Subject: RE: dinner

Dear Alex,

Thanks so much for your kind words, your support and encouragement. [redacted] (b)(6)

Best,
Barbara

From: Alex Malaspina [mailto [redacted] (b)(6)
Sent: Friday, October 10, 2014 9:49 AM
To: Bowman, Barbara (CDC/ONDIEH/NCCDPHP)
Subject: Re: dinner

My dear Barbara: [redacted] (b)(6) With all my very best wishes. Your friend Alex

-----Original Message-----
From: Bowman, Barbara (CDC/ONDIEH/NCCDPHP) (CDC/ONDIEH/NCCDPHP) <bbb8@cdc.gov>
To: Alex Malaspina [redacted] (b)(6)
Sent: Fri, Oct 10, 2014 9:36 am
Subject: RE: dinner

Dear Alex,

[redacted] (b)(6)
mid-November and would love to get together then.

I hope you and yours are doing well.

Best,
Barbara

From: Alex Malaspina [mailto [redacted] (b)(6)
Sent: Thursday, October 09, 2014 6:33 PM
To: Bowman, Barbara (CDC/ONDIEH/NCCDPHP)
Subject: Fwd: dinner

Dear Barbara:
Finally I get a response. I think that everyone at Coke Is under a lo t of Pressure. Do you have any dinner openings? Do you want to stick to the weekends:.This is fine with me . How are Thursday evenings. Kyma would not be so crow ded.
Warmest Regards. Alex

-----Original Message-----
From: Mary Stewart <marystewart@coca-cola.com>
To: Alex Malaspina [redacted] (b)(6)

Sent: Thu, Oct 9, 2014 4:56 pm
Subject: RE: dinner

Hello Dr. Malaspina,
Could you get some dates that you and Barbara are available, and I will make it work?
Mary

From: Alex Malaspina [mailto:[redacted] (b)(6)
Sent: Saturday, September 27, 2014 12:49 PM
To: Mary Stewart
Subject: Re: dinner

Dear Mary: Why don't we let Barbara tell us what fits her, provided all these possibilities are OK with Wamwary. If the are , I will ask Barbara what is best for her. For me , all three possibilities are fine .Let me know . All the best wishes Alex

-----Original Message-----
From: Mary Stewart <marystewart@coca-cola.com>
To: Alex Malaspina <[redacted] (b)(6)
Sent: Sat, Sep 27, 2014 12:36 pm
Subject: RE: dinner

What would be your preference

Sent from my Verizon Wireless 4G LTE smartphone

-------- Original messag[redacted] (b)(6)
From: Alex Malaspina <[redacted]
Date:09/27/2014 6:10 AM (GMT-05:00)
To: Mary Stewart <marystewart@coca-cola.com>
Cc: Wamwari Waichungo <wwaichungo@coca-cola.com>
Subject: Re: dinner

Dear Mary: Thursday , November 6th is not good for me. Just to be more certain , may I suggest Friday November 14th ? Barbara has indicated that Fridays ,Saturdays or Sundays, are better days for her. Maybe lunch on Saturday the 15th or lunch on the 16th, could also fit Barbara. At this late stage , I prefer to offer alternatives, as I gather that Barbara is extremely busy. Warmest personal regards. Alex

-----Original Message-----
From: Mary Stewart <marystewart@coca-cola.com>
To: Alex Malaspina <[redacted] (b)(6)
Sent: Fri, Sep 26, 2014 3:25 pm
Subject: RE: dinner

Dr. Malaspina, I can offer Thursday, November 6 if this is agreeable.

Mary

From: Alex Malaspina [mailto[redacted] (b)(6)
Sent: Friday, September 26, 2014 2:07 PM
To: Wamwari Waichungo; Mary Stewart
Cc: bbb8@cdc.gov
Subject: Fwd: dinner

Dear Wamwary and Mary: FYI. We will have to find a date in November. I am so sorry. Alex
i

-----Original Message-----
From: Bowman, Barbara (CDC/ONDIEH/NCCDPHP) (CDC/ONDIEH/NCCDPHP) <bbb8@cdc.gov>
To: Alex Malaspina <[redacted] (b)(6)
Cc: wwaichungo <wwaichungo@coca-cola.com>
Sent: Fri, Sep 26, 2014 1:57 pm
Subject: RE: dinner

Dear Alex,

I'm so sorry, but the 9th would not work for me. You know, locking in a date so easily seemed too good to be true! Will hope we can find another time soon. I'm mostly out for the rest of October.

Best,
Barbara

From: Alex Malaspina [mailto[redacted] (b)(6)
Sent: Friday, September 26, 2014 1:53 PM
To: Bowman, Barbara (CDC/ONDIEH/NCCDPHP)
Cc: wwaichungo@coca-cola.com
Subject: Fwd: dinner

Dear Barbara; Is this change OK ? I hope it does not inconvenience you. With my warmest personal regards. Alex

-----Original Message-----
From: Mary Stewart <marystewart@coca-cola.com>
To: malaspina <[redacted] (b)(6)
Sent: Fri, Sep 26, 2014 12:51 pm
Subject: dinner

Dear Dr. Malaspina,

Unfortunately, Dr. Waichungo has to go to Washington, DC next Thursday, can we reschedule the dinner for the following week, Thursday, October 9. I am so sorry it cannot take place on the 2nd. Please let me know if this is possible.
Kind regards,

Mary

Mary Stewart
Global Scientific and Regulatory Affairs |The Coca-Cola Company |Office: 404.676.2014
Mobile: 404.556.6121 |Fax: 404.598.2014
e-mail: marystewart@coca-cola.com
Technical
Scientific and Regulatory Affairs

CONFIDENTIALITY NOTICE
NOTICE: This message is intended for the use of the individual or entity to which it is addressed and may contain information that is confidential, privileged and exempt from disclosure under applicable law. If the reader of this message is not the intended recipient, you are hereby notified that any printing, copying, dissemination, distribution, disclosure or forwarding of this communication is strictly prohibited. If you have received this communication in error, please contact the sender immediately and delete it from your system. Thank You.

CONFIDENTIALITY NOTICE
NOTICE: This message is intended for the use of the individual or entity to which it is addressed and may contain information that is confidential, privileged and

exempt from disclosure under applicable law. If the reader of this message is not the intended recipient, you are hereby notified that any printing, copying, dissemination, distribution, disclosure or forwarding of this communication is strictly prohibited. If you have received this communication in error, please contact the sender immediately and delete it from your system. Thank You.

CONFIDENTIALITY NOTICE
NOTICE: This message is intended for the use of the individual or entity to which it is addressed and may contain information that is confidential, privileged and exempt from disclosure under applicable law. If the reader of this message is not the intended recipient, you are hereby notified that any printing, copying, dissemination, distribution, disclosure or forwarding of this communication is strictly prohibited. If you have received this communication in error, please contact the sender immediately and delete it from your system. Thank You.

CONFIDENTIALITY NOTICE
NOTICE: This message is intended for the use of the individual or entity to which it is addressed and may contain information that is confidential, privileged and exempt from disclosure under applicable law. If the reader of this message is not the intended recipient, you are hereby notified that any printing, copying, dissemination, distribution, disclosure or forwarding of this communication is strictly prohibited. If you have received this communication in error, please contact the sender immediately and delete it from your system. Thank You.

From: Alex Malaspina <(b)(6)
Sent: Wednesday, October 15, 2014 7:23 AM
To: Bowman, Barbara (CDC/ONDIEH/NCCDPHP)

Dear Barbara:
How are you? (b)(6) Please send me your news. As you predicted the E. problem is getting worse. I hope we can get a handle on this. Warmest wishes for (b)(6) Your Friend. Alex

Lucido, Sal (CDC/ONDIEH/NCCDPHP)

From: Bowman, Barbara (CDC/ONDIEH/NCCDPHP)
Sent: Monday, October 20, 2014 2:51 PM
To: Alex Malaspina
Subject: Re: Institute of Medicine Elects 70 New Members, 10 Foreign Associates
Attachments: ~WRD000.jpg; ~WRD000.jpg; image001.jpg; image001.jpg; image001.jpg; image001.jpg; image001.jpg

Thanks, Alex!!

Best,
Barbara

Sent from my iPhone

On Oct 20, 2014, at 1:38 PM, Alex Malaspina <malaspina@aol.com> wrote:

> Dear Barbara:
> [redacted] (b)(6) (b)(6) Warmest Regards. Alex
>
> -----Original Message-----
> From: Suzanne Harris <sharris@ilsi.org>
> To: Alex Malaspina [redacted] (b)(6) [redacted] (b)(6)
> Sent: Mon, Oct 20, 2014 11:00 am
> Subject: FW: Institute of Medicine Elects 70 New Members, 10 Foreign Associates
>
> Jim Hill has been elected as a member of the Institute of Medicine – a big deal.
>
> Suzie
>
> ---
>
> **From:** IOM News [mailto:iomnews-list@newsletters.nas.edu]
> **Sent:** Monday, October 20, 2014 10:39 AM
> **To:** Suzanne Harris
> **Subject:** Institute of Medicine Elects 70 New Members, 10 Foreign Associates

IOM Elects 70 New Members,10 Foreign Associates

The Institute of Medicine (IOM) today announced the names of 70 new members and 10 foreign associates during its 44th annual meeting. Election to the IOM is considered one of the highest honors in the fields of health and medicine and recognizes individuals who have demonstrated outstanding professional achievement and commitment to service.

"It is with great enthusiasm that we welcome our esteemed colleagues to the Institute of Medicine," said IOM President Victor J. Dzau. "These leaders' tremendous achievements have contributed significantly to advancing health and medicine. The expertise and knowledge they bring to the IOM will encourage and enhance its success."

New members are elected by current active members through a selective process that recognizes individuals who have made major contributions to the advancement of the medical sciences, health care, and public health. A diversity of talent among IOM's membership is assured by the Institute's charter, which stipulates that at least one-quarter of the membership is selected from outside the health professions, for example, from such fields as the law, engineering, social sciences, and the humanities. The newly elected members raise IOM's total active membership to 1,798 and the number of foreign associates to 128. With an additional 86 members holding emeritus status, IOM's total membership is 2,012.

"Read more >>

Manage Subscription Unsubscribe
[illegible]
[illegible]
[illegible] iomwww@nas.edu Privacy

<image002.jpg>

Lucido, Sal (CDC/ONDIEH/NCCDPHP)

From: Alex Malaspina <(b)(6)>
Sent: Thursday, October 23, 2014 2:01 PM
To: sharris@ilsi.org; (b)(6) Bowman, Barbara (CDC/ONDIEH/NCCDPHP); boonyee@ilsisea.org.sg; egeromel@coca-cola.com; (b)(6) (b)(6) wwaichungo@coca-cola.com
Subject: Fwd: (b)(6) photo
Attachments: (b)(6)

-----Original Message-----
From: Alex Malaspina <(b)(6)>
To: (b)(6)
Sent: Thu, Oct 23, 2014 1:53 pm
Subject: (b)(6)

-----Original Message-----
From: (b)(6)
To:
Sent: Thu, Oct 23, 2014 1:46 pm
Subject (b)(6)

(b)(6)

Lucido, Sal (CDC/ONDIEH/NCCDPHP)

From: Bowman, Barbara (CDC/ONDIEH/NCCDPHP)
Sent: Thursday, October 23, 2014 3:20 PM
To: Alex Malaspina
Subject: Re: [redacted] (b)(6)

[redacted] (b)(6)

Warm regards,
Barbara

Sent from my iPhone

On Oct 23, 2014, at 2:00 PM, Alex Malaspina <[redacted] (b)(6) wrote:

-----Original Message-----
From: Alex Malaspina <[redacted] (b)(6)
To: [redacted] (b)(6)
Sent: Thu, Oct 23, 2014 1:53 pm
Subject: [redacted] (b)(6)

-----Original Message-----
From: [redacted] (b)(6)
To: [redacted] (b)(6)
Sent: Thu, Oct 23, 2014 1:46 pm
Subject: [redacted] (b)(6)

[redacted] (b)(6)

Lucido, Sal (CDC/ONDIEH/NCCDPHP)

From: Goodman, Richard A. (CDC/ONDIEH/NCCDPHP)
Sent: Monday, October 26, 2015 12:43 PM
To: Bowman, Barbara (CDC/ONDIEH/NCCDPHP); Alex Malaspina ([redacted]
Cc: Deborah Kowal; Home [redacted]
Subject: RE: Note

Barbara → Many thanks for e-mail connecting.

Alex → Hope you enjoy the Chicago Tribune pieces on Mathon's and on his quest for the lost arms!

Rick

From: Bowman, Barbara (CDC/ONDIEH/NCCDPHP)
Sent: Monday, October 26, 2015 12:20 PM
To: Goodman, Richard A. (CDC/ONDIEH/NCCDPHP) <rag4@cdc.gov>; Alex Malaspina ([redacted] <[redacted]>
Cc: Deborah Kowal <[redacted]; Home [redacted]>
Subject: RE: Note

Hi Alex, Rick, and Debbie,

This is to Just share e-mail addresses/coordinates for all of us, my home e-mail is on the CC line.

Again, what a lovely time we had on Saturday nights, many thanks, Alex, for your hospitality.

Best wishes to all,
Barbara

From: Goodman, Richard A. (CDC/ONDIEH/NCCDPHP)
Sent: Sunday, October 25, 2015 10:20 PM
To: Bowman, Barbara (CDC/ONDIEH/NCCDPHP) <bbb8@cdc.gov>
Cc: Deborah Kowal <[redacted]
Subject: Note

Barbara:

Following links from the Chicago Tribune are Mathon's seafood restaurant in Waukegan (no longer exists) and are for you and Alex – please pass along to him, as I don't have his e-mail address.

1967 (contains feature on search in Greece for missing arms of Venus de Milo) http://archives.chicagotribune.com/1967/06/25/page/100/article/missing-arms-of-venus-call-again-to-waukegans-mathon

1986 - http://articles.chicagotribune.com/1986-12-19/entertainment/8604050006_1_restaurant-fresh-fish-selections-statue

1994 - http://articles.chicagotribune.com/1994-04-24/features/9404240088_1_fishing-boat-commercial-fishing-planked

Rick

Lucido, Sal (CDC/ONDIEH/NCCDPHP)

**From:**	Alex Malaspina <[redacted]
**Sent:**	Thursday, November 20, 2014 6:24 AM
**To:**	Bowman, Barbara (CDC/ONDIEH/NCCDPHP)
**Cc:**	wwaichungo@coca-cola.com

Dear Barbara:
[redacted] I hope you are feeling well enough to go to dinner . May be you prefer to do this after the holidays. Warmest wishes and regards. Alex

Lucido, Sal (CDC/ONDIEH/NCCDPHP)

From: Alex Malaspina <[redacted] (b)(6)>
Sent: Friday, November 21, 2014 8:23 AM
To: Bowman, Barbara (CDC/ONDIEH/NCCDPHP)
Subject: RE:

Dear Barbara:
[redacted] (b)(6)
The evenings of December 16 and 17 are OK for me. Wamwary will be on [redacted] (b)(6) If you agree, we could have dinner together and have a second dinner around January 10th, when Wamwary will be back. If we do have dinner in December. is it OK with you, if I also invite two close friends, who would like to meet you:: One is Clyde Tuggle, Senior VP in charge of Public Affairs. The other is: Ed Hays, who is in charge of Science. If you agree, I will check to see if they would be available. It will be great to see you again/ With my very warmest personal regards. Alex

-----Original Message-----
From: Bowman, Barbara (CDC/ONDIEH/NCCDPHP) (CDC/ONDIEH/NCCDPHP) <bbb8@cdc.gov>
To: Alex Malaspina <[redacted] (b)(6)>
Cc: wwaichungo <wwaichungo@coca-cola.com>
Sent: Thu, Nov 20, 2014 12:49 pm
Subject: RE:

Dear Alex,

How nice to hear from you! [redacted] (b)(6) It would be wonderful to get together, perhaps the week before Christmas if that could work for you both. I wish you a wonderful Thanksgiving and look forward to gettting together.

Very best regards,
Barbara

From: Alex Malaspina [mailto [redacted] (b)(6)]
Sent: Thursday, November 20, 2014 6:24 AM
To: Bowman, Barbara (CDC/ONDIEH/NCCDPHP)
Cc: wwaichungo@coca-cola.com
Subject:

Dear Barbara:
[redacted] (b)(6) I hope you are feeling well enough to go to dinner . May be you prefer to do this after the holidays. Warmest wishes and regards. Alex

Lucido, Sal (CDC/ONDIEH/NCCDPHP)

From: Bowman, Barbara (CDC/ONDIEH/NCCDPHP)
Sent: Friday, November 21, 2014 3:28 PM
To: Alex Malaspina
Cc: Home ((b)(6))
Subject: RE: RE:

Dear Alex,

The 17th would work for me, it will be wonderful to see you again, and of course, anyone else you'd like to include.

Please change to my home e-mail address above (b)(6) your messages will get to me a little faster. ☺

I wish you and yours a very happy Thanksgiving.

Best,
Barbara

From: Alex Malaspina [mailto (b)(6)
Sent: Friday, November 21, 2014 8:23 AM
To: Bowman, Barbara (CDC/ONDIEH/NCCDPHP)
Subject: RE:

Dear Barbara;
(b)(6)
The evenings of December 16 and 17 are OK for me. Wamwary will be (b)(6) If you agree, we could have dinner together and have a second dinner around January 10th, when Wamwary will be back. If we do have dinner in December. is it OK with you, if I also invite two close friends, who would like to meet you:: One is Clyde Tuggle, Senior VP in charge of Public Affairs. The other is: Ed Hays, who is in charge of Science. If you agree, I will check to see if they would be available. It will be great to see you again/ With my very warmest personal regards. Alex

-----Original Message-----
From: Bowman, Barbara (CDC/ONDIEH/NCCDPHP) (CDC/ONDIEH/NCCDPHP) <bbb8@cdc.gov>
To: Alex Malaspina <(b)(6)
Cc: wwaichungo <wwaichungo@coca-cola.com>
Sent: Thu, Nov 20, 2014 12:49 pm
Subject: RE:

Dear Alex,

How nice to hear from you! (b)(6) It would be wonderful to get together, perhaps the week before Christmas if that could work for you both. I wish you a wonderful Thanksgiving and look forward to gettting together.

Very best regards,
Barbara

From: Alex Malaspina [mailto: (b)(6)
Sent: Thursday, November 20, 2014 6:24 AM
To: Bowman, Barbara (CDC/ONDIEH/NCCDPHP)

Cc: wwaichungo@coca-cola.com
Subject:

Dear Barbara:
(b)(6) I hope you are feeling well enough to go to dinner . May be you prefer to do this after the holidays. Warmest wishes and regards. Alex

Lucido, Sal (CDC/ONDIEH/NCCDPHP)

**From:**	Alex Malaspina <(b)(6)>
**Sent:**	Sunday, November 23, 2014 5:46 PM
**To:**	Bowman, Barbara (CDC/ONDIEH/NCCDPHP)
**Subject:**	Fwd: Dinner

Dear Barbara:
I am not sure if my email with you new email address reached you. Please let me know. Warmest regards. Alex

-----Original Message-----
From: Alex Malaspina <(b)(6)>
To: (b)(6)
Sent: Sun, Nov 23, 2014 7:28 am
Subject: Fwd: Dinner

-----Original Message-----
From: Alex Malaspina <(b)(6)
To: ""\"Barbara Bowman (b)(6) <"Barbara Bowman> (b)(6)
Sent: Sun, Nov 23, 2014 7:25 am
Subject: Dinner

Dear Barbara:
Both Clyde and Ed are flying out on the 16th and can not be available on the 17th. It is a pity because I wanted them to meet you. Is it possible that you are free for dinner on the 15th? It will be wonderful to see you again whether it is the 15th or the 17th. Warmest personal regards. Alex

Lucido, Sal (CDC/ONDIEH/NCCDPHP)

From: Bowman, Barbara (CDC/ONDIEH/NCCDPHP)
Sent: Monday, November 24, 2014 6:56 AM
To: Alex Malaspina
Subject: Re: Dinner

Hi Alex, I did receive your message, had to check something last night. The 15th would be fine, I look forward to seeing you!
Best,
Barbara

Sent from my iPhone

On Nov 23, 2014, at 5:45 PM, Alex Malaspina <(b)(6)> wrote:

> Dear Barbara:
> I am not sure if my email with you new email address reached you. Please let me know. Warmest regards. Alex
>
> -----Original Message-----
> From: Alex Malaspina <(b)(6)>
> To: (b)(6)
> Sent: Sun, Nov 23, 2014 7:28 am
> Subject: Fwd: Dinner
>
> -----Original Message-----
> From: Alex Malaspina <(b)(6)
> To: ""\"Barbara Bowman (b)(6) <"Barbara Bowman> (b)(6)
> Sent: Sun, Nov 23, 2014 7:25 am
> Subject: Dinner
>
> Dear Barbara:
> Both Clyde and Ed are flying out on the 16th and can not be available on the 17th. It is a pity because I wanted them to meet you. Is it possible that you are free for dinner on the 15th? It will be wonderful to see you again whether it is the 15th or the 17th. Warmest personal regards. Alex

From: Bowman, Barbara (CDC/ONDIEH/NCCDPHP)
Sent: Wednesday, November 26, 2014 3:07 PM
To: Wamwari Waichungo
Cc: (b)(6)
Subject: RE: Dinner Meeting

Dear Wamwari,

Thank you very much for your kind note, I wish you safe travels and a wonderful time with friends and family over the holidays back home and a very happy new year.

At this point, January 8th looks fine. I look forward to meeting you, all best wishes for the Thanksgiving holiday.

Sincerely,
Barbara

P.S. Please change to my personal e-mail address (b)(6) your messages will arrive a little bit sooner (☺).

From: Wamwari Waichungo [mailto:wwaichungo@coca-cola.com]
Sent: Tuesday, November 25, 2014 8:22 PM
To: Bowman, Barbara (CDC/ONDIEH/NCCDPHP)
Cc: (b)(6)
Subject: RE: Dinner Meeting

Barbara,

I hope this message finds you well. I am glad to hear that (b)(6)

(b)(6) and therefore will not be in the US the week before Christmas. Perhaps we can plan to meet the in early January when I am back in Atlanta. I am free January 8th, 12th or 13th.

I very much look forward to meeting with you and also want to take this opportunity to wish you and your family a happy and safe holiday filled with abundance and memorable moments.

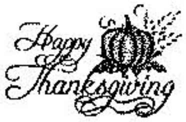

Regards,

Wamwari Waichungo, PhD
Vice President, Scientific & Regulatory Affairs
The Coca-Cola Company

From: Bowman, Barbara (CDC/ONDIEH/NCCDPHP) [mailto:bbb8@cdc.gov]
Sent: Thursday, November 20, 2014 12:50 PM
To: Alex Malaspina

Cc: Wamwari Waichungo
Subject: RE:

Dear Alex,

How nice to hear from you! [redacted] (b)(6) It would be wonderful to get together, perhaps the week before Christmas if that could work for you both. I wish you a wonderful Thanksgiving and look forward to gettting together.

Very best regards,
Barbara

From: Alex Malaspina [mailto [redacted] (b)(6)
Sent: Thursday, November 20, 2014 6:24 AM
To: Bowman, Barbara (CDC/ONDIEH/NCCDPHP)
Cc: wwaichungo@coca-cola.com
Subject:

Dear Barbara:
[redacted] (b)(6) i hope you are feeling well enough to go to dinner . May be you prefer to do this after the holidays. Warmest wishes and regards. Alex

---

CONFIDENTIALITY NOTICE
NOTICE: This message is intended for the use of the individual or entity to which it is addressed and may contain information that is confidential, privileged and exempt from disclosure under applicable law. If the reader of this message is not the intended recipient, you are hereby notified that any printing, copying dissemination, distribution, disclosure or forwarding of this communication is strictly prohibited. If you have received this communication in error, please contact the sender immediately and delete it from your system. Thank You.

Lucido, Sal (CDC/ONDIEH/NCCDPHP)

From: Alex Malaspina <(b)(6)

Sent: Tuesday, January 13, 2015 7:08 AM

To: gb.wuertzen@post.tele.dk; gchng@coca-cola.com; (b)(6) kathleen.jaynes@ucdenver.edu; (b)(6) hsteele@ilsi.org; (b)(6); (b)(6); (b)(6) (b)(6) bbrueggemeyer@ilsi.org; (b)(6) malcolmhudson@coca-cola.com; (b)(6) Jerry.j.hjelle@monsanto.com; (b)(6) Bowman, Barbara (CDC/ONDIEH/NCCDPHP); echoa@coca-cola.com; egeromel@coca-cola.com; (b)(6); (b)(6); (b)(6) david_miller@carleton.ca; dewing@coca-cola.com; (b)(6) eljefe@hgbfoundation.org

Subject: Fwd: Dr. Malaspina Video

Hi: I thought you may be interested in this video, which describes why I founded ILSI. Best regards. Alex

-----Original Message-----
From: Wamwari Waichungo <wwaichungo@coca-cola.com>
To: (b)(6)
Sent: Mon, Jan 12, 2015 12:28 pm
Subject: RE: Dr. Malaspina Video

Dr. Malapsina,

Below is a link to the video. Please confirm that you can view it.

http://cocacola.studionow.com/w/1a469e77aa3ce/

Looking forward to your feedback.

Regards,

Wamwari Waichungo, PhD
Vice President
T: (404)676-8017 M: (404)709-0274
Coca-Cola Technical Global Scientific and Regulatory Affairs

CONFIDENTIALITY NOTICE
NOTICE: This message is intended for the use of the individual or entity to which it is addressed and may contain information that is confidential, privileged and exempt from disclosure under applicable law. If the reader of this message is not the intended recipient, you are hereby notified that any printing, copying, dissemination, distribution, disclosure or forwarding of this communication is strictly prohibited. If you have received this communication in error, please contact the sender immediately and delete it from your system. Thank You

Lucido, Sal (CDC/ONDIEH/NCCDPHP)

From: Alex Malaspina <(b)(6)
Sent: Wednesday, January 14, 2015 9:24 AM
To: Bowman, Barbara (CDC/ONDIEH/NCCDPHP)
Subject: Fwd: News about Edison

Dear John : Bad News? did you know about this? Warm regards. Alex

-----Original Message-----
From: Kay T. Nichols <knichols@coca-cola.com>
To: malaspina <(b)(6)
Sent: Wed, Jan 14, 2015 8:18 am
Subject: News about Edison

Hi I'm at my desk till about 8:50
Just found out from one of (b)(6) people that he announced his retirement yesterday – at the end of April.

Regards,
Kay

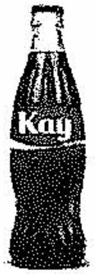

is happy to Share a Coke. with you

Kay Nichols
Associate Scientist

The Coca-Cola Company
P.O. Box 1734
Atlanta, GA [illegible]

[illegible]
T [illegible]
F [illegible]

CONFIDENTIALITY NOTICE
NOTICE: This message is intended for the use of the individual or entity to which it is addressed and may contain information that is confidential, privileged and exempt from disclosure under applicable law. If the reader of this message is not the intended recipient, you are hereby notified that any printing, copying, dissemination, distribution, disclosure or forwarding of this communication is strictly prohibited. If you have received this communication in error, please contact the sender immediately and delete it from your system. Thank You.

Lucido, Sal (CDC/ONDIEH/NCCDPHP)

From: Alex Malaspina <(b)(6)

Sent: Thursday, January 15, 2015 7:19 AM

To: Bowman, Barbara (CDC/ONDIEH/NCCDPHP) (b)(6)

Hi Julia and John:

I hope you are both well. I thought you will get a kick with some brief remarks I will make at the ILSI meeting honoring some young guest scientists. Looking forward to our luncheon some time next month. I will coordinate with John. John what do you think of (b)(6) decision to retire . I thought he was still much younger than 60.

With love and affection .

Alex

From: Bowman, Barbara (CDC/ONDIEH/NCCDPHP)
Sent: Thursday, January 15, 2015 3:11 PM
To: Home [redacted]
Subject: FW:

From: Alex Malaspina [mailto:[redacted]
Sent: Wednesday, October 15, 2014 7:23 AM
To: Bowman, Barbara (CDC/ONDIEH/NCCDPHP)
Subject:

Dear Barbara:
[redacted] As you predicted the E. problem is getting worse. I hope we can get a handle on this. Warmest wishes [redacted] Your Friend. Alex

Lucido, Sal (CDC/ONDIEH/NCCDPHP)

From: Alex Malaspina <[redacted] (b)(6)>
Sent: Monday, January 26, 2015 1:41 PM
To: Bowman, Barbara (CDC/ONDIEH/NCCDPHP)

Dear Barbara: Did you get my email about having dinner? Warm regards. Alex

Bowman, Barbara (CDC/ONDIEH/NCCDPHP)

From: Bowman, Barbara (CDC/ONDIEH/NCCDPHP)
Sent: Monday, January 26, 2015 1:58 PM
To: Alex Malaspina
Subject: RE:

Hi Alex,

I'm so sorry for the delay in my reply, unfortunately the dates just don't work for me this time, I'm in DC next week then traveling again March 2-6.

Hope you're doing well, am sure the ILSI meeting was terrific.

With best regards,
Barbara

From: Alex Malaspina [mailto [redacted] (b)(6)
Sent: Monday, January 26, 2015 1:41 PM
To: Bowman, Barbara (CDC/ONDIEH/NCCDPHP)
Subject:

Dear Barbara: Did you get my email about having dinner? Warm regards. Alex

Lucido, Sal (CDC/ONDIEH/NCCDPHP)

**From:**	Alex Malaspina <(b)(6)
**Sent:**	Friday, January 30, 2015 7:14 PM
**To:**	(b)(6)
**Subject:**	Fwd: RESEND: Thank You

Some pictures from Phoenix. The last 2 are me with some of the young awardees Alex

-----Original Message-----
From: Wamwari Waichungo <wwaichungo@coca-cola.com>
To: (b)(6)
Cc: Suzanne Harris (sharris@ilsi.org) <sharris@ilsi.org>
Sent: Wed, Jan 28, 2015 4:40 pm
Subject: RESEND: Thank You

Some files have been sent to you via the **YouSendIt** File Delivery Service.

Download the file - _MG_4219.jpg; _MG_4487.jpg; _MG_4529.JPG; _MG_4535.JPG; _MG_4343.JPG; _MG_4615.JPG

Your files will expire after 14 days.

RESEND

Dr. Malaspina,

I hope this message finds you well.

Thank you very much for the kind words. It was a privilege to host this event honoring you and your ILSI legacy. Indeed, we have received many positive comments from those who attended the event. In particular, they were very pleased to have the opportunity to spend time with you and to reflect on the ILSI journey. Thank you for being so gracious and giving us the opportunity to continue your legacy to engage emerging scientists globally.

I have attached a few pictures from the event. More to follow.

Have a great weekend.

Regards,
Wamwari

From: Alex Malaspina [mailto:(b)(6)
Sent: Thursday, January 22, 2015 5:38 PM
To: Wamwari Waichungo
Cc: Ed Hays; Clyde Tuggle; sharris@ilsi.org; Arti Arora; Mary Stewart
Subject:

Dear Wamwary; :

You arranged with grace and style for me to have a most outstanding and unforgettable evening. I cannot remember a time that I was so happy and so inspired. I am so grateful to you for initiating this event and bring it to such a successful conclusion. I was also most impressed with the young scientists that were selected. Many, many thanks again. With warmest personal regards.Alex

---

CONFIDENTIALITY NOTICE
NOTICE This message is intended for the use of the individual or entity to which it is addressed and may contain information that is confidential, privileged and exempt from disclosure under applicable law If the reader of this message is not the intended recipient, you are hereby notified that any printing copying dissemination, distribution, disclosure or forwarding of this communication is strictly prohibited If you have received this communication in error please contact the sender immediately and delete it from your system Thank You

---

Lucido, Sal (CDC/ONDIEH/NCCDPHP)

From: Alex Malaspina <(b)(6)
Sent: Monday, February 02, 2015 2:12 PM
To: (b)(6) Bowman, Barbara (CDC/ONDIEH/NCCDPHP); James.Hill@ucdenver.edu; (b)(6) (b)(6) zhangh@email.chop.edu; (b)(6)
Subject: Fwd: FYI: Coca-Cola Honors Promising Scientists

FYI. Best regards. Alex

-----Original Message-----
From: Wamwari Waichungo <wwaichungo@coca-cola.com>
To: (b)(6)
Cc: Ed Hays <ehays@coca-cola.com>; Arti Arora <artiarora@coca-cola.com>
Sent: Mon, Feb 2, 2015 1:23 pm
Subject: FYI: Coca-Cola Honors Promising Scientists

Alex,

Good afternoon. Below is a story posted on myKO.

Coca-Cola Honors Promising Scientists

Posted on: Jan 30 2015

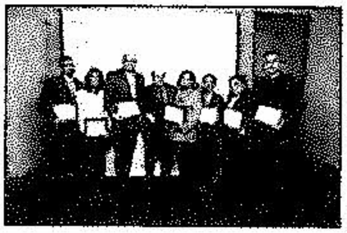

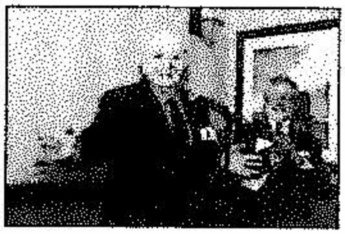

Coca-Cola has given out the first in a new series of awards for young scientists, which were established to honor the legacy of Dr. Alex Malaspina, a long-time Coca-Cola Scientific & Regulatory Affairs leader and founder of the International Life Sciences Institute (ILSI). The inaugural ILSI Malaspina International Scholars Travel Awards were presented to 10 young scientists from across the globe at the 2015 ILSI Annual Meeting in Phoenix, held Jan. 16-21.

Dr. Malaspina founded ILSI in 1978 as a nonprofit organization to advance scientific understanding of nutrition, food safety, toxicology, risk assessment and the environment. He envisioned prominent researchers from industry, government and academia would jointly lead the organization, guiding its work to conduct research, harmonize the use of science and encourage scientific dialogue.

Dr. Malaspina began ILSI to spearhead an effort to unite the food industry. Before retiring as president of ILSI in 2001, Dr. Malaspina wanted to ensure a good endowment was in place to fund a research foundation to support the ILSI mission.

Coca-Cola senior leaders were instrumental in supporting Dr. Malaspina as he established a $22 million endowment for the foundation. More than 35 years later, ILSI is still delivering on its promise. It is now a truly global organization, with 15 regional branches that help ensure food safety and nutrition science advancement in various regions around the world.

To honor Dr. Malaspina's passion for nurturing promising scientific talent, Coca-Cola established the Malaspina International Scholars Travel Award. For the inaugural awards, individual ILSI branches submitted nominations of qualified academic scientists in their regions.

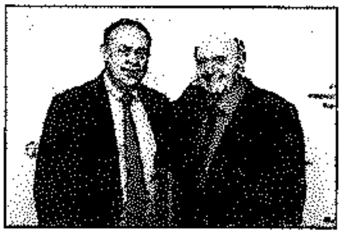

A selection committee, comprised of five members of the ILSI Board of Trustees and Dr. Arti Arora from Coca-Cola, reviewed each of the nominations.

At a special dinner event hosted by Coca-Cola, Dr. Alex Malaspina personally recognized each of the promising young scientists. Dr. Malaspina sees the awards as a "step in not only educating young scientists, but also providing them with the impulse to go back to their own country and start doing innovative, charitable work."

The awards support travel for early-career, academic scientists – in the areas of nutrition, food safety, toxicology, risk assessment or the environment – to the ILSI Annual Meeting, a scientific forum held each January.

The 10 honorees were:

- Bani Tamber Aeri, Ph.D., Assistant Professor, University of Delhi, India
- Clinton Allred, Ph.D., Associate Professor, Department of Nutrition & Food Science, Texas A&M University, U.S.
- Marisa Armeno, M.D., Pediatrician in the Nutrition Department, National Pediatric Hospital, Buenos Aires, Argentina
- Graham Finlaynson, Ph.D., Associate Professor, University of Leeds, U.K.
- Benjamin Kumah Mintah, M.Sc., Food Safety Educator, Department of Nutrition and Food Science, University of Ghana, Ghana
- Siti Muslimatun, Ph.D., Faculty Member and Researcher, The Indonesia International Institute for Life Sciences, Indonesia
- Yukiko Nakanishi, Ph.D., Professor, Department of Health and Nutrition, University of Human Arts and Sciences, Japan
- Zvonimir Šatalić, Ph.D., Assistant Professor, Faculty of Food Technology and Biotechnology, University of Zagreb, Croatia
- Anderson de Souza Sant'Ana, Ph.D., Assistant Professor, Department of Food Science, University of Campinas, Sao Paulo, Brazil
- Krista Varady, Ph.D., Associate Professor, Department of Kinesiology & Nutrition, University of Illinois at Chicago, U.S.

The Malaspina scholars actively participated in the scientific sessions at the ILSI Annual Meeting, presented their research, and met with academic, government and industry researchers from around the world. They were also paired with mentors from the ILSI Board of Trustees, who introduced them to the organization. ILSI will maintain contact with the awardees following the annual meeting and encourage them to participate actively in future ILSI activities at the regional level.

Dr. Wamwari Waichungo, Vice President, Global Scientific and Regulatory Affairs, said, "It was a privilege to establish these awards and host this event honoring Dr. Malaspina and his ILSI legacy."

Find out more about ILSI and Dr. Malaspina in this video.

Wamwari Waichungo, PhD
Vice President
T: (404)676-8017 M: (404)709-0274
Coca-Cola Technical
Global Scientific
and Regulatory Affairs

CONFIDENTIALITY NOTICE
NOTICE: This message is intended for the use of the individual or entity to which it is addressed and may contain information that is confidential, privileged and exempt from disclosure under applicable law. If the reader of this message is not the intended recipient, you are hereby notified that any printing, copying, dissemination, distribution, disclosure or forwarding of this communication is strictly prohibited. If you have received this communication in error, please contact the sender immediately and delete it from your system. Thank You

Lucido, Sal (CDC/ONDIEH/NCCDPHP)

From: Alex Malaspina <(b)(6)>
Sent: Monday, February 23, 2015 6:46 PM
To: scohen@unmc.edu; beauchamp@monell.org; James.Hill@ucdenver.edu; john.c.peters@ucdenver.edu; fergc@foodsci.umass
Subject: Fwd: UPDATE: IFIC Foundation's 2015 Dietary Guidelines Media Call

Mike makes some good points. Any ideas how we can counteract recommendations not based on science but mainly on anectodal

evidence. This matter is now becoming very critical with the latest dietary recommendations. Many thanks. Best regards. Alex

-----Original Message-----
From: Michael Ernest Knowles <(b)(6)>
To: Alex Malaspina <(b)(6)>
Sent: Mon, Feb 23, 2015 7:52 am
Subject: Re: UPDATE: IFIC Foundation's 2015 Dietary Guidelines Media Call

Alex ,

This IFIC media call is a great example of how the industry should respond to biased , non-scientifically-based recommendations . It's after the event of course but will no doubt be successful in ensuring that they do not get adopted as written . It hopefully will also demonstrate to governments that they must have credible scientists in their advisory committees , or else they risk being made to look foolish .

I think that IFIC should follow with further media calls on the key issues raised with again former panel members but reinforced with recognised experts in the subject of the call - for us it would be a call on aspartame to rebut the aspartame allegations .

As to the generation of credible , consensus science on the issues hitting the industry - obesity and causative factors , sugar , low/no calories sweetener safety - in particular we have to use external organisations in addition to any work we directly commission (and that needs to be very carefully reviewed in light of the BMY article !) ; examples are :

> ILSI : as I said in my comments to the RF Director and Board , the RF must establish both safety outside GM foods and nutrition programmes or centres ; this has been strongly supported by another RF Board member who also added that they should add ' risk - benefit ' communications to the public .ILSI was formed by you for the very reason that such contentious issues need to be addressed by all stakeholders i.e.. under the tripartite procedure . The ' One ILSI' strategy currently being developed should do this but it's too slow these issues need to be addressed now in the traditional manner of ILSI - in a transparent manner with the best international experts and the full proceedings published and further publicised by IFIC . The ILSI Branches can build on this for their own countries .

> Scientific Societies : we all belong to one or more of these and we should have leadership roles in the key ones and push for individual issues to be addressed by public conferences / workshops in the manner of ILSI above .

> Medical Associations : we do have good contacts in some and we should encourage them to address public health matters and ' suggest ' appropriate topics - I belong to the Apothecaries Society (it's a very old , traditional medical - 90% medics - organisation in the City of London) and they may be interested - they give courses in ' catastrophe medicine ' so perhaps our issues qualify ! I will talk to my friend Sir Colin Berry who's a Past President of the Society and has advised us on several issues in the past , worked with ILSI and Tox Forum - a vg toxicologist and medic .

> National Academies of Science : in EU we do have an association of these bodies and the UK's , The Royal Society , has recently opened a ' food science ' theme and we can talk to them through the IFST , of which I'm a Fellow , to suggest some form of debate on the issues , ensuring of course that the debaters are balanced !

> EU / Govt. research : this is longer term of course but we should look ahead and proposed work in those which could become ' issues ' and we have vehicles through FDE and ETP Food For Life Board in Europe . The EU Commission is pressing for greater international collaboration with the US at the top of the preferred collaborator list , so we should encourage this through ILSI and our academic contacts .

That's just a selection of ways we can address the issues we currently have and ameliorate future ones and they require an SRA / PAC collaborative approach to be successful .

Hope that helps ; always happy to discuss .

Kind regards

Mike

On 22 Feb 2015, at 16:37, Alex Malaspina <[redacted] (b)(6)> wrote:

-----Original Message-----
From: Alex Malaspina <[redacted] (b)(6)
To: ehays <ehays@coca-cola.com>; ctuggle <ctuggle@coca-cola.com>; hlaman <hlaman@coca-cola.com>
Sent: Sun, Feb 22, 2015 8:41 am
Subject: Fwd: UPDATE: IFIC Foundation's 2015 Dietary Guidelines Media Call

Dear Ed and Clyde: IFIC is coming through for industry. I am looking forward to our visiting them on March 4th. Warmest regards.Alex

-----Original Message-----
From: Dave Schmidt <schmidt@ific.org>
To: Alex Malaspina <[redacted] (b)(6)
Sent: Fri, Feb 20, 2015 9:11 pm
Subject: Fwd: UPDATE: IFIC Foundation's 2015 Dietary Guidelines Media Call

Fyi

Sent from Dave's iPhone

Begin forwarded message:

From: Marianne Smith-Edge <smithedge@ific.org>
Date: February 20, 2015 at 5:53:26 PM EST
Cc: Dave Schmidt <schmidt@ific.org>, Kimberly Reed <reed@ific.org>, !All International Food Information Council Employees <allstaff@ific.org>
Subject: UPDATE: IFIC Foundation's 2015 Dietary Guidelines Media Call

To: IFIC Board of Directors
IFIC Foundation Trustees
All IFIC Committees
IFIC Media Relations Program

From: Marianne Smith Edge

Cc: Dave Schmidt
Kimberly Reed
All IFIC Staff

Date: February 20, 2015

Subject: UPDATE: IFIC Foundation's 2015 Dietary Guidelines Media Call

This morning we had **40+** journalists participate in our DGAC report conference call (see resulting media coverage and bulleted overview below). Participants included the Associated Press, Politico, WBEZ-Chicago, Capitol Press, and trade press as well as nutrition columnists and bloggers. The former DGAC panelists included Dr. Cheryl Achterberg, Dr. Joanne Lupton, Dr. Linda Van Horn, Dr. Theresa Nicklas, Dr. Connie Weaver and Dr. Roger Clemens. This hour-long webcast was recorded and has been **posted to our website**. We also live tweeted and heavily promoted our **new Dietary Guidelines Infographic** during the call. Today's combined Dietary Guidelines communication activities have resulted in more than **393,500 total impressions.**

In addition to the media call, we have compiled a list of **20+** experts with content-specific expertise in DGAC "hot-button" issues (Added Sugars, Aspartame, Caffeine, Sustainability, Cholesterol, Red vs. Lean meat, etc.) who have agreed to be available for media inquiries.

My special thanks to Matt Raymond and Kris Sollid for coordinating the media participation and DGAC panelists, respectively.

RESULTING MEDIA COVERAGE

Politico filed a story this afternoon based on today's call. The author, Chase Purdy, was the most inquisitive reporter in attendance. If you'd like the full text of his story emailed to you, let us know as it is only available to Politico Pro subscribers at this time. We also anticipate an article to be posted by the Associated Press.

Members of the 2015 Dietary Guidelines Advisory Committee aren't just catching grief from the meat and soda industries for their report. On Friday, members of two former dietary advisory panels piled on, as well.

"I think it's good that the new guidelines contained recommendations for action, but it's not clear that all of them are science or evidence based," said Cheryl Achterberg, a nutritional development scholar at Ohio State University who served on the 2010 committee. "I'm not sure a demonization of any food group is going to be productive."

Achterberg was one of five former panel members, from both the 2005 and 2010 committees, to participate in the hour-long event organized by the International Food Information Council. There's little doubt the Ohio State professor was talking about the way the 2015 panel suggested reducing the consumption of red meat, a recommendation that received swift rebuke from the meat industry.
To view the full story online:
https://www.politicopro.com/go/?id=43972

MEDIA CALL OVERVIEW

SCIENCE REVIEW VS. POLICY RECOMMENDATIONS

- Did the 2015 DGAC overstep their bounds? The 2015 evidence review process was discussed with emphasis on how it differed from previous DGA's.
    - 2010 DGAC members noted that they were clearly restricted to providing only and evidence-based review. 2015 seemed to go outside of this guidance in crafting their recommendations.
    - 2015 DGAC has too much emphasis on public policy—specifically with sugars, taxes and labeling—and not enough on evidence-based scientific review.
- Question from reporter asked for reasons for "trendy foodie" recommendations.
    - Responses cited: DGAC selection process; no DGAC agriculture expertise, food regulation or food science members; unbalanced view of presentations made to subcommittees.

MEAT

- The science behind the "lean" and limiting red meat recommendations is conflicting.
- Recommendations should be based on totality of evidence as cultural bias exists when making recommendations about red meat and processed foods. There is no evidence for this recommendation in foreign cultures.

SUSTAINABILITY

- Recommendations on sustainability do not take into account the entire beef food system.
- They give a "soft" definition of sustainability.

ADDED SUGARS AND LCS

- Added Sugars recommendation was based on insufficient evidence to change from a limit of 25% to 10% of calorie intake.
- Evidence about restricting SSB and Added Sugars should be held to the same rigor of evidence as other recommendations.
- Conflicting recommendations about sugar and non-nutritive sweeteners based on different use of epidemiological evidence.
    - The LCS recommendations will likely confuse consumers.

FOOD REFORMULATION

- Will the 2015 DGAs drive reformulation and what are the implications? Will it spur meaningful palate and behavior change?
- Questionable effectiveness of changing the food supply to lower sodium. Instead, there needs to be an increase in appeal of healthful foods like fruits, vegetables and dairy.

DEMONIZATION OF FOODS

- All panelists concurred that the demonization of certain food groups is not productive. Potatoes cited as a prime example.

DGAC VS. DGA

- This point was made very clear—the DGAC recommendations are not the Dietary Guidelines.

CHOLESTEROL

- There will be concern and confusion with the public regarding dietary cholesterol. This should be carefully communicated.

Regards,

Marianne Smith Edge, MS, RD, LD, FADA
Senior Vice President, Nutrition & Food Safety

International Food Information Council
1100 Connecticut Ave, NW Suite 430

Washington, DC 20036
202-296-6540 (office)
202-293-1860 (office direct dial)
270-316-2118 (cell)

<image001.jpg><image004.jpg><image005.jpg>

Lucido, Sal (CDC/ONDIEH/NCCDPHP)

From: Bowman, Barbara (CDC/ONDIEH/NCCDPHP)
Sent: Friday, March 06, 2015 11:17 AM
To: Alex Malaspina
Subject: Fwd: Vital Signs

Dear Alex,
Thank you so much for your interest in our work in heart disease and stroke prevention at CDC. Each year, we do a special report in CDC's Vital Signs series to highlight important prevention issues and opportunities. Here are graphic fact sheets that summarize the most recent reports on salt/sodium intake in children and adults, preventable CVD deaths, and high blood pressure. I would appreciate your thoughts and comments.

Best regards,
Barbara
>
> http://www.cdc.gov/vitalsigns/pdf/2014-09-vitalsigns.pdf
> http://www.cdc.gov/vitalsigns/pdf/2013-09-vitalsigns.pdf
> http://www.cdc.gov/vitalsigns/pdf/2012-09-vitalsigns.pdf
> http://www.cdc.gov/vitalsigns/pdf/2012-02-vitalsigns.pdf
> http://www.cdc.gov/vitalsigns/pdf/2011-02-vitalsigns.pdf
>
>
>
>

Lucido, Sal (CDC/ONDIEH/NCCDPHP)

**From:**	Alex Malaspina (b)(6)
**Sent:**	Tuesday, March 10, 2015 8:36 AM
**To:**	Bowman, Barbara (CDC/ONDIEH/NCCDPHP)
**Subject:**	Re: Vital Signs

Dear Barbara:
I like all your bulletins. Very clear ,concise and very understanding. I wish you a great success.
Is it Ok if I send them to Suzie Harris and Jim Hill and also to Wamwary and Clyde Tuggle. Maybe you prefer to wait until finally published
Warmest Personal regards. Alex

-----Original Message-----
From: Bowman, Barbara (CDC/ONDIEH/NCCDPHP) (CDC/ONDIEH/NCCDPHP) <bbb8@cdc.gov>
To: Alex Malaspina (b)(6)
Sent: Fri, Mar 6, 2015 11:18 am
Subject: Fwd: Vital Signs

Dear Alex,
Thank you so much for your interest in our work in heart disease and stroke prevention at CDC. Each year, we do a special report in CDC's Vital Signs series to highlight important prevention issues and opportunities. Here are graphic fact sheets that summarize the most recent reports on salt/sodium intake in children and adults, preventable CVD deaths, and high blood pressure. I would appreciate your thoughts and comments.

Best regards,
Barbara
>
>
http://www.cdc.gov/vitalsigns/pdf/2014-09-vitalsigns.pdf
>
http://www.cdc.gov/vitalsigns/pdf/2013-09-vitalsigns.pdf
>
http://www.cdc.gov/vitalsigns/pdf/2012-09-vitalsigns.pdf
>
http://www.cdc.gov/vitalsigns/pdf/2012-02-vitalsigns.pdf
>
http://www.cdc.gov/vitalsigns/pdf/2011-02-vitalsigns.pdf
>
>
>
>

Bowman, Barbara (CDC/ONDIEH/NCCDPHP)

**From:**	Bowman, Barbara (CDC/ONDIEH/NCCDPHP)
**Sent:**	Tuesday, March 10, 2015 9:18 AM
**To:**	Alex Malaspina
**Subject:**	RE: Vital Signs

Dear Alex,

Many thanks, glad you like the Vital Signs releases. They are all published, so wonderful if you'd like to distribute, we do one every year for heart disease and stroke prevention. These Fact Sheets accompany a scientific/ technical report in our weekly publication MMWR (Morbidity and Mortality Weekly Report). CDC's nutrition Division also does one every year.

Best regards,
Barbara

From: Alex Malaspina (b)(6)
Sent: Tuesday, March 10, 2015 8:36 AM
To: Bowman, Barbara (CDC/ONDIEH/NCCDPHP)
Subject: Re: Vital Signs

Dear Barbara:
I like all your bulletins. Very clear ,concise and very understanding. I wish you a great success.
Is it Ok if I send them to Suzie Harris and Jim Hill and also to Wamwary and Clyde Tuggle. Maybe you prefer to wait until finally published
Warmest Personal regards. Alex

-----Original Message-----
From: Bowman, Barbara (CDC/ONDIEH/NCCDPHP) (CDC/ONDIEH/NCCDPHP) <bbb8@cdc.gov>
To: Alex Malaspina (b)(6)
Sent: Fri, Mar 6, 2015 11:18 am
Subject: Fwd: Vital Signs

Dear Alex,
Thank you so much for your interest in our work in heart disease and stroke prevention at CDC. Each year, we do a special report in CDC's Vital Signs series to highlight important prevention issues and opportunities. Here are graphic fact sheets that summarize the most recent reports on salt/sodium intake in children and adults, preventable CVD deaths, and high blood pressure. I would appreciate your thoughts and comments.

Best regards,
Barbara
>
>
http://www.cdc.gov/vitalsigns/pdf/2014-09-vitalsigns.pdf
>
http://www.cdc.gov/vitalsigns/pdf/2013-09-vitalsigns.pdf
>
http://www.cdc.gov/vitalsigns/pdf/2012-09-vitalsigns.pdf
>
http://www.cdc.gov/vitalsigns/pdf/2012-02-vitalsigns.pdf
>
http://www.cdc.gov/vitalsigns/pdf/2011-02-vitalsigns.pdf
>

HP

Peters, John C

From: Alex Malaspina
Sent: Tue 5/19/2015 4:45 PM (GMT-07:00)
To: sharris@ilsi.org; pjulkunen@coca-cola.com
Cc: Peters, John C; Hill, James; (b)(6) fergc@foodsci.umass.edu; beauchamp@monell.org; schmidt@ific.org; rowe@srstrategy.com; rwilkinson@coca-cola.com; (b)(6) (b)(6)
Bcc:
Subject: Fwd: Policy brief launched to curb global sugar consumption

FYI.Alex

-----Original Message-----
From: Herve Nordmann <herve.nordmann@asg.ajinomoto.com>
To: Alex Malaspina (b)(6)
Sent: Tue, May 19, 2015 12:18 pm
Subject: RE: Policy brief launched to curb global sugar consumption

Thank you Alex for sending this to me. It seems that WHO is forgetting about the importance of sweet taste in the whole issue. They never speak about sweeteners as alternative to sugar to satisfy sweet craving without the calories... to imagine going from 120 g sugar a day to 25 g a day in less than one generation is absurd ... the sugar containing recipes are so plenty and so deeply in rooted in our daily cuisine that we would have to change nearly everything we do, for something we may like much less! My view is that sugar has to stay in solid applications and that it should be substituted in liquid applications where LCS can do the job from the taste point of view but also from the public health point of view... that only change could save billions in public health expenditures and preserve millions of people from unduly suffering from obesity, diabetes and metabolic syndrome.
Yours sincerely

Hervé

P.S.
Thank you for the good news from Paivi!

-----Original Message-----
From:
Alex Malaspina (b)(6)
Sent: lundi 18 mai 2015 15:24
To:
Herve Nordmann
Subject: Fwd: Policy brief launched to curb global sugar

consumption

FYI. Alex

-----Original Message-----
From: Barbara Bowman
(b)(6)
To: Alex Malaspina (b)(6)
Sent: Mon, May 18, 2015
9:22 am
Subject: Fwd: Policy brief launched to curb global sugar
consumption

Sent from my iPhone

From: World Cancer
Research Fund International < international@wcrf.org>

Date:
May 18, 2015 at 5:40:28 AM EDT
Subject: Policy brief launched to
curb global sugar consumption
Reply-To: World Cancer Research Fund
International < international@wcrf.org>

2

The latest news from WCRF International

View this email in your browser

[illegible]

Policy brief launched to curb global sugar consumption

Today, World Cancer Research Fund International has launched a policy brief to help reduce the amount of sugar consumed worldwide. The brief - Curbing Global Sugar Consumption – is the first to provide an analysis of the effectiveness of sugar policies that have been implemented around the world.

Published to coincide with the World Health Assembly, the policy brief comes at a time when sugar consumption is on the rise and is designed to help countries meet the new sugar Guideline, which was published by the World Health Organization (WHO) in March of this year.

The WHO sugar Guideline
recommends that adults and children restrict their sugar intake to less than 10% of total daily energy intake - the equivalent of around 12.5 teaspoons of sugar for adults - and suggests a further reduction to below 5% of total daily energy intake for additional health benefits.

In addition to providing concrete
examples of effective policy actions - from soda taxes to a vending machine ban – and speaking to the experts who were involved in the development and implementation of the policies, the brief identifies the four key areas where action is needed to reduce the amount of sugar consumed: availability, affordability, acceptability and awareness.

Full details are available in
the policy brief or you can read a blog by Bryony Sinclair, our Policy and

Public Affairs Manager, or the guest blog by Professor Barry Popkin, one of the world's leading experts on dietary behaviour.

Facebook

Twitter

Blog

Thank you for subscribing to receive updates from World Cancer Research Fund International, the cancer prevention organisation that leads and unifies a network of cancer charities dedicated to the prevention of cancer through diet, weight and physical activity.

If you have been forwarded this eNews by a friend and would like to receive your own copy in future please subscribe here.

If you would prefer not to receive future WCRF International eNews please unsubscribe here.

www.wcrf.org

Peters, John C

From: Alex Malaspina
Sent: Fri 6/26/2015 4:44 PM (GMT-07:00)
To: (b)(6)
Cc: sharris@ilsi.org
Bcc:
Subject: Re: Daily European News Flash - 25.06.15

Dear Barbara: Thanks for your advice. (b)(6) I liked that lady so much. She did make the ILSI focal Point a Star (b)(6)
However, I had a similar idea and have asked Junshi Chen his advise. I do not know the Group you mentioned but I will Check with Suzie.
I hope you are having a great summer.
Warmest personal regards. Alex

-----Original Message-----
From: Barbara Bowman (b)(6)
To: Alex Malaspina (b)(6)
Sent: Fri, Jun 26, 2015 9:07 am
Subject: Re: Daily European News Flash - 25.06.15

Dear Alex,
Sorry for the delay in my reply, I have been off this week and away from e-mail until now.
Am wondering whether anyone with ILSI China, perhaps Madame Chen, might have ideas. Another thought, perhaps someone with connections to the PEPFAR program. Or Gates and Bloomberg people, many have close connections with the WHO regional offices. Perhaps an issue of defining legacy.
Best,
Barbara

Sent from my iPhone

> On Jun 26, 2015, at 8:28 AM, Alex Malaspina (b)(6) wrote:
>
> Dear Barbara: How are you? Are you having a nice summer?
> Any ideas how we can have a conversation with WHO?
Now, they do not want to work with industry. Who finds all the new drugs. Not WHO-, but industry. She is influenced by the Chinese Govt and is against US. Something must be done.
> Warmest regards. Alex

MP

Peters, John C

From: Alex Malaspina
Sent: Sat 6/27/2015 1:39 AM (GMT-07:00)
To: (b)(6)
Cc: ctuggle@coca-cola.com; ehays@coca-cola.com; adrewnow@fredhutch.org; sharris@ilsi.org; rwilkinson@coca-cola.com; fergc@foodsci.umass.edu; Hill, James; Peters, John C; scohen@unmc.edu; beauchamp@monell.org; hzhang@coca-cola.com
Bcc:
Subject: Re: Daily European News Flash - 25.06.15

Dear Barbara :
You gave me some very good leads. I like the one especially about having Mr. Bill Gates help. Our Chairman knows him well. I will explore this idea with Clyde. We would want WHO to start working with ILSI again ,with the GEBN and with the food industry in general to resolve issues of food-safety and nutrition and for WHO to not only consider sugary foods as the only cause of obesity but to consider also the life style changes that have been occurring through out the Universe.

Since WHO ,as you stated has been helped by the pharmaceutical industry to combat HIV/AIDS, why not work closely with the food industry to combat obesity. The Food industry is very willing to come to the table.
Let us have dinner soon. After the 4th of July , I am free until the 22nd.Then Free again From July 26 to August 24.
Warmest personal regards. Alex

-----Original Message-----
From: Barbara Bowman (b)(6)
To: Alex Malaspina (b)(6)
Sent: Fri, Jun 26, 2015 8:57 pm
Subject: Re: Daily European News Flash - 25.06.15

Dear Alex,
Very sorry to hear about Madame Chen, she was absolutely remarkable.
PEPFAR is the US government-sponsored program that makes HIV/AIDS drugs available throughout sub-Saharan Africa, a great success in saving lives.
WHO is key to the network. Will be in touch about getting together, all best wishes.
Barbara

Sent from my iPhone

> On Jun 26, 2015, at 7:44 PM, Alex Malaspina (b)(6) wrote:
>
> Dear Barbara: Thanks for your

advice. (b)(6) liked that lady
so much. She did make the ILSI focal Point a Star. (b)(6)
(b)(6)
> However, I had a similar idea and have asked Junshi Chen his advise. I
do not know the Group you mentioned but I will Check with Suzie.
> I hope you
are having a great summer.
> Warmest personal regards. Alex
>
>
>
-----Original Message-----
> From: Barbara Bowman (b)(6)
> To: Alex
Malaspina (b)(6)
> Sent: Fri, Jun 26, 2015 9:07 am
> Subject: Re:
Daily European News Flash - 25.06.15
>
>
> Dear Alex,
> Sorry for the
delay in my reply, I have been off this week and away
> from e-mail until now.

> Am wondering whether anyone with ILSI China, perhaps
> Madame Chen, might
have ideas. Another thought, perhaps someone with
> connections to the PEPFAR
program. Or Gates and Bloomberg people, many have
> close connections with the
WHO regional offices. Perhaps an issue of defining
> legacy.
> Best,
>
Barbara
>
> Sent from my iPhone
>
>>> On Jun 26, 2015, at 8:28 AM,
>>
Alex Malaspina (b)(6) wrote:
>>
>> Dear Barbara: How are you?
Are
> you having a nice summer?

>> Any ideas how we can have a conversation
with WHO?
> Now, they do not want to work with industry. Who finds all the new
drugs. Not
> WHO-, but industry. She is influenced by the Chinese Govt and is
against US.
> Something must be done.
>> Warmest regards. Alex
>>
>>
>>
-----Original
> Message-----
>> From: Alex Malaspina (b)(6)
>>
To: ctuggle
> <ctuggle@coca-cola.com>; ehays <ehays@coca-cola.com>; sharris
>
<sharris@ilsi.org>; fergc <fergc@foodsci.umass.edu>
>> Cc: James.Hill
>
<James.Hill@ucdenver.edu>; jamesrbehnke (b)(6)
john.c.peters
> <john.c.peters@ucdenver.edu>; john (b)(6);
jshchen
> <jshchen@ilsichina.org>; hzhang <hzhang@coca-cola.com>
>> Sent: Thu,
Jun 25,
> 2015 7:14 am
>> Subject: Fwd: Daily European News Flash -
25.06.15
> Please see report on WHO. This is getting a lot of publicity.
>>
We must find
> a
>> way of some one such as a famous scientist arrange to pay
her a visit .
> May be
>> Jim Hill or some one of similar stature or a US
Government scientist
> .
>>
>> As
>> the President of ILSI I had a special
and productive luncheon
> with the former

>> DG ,Dr. Nakajima in 1995 at his
private dinning room in the
> WHO Geneva
>> Headqurters to tell him about
ILSI and how the two organization
> could work with
>> each other,
>>
>>
In 1999 I visited with the new DG Mrs.
> Brutland in Geneva,
>> when I
invited her ,on behalf of The World Economic
> Forum, to come to the Davos
>>
meeting of 1999, and be the Keynote Speaker at
> the Food Governors
special
>> dinner,where she discussed that GMO foods are not
> only safe but
could be very
>> useful in feeding a hungry world. By the way ,the
> future
Coke President ,Mr.
>> Neville Isdell attended that dinner with me.
>
>> In
summary I am suggesting that
>> collectively we must find a way to start
> a
dialogue with Dr. Chen. If not, she
>> will continue to blast us with
>
significant negative consequences on a global
>> basis. This threat to our
>
business is serious,
>> Warmest regards.
>> Alex
>>
>>
>>
-----Original
> Message-----
>> From: Susanne Kettler
>>

<skettler@coca-cola.com>
>> To: Alex
> Malaspina (b)(6)
>> Sent:
Thu, Jun
>> 25, 2015 3:24 am
>> Subject:
> Fwd: Daily European News Flash -
25.06.15
>>
>>
>>
>>
>>
>>
>>
>> Sent
> from my iPhone
>>
>>

>> Begin forwarded message:
> From: Coke Email <
CokeEmail@bluerubicon.com>
>> Date: 25 Jun
>> 2015
> 08:42:42 CEST
>>
To: Undisclosed recipients:;
>> Subject:
> Daily European News Flash -
25.06.15
>>
>>
>>
>>
>>
>>
>>
>>
>> Key
> Highlights: In
the UK, marketing title Thedrum.com quotes Wendy Clark,
> President of
Sparkling Brands & Strategic Marketing at Coca-Cola North
> America,

>> who
addressed the audience at the Cannes Lions festival. She is
> cited as
saying
>> that Coca-Cola aligns its marketing to the passion points of
>
teenagers and young
>> adults to stay relevant. Media in Spain, Ireland,
Denmark
> and UK discuss the
>> negative impact of products with a high sugar
content on
> dental health. Finally,
>> media in Belgium, UK and France
continue to report
> that celebrity chef Jamie
>> Oliver will implement a
'soda tax' on all
> sugar-sweetened beverages available in
>> his restaurants
in the hope that the
> UK government will also implement such a
>> tax.
>>

>>
>>
>> All other
> coverage shared as background.
>>
>>
>>
>>

>> Coca-Cola News
>
>> Coca-Cola and Cannes Lions (UK)
>>
>>
>>
>>

>> Thedrum.com – "When
> you get the relevance right the consistency
pays
>> off in spades' – Coca-Cola's

> Wendy Clark on keeping the brand
fresh', 24.06.15
>>
>>
>> By Seb Joseph
>
>>
>>
>> Reports on
Coca-Cola's President of
>> Sparkling Brands &
> Strategic Marketing at
Coca-Cola North America, Wendy Clark,
>> and her address
> to the audience at
Cannes Lions. Ms Clark is cited as saying
>> that
> "consistency, times
relevance, done at scale and done over time - those
>> four
> things have been
at the core of what Coke has done..." It is noted that to
>> stay
> relevant the
brand looks to the passion points that appeal to teenagers and
> young adults
and wraps its marketing around those elements.
>>
>> (KO
> mention)
>>

>> Blog Authority: 5
>>
>> Shares: Twitter 12, LinkedIn 1,
> Facebook
1
>>
>> Readership: 25,000
>>
>> Action: FYI
> Coca-Cola HBC Annual
General Meeting (UK, Greece)
> Shares.telegraph.co.uk – 'Coca-Cola HBC
resolutions passed at AGM', 24.06.15
>

>>
>>
>>
>> Reports that
Coca-Cola HBC has confirmed that all of the
> proposals
>> set out in the
notice of the annual general meeting were passed by
> shareholders
>>
yesterday. It is also noted that Coca-Cola HBC AG confirmed the
> election
of
>> Olusola (Sola) David-Borha and Alexandra Papalexopoulou as
>
non-executive
>> directors at the annual general meeting yesterday.
>>
>>
Also
> reported:
>> Halifaxmarketwatch.co.uk, Investing.thisismoney.co.uk,
>
Stockmarketwire.com,
>> Moneyam.com, Shares.telegraph.co.uk (2),
Uk.advfn.com,
> Hl.co.uk, Iii.co.uk,
>> Sharewatchglobal.digitallook.com,
Lse.co.uk,
> Bullbearings.co.uk, Capital.gr,
>> Uk.finance.yahoo.com,
Capital.gr (2),
> Livester.gr
>>
>> (KO mention)
>>
>>
>> Authority:
10
>>
>> Shares: N/A
>
>> Readership: c.1,371,000
>>
>>
>> Action:

FYI
>>
>>
>>
>> Coca-Cola
> Hilltop Parody (Spain)
>>
>>
>>
>>

>> Marketingdirecto.com – '"Change the
> tune": el remake de "Hilltop"
para
>> concienciar sobre el consumo excesivo de
> refrescos', 24.06.15
>>

>>
>>
>>
>> Reports on a new video from the
> Center for Science in the
Public Interest,
>> which parodies Coca-Cola's famous
> 1971 advert known as
Hilltop. It is noted that
>> the advert recently made an
> appearance in the
Mad Men TV show finale. The parody
>> version claims to draw
> attention to
illnesses such as hypertension, diabetes and
>> obesity said to be
>
associated with overconsumption of fizzy drinks.
>>
>>
>> Also
reported:
> Horizont.net, Gastronomiaycia.com
>>
>> (KO mention)
>>
>>

>> Authority:
> 2
>>
>> Shares: Google+ 1, Twitter 68, Facebook 10
>>

>>
>> Readership: c.
> 22,300
>>
>> Action: Spain PAC team to monitor

>>
>>
>>
>>
>> Mad Men
> Finale (UK)
>>
>>
>>
>>
Dailymail.co.uk – 'Hard to get the 'real
>> thing'!
> Jon Hamm says it took
Mad Men creator Matthew Weiner 'a couple of years'
>> to
> persuade Coca Cola
to let him use their ad in the finale', 24.06.15
> By Rachel McGrath
>>
>>

>>
>> Reports that Mad Men star, Jon Hamm, said
> that it took Mad Men
creator Matthew Weiner two years to persuade The Coca
> Cola
>> Company to use
its 1971 I'd Like To Buy The World A Coke commercial to
> end the
>> AMC
series finale in May this year. It is noted that after the finale
> aired,
a
>> spokesperson from Coca Cola told People Magazine that "no money

>
exchanged hands"
>> In the agreement between AMC and the fizzy-drink
company.
>
>> Also reported:
>> Dailymail.co.uk (2), Dailymail.co.uk (3)

>>
>> (KO
> mention)
>>
>>
>> Authority: 10
>>
>> Shares: Shared
31, Comments 49
> Readership: c.
>> 2,912,000
>>
>> Action: FYI
>>
>>

>>
>> Industry
> News
>>
>>
>>
>>
>> Nutritional Measures
>>
>>

>>
>> US Fizzy Drink Tax
> Proposal (France)
>>
>>
>>
>>
>>
Nytimes.com – 'Republicans Push Back
> Against Proposed Dietary
>>

Guidelines', 24.06.15
>>
>>
>>
>> Reports on
> political debates in the
US
>> House and Senate regarding nutrition and diet
> regulations. It is noted
that an
>> advisory panel of independent doctors and
> nutrition experts have
suggested a tax
>> on sugar-sweetened drinks and snacks
> as means to
encourage better eating habits.
>> The publication underscores that
> the idea
angered beverage companies and
>> conservatives in Congress.
>>
>> (No
> KO
mention)
>>
>> Authority: 10
>>
>>
>> Shares: N/A
>>
>> Readership:
c.
> 3,072,000
>>
>> Action: FYI
>>
>>
>>
>>
>> VAT Increase
(Greece)
>
>>
>> Madata.gr – 'Τα προϊόντα και οι
>> υπηρεσίες που θα
ακριβύνουν με το νέο

> ΦΠΑ', 24.06.15
>>
>>
>>
>> Reports
>> on the new
VAT regime in Greece,
> which will increase the rate to 23 per cent. It
>> is
noted that the increase
> will affect goods such as soft drinks and juice,
>>
sugar, coffee, chocolates as
> well as all processed and canned foods.
>>

>>
>> Also reported: Lay-out.gr,
> Newpost.gr, Karfitsa.gr,
Lefimerida.gr,
>> Skaipatras.gr, Aftodioikisi.gr
> (No KO mention)
>>
>>
Authority: 2
>>
>>
>> Shares: Twitter 1, Facebook 2
>
>> Readership: c.
12,600
>>
>> Action:
>> FYI
>>
>>
>>
>> Jamie Oliver's
> Sugar Tax
(Belgium, UK, France)
>>
>>
>>
>>
>> Nieuwsblad.be – 'Jamie
> Oliver

heft belasting op frisdranken',
>> 24.06.15
>>
>>
>>
>> Reports
> that
British celebrity chef Jamie Oliver is
>> going to implement a "soda tax"
> on
all sugary drinks available in his
>> restaurants in hopes that the
>
government will also implement such a tax. It is
>> stated that the extra
>
revenue from the price increase will be invested in
>> teaching children
about
> healthy eating. A UK publication reports that a Tory
>> backbencher
has
> commented on Oliver's move, noting that he is against a sugar
>> tax and
that
> there are "far more dangerous white powders." Another publication
>>
reports
> that the Food and Drink Federation has welcomed Oliver's commitment
to
> improving public health, but has insisted that a real sugar tax would fail
as
> such measures have reportedly proven ineffective.
>>
>> Also
reported:
> Meltyfood.fr, Atabula.com, Homemadebyyou.co.uk,
Foodmanufacture.co.uk,
>
>> Atabula.com, Nieuws.be, Hbvl.be,
Foodmanufacture.co.uk, Lexpress.fr,
> Agripress.be,
>>
>> Standaard.be,
Planet.fr, Bristolpost.co.uk,
> Foodmanufacture.co.uk

>>
>> (No KO
mention)
>>
>> Authority: 3
> Shares: 11
>>
>> Readership: c. 70,400
>>

>> Action: Local Pac teams to
> monitor
>>
>>
>>
>> WHO Proposals on
Soft Drinks (UK, Spain)
>
>>
>> Just-drinks.com – 'World Health
Organization calls for full-sugar
> soft
>> drinks regulations', 24.06.15
>>

>> By Keith Nuthall
>
>> Reports that the Director General of the World
Health Organization (WHO)
> has
>> accused marketers of full sugar
non-alcoholic beverages of contributing
> to
>> rising obesity rates amongst
children globally, especially in
> developing
>> countries. Dr Margaret Chan
is also backing regulations that would
> restrict the
>> consumption of full
sugar soft drinks, noting that voluntary
> initiatives are
>> less likely to
be enough. A Spanish publication reports that
> the introduction of
>> a tax

on sugar-sweetened drinks in Mexico has resulted in
> a 12 per cent
>>
reduction of consumption of these beverages. It is also noted
> that in 2012,
the
>> Catalonian Parliament approved a bill mandating a tax on
>
sugar-sweetened drinks,
>> but it was never implemented.
>>
>> Also
reported:
> Consalud.es,
>> Cronicanorte.es
>>
>> (No KO mention)
>>

>> Authority:
> 1
>>
>>
>> Shares: 9
>>
>> Readership: c. 1,400
>>

>> Action: Local PAC
> teams to
>> monitor
>>
>>
>>
>> Sugar and
Sweeteners
>>
>>
>>
>> Sugar
> and
>> Health (Spain, France)
>>
>>

>>

>> Consumer.es – '¿Cuánta azúcar
> debemos
>> consumir?', 24.06.15
>>

>> By Isabel Megias
> Reports that
>> sugar is becoming more prevalent in
our diets due to quantities
> now found in
>> everyday products. The article
also mentions guidelines on daily
> sugar intake
>> from the World Health
Organization (WHO) for adults and
> children. Illnesses
>> associated with
overconsumption of sugar, such as
> obesity, hypertension, high
>> cholesterol
and vascular diseases, are also
> discussed. Additional coverage also
>>
discusses the prevalence of hidden sugars
> in everyday foods.
>>
>>
Also
>> reported: Femmeactuelle.fr
>>
>> (No KO
> mention)
>>
>>
Authority: 5
>>
>>
>> Shares: 0
>>
>> Readership: c.
> 28,400
>>
>>
Action: FYI
>>
>>

>>
>>
>> Soft Drinks and Health
>
>>
>>
Coca-Cola Life (Germany)
>>
>>
>>
>>
>> Badische-zeitung.de –
> 'Ist
grüne Coke gesünder?', 24.06.15
>>
>> By
>> Claudia Füßler
>
>> Discusses
Coca-Cola Life, noting that even
>> though the drink is partly
> sweetened
with stevia, has a lower calorie content
>> than regular Coke and is
>
marketed as a healthier alternative, it still contains
>> more sugar in a
>
half-litre bottle than the daily limit recommended by the World
>> Health
>
Organization. A nutritionist is cited as commenting that Coca-Cola Life
>>
is
> still a "sugar bomb" despite its lower sugar content.
>>
>> (KO
mention:
> Coca-Cola Life)
>>
>> Authority: N/A
>>
>> Shares: 0
>>
>>
Readership: c.

> 19,100
>>
>> Action: Germany PAC team to monitor
>>
>>

>>
>> Fizzy
> Drinks and Health (Spain, Ireland, Denmark, UK)
>
Eleconomista.es – 'La erosión dental aumenta en verano por el consumo de
>
bebidas azucaradas y la exposición al cloro', 24.06.15
> Reports on factors
that can negatively impact dental health during the summer
> months, including
consumption of carbonated soft drinks and other products
> with
>> high sugar
content such as ice-cream. It is claimed that these products
> increase
>> the
risk of dental cavities. An Irish publication reports that
> despite the
>>
alleged health benefits of smoothies, they may also have a
> detrimental effect
to
>> human health. Celebrity dentist, Dr Uchenna Okoye is
> quoted as arguing
that
>> fructose found in most fruit damages tooth enamel,
> which in turn
leads to decay.
>> A Danish source comments on the negative effect
> of
phosphoric acid contained in
>> cola drinks does to calcium in bones. A UK
>
publication quotes Professor Colin
>> Hill, a microbiologist at University
>
College Cork in Ireland, as stating that
>> "no intervention will overcome
>
someone drinking lots of fizzy drinks."

>>
>>
>> Also reported:
>
Diariosigloxxi.com, Larazon.es, Her.ie, Samvirke.dk,
>> Dailymail.co.uk
> (No
KO mention)
>>
>> Authority: 4
>>
>> Shares: 3
>>
>>
>> Readership:
c.
> 126,000
>>
>> Action: Local PAC teams to monitor
> Methodology
>>

>>
>>
>> Authority – Authority score is provided by
> Sysomos. Each data
source is assigned a score between 0 (lowest) – 10
> (highest).
>> Authority
is measured using a number of factors including in-link
> count and
>> reach
for traditional media and in-link count, comments, reach and
> bookmarks
for
>> blogs. Where a specific source is retrieved using another
> platform
'N/A' will be
>> assigned.
>>
>>
>>
>> Shares - This data is taken
>
from the publication
>> itself, and includes the number of shares across

>
platforms. Shares include
>> recommendations on Facebook and Twitter and
>
comments on the article in question.
>> When this information is not
provided,
> 'N/A' is shown. The type of shares will
>> be broken down by
platform when the
> total sum is greater than 10.
>>
>>
>>
>>
>>
Readership – Readership gives
> an indication of the size of audience
reached
>> and is based on a unique
> visitors estimate by
Siteworthtraffic.com, which uses
>> data by web analysis
> platform Alexa.
>

> CONFIDENTIALITY
NOTICE
>> NOTICE: This message is intended for the use of the
> individual or
entity to which it is addressed and may contain information
> that
>> is
confidential, privileged and exempt from disclosure under applicable
> law.
If
>> the reader of this message is not the intended recipient, you are
>
hereby
>> notified that any printing, copying, dissemination, distribution,
>
disclosure or
>> forwarding of this communication is strictly prohibited. If
you
> have received
>> this communication in error, please contact the

sender
> immediately and delete it
>> from your system. Thank You.
>

>
>
>

>
>
> -----Original
Message-----
> From: Alex Malaspina <(b)(6)
> To: ctuggle
<ctuggle@coca-cola.com>; ehays <ehays@coca-cola.com>; sharris
<sharris@ilsi.org>; fergc <fergc@foodsci.umass.edu>
> Cc: James.Hill
<James.Hill@ucdenver.edu>; jamesrbehnke <(b)(6); john.c.peters
<john.c.peters@ucdenver.edu>; john (b)(6); jshchen
<jshchen@ilsichina.org>; hzhang <hzhang@coca-cola.com>
> Sent: Thu, Jun 25,
2015 7:14 am
> Subject: Fwd: Daily European News Flash - 25.06.15
>
>
>
Please see report on WHO. This is getting a lot of publicity.
> We must find
a
> way of some one such as a famous scientist arrange to pay her a visit .
May be
> Jim Hill or some one of similar stature or a US Government scientist
.
>
> As
> the President of ILSI I had a special and productive luncheon
with the former
> DG ,Dr. Nakajima in 1995 at his private dinning room in the
WHO Geneva
> Headqurters to tell him about ILSI and how the two organization
could work with
> each other,
>
> In 1999 I visited with the new DG Mrs.
Brutland in Geneva,
> when I invited her ,on behalf of The World Economic
Forum, to come to the Davos
> meeting of 1999, and be the Keynote Speaker at
the Food Governors special
> dinner,where she discussed that GMO foods are not
only safe but could be very
> useful in feeding a hungry world. By the way ,the
future Coke President ,Mr.
> Neville Isdell attended that dinner with me.

>

> In summary I am suggesting that
> collectively we must find a way to start
a dialogue with Dr. Chen. If not, she
> will continue to blast us with
significant negative consequences on a global
> basis. This threat to our
business is serious,
> Warmest regards,
> Alex
>
>
> -----Original
Message-----
> From: Susanne Kettler
> <skettler@coca-cola.com>
> To: Alex
Malaspina (b)(6)
> Sent: Thu, Jun
> 25, 2015 3:24 am
> Subject:
Fwd: Daily European News Flash - 25.06.15
>
>
>
>
>
>
>
> Sent
from my iPhone
>
>
> Begin forwarded message:
>
>
>
>
>
>
From: Coke Email < CokeEmail@bluerubicon.com>
> Date: 25 Jun
> 2015
08:42:42 CEST
> To: Undisclosed recipients:;

> Subject:
>
Daily European News Flash - 25.06.15
>
>
>
>
>
>
>
>
> Key
Highlights: In the UK, marketing title Thedrum.com quotes Wendy Clark,
>
President of Sparkling Brands & Strategic Marketing at Coca-Cola North America,
> who addressed the audience at the Cannes Lions festival. She is cited as saying
> that Coca-Cola aligns its marketing to the passion points of teenagers and young
> adults to stay relevant. Media in Spain, Ireland, Denmark and UK discuss the
> negative impact of products with a high sugar content on dental health. Finally,
> media in Belgium, UK and France continue to report that celebrity chef Jamie
> Oliver will implement a 'soda tax' on all sugar-sweetened beverages available in
> his restaurants in the hope that the UK government will also implement such a
> tax.
>
>
>
> All other
coverage shared as background.
>
>
>
>
> Coca-Cola News
>
>
>

> Coca-Cola and Cannes Lions (UK)

14

>
>
>
>
> Thedrum.com – "When
you get the relevance right the consistency pays
> off in spades' – Coca-Cola's
Wendy Clark on keeping the brand fresh', 24.06.15
>
>
> By Seb Joseph
>

>
>
> Reports on Coca-Cola's President of
> Sparkling Brands &
Strategic Marketing at Coca-Cola North America, Wendy Clark,
> and her address
to the audience at Cannes Lions. Ms Clark is cited as saying
> that
"consistency, times relevance, done at scale and done over time - those
> four
things have been at the core of what Coke has done..." It is noted that to
> stay
relevant the brand looks to the passion points that appeal to teenagers and
>
young adults and wraps its marketing around those elements.
>
> (KO
>
mention)
>
> Blog Authority: 5
>
> Shares: Twitter 12, LinkedIn 1,
>
Facebook 1
>
> Readership: 25,000
>
> Action: FYI
>
>
>
>

>
Coca-Cola HBC Annual General Meeting (UK, Greece)
>
>
>
>
>
Shares.telegraph.co.uk – 'Coca-Cola HBC resolutions passed at AGM', 24.06.15
>

>
>
>
> Reports that Coca-Cola HBC has confirmed that all of the
proposals
> set out in the notice of the annual general meeting were passed by
shareholders
> yesterday. It is also noted that Coca-Cola HBC AG confirmed the
election of
> Olusola (Sola) David-Borha and Alexandra Papalexopoulou as
non-executive
> directors at the annual general meeting yesterday.
>
> Also
reported:
> Halifaxmarketwatch.co.uk, Investing.thisismoney.co.uk,
Stockmarketwire.com,
> Moneyam.com, Shares.telegraph.co.uk (2), Uk.advfn.com,
Hl.co.uk, Iii.co.uk,
> Sharewatchglobal.digitallook.com, Lse.co.uk,
Bullbearings.co.uk, Capital.gr,
> Uk.finance.yahoo.com, Capital.gr (2),
Livester.gr
>
> (KO mention)
>
>
> Authority: 10
>
> Shares: N/A
>

> Readership: c.1,371,000
>
>
> Action: FYI

>
>
>
> Coca-Cola
Hilltop Parody (Spain)
>
>
>
>
> Marketingdirecto.com – "'Change the
tune": el remake de "Hilltop" para
> concienciar sobre el consumo excesivo de
refrescos', 24.06.15
>
>
>
>
> Reports on a new video from the
Center for Science in the Public Interest,
> which parodies Coca-Cola's famous
1971 advert known as Hilltop. It is noted that
> the advert recently made an
appearance in the Mad Men TV show finale. The parody
> version claims to draw
attention to illnesses such as hypertension, diabetes and
> obesity said to be
associated with overconsumption of fizzy drinks.
>
>
> Also reported:
Horizont.net, Gastronomiaycia.com
>
> (KO mention)
>
>
> Authority:
2
>
> Shares: Google+ 1, Twitter 68, Facebook 10
>
>
> Readership: c.
22,300
>
> Action: Spain PAC team to monitor
>

>
>
>
> Mad Men
Finale (UK)
>
>
>
> Dailymail.co.uk – 'Hard to get the 'real
> thing'!
Jon Hamm says it took Mad Men creator Matthew Weiner 'a couple of years'
> to
persuade Coca Cola to let him use their ad in the finale', 24.06.15
>
>
>
By Rachel McGrath
>
>
>
> Reports that Mad Men star, Jon Hamm, said
>
that it took Mad Men creator Matthew Weiner two years to persuade The Coca
Cola
> Company to use its 1971 I'd Like To Buy The World A Coke commercial to
end the
> AMC series finale in May this year. It is noted that after the finale
aired, a
> spokesperson from Coca Cola told People Magazine that "no money
exchanged hands"
> in the agreement between AMC and the fizzy-drink company.
>

> Also reported:
> Dailymail.co.uk (2), Dailymail.co.uk (3)
>
> (KO
mention)
>
>
> Authority: 10
>
> Shares: Shared 31, Comments 49
>
>
Readership: c.

> 2,912,000
>
> Action: FYI
>
>
>
> Industry
News
>
>
>
>
> Nutritional Measures
>
>
>
> US Fizzy Drink Tax
Proposal (France)
>
>
>
>
> Nytimes.com – 'Republicans Push Back
Against Proposed Dietary
> Guidelines', 24.06.15
>
>
>
> Reports on
political debates in the US
> House and Senate regarding nutrition and diet
regulations. It is noted that an
> advisory panel of independent doctors and
nutrition experts have suggested a tax
> on sugar-sweetened drinks and snacks
as means to encourage better eating habits.
> The publication underscores that
the idea angered beverage companies and
> conservatives in Congress.
>
> (No
KO mention)
>
> Authority: 10
>
>

> Shares: N/A
>
> Readership: c.
3,072,000
>
> Action: FYI
>
>
>
>
> VAT Increase (Greece)
>
>

>
> Madata.gr – 'Τα προϊόντα και οι
> υπηρεσίες που θα ακριβύνουν με το νέο
ΦΠΑ', 24.06.15
>
>
>
> Reports
> on the new VAT regime in Greece,
which will increase the rate to 23 per cent. It
> is noted that the increase
will affect goods such as soft drinks and juice,
> sugar, coffee, chocolates as
well as all processed and canned foods.
>
>
> Also reported: Lay-out.gr,
Newpost.gr, Karfitsa.gr, Lefimerida.gr,
> Skaipatras.gr, Aftodioikisi.gr
>
>
(No KO mention)
>
> Authority: 2
>
>
> Shares: Twitter 1, Facebook 2
>

> Readership: c. 12,600
>
> Action:

> FYI
>
>
>
> Jamie Oliver's
Sugar Tax (Belgium, UK, France)
>
>
>
>
> Nieuwsblad.be – 'Jamie
Oliver heft belasting op frisdranken',
> 24.06.15
>
>
>
> Reports
that British celebrity chef Jamie Oliver is
> going to implement a "soda tax"
on all sugary drinks available in his
> restaurants in hopes that the
government will also implement such a tax. It is
> stated that the extra
revenue from the price increase will be invested in
> teaching children about
healthy eating. A UK publication reports that a Tory
> backbencher has
commented on Oliver's move, noting that he is against a sugar
> tax and that
there are "far more dangerous white powders." Another publication
> reports
that the Food and Drink Federation has welcomed Oliver's commitment to
>
improving public health, but has insisted that a real sugar tax would fail as
>
such measures have reportedly proven ineffective.
>
> Also reported:
>
Meltyfood.fr, Atabula.com, Homemadebyyou.co.uk, Foodmanufacture.co.uk,
>
>

> Atabula.com, Nieuws.be, Hbvl.be, Foodmanufacture.co.uk, Lexpress.fr,
>
Agripress.be,

>
> Standaard.be, Planet.fr, Bristolpost.co.uk,
>
Foodmanufacture.co.uk
>
> (No KO mention)
>
> Authority: 3
>
>
>
Shares: 11
>
> Readership: c. 70,400
>
> Action: Local Pac teams to
>
monitor
>
>
>
> WHO Proposals on Soft Drinks (UK, Spain)
>
>
>

>
> Just-drinks.com – 'World Health Organization calls for full-sugar
soft
> drinks regulations', 24.06.15
>
> By Keith Nuthall
>
>
>
>

> Reports that the Director General of the World Health Organization (WHO)
has
> accused marketers of full sugar non-alcoholic beverages of contributing
to
> rising obesity rates amongst children globally, especially in
developing
> countries. Dr Margaret Chan is also backing regulations that would
restrict the
> consumption of full sugar soft drinks, noting that voluntary

initiatives are
> less likely to be enough. A Spanish publication reports that the introduction of
> a tax on sugar-sweetened drinks in Mexico has resulted in a 12 per cent
> reduction of consumption of these beverages. It is also noted that in 2012, the
> Catalonian Parliament approved a bill mandating a tax on sugar-sweetened drinks,
> but it was never implemented.
>
> Also reported:
Consalud.es,
> Cronicanorte.es
>
> (No KO mention)
>
> Authority:
1
>
>
> Shares: 9
>
> Readership: c. 1,400
>
> Action: Local PAC
teams to
> monitor
>
>
>
> Sugar and Sweeteners
>
>
>
> Sugar
and
> Health (Spain, France)
>
>
>
> Consumer.es – '¿Cuánta azúcar
debemos
> consumir?', 24.06.15
>
> By Isabel Megías

>
>
>
>
Reports that
> sugar is becoming more prevalent in our diets due to quantities
now found in
> everyday products. The article also mentions guidelines on daily
sugar intake
> from the World Health Organization (WHO) for adults and
children. Illnesses
> associated with overconsumption of sugar, such as
obesity, hypertension, high
> cholesterol and vascular diseases, are also
discussed. Additional coverage also
> discusses the prevalence of hidden sugars
in everyday foods.
>
> Also
> reported: Femmeactuelle.fr
>
> (No KO
mention)
>
> Authority: 5
>
>
> Shares: 0
>
> Readership: c.
28,400
>
> Action: FYI
>
>
>
>
> Soft Drinks and Health
>
>

>
> Coca-Cola Life (Germany)
>
>
>

>
> Badische-zeitung.de –
'Ist grüne Coke gesünder?', 24.06.15
>
> By
> Claudia Füßler
>
>
>

> Discusses Coca-Cola Life, noting that even
> though the drink is partly
sweetened with stevia, has a lower calorie content
> than regular Coke and is
marketed as a healthier alternative, it still contains
> more sugar in a
half-litre bottle than the daily limit recommended by the World
> Health
Organization. A nutritionist is cited as commenting that Coca-Cola Life
> is
still a "sugar bomb" despite its lower sugar content.
>
> (KO mention:
>
Coca-Cola Life)
>
> Authority: N/A
>
> Shares: 0
>
> Readership: c.
>
19,100
>
> Action: Germany PAC team to monitor
>
>
>
> Fizzy
>
Drinks and Health (Spain, Ireland, Denmark, UK)
>
>
>
>
>

Eleconomista.es – 'La erosión dental aumenta en verano por el consumo de
>
bebidas azucaradas y la exposición al cloro', 24.06.15
>
>
>
>
>
Reports on factors that can negatively impact dental health during the summer
>
months, including consumption of carbonated soft drinks and other products with
> high sugar content such as ice-cream. It is claimed that these products increase
> the risk of dental cavities. An Irish publication reports that despite the
> alleged health benefits of smoothies, they may also have a detrimental effect to
> human health. Celebrity dentist, Dr Uchenna Okoye is quoted as arguing that
> fructose found in most fruit damages tooth enamel, which in turn leads to decay.
> A Danish source comments on the negative effect of phosphoric acid contained in
> cola drinks does to calcium in bones. A UK publication quotes Professor Colin
> Hill, a microbiologist at University College Cork in Ireland, as stating that
> "no intervention will overcome someone drinking lots of fizzy drinks."
>
>
> Also reported: Diariosigloxxi.com, Larazon.es, Her.ie, Samvirke.dk,
> Dailymail.co.uk
>
>
(No KO mention)
>
> Authority: 4
>
> Shares: 3
>
>
> Readership: c. 126,000

>
> Action: Local PAC teams to monitor
>
>
>
>
>
Methodology
>
>
>
> Authority – Authority score is provided by
>
Sysomos. Each data source is assigned a score between 0 (lowest) – 10
(highest).
> Authority is measured using a number of factors including in-link
count and
> reach for traditional media and in-link count, comments, reach and
bookmarks for
> blogs. Where a specific source is retrieved using another
platform 'N/A' will be
> assigned.
>
>
>
> Shares - This data is taken
from the publication
> itself, and includes the number of shares across
platforms. Shares include
> recommendations on Facebook and Twitter and
comments on the article in question.
> When this information is not provided,
'N/A' is shown. The type of shares will
> be broken down by platform when the
total sum is greater than 10.
>
>
>
>
> Readership – Readership gives
an indication of the size of audience reached
> and is based on a unique
visitors estimate by Siteworthtraffic.com, which uses
> data by web analysis
platform Alexa.
>

>
>
>
>
>
>
>
>

>
>
>
CONFIDENTIALITY NOTICE
> NOTICE: This message is intended for the use of the
>
individual or entity to which it is addressed and may contain information
that
> is confidential, privileged and exempt from disclosure under applicable
law. If
> the reader of this message is not the intended recipient, you are
hereby
> notified that any printing, copying, dissemination, distribution,
disclosure or
> forwarding of this communication is strictly prohibited. If you
have received
> this communication in error, please contact the sender
immediately and delete it
> from your system. Thank You.
>
>
>
>

>
>
>
>
>

>
