# Supplementary material for: Public Meets Private: Conversations Between Coca‐Cola and the CDC
Source: Milbank Q. 2019 Jan 29;97(1):74–90. doi: 10.1111/1468-0009.12368 (PMC6422605; doi:10.1111/1468-0009.12368)
Supplement: Supplementary file 3 — Responses from the CDC to our FOIA request dated January 27, 2017, regarding communications between CDC employees Maureen Culbertson, Kevin Ryan, Leandris Liburd, and Deborah Galuska and (1) the Coca‐Cola Company, (2) Rhona Applebaum, (3) Alex Malaspina, and (4) the International Life Sciences Institute (January 1, 2011‐present). [file MILQ-97-74-s003.pdf]

Subject: FOIA REQUEST
Attachments: image001.jpg

From: Susan A. Roberts [mailto:suroberts@coca-cola.com]
Sent: Wednesday, June 25, 2014 10:07 AM
To: Collins, Janet L. (CDC/ONDIEH/NCCDPHP) <jlc1@cdc.gov>
Cc: Beate Lloyd <belloyd@coca-cola.com>; Ryan, Kevin A. (CDC/ONDIEH/NCCDPHP) <kir0@cdc.gov>; Liburd, Leandris C. (CDC/OD/OMHHE) <lel1@cdc.gov>; Karima Kendall <kakendall@coca-cola.com>; Galuska, Deborah A. (CDC/ONDIEH/NCCDPHP) <dbg6@cdc.gov>
Subject: Re: Thank you.

Janet, thank you.
Deb, feel free to reach our to me to discuss any of this or to learn more about our current research efforts in this area.
Best wishes, Susan

On Jun 25, 2014, at 8:48 AM, "Collins, Janet L. (CDC/ONDIEH/NCCDPHP)" <jlc1@cdc.gov> wrote:

> Thank you so much for these resources. I am copying our Associate Director of Science, Deb Galuska, to help us make optimal use of these resources.
>
> Thanks!
> Janet
>
> Janet Collins, Ph.D.
> Director, Division of Nutrition, Physical Activity, and Obesity
> National Center for Chronic Disease Prevention and Health Promotion, CDC
> 770-488-6042 (w); 404-409-0522 (c); jcollins@cdc.gov
>
> <image001.jpg>
>
> ---
>
> **From:** Susan A. Roberts [mailto:suroberts@coca-cola.com]
> **Sent:** Tuesday, June 24, 2014 10:27 PM
> **To:** Beate Lloyd; Collins, Janet L. (CDC/ONDIEH/NCCDPHP); Ryan, Kevin A. (CDC/ONDIEH/NCCDPHP); Liburd, Leandris C. (CDC/OD/OMHHE)
> **Cc:** Karima Kendall
> **Subject:** RE: Thank you.
>
> Dear Janet, Kevin and Leandris,
> I was very glad to be able to participate in the meeting by phone on Friday and also look forward to on-going discussions with you to identify areas of potential collaboration and information sharing.
> In follow up to Beate's note below, please find attached two recent publications on LNCS as well as a draft manuscript on heterogeneity in meta-analyses with suggested approaches to help the non-expert better interpret the outcome statistic.

1) Low-calorie sweeteners vs water during weight loss study (Jim Hill from Univ of Colorado and Gary Foster from Temple Univ, both former presidents of The Obesity Society, were the co-PIs). As mentioned on the call, this is phase I of a two-part study. Phase 2 , the maintenance phase (additional 9 months) will be completed by end of year. Leandris, this is the paper I sent to you on Friday. I have also attached the editorial associated with the paper for an additional perspective. We plan to address many of the outstanding questions of mechanism/appetite in future studies.
2) Meta-analysis of low-calorie sweeteners and weight – the analysis is done on both RCTs and prospective population studies. The findings show a modest but significant benefit of low-calorie sweeteners in weight loss and the effect is largely driven by the beverage studies. It also provides additional support for the view that the positive associations between diet beverages and weight in the epidemiological studies is likely the result of reverse causality.
3) The heterogeneity methods paper. This paper has been accepted in the journal Systematic Reviews. Therefore please treat as confidential until it is published.

I would be glad to discuss any of this further.

Best wishes, Susan

From: Beate Lloyd
Sent: Tuesday, June 24, 2014 6:39 PM
To: jcollins@cdc.gov; kir0@cdc.gov; Liburd, Leandris C. (CDC/OD/OMHHE)
Cc: Susan A. Roberts; Karima Kendall
Subject: Thank you.

Dear Janet,
I would like to thank you, Kevin and Leandris very much for taking the time to meet with us. It was very helpful to understand your areas of focus and where we may have mutual interests. There are clearly areas where we can work collaboratively and share insights to advance the work in prevention of obesity and inform of the consumer of choices.

We valued getting to know you and your team better and enjoyed the rich discussion relating to your main initiatives. Susan will be sharing with you further the work on the low and no calorie beverage research and will follow-up as more of the data become publically available. We can also forward the papers on the scientific method and interpretation of the epidemiological studies as per our discussion and impact of heterogeneity.

It would be helpful to have another meeting in the future to follow-up on the key discussions on methods and interventions, especially with regards to the fortification programs and grocery channels. Thank you again very much for your interest and time.

Warmest regards,
Beate

Beate B. Lloyd PhD RD LD

Senior Director Nutrition COE
Global Scientific and Regulatory Affairs
The Coca-Cola Company
P: (404) 676-2149
C: (404) 545-3141

CONFIDENTIALITY NOTICE
NOTICE: This message is intended for the use of the individual or entity to which it is addressed and may contain information that is confidential, privileged and exempt from disclosure under applicable law. If the reader of this message is not the intended recipient, you are hereby notified that any printing, copying, dissemination, distribution, disclosure or forwarding of this communication is strictly prohibited. If you have received this communication in error, please contact the sender immediately and delete it from your system. Thank You.

CONFIDENTIALITY NOTICE
NOTICE: This message is intended for the use of the individual or entity to which it is addressed and may contain information that is confidential, privileged and exempt from disclosure under applicable law. If the reader of this message is not the intended recipient, you are hereby notified that any printing, copying, dissemination, distribution, disclosure or forwarding of this communication is strictly prohibited. If you have received this communication in error, please contact the sender immediately and delete it from your system. Thank You.

---

25 HEALTHY LIVING FOR ALL YEARS

National Center for
Chronic Disease Prevention
and Health Promotion

From: Liburd, Leandris C. (CDC/OD/OMHHE)
Sent: 5 Feb 2017 08:29:11 -0500
To: Coleman, Sharon R. (CDC/OD/OMHHE)
Subject: FW: 1-to-1 agenda items
Attachments: Liburd_06 20 2014_Individual meeting.docx

From: Liburd, Leandris C. (CDC/OD/OMHHE)
Sent: Friday, June 20, 2014 11:55 AM
To: Arias, Ileana (CDC/OD) <iaa4@cdc.gov>
Subject: 1-to-1 agenda items

Ileana,

I am aware that our meeting today is canceled due to the anthrax incident. I am forwarding the notes for our meeting so that you will have them in advance.

Leandris

Leandris C. Liburd, PhD, MPH
Associate Director for Minority Health and Health Equity
Office of Minority Health and Health Equity
Centers for Disease Control and Prevention
4770 Buford Hwy, Mailstop K-77
Atlanta, GA 30341
770-488-8343
770-488-8160 (FAX)

CDC's OFFICE OF MINORITY HEALTH & HEALTH EQUITY 25 YEARS
A CHAMPION FOR HEALTH EQUITY

OMHHE Updates

June 20, 2014

Meeting (June 20, 2014) with Coca Cola Representatives and Janet Collins (NCCDPHP).

Attendees expected are:

1) Beate Lloyd, PhD, RD, LD, Senior Director Nutrition COE, Global Scientific and Regulatory Affairs
2) Susan Roberts PhD, Director of Nutrition in Global Scientific and Regulatory Affairs, (via phone)
3) Robert Earl MS RD, Director of Policy in Global Scientific and Regulatory Affairs
4) Karima Kendall PhD RD, Nutrition Intern in Global Scientific and Regulatory Affairs
5) Mary Hall, OMHHE

- 2015 CDC Health Disparities and Inequalities Report (biennial MMWR supplement)

2015 CDC Health Disparities and Inequalities Report – update

Ana Penman-Aguilar and I met with Chesley Richards, Michael Iademarco, and Shawna Mercer to discuss the status/progress toward development of the 2015 CHDIR.

Key findings:

- Of the staff involved in coordinating the 2013 CHDIR, no one is left in CSELS to lead/oversee/coordinate the 2015 report. We discussed at length pros and cons of continuing to produce the report (methodological issues, utility, efficiency, etc.). The meeting ended with the expectation that we would consider "options" for the CHDIR. See options below -

- Possible options to ensure the report is released in 2015 per OMHHE:

Option A:

A report and a website both created/updated biennially. The report includes only 10 topics – with abbreviated narratives for most topics. The website is a place where people can go for information on the landscape of ~30 important topics (of which the 10 are a subset).

This is how it would work:

- Data analyses for all 30 topics are conducted biennially. This is the monitoring function of CDC. Looking at all 30 provides the landscape.

- Only 10 topics are in the biennial report, however! (Rotate through the 30 topics on a 6 year cycle – 10 in year 1, 10 in year 3, 10 in year 5 – revert to the initial 10 in year 7)

- The expectations of the narratives for the 10 topics will vary depending on the findings. If there are no changes in the disparities, there will be a very brief accompanying narrative. Topics where things are getting better or worse will have longer, more in-depth narratives explaining opportunities and reasons for action.

- The findings for the 20 topics that are not featured in the biennial report would be updated on the web only – with minimal explanatory text.

Option B is status quo.

We couldn't think of an Option C.

Possible options per CSELS:

- Convert the MMWR to a Compendium or smaller product, i.e., instead of 30 indicators, reduce to the top 5 leading indicators for health disparities and inequalities
- Put the data on the Website and let the user extrapolate specific analyses or data points
- Vital Signs – replace the MMWR with a Vital Signs on health disparities and inequalities

Reactions from OMHHE and other CDC scientists who were consulted about these options:

We considered a website without interpretive essays insufficient. We don't want to transfer the burden of interpretation to the reader. Worse, if the website consists of a query system only, we will be transferring the burden of interpretation <u>and analysis t</u>o the reader. Since there are gaps in many people's understanding of how to think about health disparities (including many epidemiologists lacking acquaintance with the principles underlying the attached presentation by Makram Talih at the 2013 State of Health Equity forum), this would not educate readers or advance the science.

Additional comments:

- There may be a downside to attempts at centralizing all the work in CSELS or some other CDC/OD entity in the interest of standardizing the methods and write-ups too much – hinders creativity and limits the benefit of the authors' SME, authors may be less excited about the work…

- CHDIR methods generally sufficient to know if things are getting better, worse, or staying bad in order to motivate action. However, one important improvement would be to more often look at intersections between characteristics (e.g. race/ethnicity and SE factors) when this is appropriate and feasible. (Better representation of various populations and subpops in the analyses is important too but that is more of a data issue – not methods per se). So we don't see a pressing need for a data methods group at this time. Such a group should be formed when the team who will create the CHDIR has been identified.

Principle OMHHE concerns:

- All indications are that CSELS does not currently have the capacity to produce the 2015 report, and neither does OMHHE. We need assistance in deciding where the organizational responsibility for this data report should reside, and then identifying the resources for getting it done.

2015 Hispanic Health Vital Signs

- **On track!**
- Ken Dominguez (NCHHSTP) is approved to devote 6 months to completing this Vital Signs (May 2015) and his detail to OMHHE will begin in November 2014.

2015 MMWR Report on strategies that are working to reduce health disparities

- On track!
- I will share the abstracts of the proposed strategies during one of our July 1-to-1 meetings

Updates for July meetings

- HDS recommendations
- Health Equity Framework for Action – supplement proposal
-

From: Liburd, Leandris C. (CDC/OD/OMHHE)
Sent: 5 Feb 2017 08:36:14 -0500
To: Coleman, Sharon R. (CDC/OD/OMHHE)
Subject: FW: Accepted: Conference call with Susan Roberts - Coca Cola @ Mon Sep 9, 2013 6pm - 7pm (cdastra066@gmail.com)
Attachments: invite.ics

From: Liburd, Leandris C. (CDC/OD/OMHHE)
Sent: Monday, September 09, 2013 2:31 PM
To: cdastra066@gmail.com
Subject: FW: Accepted: Conference call with Susan Roberts - Coca Cola @ Mon Sep 9, 2013 6pm - 7pm (cdastra066@gmail.com)

-----Original Appointment-----
From: Google Calendar [mailto:calendar-notification@google.com] **On Behalf Of** Carlton Duncan
Sent: Monday, September 09, 2013 1:15 PM
To: Liburd, Leandris C. (CDC/OD/OMHHE)
Subject: Accepted: Conference call with Susan Roberts - Coca Cola @ Mon Sep 9, 2013 6pm - 7pm (cdastra066@gmail.com)
When: Monday, September 09, 2013 2:00 PM-3:00 PM (UTC-05:00) Eastern Time (US & Canada).
Where: Bridgeline: (b)(6) Passcode: (b)(6)

Carlton Duncan has accepted this invitation.

Conference call with Susan Roberts - Coca Cola

Leandris, the leader code is: (b)(6)
This meeting is being rescheduled at Susan's request.

When Mon Sep 9, 2013 6pm – 7pm GMT (no daylight saving)

Where Bridgeline: (b)(6) Passcode (b)(6) (map)

Calendar	cdastra066@gmail.com
Who	• Liburd, Leandris C. (CDC/OD/OMHHE) - organizer<br>• Carlton Duncan - creator, optional<br>• Duncan, Carlton (CDC/OCOO)

Invitation from Google Calendar

You are receiving this courtesy email at the account lel1@cdc.gov because you are an attendee of this event.

To stop receiving future notifications for this event, decline this event. Alternatively you can sign up for a Google account at https://www.google.com/calendar/ and control your notification settings for your entire calendar.

From: Liburd, Leandris C. (CDC/OD/OMHHE)
Sent: 5 Feb 2017 08:35:43 -0500
To: Coleman, Sharon R. (CDC/OD/OMHHE)
Subject: FW: Accepted: Conference call with Susan Roberts - Coca Cola @ Mon Sep 9, 2013 6pm - 7pm (cdastra066@gmail.com)
Attachments: invite.ics

From: Baker, Gwendolyn (CDC/OD/OMHHE) **On Behalf Of** Liburd, Leandris C. (CDC/OD/OMHHE)
Sent: Monday, September 09, 2013 3:12 PM
To: Liburd, Leandris C. (CDC/OD/OMHHE) <lel1@cdc.gov>
Cc: C Md (cdastra066@gmail.com) <cdastra066@gmail.com>
Subject: FW: Accepted: Conference call with Susan Roberts - Coca Cola @ Mon Sep 9, 2013 6pm - 7pm (cdastra066@gmail.com)

Leandris, just FYI, I left to run an errand at 1:15 PM when this came in. I didn't see it until I returned at 2:05 PM when the call was supposed to have started. I'll contact her and reschedule it.

Gwen

-----Original Appointment-----
From: Google Calendar [mailto:calendar-notification@google.com] **On Behalf Of** Carlton Duncan
Sent: Monday, September 09, 2013 1:15 PM
To: Green, Sheneta Marchet (CDC/OD/OMHHE) (CTR); Baker, Gwendolyn (CDC/OD/OMHHE)
Subject: Accepted: Conference call with Susan Roberts - Coca Cola @ Mon Sep 9, 2013 6pm - 7pm (cdastra066@gmail.com)
When: Monday, September 09, 2013 2:00 PM-3:00 PM (UTC-05:00) Eastern Time (US & Canada).
Where: Bridgeline (b)(6) Passcode: (b)(6)

Carlton Duncan has accepted this invitation.

Conference call with Susan Roberts - Coca Cola

Leandris, the leader code is (b)(6)
This meeting is being rescheduled at Susan's request.

When Mon Sep 9, 2013 6pm – 7pm GMT (no daylight saving)

Where Bridgeline: (b)(6) Passcode: (b)((map)

Calendar	cdastra066@gmail.com	
Who	• Liburd, Leandris C. (CDC/OD/OMHHE) - organizer<br>• Carlton Duncan - creator, optional<br>• Duncan, Carlton (CDC/OCOO)	

Invitation from Google Calendar

You are receiving this courtesy email at the account lel1@cdc.gov because you are an attendee of this event.

To stop receiving future notifications for this event, decline this event. Alternatively you can sign up for a Google account at https://www.google.com/calendar/ and control your notification settings for your entire calendar.

From: Liburd, Leandris C. (CDC/OD/OMHHE)
Sent: 5 Feb 2017 08:26:17 -0500
To: Coleman, Sharon R. (CDC/OD/OMHHE)
Subject: FW: Confirming meeting at CDC

From: Liburd, Leandris C. (CDC/OD/OMHHE)
Sent: Thursday, June 19, 2014 9:55 AM
To: Brown, Andrea (CDC/ONDIEH/NCCDPHP) (CTR) <bql2@cdc.gov>; Collins, Janet L. (CDC/ONDIEH/NCCDPHP) <jlc1@cdc.gov>
Cc: Green, Sheneta Marchet (CDC/OD/OMHHE) (CTR) <xdy8@cdc.gov>
Subject: RE: Confirming meeting at CDC

Thanks, Andrea... I will let them know, and suggest a conference call option for Dr. Roberts. Leandris

---

From: Brown, Andrea (CDC/ONDIEH/NCCDPHP) (CTR)
Sent: Thursday, June 19, 2014 8:01 AM
To: Collins, Janet L. (CDC/ONDIEH/NCCDPHP)
Cc: Liburd, Leandris C. (CDC/OD/OMHHE); Brown, Andrea (CDC/ONDIEH/NCCDPHP) (CTR)
Subject: RE: Confirming meeting at CDC

It looks like I will not be able to get Dr. Susan Roberts on the visitor's list because it require 3wks to process international visitors.

Andrea

From: Collins, Janet L. (CDC/ONDIEH/NCCDPHP)
Sent: Wednesday, June 18, 2014 5:01 PM
To: Brown, Andrea (CDC/ONDIEH/NCCDPHP) (CTR)
Cc: Liburd, Leandris C. (CDC/OD/OMHHE)
Subject: Fw: Confirming meeting at CDC

Just fyi in case we need to take steps to get them on campus. Leandris may have arranged. I'm not sure of details.
Thx
Janet

Sent from my BlackBerry 10 smartphone.
From: Liburd, Leandris C. (CDC/OD/OMHHE)
Sent: Wednesday, June 18, 2014 3:31 PM
To: Collins, Janet L. (CDC/ONDIEH/NCCDPHP)
Subject: FW: Confirming meeting at CDC

FYI...

From: Beate Lloyd [mailto:belloyd@coca-cola.com]
Sent: Wednesday, June 18, 2014 4:16 PM

To: Liburd, Leandris C. (CDC/OD/OMHHE)
Cc: Green, Sheneta Marchet (CDC/OD/OMHHE) (CTR); Hall, Mary E. (CDC/OD/OMHHE); Mechelle Crawford
Subject: RE: Confirming meeting at CDC

Dear Leandris,
We are very much looking forward to coming to see you and Janet this Friday. I will be accompanied by the following colleagues:

1) Susan Roberts PhD, Director of Nutrition in Global Scientific and Regulatory Affairs, whom you have met,
2) Robert Earl MS RD, Director of Policy in Global Scientific and Regulatory Affairs
3) Karima Kendall PhD RD, Nutrition Intern in Global Scientific and Regulatory Affairs

Susan Roberts is a (b)(6) by passport, is she required to complete any special forms to participate in the meeting?

Please let me know, if you have any questions or think a brief call is needed prior to our meeting. We look forward to this great opportunity to meet with you.

Warmest regards,
Beate

Beate B. Lloyd PhD RD LD
Senior Director Nutrition COE
Global Scientific and Regulatory Affairs
The Coca-Cola Company
P: (404) 676-2149
C: (404) 545-3141

From: Liburd, Leandris C. (CDC/OD/OMHHE) [mailto:lel1@cdc.gov]
Sent: Saturday, June 07, 2014 8:01 PM
To: Beate Lloyd
Cc: Green, Sheneta Marchet (CDC/OD/OMHHE) (CTR); Hall, Mary E. (CDC/OD/OMHHE)
Subject: Confirming meeting at CDC

Hi Beate,

I am writing to confirm the meeting at CDC for June 20, 2014 at 1:30 pm. The meeting will be held on the Chamblee campus in Building 107 in Conference Room 5A.

The CDC Chamblee campus is located at 4770 Buford Hwy, Atlanta, GA 30341.

If you questions, please don't hesitate to contact me or Sheneta Green copied above…

Looking forward to seeing you…

Best, Leandris

From: Beate Lloyd [mailto:belloyd@coca-cola.com]
Sent: Thursday, May 29, 2014 6:42 PM
To: Liburd, Leandris C. (CDC/OD/OMHHE)
Subject: RE: Leandris Liburd and Beate Lloyd

Thank you. I will be glad to move this meeting.
Kind regards,
Beate

From: Liburd, Leandris C. (CDC/OD/OMHHE) [mailto:lel1@cdc.gov]
Sent: Thursday, May 29, 2014 6:22 PM
To: Beate Lloyd
Subject: RE: Leandris Liburd and Beate Lloyd

Hi Beate,

No worries, I certainly understand how these things go… Tomorrow I have meetings until 3:00 pm, but am available after that. Would 3:30 pm work for you? Let me know and we can talk then.

My office number is 770-488-8180 and my cell number is (b)(6)

Thanks, Leandris

-----Original Appointment-----
From: Beate Lloyd [mailto:belloyd@coca-cola.com]
Sent: Thursday, May 29, 2014 6:10 PM
To: Liburd, Leandris C. (CDC/OD/OMHHE)
Subject: Leandris Liburd and Beate Lloyd
When: Friday, May 30, 2014 10:00 AM-10:30 AM (UTC-05:00) Eastern Time (US & Canada).
Where: Beate to call Leandris at 770-488-8343

Dear Leandris,
We had visitors from the UK that were meeting with us. I am sorry I missed calling you back while you were still in the office. Would this proposed time on Friday work for you? If not please let me know and I can find another time.

Sincerely,
Beate

CONFIDENTIALITY NOTICE
NOTICE: This message is intended for the use of the individual or entity to which it is addressed and may contain information that is confidential, privileged and exempt from disclosure under applicable law. If the reader of this message is not the intended recipient, you are hereby notified that any printing, copying, dissemination, distribution, disclosure or forwarding of this communication is strictly prohibited. If you have received this communication in error, please contact the sender immediately and delete it from your system. Thank You.

CONFIDENTIALITY NOTICE
NOTICE: This message is intended for the use of the individual or entity to which it is addressed and may contain information that is confidential, privileged and exempt from disclosure under applicable law. If the reader of this message is not the intended recipient, you are hereby notified that any printing, copying, dissemination, distribution, disclosure or forwarding of this communication is strictly prohibited. If you have received this communication in error, please contact the sender immediately and delete it from your system. Thank You.

CONFIDENTIALITY NOTICE
NOTICE: This message is intended for the use of the individual or entity to which it is addressed and may contain information that is confidential, privileged and exempt from disclosure under applicable law. If the reader of this message is not the intended recipient, you are hereby notified that any printing, copying, dissemination, distribution, disclosure or forwarding of this communication is strictly prohibited. If you have received this communication in error, please contact the sender immediately and delete it from your system. Thank You.

From: Liburd, Leandris C. (CDC/OD/OMHHE)
Sent: 5 Feb 2017 08:27:10 -0500
To: Coleman, Sharon R. (CDC/OD/OMHHE)
Subject: FW: Confirming meeting at CDC

From: Liburd, Leandris C. (CDC/OD/OMHHE)
Sent: Wednesday, June 18, 2014 4:31 PM
To: Collins, Janet L. (CDC/ONDIEH/NCCDPHP) <jlc1@cdc.gov>
Subject: FW: Confirming meeting at CDC

FYI…

From: Beate Lloyd [mailto:belloyd@coca-cola.com]
Sent: Wednesday, June 18, 2014 4:16 PM
To: Liburd, Leandris C. (CDC/OD/OMHHE)
Cc: Green, Sheneta Marchet (CDC/OD/OMHHE) (CTR); Hall, Mary E. (CDC/OD/OMHHE); Mechelle Crawford
Subject: RE: Confirming meeting at CDC

Dear Leandris,
We are very much looking forward to coming to see you and Janet this Friday. I will be accompanied by the following colleagues:

1) Susan Roberts PhD, Director of Nutrition in Global Scientific and Regulatory Affairs, whom you have met,
2) Robert Earl MS RD, Director of Policy in Global Scientific and Regulatory Affairs
3) Karima Kendall PhD RD, Nutrition Intern in Global Scientific and Regulatory Affairs

Susan Roberts is a (b)(6) by passport, is she required to complete any special forms to participate in the meeting?

Please let me know, if you have any questions or think a brief call is needed prior to our meeting. We look forward to this great opportunity to meet with you.

Warmest regards,
Beate

Beate B. Lloyd PhD RD LD
Senior Director Nutrition COE
Global Scientific and Regulatory Affairs
The Coca-Cola Company
P: (404) 676-2149
C: (404) 545-3141

From: Liburd, Leandris C. (CDC/OD/OMHHE) [mailto:lel1@cdc.gov]
Sent: Saturday, June 07, 2014 8:01 PM
To: Beate Lloyd
Cc: Green, Sheneta Marchet (CDC/OD/OMHHE) (CTR); Hall, Mary E. (CDC/OD/OMHHE)
Subject: Confirming meeting at CDC

Hi Beate,

I am writing to confirm the meeting at CDC for June 20, 2014 at 1:30 pm. The meeting will be held on the Chamblee campus in Building 107 in Conference Room 5A.

The CDC Chamblee campus is located at 4770 Buford Hwy, Atlanta, GA 30341.

If you questions, please don't hesitate to contact me or Sheneta Green copied above…

Looking forward to seeing you…

Best, Leandris

From: Beate Lloyd [mailto:belloyd@coca-cola.com]
Sent: Thursday, May 29, 2014 6:42 PM
To: Liburd, Leandris C. (CDC/OD/OMHHE)
Subject: RE: Leandris Liburd and Beate Lloyd

Thank you. I will be glad to move this meeting.
Kind regards,
Beate

From: Liburd, Leandris C. (CDC/OD/OMHHE) [mailto:lel1@cdc.gov]
Sent: Thursday, May 29, 2014 6:22 PM
To: Beate Lloyd
Subject: RE: Leandris Liburd and Beate Lloyd

Hi Beate,

No worries, I certainly understand how these things go… Tomorrow I have meetings until 3:00 pm, but am available after that. Would 3:30 pm work for you? Let me know and we can talk then.

My office number is 770-488-8180 and my cell number is (b)(6)

Thanks, Leandris

-----Original Appointment-----
From: Beate Lloyd [mailto:belloyd@coca-cola.com]

Sent: Thursday, May 29, 2014 6:10 PM
To: Liburd, Leandris C. (CDC/OD/OMHHE)
Subject: Leandris Liburd and Beate Lloyd
When: Friday, May 30, 2014 10:00 AM-10:30 AM (UTC-05:00) Eastern Time (US & Canada).
Where: Beate to call Leandris at 770-488-8343

Dear Leandris,
We had visitors from the UK that were meeting with us. I am sorry I missed calling you back while you were still in the office. Would this proposed time on Friday work for you? If not please let me know and I can find another time.

Sincerely,
Beate

CONFIDENTIALITY NOTICE
NOTICE: This message is intended for the use of the individual or entity to which it is addressed and may contain information that is confidential, privileged and exempt from disclosure under applicable law. If the reader of this message is not the intended recipient, you are hereby notified that any printing, copying, dissemination, distribution, disclosure or forwarding of this communication is strictly prohibited. If you have received this communication in error, please contact the sender immediately and delete it from your system. Thank You.

CONFIDENTIALITY NOTICE
NOTICE: This message is intended for the use of the individual or entity to which it is addressed and may contain information that is confidential, privileged and exempt from disclosure under applicable law. If the reader of this message is not the intended recipient, you are hereby notified that any printing, copying, dissemination, distribution, disclosure or forwarding of this communication is strictly prohibited. If you have received this communication in error, please contact the sender immediately and delete it from your system. Thank You.

CONFIDENTIALITY NOTICE
NOTICE: This message is intended for the use of the individual or entity to which it is addressed and may contain information that is confidential, privileged and exempt from disclosure under applicable law. If the reader of this message is not the intended recipient, you are hereby notified that any printing, copying, dissemination, distribution, disclosure or forwarding of this communication is strictly prohibited. If you have received this communication in error, please contact the sender immediately and delete it from your system. Thank You.

From: Liburd, Leandris C. (CDC/OD/OMHHE)
Sent: 5 Feb 2017 08:25:31 -0500
To: Coleman, Sharon R. (CDC/OD/OMHHE)
Subject: FW: Confirming meeting at CDC

From: Liburd, Leandris C. (CDC/OD/OMHHE)
Sent: Thursday, June 19, 2014 11:44 AM
To: Beate Lloyd <belloyd@coca-cola.com>
Cc: Green, Sheneta Marchet (CDC/OD/OMHHE) (CTR) <xdy8@cdc.gov>; Hall, Mary E. (CDC/OD/OMHHE) <moh4@cdc.gov>; Mechelle Crawford <meccrawford@coca-cola.com>; Brown, Andrea (CDC/ONDIEH/NCCDPHP) (CTR) <bql2@cdc.gov>
Subject: RE: Confirming meeting at CDC

Beate,

Given the security policies for CDC, we would have had to submit Susan's name to the security office 10 days prior to our meeting tomorrow. Unfortunately, we won't be able to get her cleared to come on the Chamblee campus in time for our meeting. We are arranging a conference call option so that she can participate by phone in the discussion.

If you are free at 4:00 pm today, let's have a brief call. I can be reached at 770-488-8180.

Thanks, Leandris

From: Beate Lloyd [mailto:belloyd@coca-cola.com]
Sent: Wednesday, June 18, 2014 4:16 PM
To: Liburd, Leandris C. (CDC/OD/OMHHE)
Cc: Green, Sheneta Marchet (CDC/OD/OMHHE) (CTR); Hall, Mary E. (CDC/OD/OMHHE); Mechelle Crawford
Subject: RE: Confirming meeting at CDC

Dear Leandris,
We are very much looking forward to coming to see you and Janet this Friday. I will be accompanied by the following colleagues:

1) Susan Roberts PhD, Director of Nutrition in Global Scientific and Regulatory Affairs, whom you have met,
2) Robert Earl MS RD, Director of Policy in Global Scientific and Regulatory Affairs
3) Karima Kendall PhD RD, Nutrition Intern in Global Scientific and Regulatory Affairs

Susan Roberts is a Canadian by passport, is she required to complete any special forms to participate in the meeting?

Please let me know, if you have any questions or think a brief call is needed prior to our meeting. We look forward to this great opportunity to meet with you.

Warmest regards,
Beate

Beate B. Lloyd PhD RD LD
Senior Director Nutrition COE
Global Scientific and Regulatory Affairs
The Coca-Cola Company
P: (404) 676-2149
C: (404) 545-3141

From: Liburd, Leandris C. (CDC/OD/OMHHE) [mailto:lel1@cdc.gov]
Sent: Saturday, June 07, 2014 8:01 PM
To: Beate Lloyd
Cc: Green, Sheneta Marchet (CDC/OD/OMHHE) (CTR); Hall, Mary E. (CDC/OD/OMHHE)
Subject: Confirming meeting at CDC

Hi Beate,

I am writing to confirm the meeting at CDC for June 20, 2014 at 1:30 pm. The meeting will be held on the Chamblee campus in Building 107 in Conference Room 5A.

The CDC Chamblee campus is located at 4770 Buford Hwy, Atlanta, GA 30341.

If you questions, please don't hesitate to contact me or Sheneta Green copied above…

Looking forward to seeing you…

Best, Leandris

From: Beate Lloyd [mailto:belloyd@coca-cola.com]
Sent: Thursday, May 29, 2014 6:42 PM
To: Liburd, Leandris C. (CDC/OD/OMHHE)
Subject: RE: Leandris Liburd and Beate Lloyd

Thank you. I will be glad to move this meeting.
Kind regards,
Beate

From: Liburd, Leandris C. (CDC/OD/OMHHE) [mailto:lel1@cdc.gov]
Sent: Thursday, May 29, 2014 6:22 PM
To: Beate Lloyd
Subject: RE: Leandris Liburd and Beate Lloyd

Hi Beate,

No worries, I certainly understand how these things go... Tomorrow I have meetings until 3:00 pm, but am available after that. Would 3:30 pm work for you? Let me know and we can talk then.

My office number is 770-488-8180 and my cell number is (b)(6)

Thanks, Leandris

-----Original Appointment-----
From: Beate Lloyd [mailto:belloyd@coca-cola.com]
Sent: Thursday, May 29, 2014 6:10 PM
To: Liburd, Leandris C. (CDC/OD/OMHHE)
Subject: Leandris Liburd and Beate Lloyd
When: Friday, May 30, 2014 10:00 AM-10:30 AM (UTC-05:00) Eastern Time (US & Canada).
Where: Beate to call Leandris at 770-488-8343

Dear Leandris,
We had visitors from the UK that were meeting with us. I am sorry I missed calling you back while you were still in the office. Would this proposed time on Friday work for you? If not please let me know and I can find another time.

Sincerely,
Beate

CONFIDENTIALITY NOTICE
NOTICE: This message is intended for the use of the individual or entity to which it is addressed and may contain information that is confidential, privileged and exempt from disclosure under applicable law. If the reader of this message is not the intended recipient, you are hereby notified that any printing, copying, dissemination, distribution, disclosure or forwarding of this communication is strictly prohibited. If you have received this communication in error, please contact the sender immediately and delete it from your system. Thank You.

CONFIDENTIALITY NOTICE
NOTICE: This message is intended for the use of the individual or entity to which it is addressed and may contain information that is confidential, privileged and exempt from disclosure under applicable law. If the reader of this message is not the intended recipient, you are hereby notified that any printing, copying, dissemination, distribution, disclosure or forwarding of this communication is strictly prohibited. If you have received this communication in error, please contact the sender immediately and delete it from your system. Thank You.

CONFIDENTIALITY NOTICE
NOTICE: This message is intended for the use of the individual or entity to which it is addressed and may contain information that is confidential, privileged and exempt from disclosure under applicable law. If the reader of this message is not the intended recipient, you are hereby notified that any printing, copying, dissemination, distribution, disclosure or forwarding of this communication is strictly prohibited. If you have received this communication in error, please contact the sender immediately and delete it from your system. Thank You.

From: Liburd, Leandris C. (CDC/OD/OMHHE)
Sent: 5 Feb 2017 08:31:16 -0500
To: Coleman, Sharon R. (CDC/OD/OMHHE)
Subject: FW: Draft Agenda for Meeting at Coca-Cola
Attachments: CDC Meeting Agenda Jan 7 2014.docx

From: Susan A. Roberts [mailto:suroberts@coca-cola.com]
Sent: Monday, January 06, 2014 3:18 PM
To: Liburd, Leandris C. (CDC/OD/OMHHE) <lel1@cdc.gov>
Subject: RE: Draft Agenda for Meeting at Coca-Cola

Leandris,
If there are areas where you see potential collaboration.
However, this may come out of our discussion.

Please let us know if from our end, there anything in particular you would like us to cover as well.
Here is an updated agenda including objectives. Kindly share with the others.
Thank you and looking forward to meeting you tomorrow,

Susan
832-283-5209 (cell)
404-676-1330 (office)

From: Liburd, Leandris C. (CDC/OD/OMHHE) [mailto:lel1@cdc.gov]
Sent: Monday, January 06, 2014 2:19 PM
To: Susan A. Roberts
Subject: RE: Draft Agenda for Meeting at Coca-Cola

Hi Susan,

Are there specific questions that you all are interested in having me address in my presentation?

Let me know…

Leandris

From: Susan A. Roberts [mailto:suroberts@coca-cola.com]
Sent: Thursday, January 02, 2014 4:42 PM
To: Liburd, Leandris C. (CDC/OD/OMHHE)
Cc: Mebane, Reggie (CDC/OD/OEEO); Tonney, Chloe (CDC cdcfoundation.org); Baker, Gwendolyn (CDC/OD/OMHHE)
Subject: RE: Draft Agenda for Meeting at Coca-Cola

Hi Leandris,
We will be set up to project a PowerPoint presentation, so that format works well.
Regarding coming to our offices:

The address is 1 Coca-Cola Plaza, Atlanta, GA 303013.
Please enter by the front gate which is on North Ave. The parking is below the main building from which you take the elevator up to the main floor where the receptionist will give you an ID badge. I will have everyone pre-registered so it should go smoothly enough. Also, please call me when you arrive and I will come down to receive you and take you to the meeting room (my office number is 404-676-1330 and cell 832-283-5209).

See you next week, Susan

From: Liburd, Leandris C. (CDC/OD/OMHHE) [mailto:lel1@cdc.gov]
Sent: Thursday, January 02, 2014 3:41 PM
To: Susan A. Roberts
Cc: Mebane, Reggie (CDC/OD/OEEO); Tonney, Chloe (CDC cdcfoundation.org); Baker, Gwendolyn (CDC/OD/OMHHE)
Subject: FW: Draft Agenda for Meeting at Coca-Cola

Hi Susan, and Happy New Year!

Yes, I enjoyed the holidays and can hardly believe we have ended one year and started another! It seems like time is passing quickly! I hope your Christmas was joyful as well!

Thanks for the agenda. It looks fine, and I am looking forward to meeting everyone at TCCC. I do have 2 questions – (1) are there particular parking arrangements that we need to be aware of, and (2) should I bring a PowerPoint presentation or handouts?

I look forward to seeing you next week… best, Leandris

From: Susan A. Roberts [mailto:suroberts@coca-cola.com]
Sent: Tuesday, December 31, 2013 3:51 PM
To: Liburd, Leandris C. (CDC/OD/OMHHE)
Subject: Draft Agenda for Meeting at Coca-Cola

Dear Leandris,
I hope you had a Merry Christmas and I wish you the best for 2014.
I have drafted an agenda for our meeting next Tuesday. Please let me know if this is what you were thinking. Feel free to make changes as you see fit.

Looking forward to our discussion and to meeting you in person, Susan

---

CONFIDENTIALITY NOTICE
NOTICE: This message is intended for the use of the individual or entity to which it is addressed and may contain information that is confidential, privileged and exempt from disclosure under applicable law. If the reader of this message is not the intended recipient, you are hereby notified that any printing, copying, dissemination, distribution, disclosure or forwarding of this communication is strictly prohibited. If you have received this communication in error, please contact the sender immediately and delete it from your system. Thank You.

CONFIDENTIALITY NOTICE
NOTICE: This message is intended for the use of the individual or entity to which it is addressed and may contain information that is confidential, privileged and exempt from disclosure under applicable law. If the reader of this message is not the intended recipient, you are hereby notified that any printing, copying, dissemination, distribution, disclosure or forwarding of this communication is strictly prohibited. If you have received this communication in error, please contact the sender immediately and delete it from your system. Thank You.

CONFIDENTIALITY NOTICE
NOTICE: This message is intended for the use of the individual or entity to which it is addressed and may contain information that is confidential, privileged and exempt from disclosure under applicable law. If the reader of this message is not the intended recipient, you are hereby notified that any printing, copying, dissemination, distribution, disclosure or forwarding of this communication is strictly prohibited. If you have received this communication in error, please contact the sender immediately and delete it from your system. Thank You.

CDC – The Coca-Cola Company

Coca-Cola Technical

Location: The Coca-Cola Company

Date: January 7th 10-11:30am

Objectives:

1) Share updates
2) Identify common areas of focus
3) Discuss potential areas of collaboration

Participants:

CDC

Leandris Liburd, Associate Director for Minority Health and Health Equity, Office of Minority Health and Health Equity

Chloe Tonney, Senior VP External Affairs, CDC Foundation

Reggie Mebane, Director, Office of Diversity Management and Equal Employment Opportunity

TCCC

Beate Lloyd, Senior Director Nutrition, Global Scientific and Regulatory Affairs

Susan Roberts, Director Nutrition, Global Scientific and Regulatory Affairs

Maxime Buyckx, Director Nutrition, Global Scientific and Regulatory Affairs

Agenda:

Welcome and Introductions

TCCC

Vision and Mission of Organization

Company Commitments

Overview of Nutrition and Health Research

CDC

Overview of the Office of Minority Health and Health Equity Priorities

SELMA Project Update

Current/Past collaborations between the CDC Foundation and TCCC

CDC and TCCC

Discussion of Future Opportunities / Public- Private Partnerships

From: Liburd, Leandris C. (CDC/OD/OMHHE)
Sent: 5 Feb 2017 08:33:48 -0500
To: Coleman, Sharon R. (CDC/OD/OMHHE)
Subject: FW: follow up to our recent call

From: McNicholas, Jan [mailto:JMcNicholas@cdcfoundation.org]
Sent: Friday, October 25, 2013 8:11 AM
To: Liburd, Leandris C. (CDC/OD/OMHHE) <lel1@cdc.gov>; suroberts@coca-cola.com
Cc: Tonney, Chloe (CDC cdcfoundation.org) <ctonney@cdcfoundation.org>; McNicholas, Jan <JMcNicholas@cdcfoundation.org>
Subject: RE: follow up to our recent call

Good Morning,

Susan, Chloe's calendar has changed somewhat for December, she is now available the first week of December. Let me know when you are ready to confirm
With a day/time.

Thanks so much and have a great day,

Janis

Janis McNicholas

CDC Foundation
55 Park Place
Suite 400
Atlanta, GA 30303
Direct: (404) 523-1109
Main: (404) 653-0790
Fax: (404) 653-0330
jmcnicholas@cdcfoundation.org
www.cdcfoundation.org

Building innovative partnerships to
advance CDC's 24/7 life-saving work
to protect us all
Combine Federal Campaign No. 10141

From: McNicholas, Jan
Sent: Monday, October 21, 2013 11:28 AM
To: Liburd, Leandris C. (CDC/OD/OMHHE); suroberts@coca-cola.com
Cc: McNicholas, Jan
Subject: RE: follow up to our recent call

In checking Chloe's calendar, as of now she is available both days. The afternoon is also a better time for Chloe.

Thanks so much,

Janis

Janis McNicholas

CDC Foundation
55 Park Place
Suite 400
Atlanta, GA 30303
Direct: (404) 523-1109
Main: (404) 653-0790
Fax: (404) 653-0330
jmcnicholas@cdcfoundation.org
www.cdcfoundation.org

Building innovative partnerships to advance CDC's 24/7 life-saving work to protect us all
Combine Federal Campaign No. 10141

From: Liburd, Leandris C. (CDC/OD/OMHHE) [mailto:lel1@cdc.gov]
Sent: Friday, October 18, 2013 4:20 PM
To: Susan A. Roberts
Cc: Tonney, Chloe; Baker, Gwendolyn (CDC/OD/OMHHE); McNicholas, Jan
Subject: RE: follow up to our recent call

Hi Susan,

The first week in December, any afternoon, is good for me. December 12 & 13 are also good after 12 noon. Gwen can assist you when you are ready to confirm.
I also added Janis McNicholas to this e-mail. She is Chloe's assistant at the CDC Foundation.

Thanks, Leandris

From: Susan A. Roberts [mailto:suroberts@coca-cola.com]
Sent: Friday, October 18, 2013 4:13 PM
To: Liburd, Leandris C. (CDC/OD/OMHHE)
Cc: Tonney, Chloe (CDC cdcfoundation.org); Baker, Gwendolyn (CDC/OD/OMHHE)
Subject: RE: follow up to our recent call

Dear Leandris,
I want to give you an update on our plans for a follow up meeting. There are new people joining the Global Scientific and Regulatory Affairs team as a result of our recent reorganization and we feel it will be important to involve them in our discussion. As a result of this, travel schedules and the Thanksgiving

holidays, we will be looking toward the end early December to set up this meeting. Please let me know if there are preferred dates/times from your end.
Thank you, Susan

From: suroberts@coca-cola.com
Sent: Monday, September 30, 2013 1:25 PM
To: 'Liburd, Leandris C. (CDC/OD/OMHHE)'
Cc: Tonney, Chloe (CDC cdcfoundation.org); Baker, Gwendolyn (CDC/OD/OMHHE)
Subject: RE: follow up to our recent call

Dear Leandris,
Yes, that is correct, I will take the lead on organizing a date for a meeting. I will coordinate with key people internally and once we have several dates identified I will get back to you.
Looking forward to our further discussion, Susan

Susan Roberts, PhD | Global Scientific and Regulatory Affairs | The Coca-Cola Company | Tel 404-676-1330 email: suroberts@coca-cola.com

---

From: Liburd, Leandris C. (CDC/OD/OMHHE) [mailto:lel1@cdc.gov]
Sent: Thursday, September 26, 2013 10:17 AM
To: Susan A. Roberts
Cc: Tonney, Chloe (CDC cdcfoundation.org); Baker, Gwendolyn (CDC/OD/OMHHE)
Subject: follow up to our recent call

Good morning, Susan,

I wanted to confirm that you will take the lead in identifying a date for Chloe and I to come to Coca Cola to provide an overview of OMHHE's priorities, and share other collaborations between the CDC Foundation and Coca Cola.

I am copying Chloe on this email so that we can coordinate calendars to schedule this meeting.

Thanks, and let me know if there is anything I need to do on this end to facilitate our meeting.

Best, Leandris

Leandris C. Liburd, PhD, MPH
Associate Director for Minority Health and Health Equity
Office of Minority Health and Health Equity
Centers for Disease Control and Prevention
4770 Buford Hwy, Mailstop K-77
Atlanta, GA 30341
770-488-8343
770-488-8160 (FAX)

CONFIDENTIALITY NOTICE
NOTICE: This message is intended for the use of the individual or entity to which it is addressed and may contain information that is confidential, privileged and exempt from disclosure under applicable law. If the reader of this message is not the intended recipient, you are hereby notified that any printing, copying, dissemination, distribution, disclosure or forwarding of this communication is strictly prohibited. If you have received this communication in error, please contact the sender immediately and delete it from your system. Thank You.

From: Liburd, Leandris C. (CDC/OD/OMHHE)
Sent: 5 Feb 2017 08:24:53 -0500
To: Coleman, Sharon R. (CDC/OD/OMHHE)
Subject: FW: Obesity paper on low-calorie sweetnened beverages vs water
Attachments: Peters et al 2014 Obesity LNCS vs Water on weight loss.pdf

---

From: Susan A. Roberts [mailto:suroberts@coca-cola.com]
Sent: Friday, June 20, 2014 2:16 PM
To: Liburd, Leandris C. (CDC/OD/OMHHE) <lel1@cdc.gov>
Subject: Obesity paper on low-calorie sweetnened beverages vs water

Leandris,
Glad to share this publication with you.
Please let me know if you have any questions. As mentioned, the weight maintenance study will be finished the end of the year.
Best wishes, Susan

Susan Roberts, PhD | Global Scientific and Regulatory Affairs | The Coca-Cola Company | Tel 404-676-1330 email: suroberts@coca-cola.com

---

CONFIDENTIALITY NOTICE
NOTICE: This message is intended for the use of the individual or entity to which it is addressed and may contain information that is confidential, privileged and exempt from disclosure under applicable law. If the reader of this message is not the intended recipient, you are hereby notified that any printing, copying, dissemination, distribution, disclosure or forwarding of this communication is strictly prohibited. If you have received this communication in error, please contact the sender immediately and delete it from your system. Thank You.

Obesity

The Effects of Water and Non-Nutritive Sweetened Beverages on Weight Loss During a 12-week Weight Loss Treatment Program

John C. Peters[1], Holly R. Wyatt[1], Gary D. Foster[2], Zhaoxing Pan[1], Alexis C. Wojtanowski[2], Stephanie S. Vander Veur[2], Sharon J. Herring[2], Carrie Brill[1] and James O. Hill[1]

(b)(4)

From: Liburd, Leandris C. (CDC/OD/OMHHE)
Sent: 5 Feb 2017 08:11:18 -0500
To: Coleman, Sharon R. (CDC/OD/OMHHE)
Subject: FW: Obesity paper on low-calorie sweetnened beverages vs water
Attachments: Peters et al 2014 Obesity LNCS vs Water on weight loss.pdf

---

From: Liburd, Leandris C. (CDC/OD/OMHHE)
Sent: Monday, June 23, 2014 9:50 AM
To: Collins, Janet L. (CDC/ONDIEH/NCCDPHP) <jlc1@cdc.gov>; Ryan, Kevin A. (CDC/ONDIEH/NCCDPHP) <kir0@cdc.gov>
Cc: Hall, Mary E. (CDC/OD/OMHHE) <moh4@cdc.gov>
Subject: FW: Obesity paper on low-calorie sweetnened beverages vs water

FYI… thanks for a rich discussion Friday… Leandris

From: Susan A. Roberts [mailto:suroberts@coca-cola.com]
Sent: Friday, June 20, 2014 2:16 PM
To: Liburd, Leandris C. (CDC/OD/OMHHE)
Subject: Obesity paper on low-calorie sweetnened beverages vs water

Leandris,
Glad to share this publication with you.
Please let me know if you have any questions. As mentioned, the weight maintenance study will be finished the end of the year.
Best wishes, Susan

Susan Roberts, PhD | Global Scientific and Regulatory Affairs | The Coca-Cola Company | Tel 404-676-1330 email: suroberts@coca-cola.com

---

CONFIDENTIALITY NOTICE
NOTICE: This message is intended for the use of the individual or entity to which it is addressed and may contain information that is confidential, privileged and exempt from disclosure under applicable law. If the reader of this message is not the intended recipient, you are hereby notified that any printing, copying, dissemination, distribution, disclosure or forwarding of this communication is strictly prohibited. If you have received this communication in error, please contact the sender immediately and delete it from your system. Thank You.

Obesity

The Effects of Water and Non-Nutritive Sweetened Beverages on Weight Loss During a 12-week Weight Loss Treatment Program

John C. Peters[1], Holly R. Wyatt[1], Gary D. Foster[2], Zhaoxing Pan[1], Alexis C. Wojtanowski[2], Stephanie S. Vander Veur[2], Sharon J. Herring[2], Carrie Brill[1] and James O. Hill[1]

[redacted]

(b)(4)

From: Liburd, Leandris C. (CDC/OD/OMHHE)
Sent: 5 Feb 2017 08:10:44 -0500
To: Coleman, Sharon R. (CDC/OD/OMHHE)
Subject: FW: So great meeting you!

From: Karima Kendall [mailto:kakendall@coca-cola.com]
Sent: Tuesday, June 24, 2014 9:48 AM
To: Liburd, Leandris C. (CDC/OD/OMHHE) <lel1@cdc.gov>
Subject: So great meeting you!

Good morning, Dr. Liburd!

I just wanted to send you a little note to let you know how great it was to meet you and your colleagues. I am not sure if I mentioned it, but I am new to the area, and I am looking forward to meeting as many people as I can. I would love an opportunity to chat with you informally, so that I may learn more about you, your career and your current position.

Please let me know if you are willing to meet with me for dinner or brunch sometime, so we can have a little “girl talk”☺

Thanks so much, and I hope to hear from you soon,

Karima

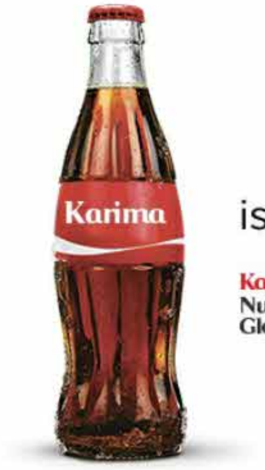

is happy to Share a Coke. with you

Karima A. Kendall, PhD, RD, LDN
Nutrition Center of Excellence,
Global Scientific and Regulatory Affairs

kakendall@coca-cola
T (404) 515 - 1603
F (404) 598 - 1603

CONFIDENTIALITY NOTICE
NOTICE: This message is intended for the use of the individual or entity to which it is addressed and may contain information that is confidential, privileged and exempt from disclosure under applicable law. If the reader of this message is not the intended recipient, you are hereby notified that any printing, copying, dissemination, distribution, disclosure or forwarding of this communication is strictly prohibited. If you have received this communication in error, please contact the sender immediately and delete it from your system. Thank You.

From: Liburd, Leandris C. (CDC/OD/OMHHE)
Sent: 5 Feb 2017 08:09:02 -0500
To: Coleman, Sharon R. (CDC/OD/OMHHE)
Subject: FW: Thank you.

From: Collins, Janet L. (CDC/ONDIEH/NCCDPHP)
Sent: Wednesday, June 25, 2014 8:46 AM
To: Beate Lloyd <belloyd@coca-cola.com>
Cc: Susan A. Roberts <suroberts@coca-cola.com>; Karima Kendall <kakendall@coca-cola.com>; Ryan, Kevin A. (CDC/ONDIEH/NCCDPHP) <kir0@cdc.gov>; Liburd, Leandris C. (CDC/OD/OMHHE) <lel1@cdc.gov>
Subject: RE: Thank you.

Beate,
Thanks again for meeting with us. We enjoyed it and learned a lot. I would be happy to set up a meeting with my fortification group either here or at the Coca-Cola offices. We also look forward to thinking about other areas of mutual interest and continuing our discussions.

Thanks,
Janet

Janet Collins, Ph.D.
Director, Division of Nutrition, Physical Activity, and Obesity
National Center for Chronic Disease Prevention and Health Promotion, CDC
770-488-6042 (w); 404-409-0522 (c); jcollins@cdc.gov

25 HEALTHY LIVING FOR ALL YEARS
National Center for
Chronic Disease Prevention
and Health Promotion

From: Beate Lloyd [mailto:belloyd@coca-cola.com]
Sent: Tuesday, June 24, 2014 6:39 PM
To: Collins, Janet L. (CDC/ONDIEH/NCCDPHP); Ryan, Kevin A. (CDC/ONDIEH/NCCDPHP); Liburd, Leandris C. (CDC/OD/OMHHE)
Cc: Susan A. Roberts; Karima Kendall
Subject: Thank you.

Dear Janet,
I would like to thank you, Kevin and Leandris very much for taking the time to meet with us. It was very helpful to understand your areas of focus and where we may have mutual interests. There are clearly areas where we can work collaboratively and share insights to advance the work in prevention of obesity and inform of the consumer of choices.

We valued getting to know you and your team better and enjoyed the rich discussion relating to your main initiatives. Susan will be sharing with you further the work on the low and no calorie beverage research and will follow-up as more of the data become publically available. We can also forward the papers on the scientific method and interpretation of the epidemiological studies as per our discussion and impact of heterogeneity.

It would be helpful to have another meeting in the future to follow-up on the key discussions on methods and interventions, especially with regards to the fortification programs and grocery channels. Thank you again very much for your interest and time.

Warmest regards,
Beate

Beate B. Lloyd PhD RD LD
Senior Director Nutrition COE
Global Scientific and Regulatory Affairs
The Coca-Cola Company
P: (404) 676-2149
C: (404) 545-3141

CONFIDENTIALITY NOTICE
NOTICE: This message is intended for the use of the individual or entity to which it is addressed and may contain information that is confidential, privileged and exempt from disclosure under applicable law. If the reader of this message is not the intended recipient, you are hereby notified that any printing, copying, dissemination, distribution, disclosure or forwarding of this communication is strictly prohibited. If you have received this communication in error, please contact the sender immediately and delete it from your system. Thank You.

From: Liburd, Leandris C. (CDC/OD/OMHHE)
Sent: 5 Feb 2017 08:09:30 -0500
To: Coleman, Sharon R. (CDC/OD/OMHHE)
Subject: FW: Thank you.
Attachments: Miller 2014 AJCN Meta-analysis o fLow-Calorie Sweeteners Body Weight.pdf, Anton 2014 Obesity editorial.pdf, Peters et al 2014 Obesity LNCS vs Water on weight loss.pdf, Heterogeneity Final March 28 2014.docx

---

From: Susan A. Roberts [mailto:suroberts@coca-cola.com]
Sent: Tuesday, June 24, 2014 10:27 PM
To: Beate Lloyd <belloyd@coca-cola.com>; Collins, Janet L. (CDC/ONDIEH/NCCDPHP) <jlc1@cdc.gov>; Ryan, Kevin A. (CDC/ONDIEH/NCCDPHP) <kir0@cdc.gov>; Liburd, Leandris C. (CDC/OD/OMHHE) <lel1@cdc.gov>
Cc: Karima Kendall <kakendall@coca-cola.com>
Subject: RE: Thank you.

Dear Janet, Kevin and Leandris,
I was very glad to be able to participate in the meeting by phone on Friday and also look forward to on-going discussions with you to identify areas of potential collaboration and information sharing.
In follow up to Beate's note below, please find attached two recent publications on LNCS as well as a draft manuscript on heterogeneity in meta-analyses with suggested approaches to help the non-expert better interpret the outcome statistic.

1) Low-calorie sweeteners vs water during weight loss study (Jim Hill from Univ of Colorado and Gary Foster from Temple Univ, both former presidents of The Obesity Society, were the co-PIs). As mentioned on the call, this is phase I of a two-part study. Phase 2 , the maintenance phase (additional 9 months) will be completed by end of year. Leandris, this is the paper I sent to you on Friday. I have also attached the editorial associated with the paper for an additional perspective. We plan to address many of the outstanding questions of mechanism/appetite in future studies.
2) Meta-analysis of low-calorie sweeteners and weight – the analysis is done on both RCTs and prospective population studies. The findings show a modest but significant benefit of low-calorie sweeteners in weight loss and the effect is largely driven by the beverage studies. It also provides additional support for the view that the positive associations between diet beverages and weight in the epidemiological studies is likely the result of reverse causality.
3) The heterogeneity methods paper. This paper has been accepted in the journal Systematic Reviews. Therefore please treat as confidential until it is published.

I would be glad to discuss any of this further.

Best wishes, Susan

From: Beate Lloyd
Sent: Tuesday, June 24, 2014 6:39 PM
To: jcollins@cdc.gov; kir0@cdc.gov; Liburd, Leandris C. (CDC/OD/OMHHE)

Cc: Susan A. Roberts; Karima Kendall
Subject: Thank you.

Dear Janet,
I would like to thank you, Kevin and Leandris very much for taking the time to meet with us. It was very helpful to understand your areas of focus and where we may have mutual interests. There are clearly areas where we can work collaboratively and share insights to advance the work in prevention of obesity and inform of the consumer of choices.

We valued getting to know you and your team better and enjoyed the rich discussion relating to your main initiatives. Susan will be sharing with you further the work on the low and no calorie beverage research and will follow-up as more of the data become publically available. We can also forward the papers on the scientific method and interpretation of the epidemiological studies as per our discussion and impact of heterogeneity.

It would be helpful to have another meeting in the future to follow-up on the key discussions on methods and interventions, especially with regards to the fortification programs and grocery channels. Thank you again very much for your interest and time.

Warmest regards,
Beate

Beate B. Lloyd PhD RD LD
Senior Director Nutrition COE
Global Scientific and Regulatory Affairs
The Coca-Cola Company
P: (404) 676-2149
C: (404) 545-3141

CONFIDENTIALITY NOTICE
NOTICE: This message is intended for the use of the individual or entity to which it is addressed and may contain information that is confidential, privileged and exempt from disclosure under applicable law. If the reader of this message is not the intended recipient, you are hereby notified that any printing, copying, dissemination, distribution, disclosure or forwarding of this communication is strictly prohibited. If you have received this communication in error, please contact the sender immediately and delete it from your system. Thank You.

AJCN. First published ahead of print June 18, 2014 as doi: 10.3945/ajcn.113.082826.

Low-calorie sweeteners and body weight and composition: a meta-analysis of randomized controlled trials and prospective cohort studies[1–3]

Paige E Miller and Vanessa Perez

(b)(4)

Copyright (C) 2014 by the American Society for Nutrition

Obesity

Can Non-Nutritive Sweeteners Enhance Outcomes of Weight Loss Interventions?

Stephen D. Anton[1,2]

(b)(4)

The Effects of Water and Non-Nutritive Sweetened Beverages on Weight Loss During a 12-week Weight Loss Treatment Program

John C. Peters[1], Holly R. Wyatt[1], Gary D. Foster[2], Zhaoxing Pan[1], Alexis C. Wojtanowski[2], Stephanie S. Vander Veur[2], Sharon J. Herring[2], Carrie Brill[1] and James O. Hill[1]

(b)(4)

Evidence-based mapping of design heterogeneity prior to meta-analysis: a systematic review and research synthesis

March 28, 2014

1. Michelle D. Althuis, PhD – corresponding author
EpiContext
1115 East Capitol Street SE
Washington, DC 20003
Michelle@EpiContext.com

2. Douglas L. Weed, MD, MPH, PhD
DLW Consulting Services, LLC
1302 N. Oak Forest Rd.
Salt Lake City, UT 84103
Douglaslweed@aol.com

3. Cara L. Frankenfeld, PhD
Department of Global and Community Health
George Mason University
4400 University Drive, MS 5B7
Fairfax, VA 22030
cfranken@gmu.edu

From: Liburd, Leandris C. (CDC/OD/OMHHE)
Sent: 5 Feb 2017 08:22:24 -0500
To: Coleman, Sharon R. (CDC/OD/OMHHE)
Subject: FW: Thank you.
Attachments: Miller 2014 AJCN Meta-analysis o fLow-Calorie Sweeteners Body Weight.pdf, Anton 2014 Obesity editorial.pdf, Peters et al 2014 Obesity LNCS vs Water on weight loss.pdf, Heterogeneity Final March 28 2014.docx

---

From: Liburd, Leandris C. (CDC/OD/OMHHE)
Sent: Thursday, June 26, 2014 11:41 AM
To: Arias, Ileana (CDC/OD) <iaa4@cdc.gov>
Subject: FW: Thank you.

Fyi…

From: Susan A. Roberts [mailto:suroberts@coca-cola.com]
Sent: Tuesday, June 24, 2014 10:27 PM
To: Beate Lloyd; Collins, Janet L. (CDC/ONDIEH/NCCDPHP); Ryan, Kevin A. (CDC/ONDIEH/NCCDPHP); Liburd, Leandris C. (CDC/OD/OMHHE)
Cc: Karima Kendall
Subject: RE: Thank you.

Dear Janet, Kevin and Leandris,
I was very glad to be able to participate in the meeting by phone on Friday and also look forward to on-going discussions with you to identify areas of potential collaboration and information sharing.
In follow up to Beate's note below, please find attached two recent publications on LNCS as well as a draft manuscript on heterogeneity in meta-analyses with suggested approaches to help the non-expert better interpret the outcome statistic.

1) Low-calorie sweeteners vs water during weight loss study (Jim Hill from Univ of Colorado and Gary Foster from Temple Univ, both former presidents of The Obesity Society, were the co-PIs). As mentioned on the call, this is phase I of a two-part study. Phase 2 , the maintenance phase (additional 9 months) will be completed by end of year. Leandris, this is the paper I sent to you on Friday. I have also attached the editorial associated with the paper for an additional perspective. We plan to address many of the outstanding questions of mechanism/appetite in future studies.
2) Meta-analysis of low-calorie sweeteners and weight – the analysis is done on both RCTs and prospective population studies. The findings show a modest but significant benefit of low-calorie sweeteners in weight loss and the effect is largely driven by the beverage studies. It also provides additional support for the view that the positive associations between diet beverages and weight in the epidemiological studies is likely the result of reverse causality.
3) The heterogeneity methods paper. This paper has been accepted in the journal Systematic Reviews. Therefore please treat as confidential until it is published.

I would be glad to discuss any of this further.

Best wishes, Susan

From: Beate Lloyd
Sent: Tuesday, June 24, 2014 6:39 PM
To: jcollins@cdc.gov; kir0@cdc.gov; Liburd, Leandris C. (CDC/OD/OMHHE)
Cc: Susan A. Roberts; Karima Kendall
Subject: Thank you.

Dear Janet,
I would like to thank you, Kevin and Leandris very much for taking the time to meet with us. It was very helpful to understand your areas of focus and where we may have mutual interests. There are clearly areas where we can work collaboratively and share insights to advance the work in prevention of obesity and inform of the consumer of choices.

We valued getting to know you and your team better and enjoyed the rich discussion relating to your main initiatives. Susan will be sharing with you further the work on the low and no calorie beverage research and will follow-up as more of the data become publically available. We can also forward the papers on the scientific method and interpretation of the epidemiological studies as per our discussion and impact of heterogeneity.

It would be helpful to have another meeting in the future to follow-up on the key discussions on methods and interventions, especially with regards to the fortification programs and grocery channels. Thank you again very much for your interest and time.

Warmest regards,
Beate

Beate B. Lloyd PhD RD LD
Senior Director Nutrition COE
Global Scientific and Regulatory Affairs
The Coca-Cola Company
P: (404) 676-2149
C: (404) 545-3141

CONFIDENTIALITY NOTICE
NOTICE: This message is intended for the use of the individual or entity to which it is addressed and may contain information that is confidential, privileged and exempt from disclosure under applicable law. If the reader of this message is not the intended recipient, you are hereby notified that any printing, copying, dissemination, distribution, disclosure or forwarding of this communication is strictly prohibited. If you have received this communication in error, please contact the sender immediately and delete it from your system. Thank You.

AJCN. First published ahead of print June 18, 2014 as doi: 10.3945/ajcn.113.082826.

Low-calorie sweeteners and body weight and composition: a meta-analysis of randomized controlled trials and prospective cohort studies[1–3]

Paige E Miller and Vanessa Perez

[redacted]

(b)(4)

Copyright (C) 2014 by the American Society for Nutrition

Obesity

Can Non-Nutritive Sweeteners Enhance Outcomes of Weight Loss Interventions?

Stephen D. Anton[1,2]

(b)(4)

Obesity

The Effects of Water and Non-Nutritive Sweetened Beverages on Weight Loss During a 12-week Weight Loss Treatment Program

John C. Peters[1], Holly R. Wyatt[1], Gary D. Foster[2], Zhaoxing Pan[1], Alexis C. Wojtanowski[2], Stephanie S. Vander Veur[2], Sharon J. Herring[2], Carrie Brill[1] and James O. Hill[1]

(b)(4)

Evidence-based mapping of design heterogeneity prior to meta-analysis: a systematic review and research synthesis

March 28, 2014

1. Michelle D. Althuis, PhD – corresponding author
EpiContext
1115 East Capitol Street SE
Washington, DC 20003
Michelle@EpiContext.com

2. Douglas L. Weed, MD, MPH, PhD
DLW Consulting Services, LLC
1302 N. Oak Forest Rd.
Salt Lake City, UT 84103
Douglaslweed@aol.com

3. Cara L. Frankenfeld, PhD
Department of Global and Community Health
George Mason University
4400 University Drive, MS 5B7
Fairfax, VA 22030
cfranken@gmu.edu

From: Liburd, Leandris C. (CDC/OD/OMHHE)
Sent: 5 Feb 2017 08:23:02 -0500
To: Coleman, Sharon R. (CDC/OD/OMHHE)
Subject: FW: Thank you.
Attachments: image001.jpg

From: Susan A. Roberts [mailto:suroberts@coca-cola.com]
Sent: Wednesday, June 25, 2014 10:07 AM
To: Collins, Janet L. (CDC/ONDIEH/NCCDPHP) <jlc1@cdc.gov>
Cc: Beate Lloyd <belloyd@coca-cola.com>; Ryan, Kevin A. (CDC/ONDIEH/NCCDPHP) <kir0@cdc.gov>; Liburd, Leandris C. (CDC/OD/OMHHE) <lel1@cdc.gov>; Karima Kendall <kakendall@coca-cola.com>; Galuska, Deborah A. (CDC/ONDIEH/NCCDPHP) <dbg6@cdc.gov>
Subject: Re: Thank you.

Janet, thank you.
Deb, feel free to reach our to me to discuss any of this or to learn more about our current research efforts in this area.
Best wishes, Susan

On Jun 25, 2014, at 8:48 AM, "Collins, Janet L. (CDC/ONDIEH/NCCDPHP)" <jlc1@cdc.gov> wrote:

> Thank you so much for these resources. I am copying our Associate Director of Science, Deb Galuska, to help us make optimal use of these resources.
>
> Thanks!
> Janet
>
> Janet Collins, Ph.D.
> Director, Division of Nutrition, Physical Activity, and Obesity
> National Center for Chronic Disease Prevention and Health Promotion, CDC
> 770-488-6042 (w); 404-409-0522 (c); jcollins@cdc.gov
>
> <image001.jpg>
>
> ---
>
> **From:** Susan A. Roberts [mailto:suroberts@coca-cola.com]
> **Sent:** Tuesday, June 24, 2014 10:27 PM
> **To:** Beate Lloyd; Collins, Janet L. (CDC/ONDIEH/NCCDPHP); Ryan, Kevin A. (CDC/ONDIEH/NCCDPHP); Liburd, Leandris C. (CDC/OD/OMHHE)
> **Cc:** Karima Kendall
> **Subject:** RE: Thank you.
>
> Dear Janet, Kevin and Leandris,
> I was very glad to be able to participate in the meeting by phone on Friday and also look forward to on-going discussions with you to identify areas of potential collaboration and information sharing.

In follow up to Beate's note below, please find attached two recent publications on LNCS as well as a draft manuscript on heterogeneity in meta-analyses with suggested approaches to help the non-expert better interpret the outcome statistic.

1) Low-calorie sweeteners vs water during weight loss study (Jim Hill from Univ of Colorado and Gary Foster from Temple Univ, both former presidents of The Obesity Society, were the co-PIs). As mentioned on the call, this is phase I of a two-part study. Phase 2 , the maintenance phase (additional 9 months) will be completed by end of year. Leandris, this is the paper I sent to you on Friday. I have also attached the editorial associated with the paper for an additional perspective. We plan to address many of the outstanding questions of mechanism/appetite in future studies.
2) Meta-analysis of low-calorie sweeteners and weight – the analysis is done on both RCTs and prospective population studies. The findings show a modest but significant benefit of low-calorie sweeteners in weight loss and the effect is largely driven by the beverage studies. It also provides additional support for the view that the positive associations between diet beverages and weight in the epidemiological studies is likely the result of reverse causality.
3) The heterogeneity methods paper. This paper has been accepted in the journal Systematic Reviews. Therefore please treat as confidential until it is published.

I would be glad to discuss any of this further.

Best wishes, Susan

From: Beate Lloyd
Sent: Tuesday, June 24, 2014 6:39 PM
To: jcollins@cdc.gov; kir0@cdc.gov; Liburd, Leandris C. (CDC/OD/OMHHE)
Cc: Susan A. Roberts; Karima Kendall
Subject: Thank you.

Dear Janet,
I would like to thank you, Kevin and Leandris very much for taking the time to meet with us. It was very helpful to understand your areas of focus and where we may have mutual interests. There are clearly areas where we can work collaboratively and share insights to advance the work in prevention of obesity and inform of the consumer of choices.

We valued getting to know you and your team better and enjoyed the rich discussion relating to your main initiatives. Susan will be sharing with you further the work on the low and no calorie beverage research and will follow-up as more of the data become publically available. We can also forward the papers on the scientific method and interpretation of the epidemiological studies as per our discussion and impact of heterogeneity.

It would be helpful to have another meeting in the future to follow-up on the key discussions on methods and interventions, especially with regards to the fortification programs and grocery channels. Thank you again very much for your interest and time.

Warmest regards,
Beate

Beate B. Lloyd PhD RD LD
Senior Director Nutrition COE
Global Scientific and Regulatory Affairs
The Coca-Cola Company
P: (404) 676-2149
C: (404) 545-3141

CONFIDENTIALITY NOTICE
NOTICE: This message is intended for the use of the individual or entity to which it is addressed and may contain information that is confidential, privileged and exempt from disclosure under applicable law. If the reader of this message is not the intended recipient, you are hereby notified that any printing, copying, dissemination, distribution, disclosure or forwarding of this communication is strictly prohibited. If you have received this communication in error, please contact the sender immediately and delete it from your system. Thank You.

CONFIDENTIALITY NOTICE
NOTICE: This message is intended for the use of the individual or entity to which it is addressed and may contain information that is confidential, privileged and exempt from disclosure under applicable law. If the reader of this message is not the intended recipient, you are hereby notified that any printing, copying, dissemination, distribution, disclosure or forwarding of this communication is strictly prohibited. If you have received this communication in error, please contact the sender immediately and delete it from your system. Thank You.

25 HEALTHY LIVING FOR ALL YEARS

National Center for
Chronic Disease Prevention
and Health Promotion

From: Liburd, Leandris C. (CDC/OD/OMHHE)
Sent: 5 Feb 2017 08:23:56 -0500
To: Coleman, Sharon R. (CDC/OD/OMHHE)
Subject: FW: Thank you.

From: Collins, Janet L. (CDC/ONDIEH/NCCDPHP)
Sent: Wednesday, June 25, 2014 8:48 AM
To: Susan A. Roberts <suroberts@coca-cola.com>; Beate Lloyd <belloyd@coca-cola.com>; Ryan, Kevin A. (CDC/ONDIEH/NCCDPHP) <kir0@cdc.gov>; Liburd, Leandris C. (CDC/OD/OMHHE) <lel1@cdc.gov>
Cc: Karima Kendall <kakendall@coca-cola.com>; Galuska, Deborah A. (CDC/ONDIEH/NCCDPHP) <dbg6@cdc.gov>
Subject: RE: Thank you.

Thank you so much for these resources. I am copying our Associate Director of Science, Deb Galuska, to help us make optimal use of these resources.

Thanks!
Janet

Janet Collins, Ph.D.
Director, Division of Nutrition, Physical Activity, and Obesity
National Center for Chronic Disease Prevention and Health Promotion, CDC
770-488-6042 (w); 404-409-0522 (c); jcollins@cdc.gov

25 HEALTHY LIVING FOR ALL YEARS
National Center for Chronic Disease Prevention and Health Promotion

---

From: Susan A. Roberts [mailto:suroberts@coca-cola.com]
Sent: Tuesday, June 24, 2014 10:27 PM
To: Beate Lloyd; Collins, Janet L. (CDC/ONDIEH/NCCDPHP); Ryan, Kevin A. (CDC/ONDIEH/NCCDPHP); Liburd, Leandris C. (CDC/OD/OMHHE)
Cc: Karima Kendall
Subject: RE: Thank you.

Dear Janet, Kevin and Leandris,
I was very glad to be able to participate in the meeting by phone on Friday and also look forward to on-going discussions with you to identify areas of potential collaboration and information sharing.
In follow up to Beate's note below, please find attached two recent publications on LNCS as well as a draft manuscript on heterogeneity in meta-analyses with suggested approaches to help the non-expert better interpret the outcome statistic.

1) Low-calorie sweeteners vs water during weight loss study (Jim Hill from Univ of Colorado and Gary Foster from Temple Univ, both former presidents of The Obesity Society, were the co-PIs). As mentioned on the call, this is phase I of a two-part study. Phase 2 , the maintenance phase (additional 9 months) will be completed by end of year. Leandris, this is the paper I sent to you on Friday. I have also attached the editorial associated with the paper for an additional perspective. We plan to address many of the outstanding questions of mechanism/appetite in future studies.
2) Meta-analysis of low-calorie sweeteners and weight – the analysis is done on both RCTs and prospective population studies. The findings show a modest but significant benefit of low-calorie sweeteners in weight loss and the effect is largely driven by the beverage studies. It also provides additional support for the view that the positive associations between diet beverages and weight in the epidemiological studies is likely the result of reverse causality.
3) The heterogeneity methods paper. This paper has been accepted in the journal Systematic Reviews. Therefore please treat as confidential until it is published.

I would be glad to discuss any of this further.

Best wishes, Susan

From: Beate Lloyd
Sent: Tuesday, June 24, 2014 6:39 PM
To: jcollins@cdc.gov; kir0@cdc.gov; Liburd, Leandris C. (CDC/OD/OMHHE)
Cc: Susan A. Roberts; Karima Kendall
Subject: Thank you.

Dear Janet,
I would like to thank you, Kevin and Leandris very much for taking the time to meet with us. It was very helpful to understand your areas of focus and where we may have mutual interests. There are clearly areas where we can work collaboratively and share insights to advance the work in prevention of obesity and inform of the consumer of choices.

We valued getting to know you and your team better and enjoyed the rich discussion relating to your main initiatives. Susan will be sharing with you further the work on the low and no calorie beverage research and will follow-up as more of the data become publically available. We can also forward the papers on the scientific method and interpretation of the epidemiological studies as per our discussion and impact of heterogeneity.

It would be helpful to have another meeting in the future to follow-up on the key discussions on methods and interventions, especially with regards to the fortification programs and grocery channels. Thank you again very much for your interest and time.

Warmest regards,
Beate

Beate B. Lloyd PhD RD LD
Senior Director Nutrition COE
Global Scientific and Regulatory Affairs

The Coca-Cola Company
P: (404) 676-2149
C: (404) 545-3141

CONFIDENTIALITY NOTICE
NOTICE: This message is intended for the use of the individual or entity to which it is addressed and may contain information that is confidential, privileged and exempt from disclosure under applicable law. If the reader of this message is not the intended recipient, you are hereby notified that any printing, copying, dissemination, distribution, disclosure or forwarding of this communication is strictly prohibited. If you have received this communication in error, please contact the sender immediately and delete it from your system. Thank You.

From: Liburd, Leandris C. (CDC/OD/OMHHE)
Sent: 5 Feb 2017 08:08:30 -0500
To: Coleman, Sharon R. (CDC/OD/OMHHE)
Subject: FW: Thank you.

From: Collins, Janet L. (CDC/ONDIEH/NCCDPHP)
Sent: Wednesday, June 25, 2014 8:48 AM
To: Susan A. Roberts <suroberts@coca-cola.com>; Beate Lloyd <belloyd@coca-cola.com>; Ryan, Kevin A. (CDC/ONDIEH/NCCDPHP) <kir0@cdc.gov>; Liburd, Leandris C. (CDC/OD/OMHHE) <lel1@cdc.gov>
Cc: Karima Kendall <kakendall@coca-cola.com>; Galuska, Deborah A. (CDC/ONDIEH/NCCDPHP) <dbg6@cdc.gov>
Subject: RE: Thank you.

Thank you so much for these resources. I am copying our Associate Director of Science, Deb Galuska, to help us make optimal use of these resources.

Thanks!
Janet

Janet Collins, Ph.D.
Director, Division of Nutrition, Physical Activity, and Obesity
National Center for Chronic Disease Prevention and Health Promotion, CDC
770-488-6042 (w); 404-409-0522 (c); jcollins@cdc.gov

25 YEARS HEALTHY LIVING FOR ALL
National Center for Chronic Disease Prevention and Health Promotion

---

From: Susan A. Roberts [mailto:suroberts@coca-cola.com]
Sent: Tuesday, June 24, 2014 10:27 PM
To: Beate Lloyd; Collins, Janet L. (CDC/ONDIEH/NCCDPHP); Ryan, Kevin A. (CDC/ONDIEH/NCCDPHP); Liburd, Leandris C. (CDC/OD/OMHHE)
Cc: Karima Kendall
Subject: RE: Thank you.

Dear Janet, Kevin and Leandris,
I was very glad to be able to participate in the meeting by phone on Friday and also look forward to on-going discussions with you to identify areas of potential collaboration and information sharing.
In follow up to Beate's note below, please find attached two recent publications on LNCS as well as a draft manuscript on heterogeneity in meta-analyses with suggested approaches to help the non-expert better interpret the outcome statistic.

1) Low-calorie sweeteners vs water during weight loss study (Jim Hill from Univ of Colorado and Gary Foster from Temple Univ, both former presidents of The Obesity Society, were the co-PIs). As mentioned on the call, this is phase I of a two-part study. Phase 2 , the maintenance phase (additional 9 months) will be completed by end of year. Leandris, this is the paper I sent to you on Friday. I have also attached the editorial associated with the paper for an additional perspective. We plan to address many of the outstanding questions of mechanism/appetite in future studies.
2) Meta-analysis of low-calorie sweeteners and weight – the analysis is done on both RCTs and prospective population studies. The findings show a modest but significant benefit of low-calorie sweeteners in weight loss and the effect is largely driven by the beverage studies. It also provides additional support for the view that the positive associations between diet beverages and weight in the epidemiological studies is likely the result of reverse causality.
3) The heterogeneity methods paper. This paper has been accepted in the journal Systematic Reviews. Therefore please treat as confidential until it is published.

I would be glad to discuss any of this further.

Best wishes, Susan

From: Beate Lloyd
Sent: Tuesday, June 24, 2014 6:39 PM
To: jcollins@cdc.gov; kir0@cdc.gov; Liburd, Leandris C. (CDC/OD/OMHHE)
Cc: Susan A. Roberts; Karima Kendall
Subject: Thank you.

Dear Janet,
I would like to thank you, Kevin and Leandris very much for taking the time to meet with us. It was very helpful to understand your areas of focus and where we may have mutual interests. There are clearly areas where we can work collaboratively and share insights to advance the work in prevention of obesity and inform of the consumer of choices.

We valued getting to know you and your team better and enjoyed the rich discussion relating to your main initiatives. Susan will be sharing with you further the work on the low and no calorie beverage research and will follow-up as more of the data become publically available. We can also forward the papers on the scientific method and interpretation of the epidemiological studies as per our discussion and impact of heterogeneity.

It would be helpful to have another meeting in the future to follow-up on the key discussions on methods and interventions, especially with regards to the fortification programs and grocery channels. Thank you again very much for your interest and time.

Warmest regards,
Beate

Beate B. Lloyd PhD RD LD
Senior Director Nutrition COE
Global Scientific and Regulatory Affairs

The Coca-Cola Company
P: (404) 676-2149
C: (404) 545-3141

CONFIDENTIALITY NOTICE
NOTICE: This message is intended for the use of the individual or entity to which it is addressed and may contain information that is confidential, privileged and exempt from disclosure under applicable law. If the reader of this message is not the intended recipient, you are hereby notified that any printing, copying, dissemination, distribution, disclosure or forwarding of this communication is strictly prohibited. If you have received this communication in error, please contact the sender immediately and delete it from your system. Thank You.
