# Supplementary material for: Public Meets Private: Conversations Between Coca‐Cola and the CDC
Source: Milbank Q. 2019 Jan 29;97(1):74–90. doi: 10.1111/1468-0009.12368 (PMC6422605; doi:10.1111/1468-0009.12368)
Supplement: Supplementary file 4 — Email response (subject: Dangers of sitting) from a Louisiana Public Records Act request to Louisiana State University dated September 19, 2016, regarding communications to or from (or Cc or Bcc) Professor Katzmarzyk or Professor Church with any staff or employees of the Coca‐Cola Company or the American Beverage Association, including any contract related to the International Study of Childhood Obesity, Lifestyle and the Environment (ISCOLE) study. [file MILQ-97-74-s004.pdf]

**From:**	Rhona Applebaum
**To:**	sblair@mailbox.sc.edu; DAllison@ms.soph.uab.edu; Timothy Church; Peter Katzmarzyk; clavie@ochsner.org; K.R.Fox@bristol.ac.uk; GREHAND@mailbox.sc.edu; hamiltonmarc@mac.com; agil@ugr.es; john.duperly@ama.com.co; mxp4@cdc.gov; Robert.E.Sallis@kp.org; william.bird@intelligenthealth.co.uk; Mike Loosemore
**Subject:**	Dangers of sitting
**Date:**	Thursday, March 29, 2012 1:58:41 PM
**Attachments:**	sitting mortality van der Ploeg12.pdf

Hello Everyone…

Another in the 'series' Hot week for PA and the dangers of sedentarism

Seems folks have gotten religion

Now to just amplify these msges.

Conclusions: Prolonged sitting is a risk factor for all cause mortality, independent of physical activity. Public health programs should focus on reducing sitting time in addition to increasing physical activity levels.

Rhona

CONFIDENTIALITY NOTICE
NOTICE: This message is intended for the use of the individual or entity to which it is addressed and may contain information that is confidential, privileged and exempt from disclosure under applicable law. If the reader of this message is not the intended recipient, you are hereby notified that any printing, copying, dissemination, distribution, disclosure or forwarding of this communication is strictly prohibited. If you have received this communication in error, please contact the sender immediately and delete it from your system. Thank You.
