# Supplementary material for: Public Meets Private: Conversations Between Coca‐Cola and the CDC
Source: Milbank Q. 2019 Jan 29;97(1):74–90. doi: 10.1111/1468-0009.12368 (PMC6422605; doi:10.1111/1468-0009.12368)
Supplement: Supplementary file 5 — Email response (subject: RE: ISCOLE news: Confidential) from a Louisiana Public Records Act request to Louisiana State University dated September 19, 2016, regarding communications to or from (or Cc or Bcc) Professor Katzmarzyk or Professor Church with any staff or employees of the Coca‐Cola Company or the American Beverage Association, including any contract related to the International Study of Childhood Obesity, Lifestyle and the Environment (ISCOLE) study. [file MILQ-97-74-s005.pdf]

**From:**	Rhona Applebaum
**To:**	mxp4@cdc.gov; Peter Katzmarzyk; Timothy Church
**Subject:**	Re: ISCOLE news: Confidential
**Date:**	Wednesday, April 04, 2012 7:55:06 AM

Mike--from what was explained to me and during the BtD Symposium--They don't appear to understand the importance of routine and discipline as it relates to the data. It's not a "manana" exercise--it's a 'today' requirement

They didn't seem to get it despite outreach from the PI's

R

----- Original Message -----
From: Pratt, Michael (CDC/ONDIEH/NCCDPHP) [mailto:mxp4@cdc.gov]
Sent: Wednesday, April 04, 2012 08:50 AM
To: 'Peter.Katzmarzyk@pbrc.edu' <Peter.Katzmarzyk@pbrc.edu>; 'Timothy.Church@pbrc.edu' <Timothy.Church@pbrc.edu>; Rhona Applebaum
Subject: ISCOLE news: Confidential

Hi Peter, Tim, and Rhona,
I learned through the grapevine that Mexico is being dropped from ISCOLE. Given the importance of Mexico related to childhood obesity this is of concern. I understand at least some of the issues at play here and believe that there may be solutions. I am also concerned that you may face a similar situation in Brazil. I am happy to discuss in my role as an advisor to ISCOLE.
All the best,
Mike

CONFIDENTIALITY NOTICE
NOTICE: This message is intended for the use of the individual or entity to which it is addressed and may contain information that is confidential, privileged and exempt from disclosure under applicable law. If the reader of this message is not the intended recipient, you are hereby notified that any printing, copying, dissemination, distribution, disclosure or forwarding of this communication is strictly prohibited. If you have received this communication in error, please contact the sender immediately and delete it from your system. Thank You.
